# Supplementary figures and images for: H2BK120ub and its reader RNF169 sequentially regulate replication fork remodeling and stability (part 1 of 2)
Source: EMBO J. 2025 Oct 27;44(22):6598–625. doi: 10.1038/s44318-025-00602-1 (PMC12623888; doi:10.1038/s44318-025-00602-1)

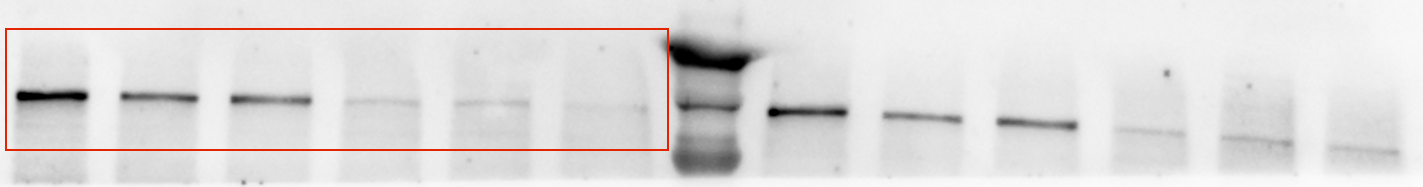

Supplement: Supplementary file 2 — Source data Fig. 1 [file 44318_2025_602_MOESM2_ESM.zip › Fig 1/A/WB RNF20.tiff]

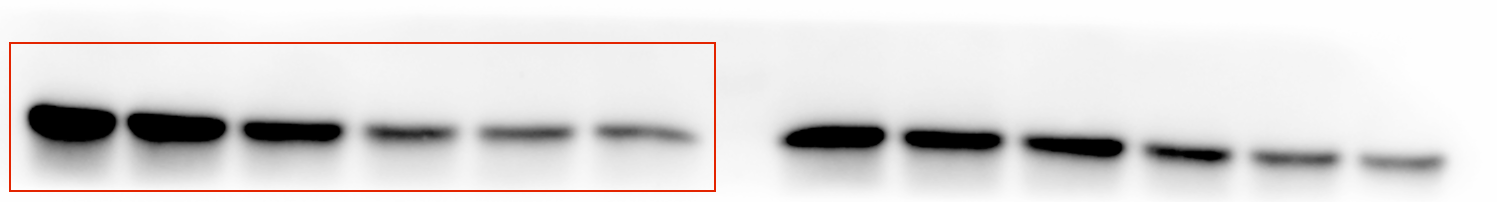

Supplement: Supplementary file 2 — Source data Fig. 1 [file 44318_2025_602_MOESM2_ESM.zip › Fig 1/A/WB H2Bub.tiff]

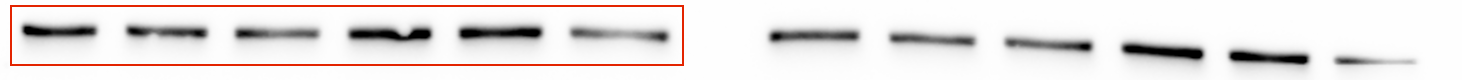

Supplement: Supplementary file 2 — Source data Fig. 1 [file 44318_2025_602_MOESM2_ESM.zip › Fig 1/A/WB Lamin.tiff]

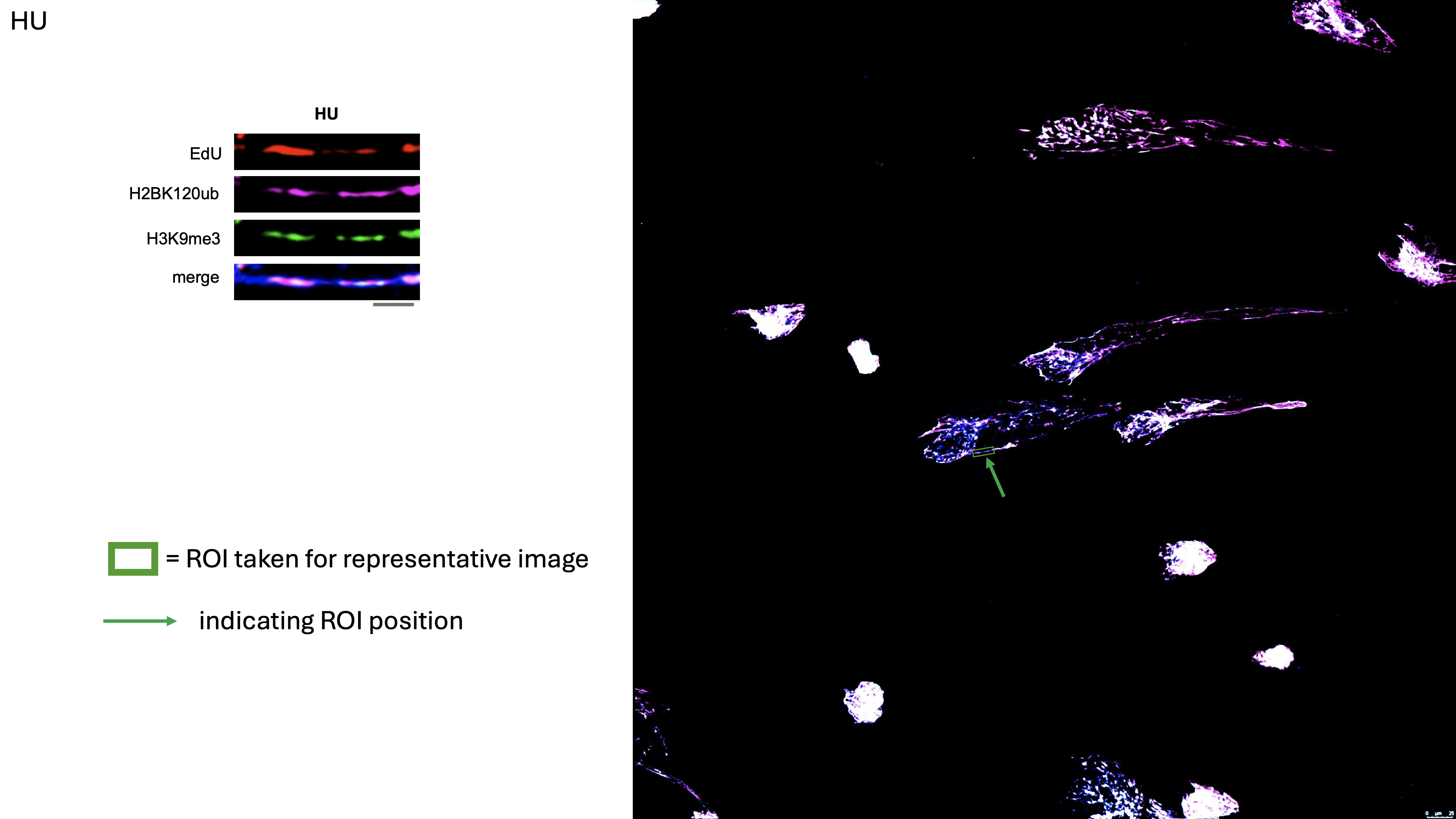

Supplement: Supplementary file 2 — Source data Fig. 1 [file 44318_2025_602_MOESM2_ESM.zip › Fig 1/F/HU.png]

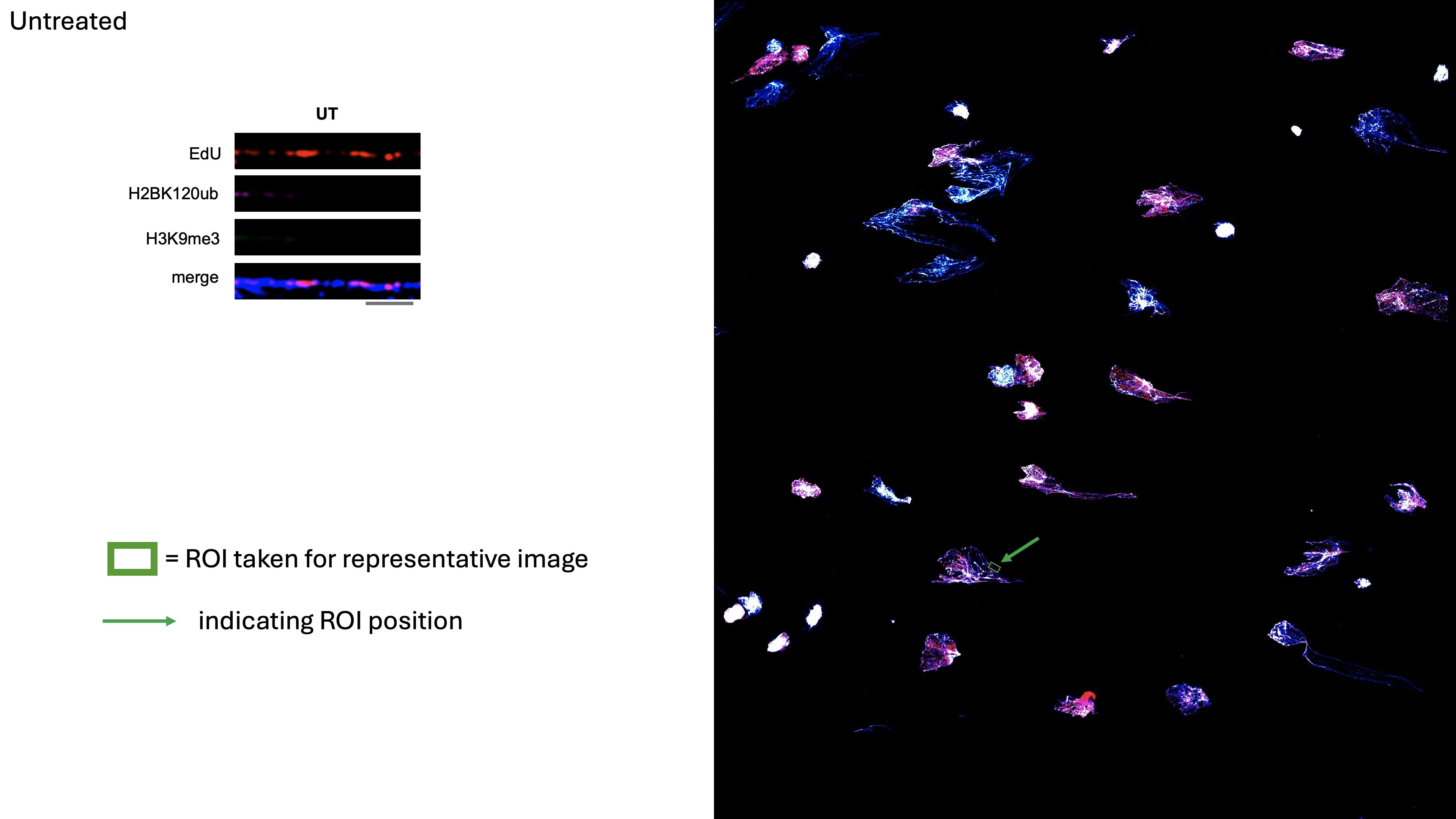

Supplement: Supplementary file 2 — Source data Fig. 1 [file 44318_2025_602_MOESM2_ESM.zip › Fig 1/F/untreated.png]

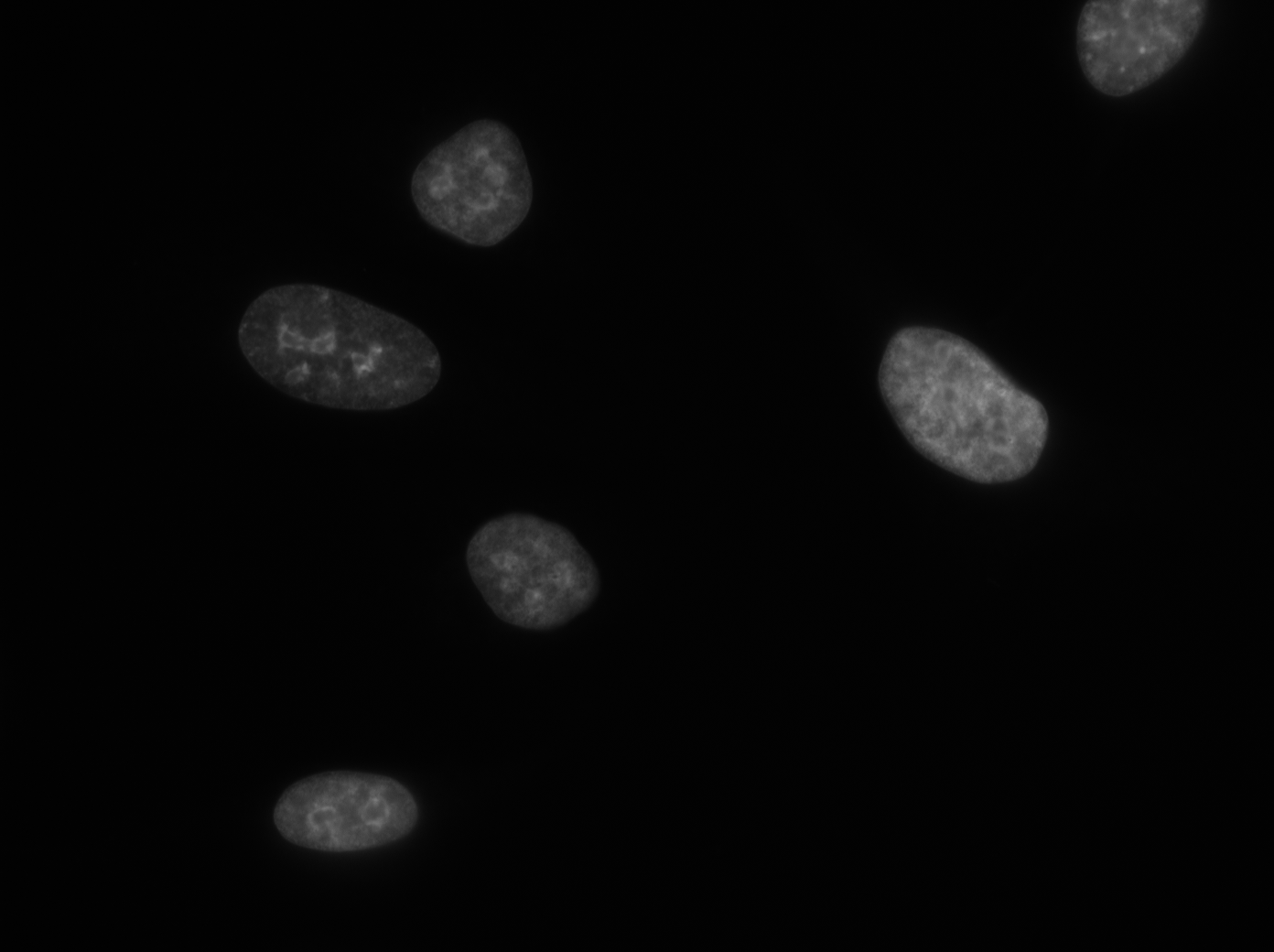

Supplement: Supplementary file 2 — Source data Fig. 1 [file 44318_2025_602_MOESM2_ESM.zip › Fig 1/C/siRNF20 CPT DAPI.tif]

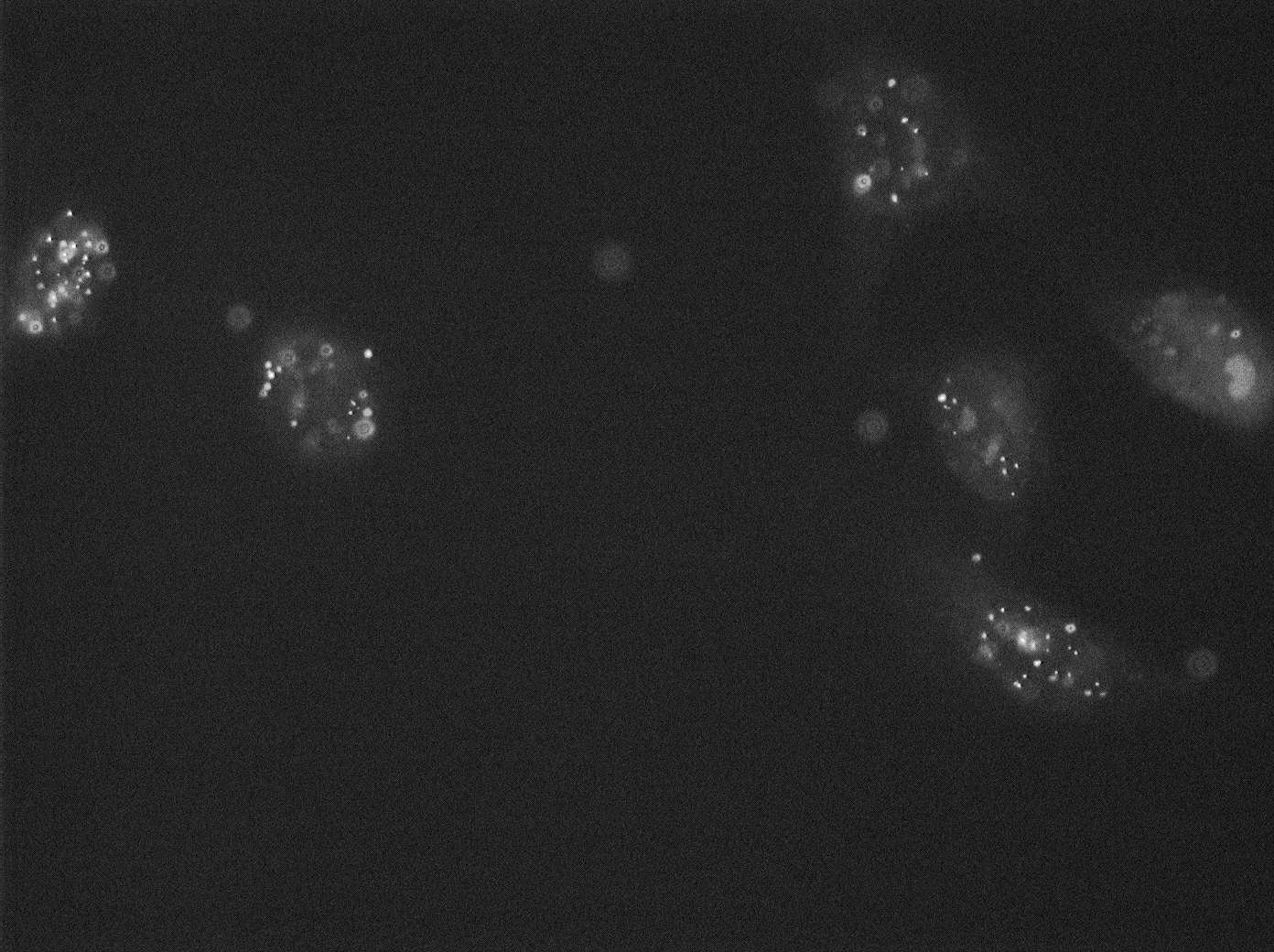

Supplement: Supplementary file 2 — Source data Fig. 1 [file 44318_2025_602_MOESM2_ESM.zip › Fig 1/C/siRNF20 UT SIRF.tif]

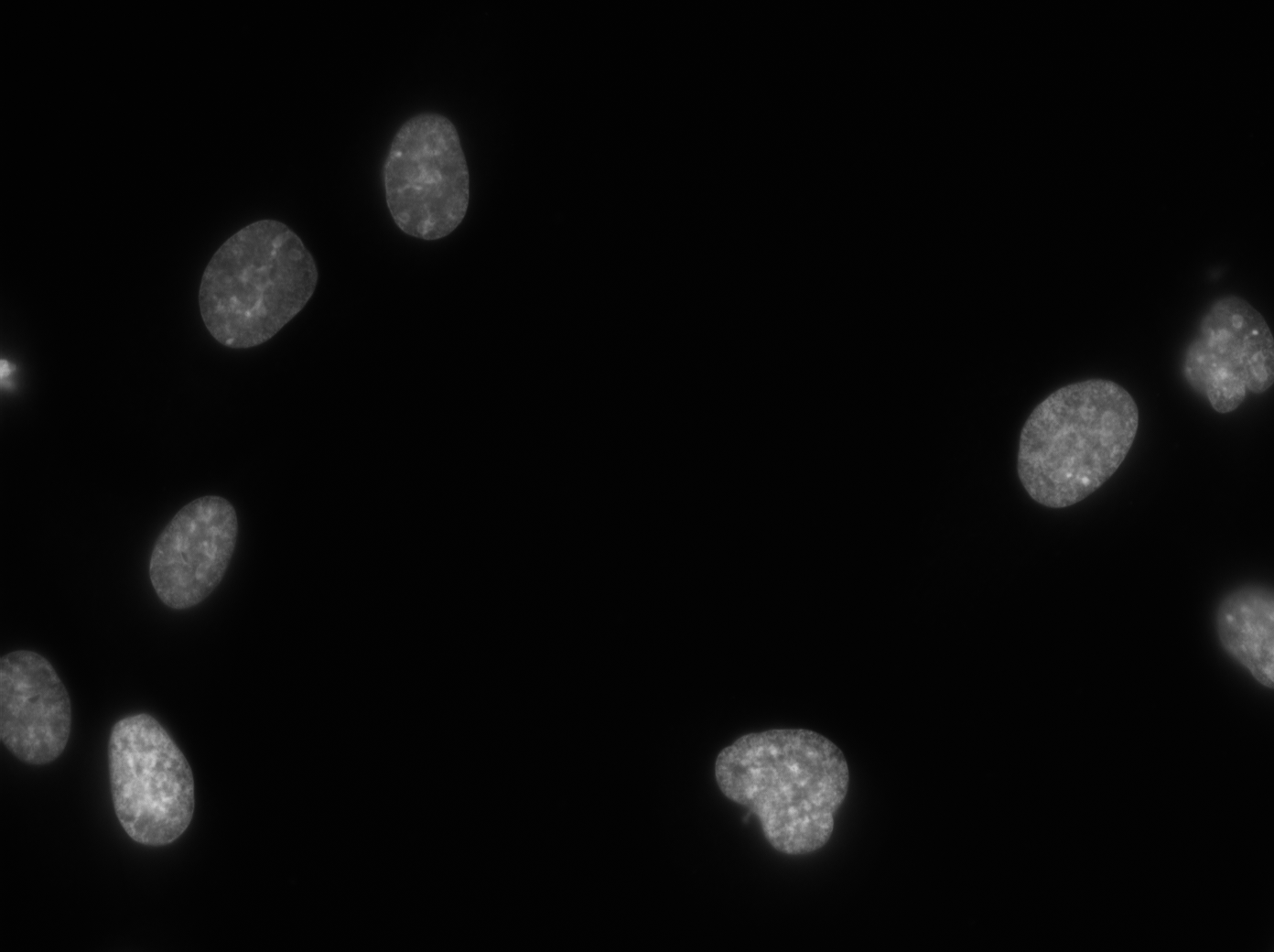

Supplement: Supplementary file 2 — Source data Fig. 1 [file 44318_2025_602_MOESM2_ESM.zip › Fig 1/C/siLuc UT DAPI.tif]

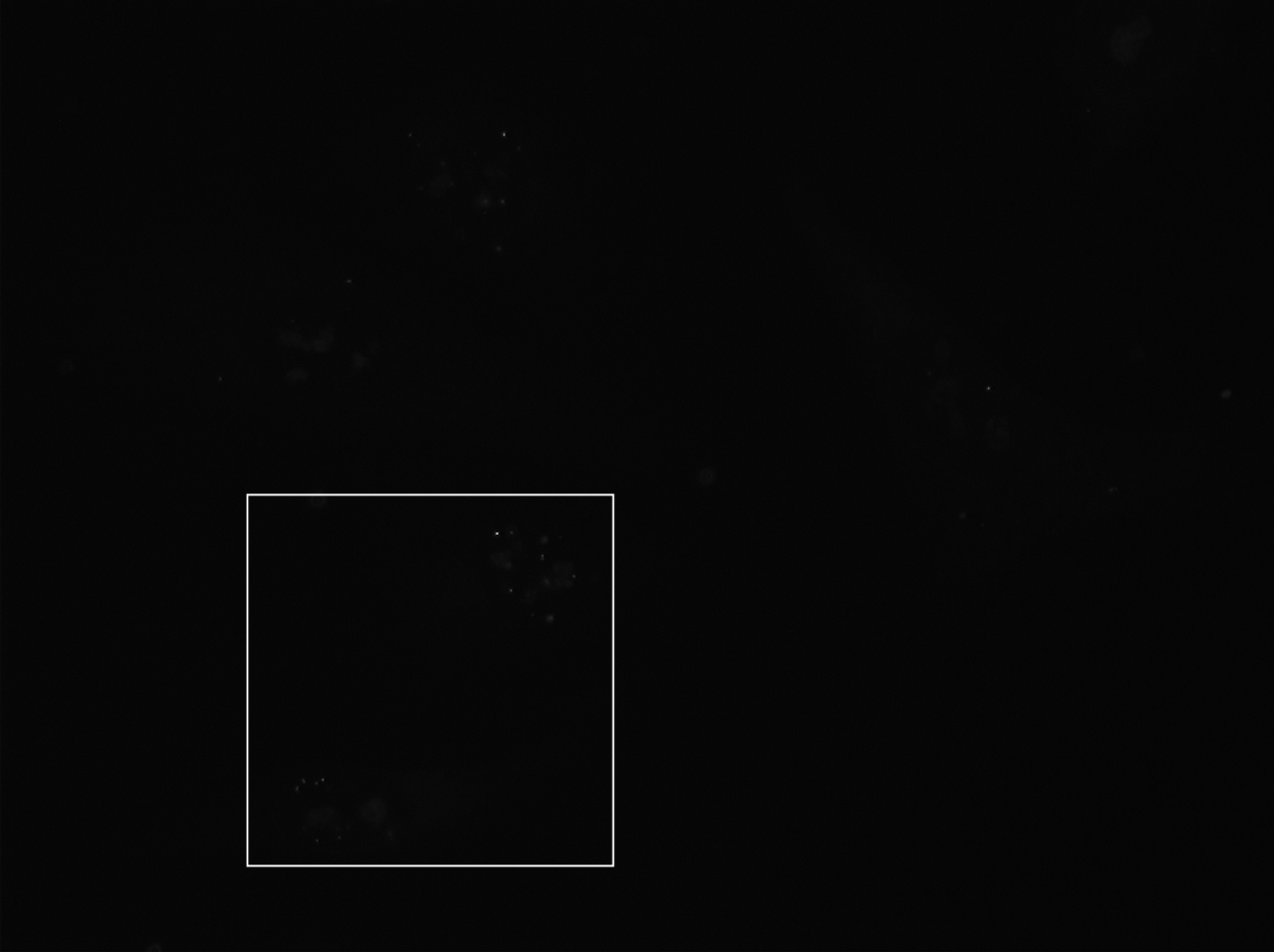

Supplement: Supplementary file 2 — Source data Fig. 1 [file 44318_2025_602_MOESM2_ESM.zip › Fig 1/C/siRNF20 CPT SIRF Screenshot.png]

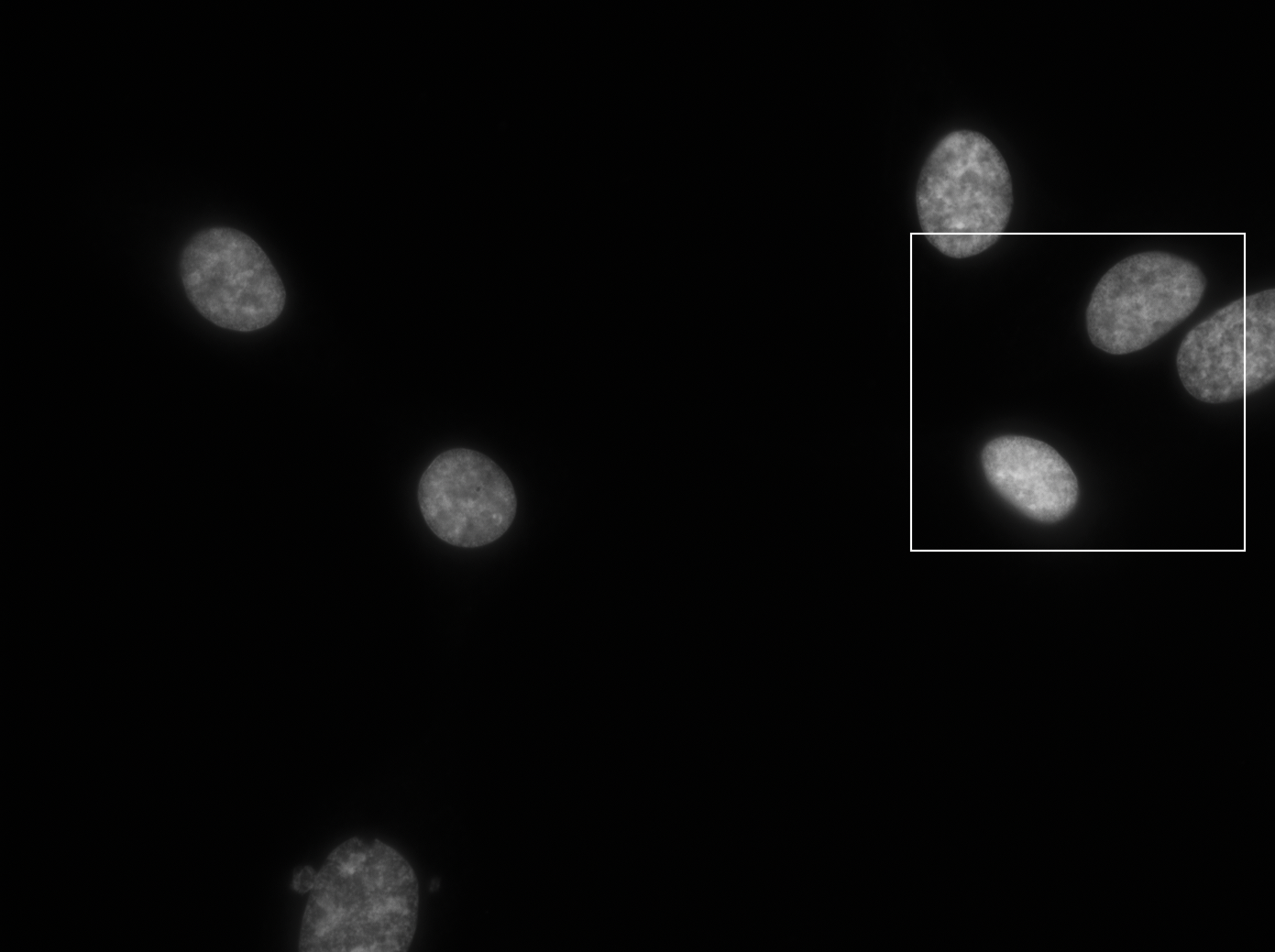

Supplement: Supplementary file 2 — Source data Fig. 1 [file 44318_2025_602_MOESM2_ESM.zip › Fig 1/C/siLuc CPT DAPI Screenshot.png]

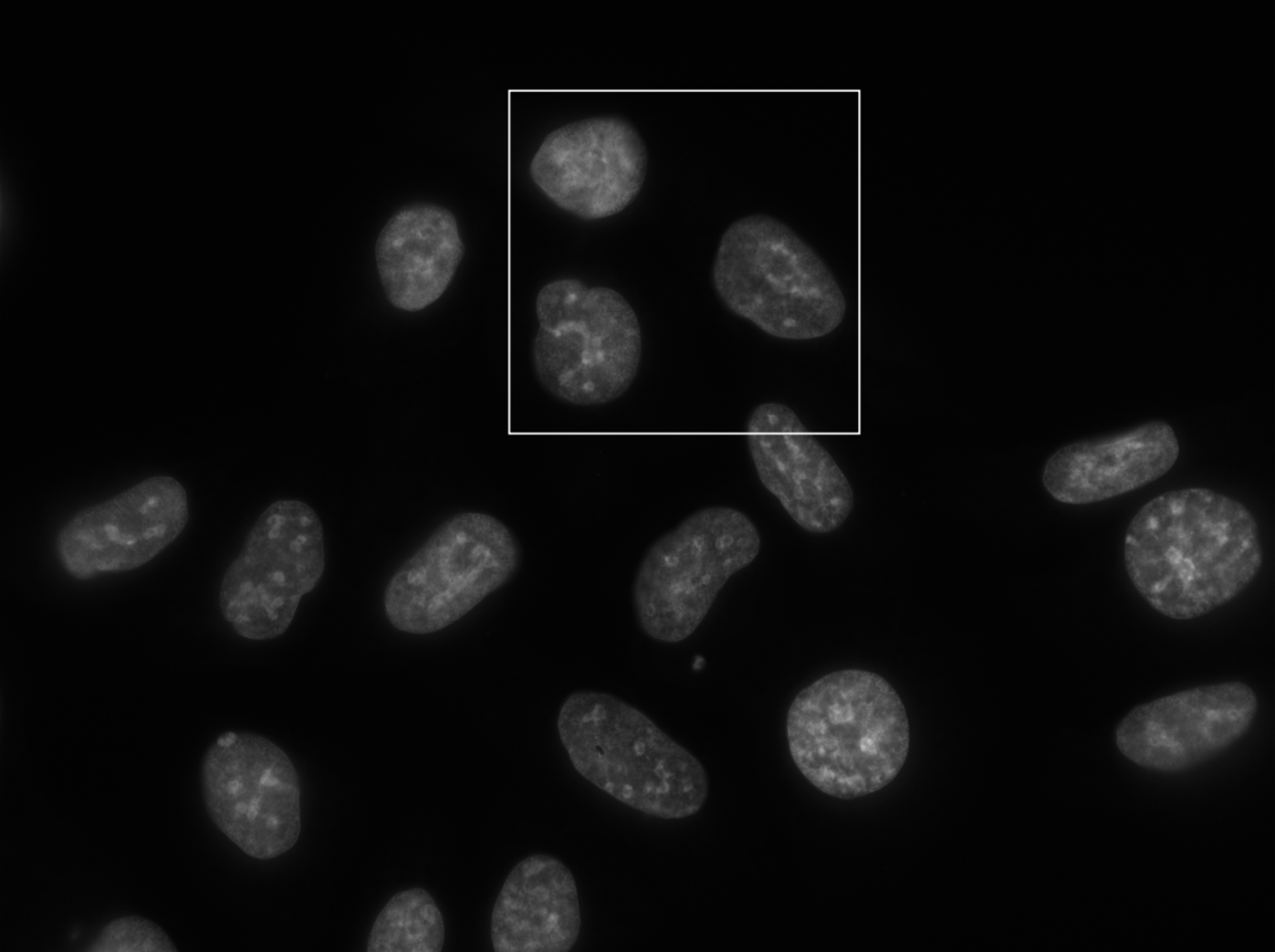

Supplement: Supplementary file 2 — Source data Fig. 1 [file 44318_2025_602_MOESM2_ESM.zip › Fig 1/C/siLuc HU DAPI Screenshot.png]

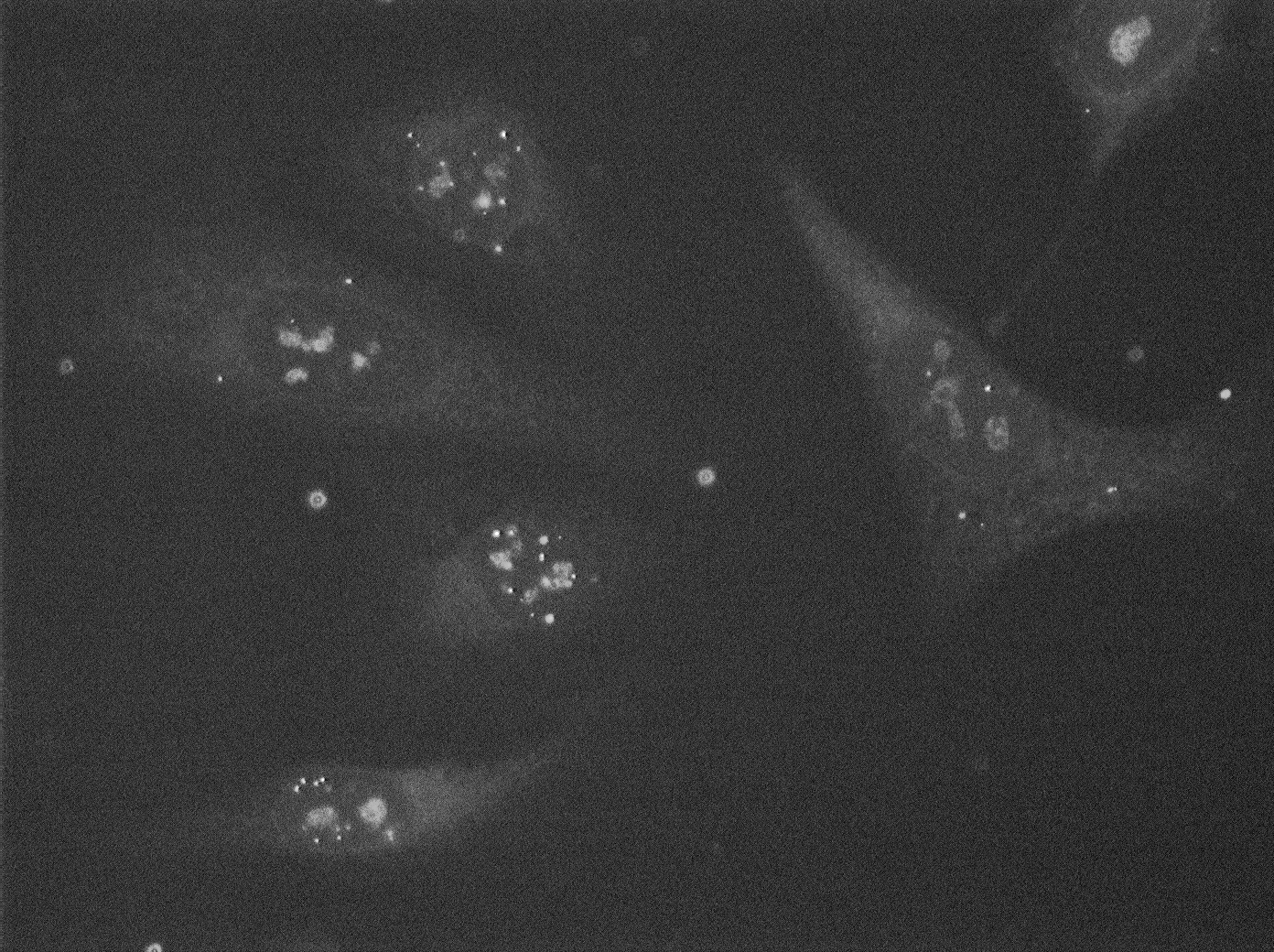

Supplement: Supplementary file 2 — Source data Fig. 1 [file 44318_2025_602_MOESM2_ESM.zip › Fig 1/C/siRNF20 CPT SIRF.tif]

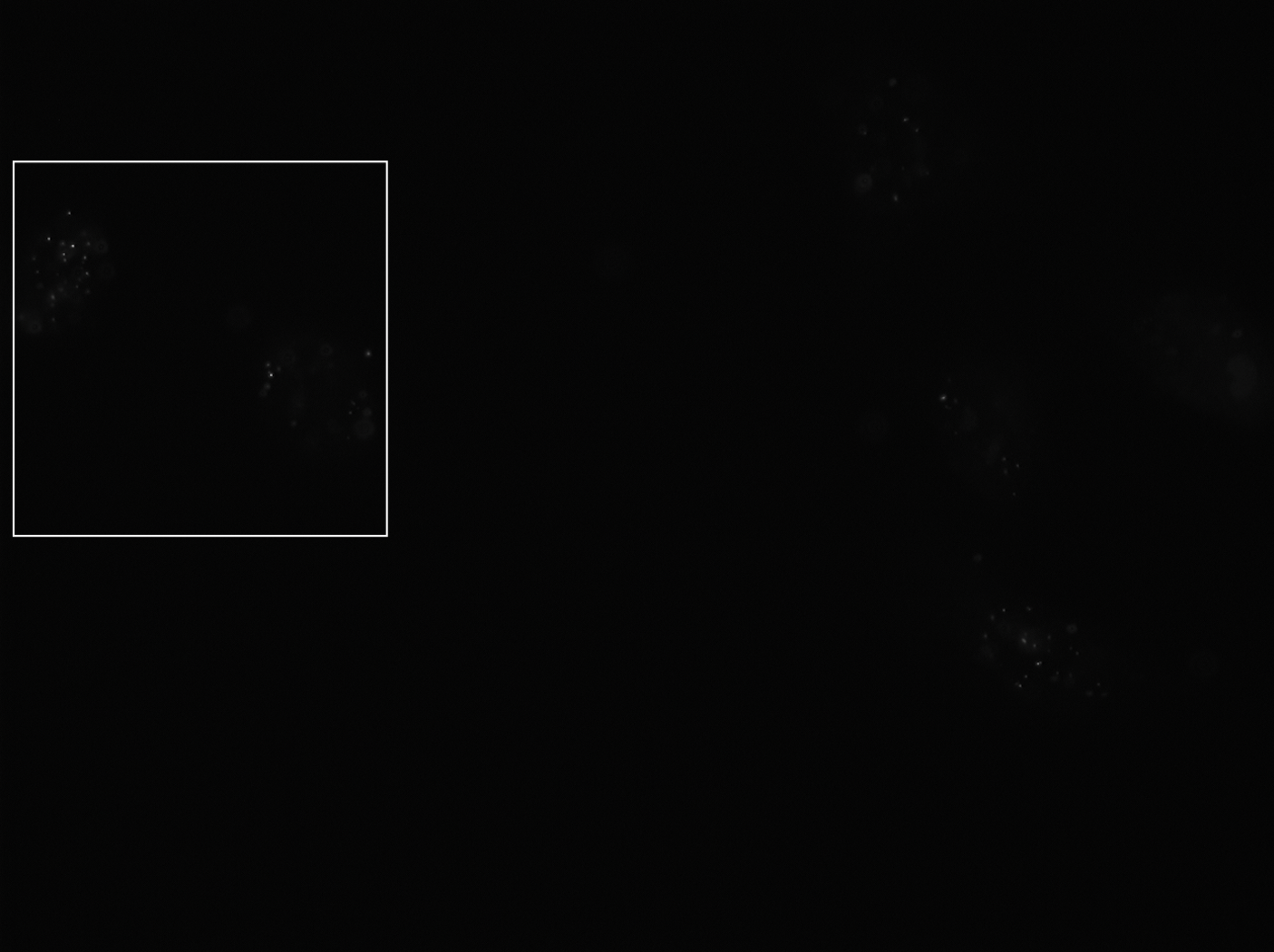

Supplement: Supplementary file 2 — Source data Fig. 1 [file 44318_2025_602_MOESM2_ESM.zip › Fig 1/C/siRNF20 UT SIRF Screenshot.png]

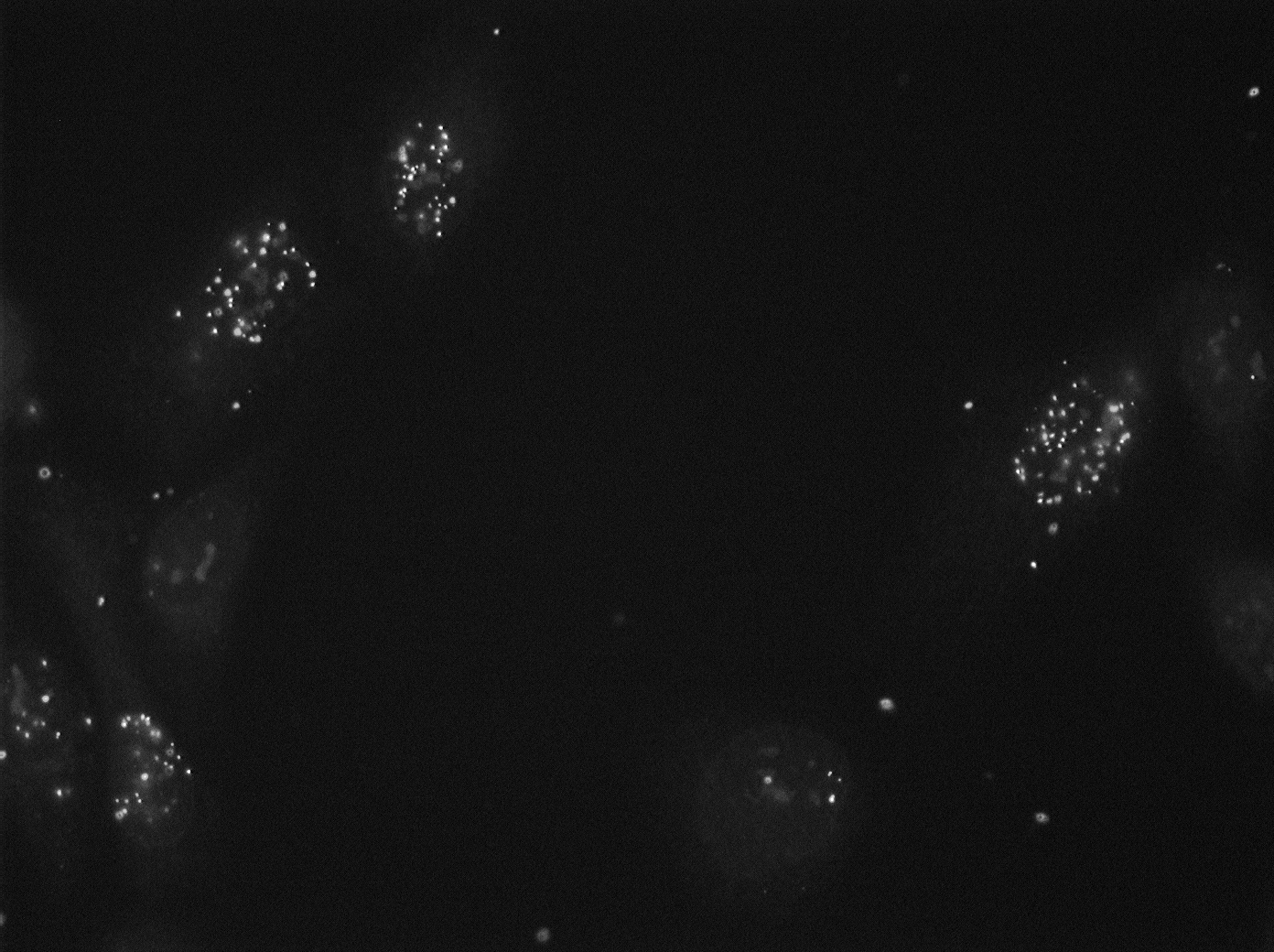

Supplement: Supplementary file 2 — Source data Fig. 1 [file 44318_2025_602_MOESM2_ESM.zip › Fig 1/C/siLuc UT SIRF.tif]

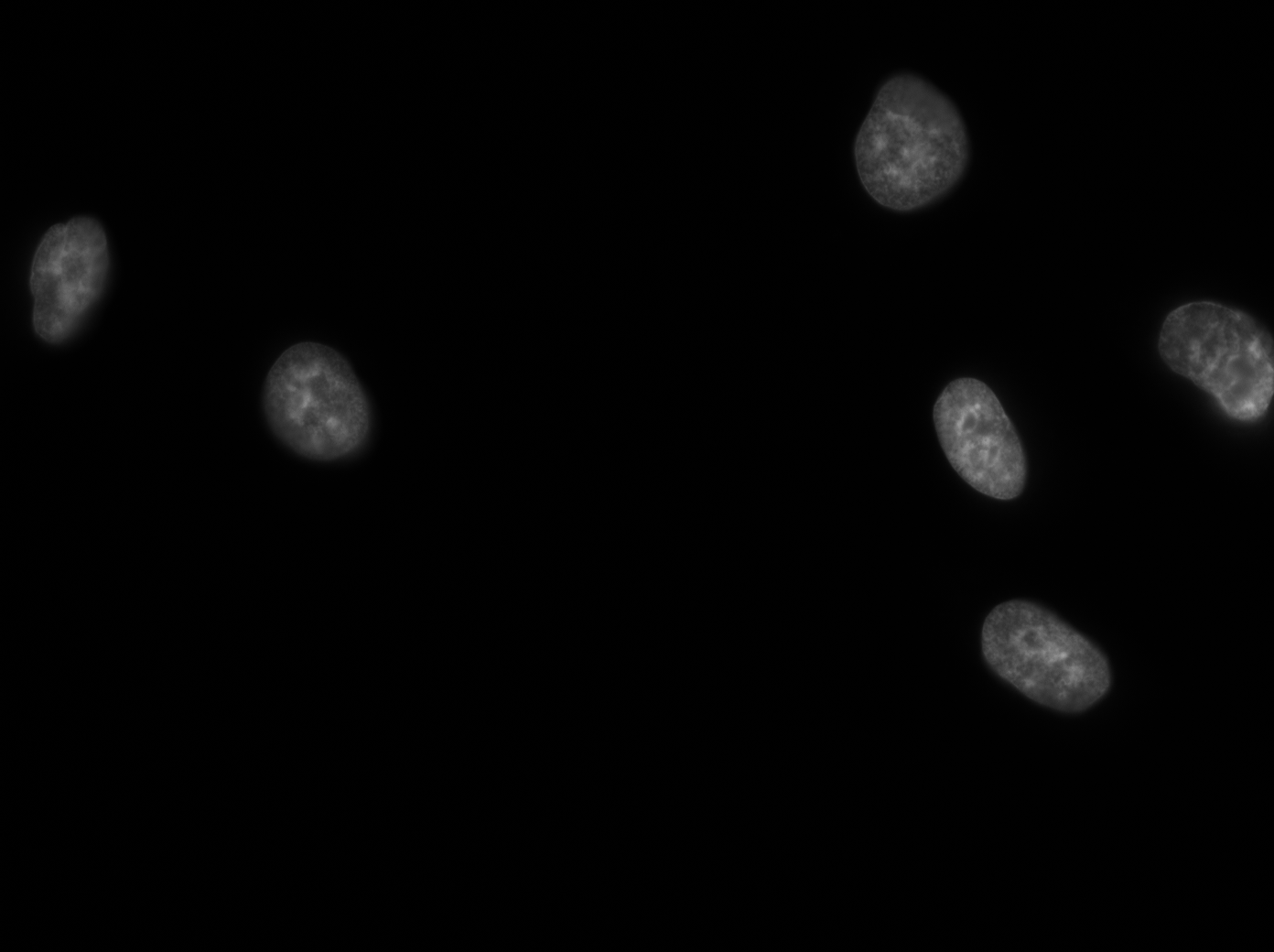

Supplement: Supplementary file 2 — Source data Fig. 1 [file 44318_2025_602_MOESM2_ESM.zip › Fig 1/C/siRNF20 UT DAPI.tif]

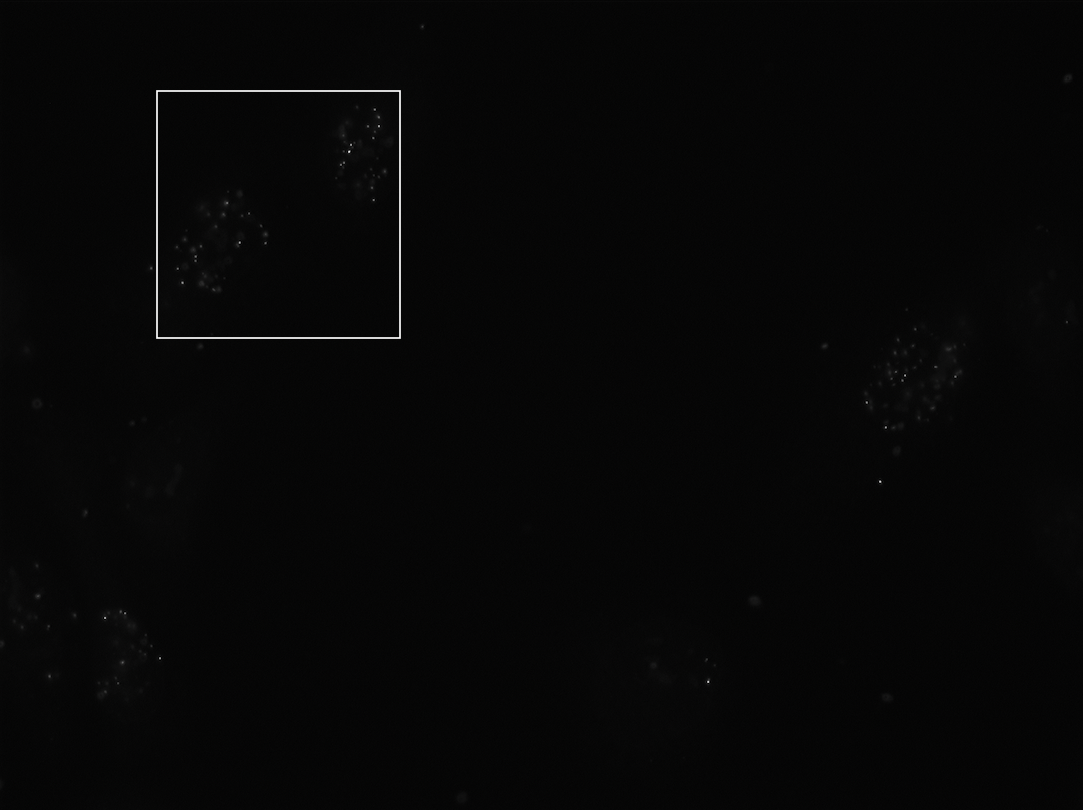

Supplement: Supplementary file 2 — Source data Fig. 1 [file 44318_2025_602_MOESM2_ESM.zip › Fig 1/C/siLuc UT SIRF Screenshot.png]

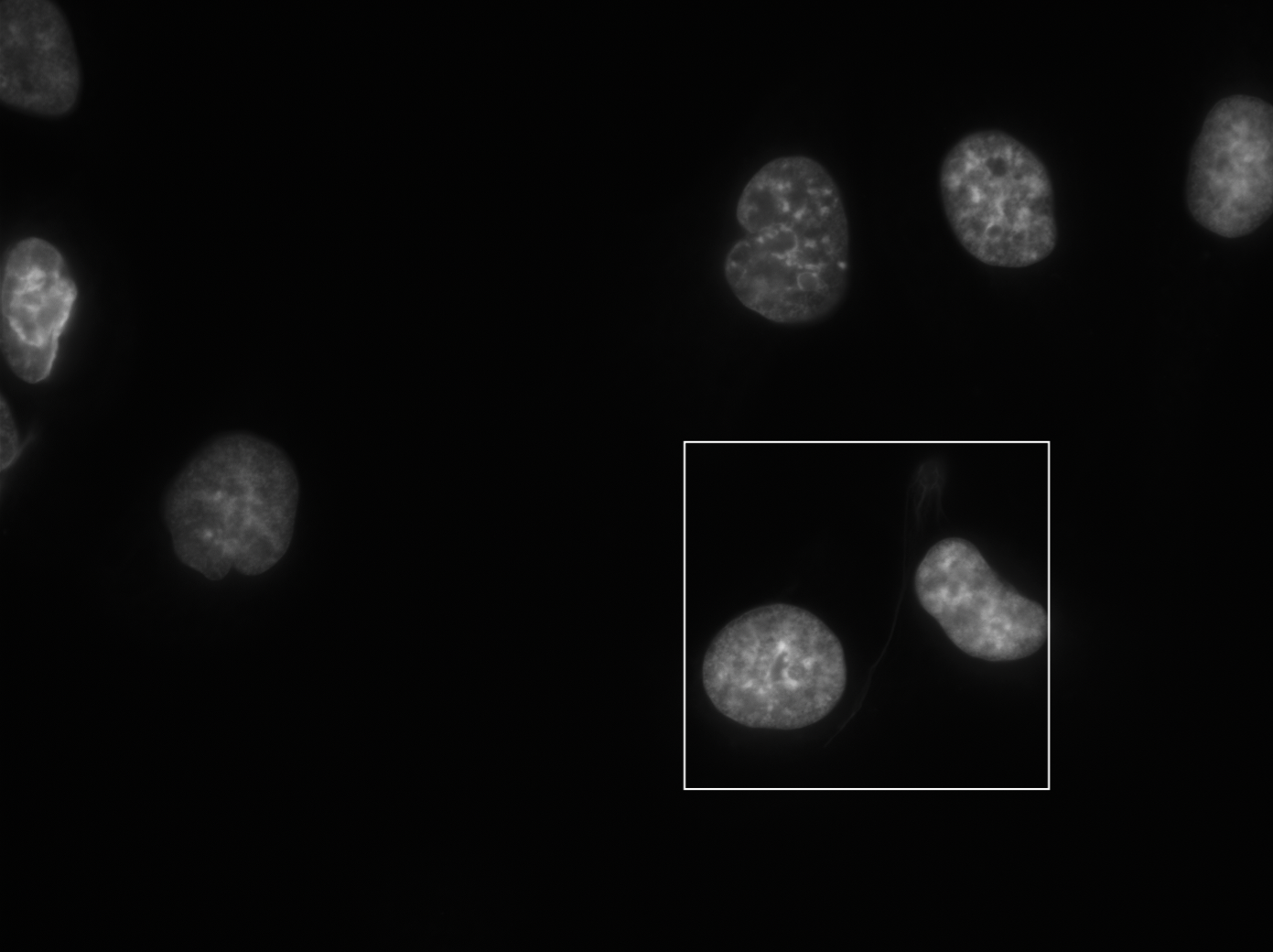

Supplement: Supplementary file 2 — Source data Fig. 1 [file 44318_2025_602_MOESM2_ESM.zip › Fig 1/C/siRNF20 HU DAPI Screenshot.png]

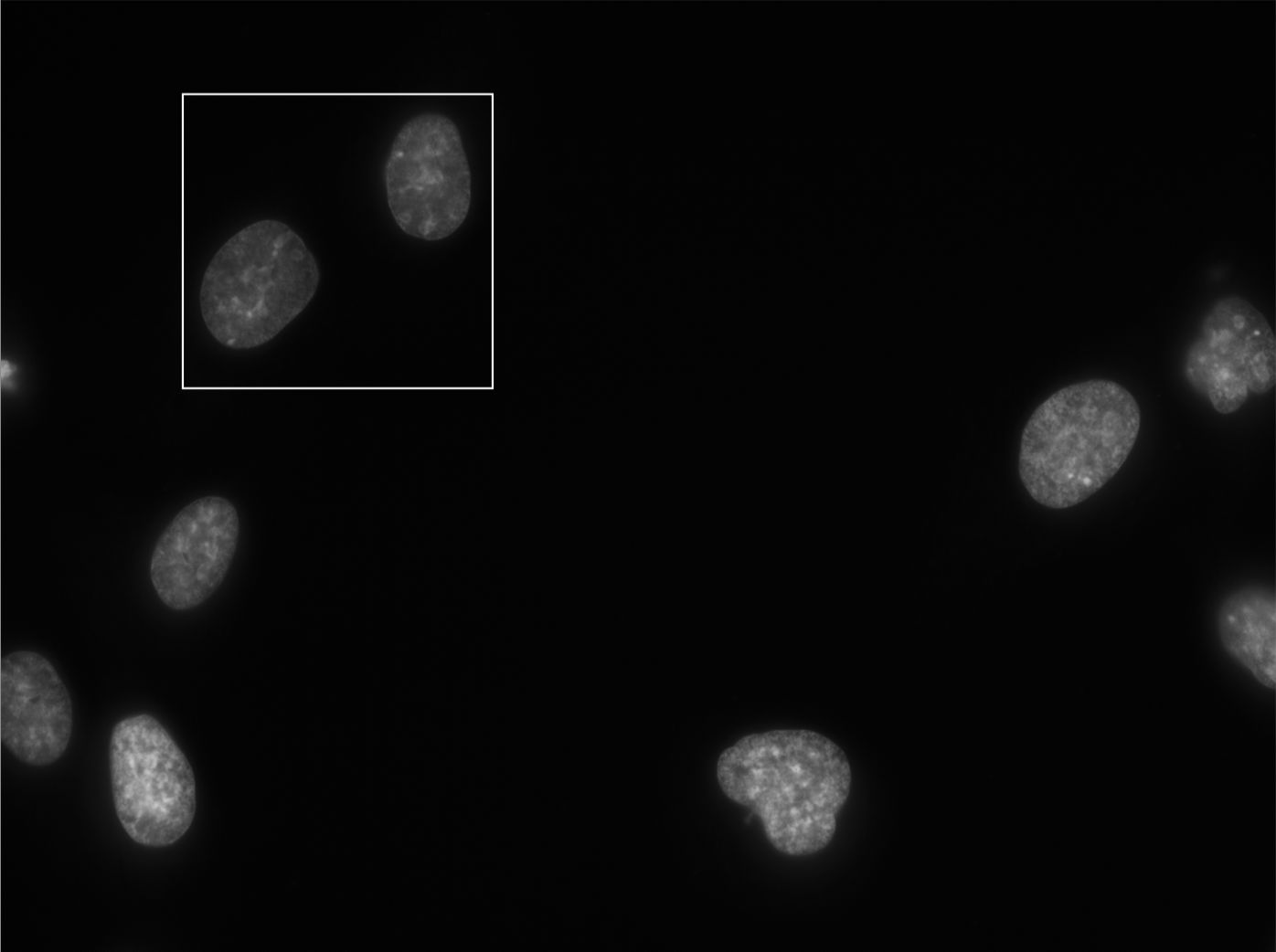

Supplement: Supplementary file 2 — Source data Fig. 1 [file 44318_2025_602_MOESM2_ESM.zip › Fig 1/C/siLuc UT DAPI Screenshot.png]

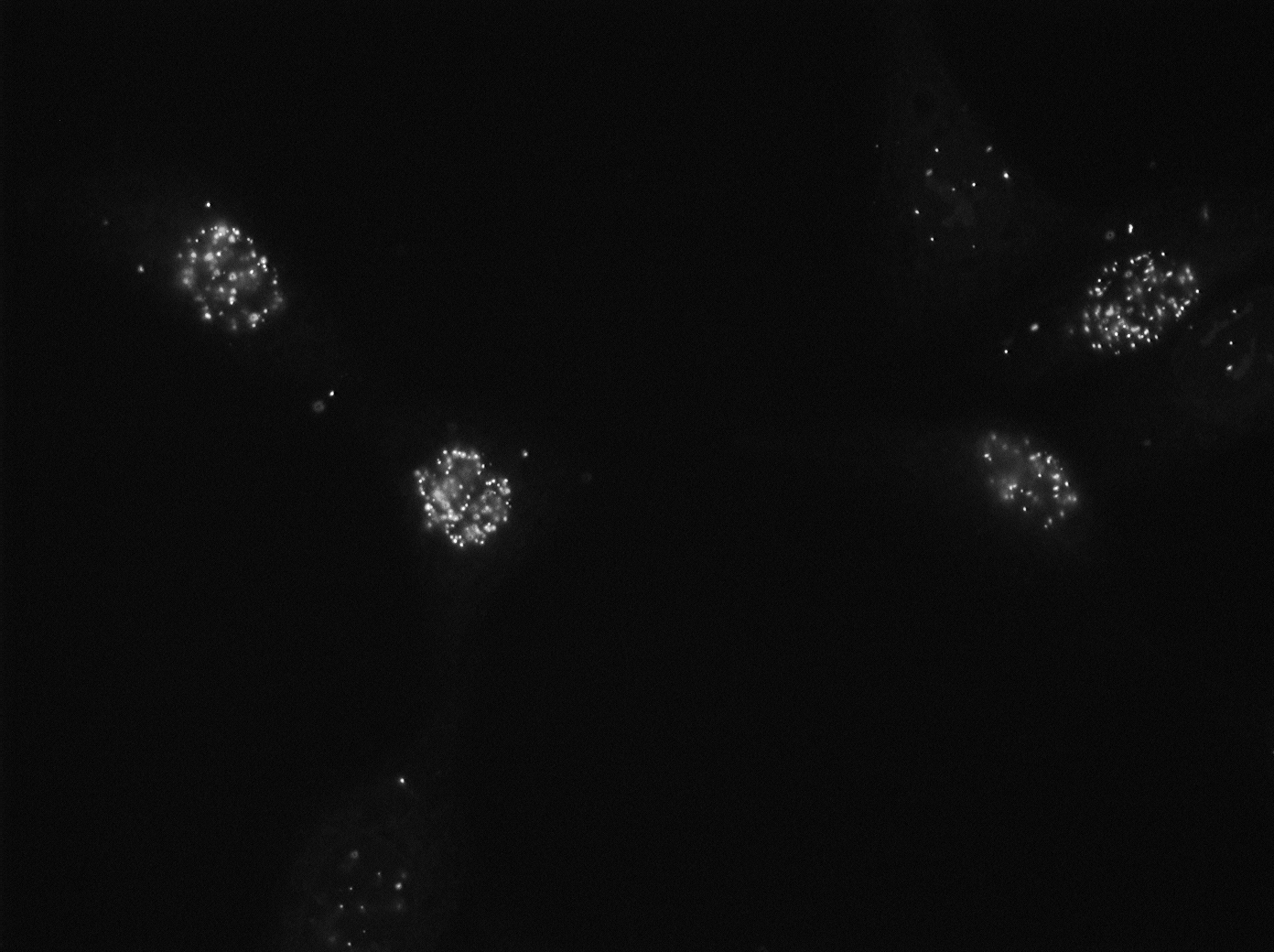

Supplement: Supplementary file 2 — Source data Fig. 1 [file 44318_2025_602_MOESM2_ESM.zip › Fig 1/C/siLuc CPT SIRF.tif]

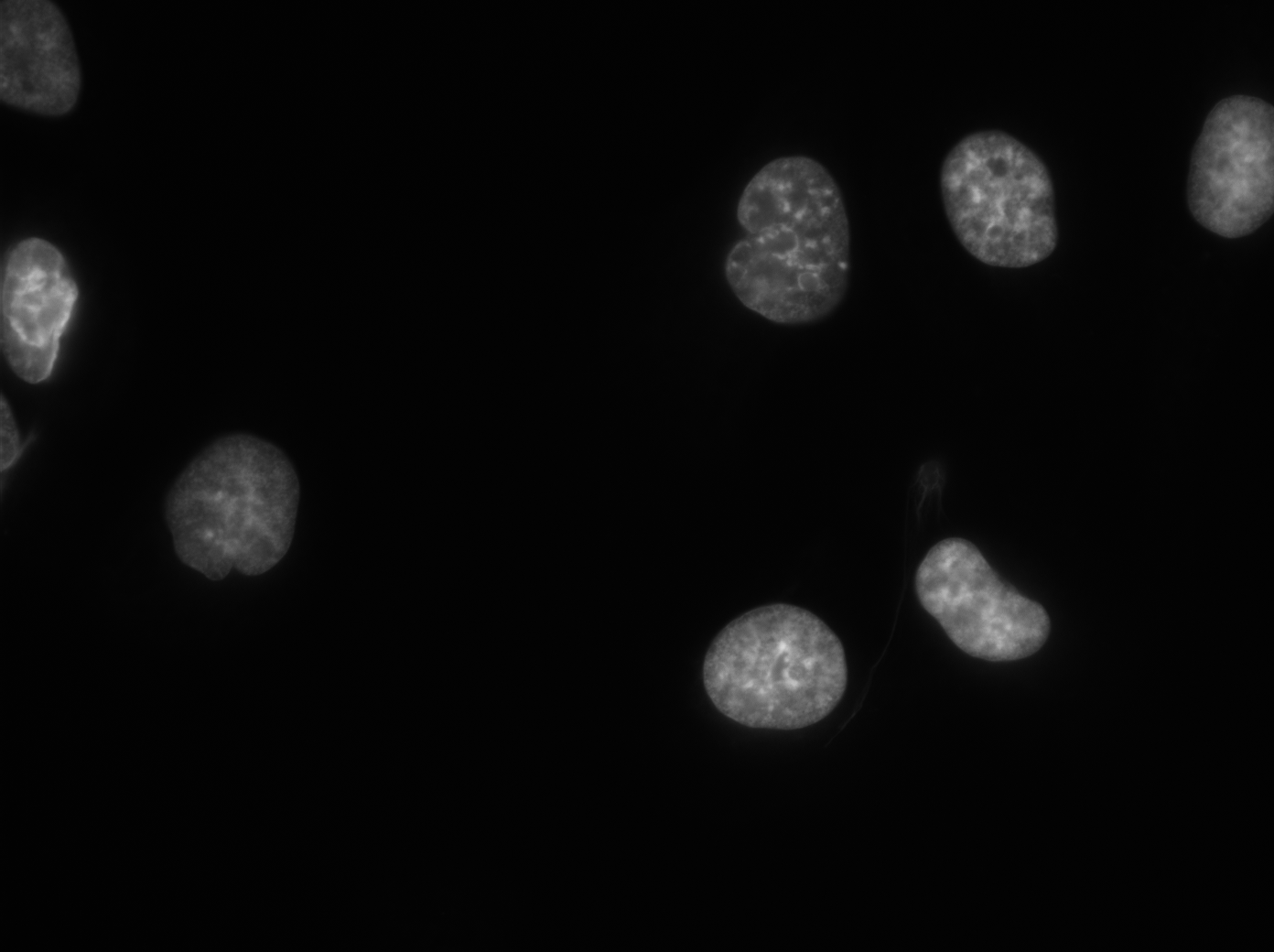

Supplement: Supplementary file 2 — Source data Fig. 1 [file 44318_2025_602_MOESM2_ESM.zip › Fig 1/C/siRNF20 HU DAPI.tif]

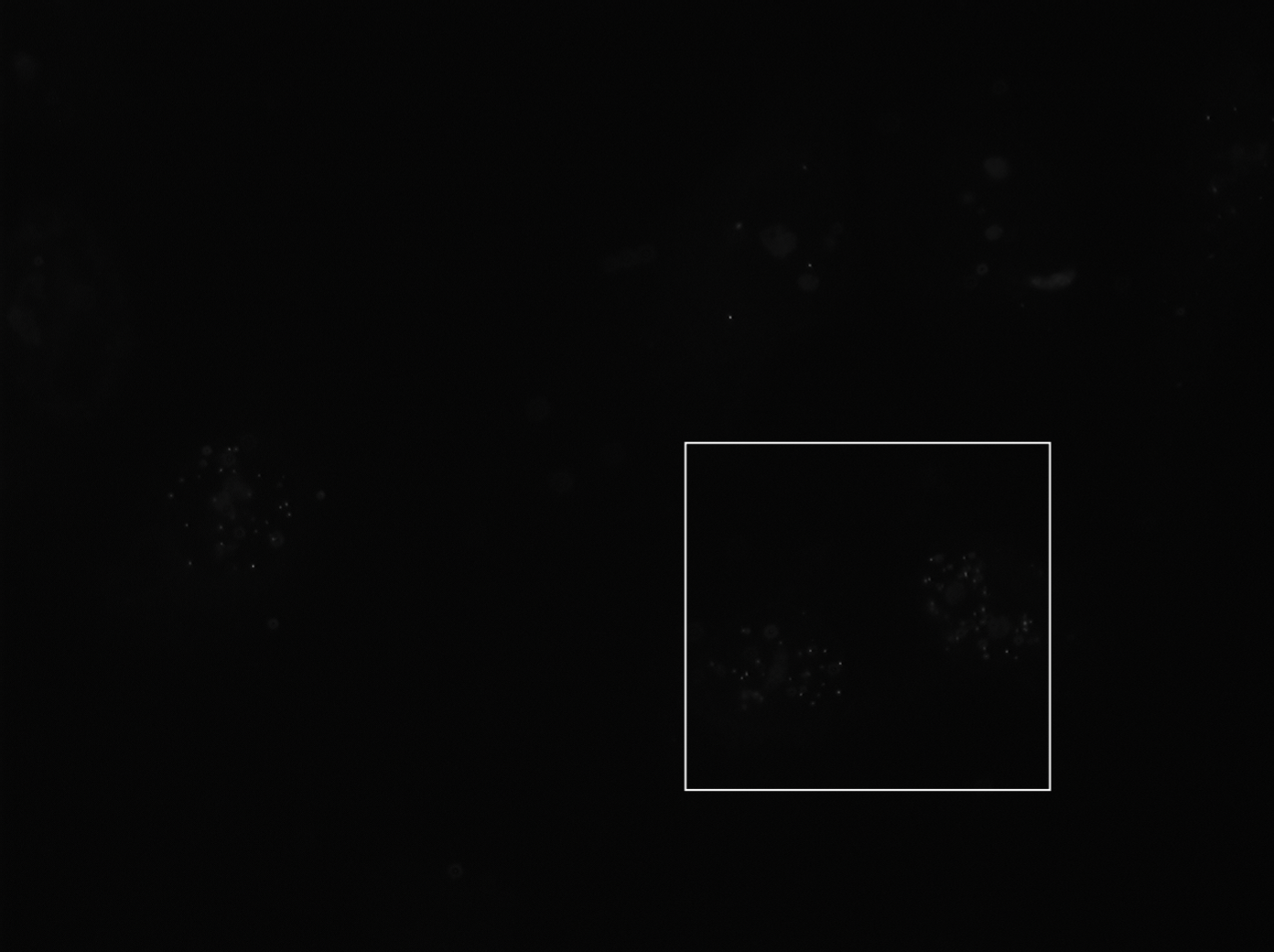

Supplement: Supplementary file 2 — Source data Fig. 1 [file 44318_2025_602_MOESM2_ESM.zip › Fig 1/C/siRNF20 HU SIRF Screenshot.png]

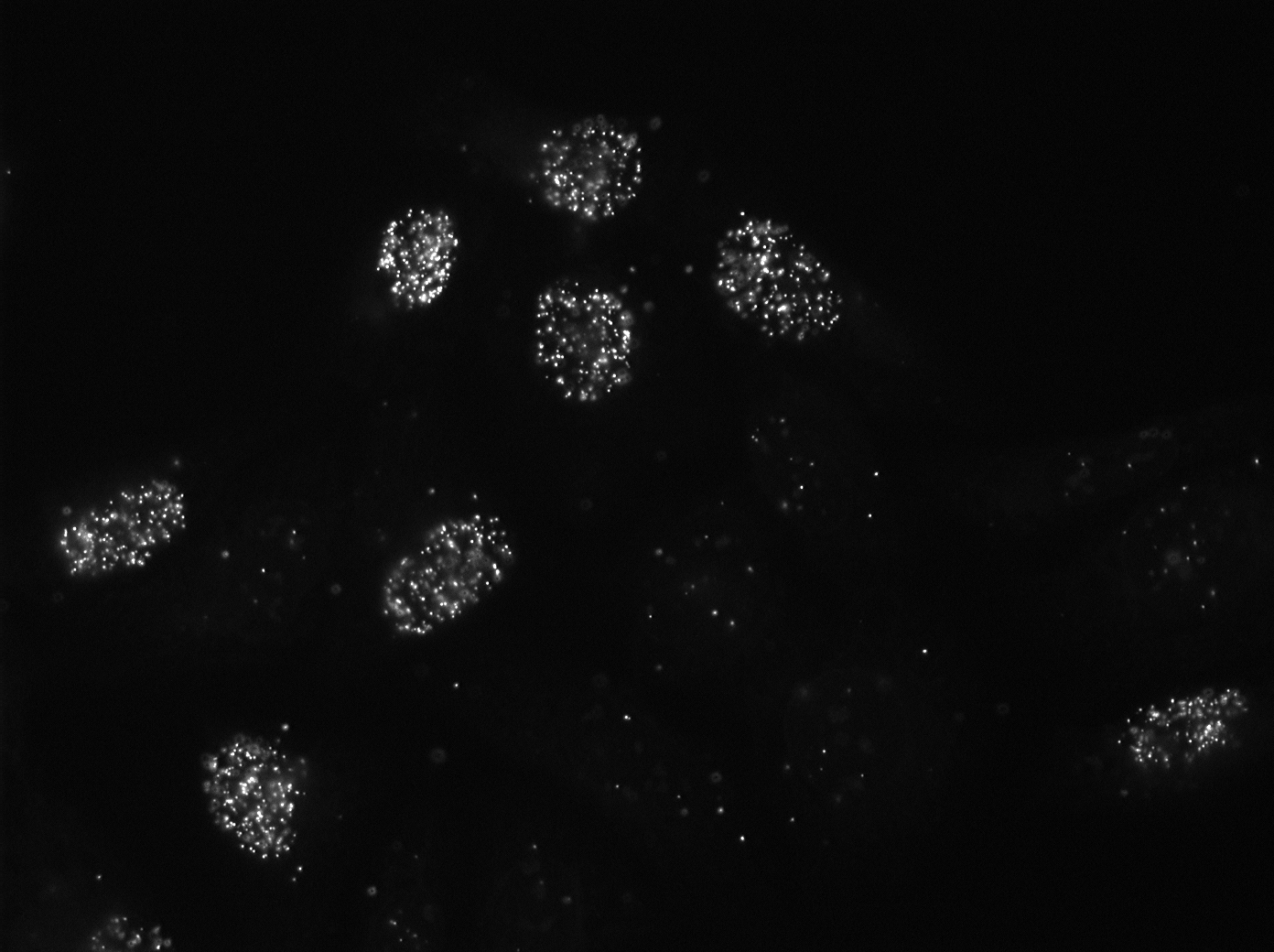

Supplement: Supplementary file 2 — Source data Fig. 1 [file 44318_2025_602_MOESM2_ESM.zip › Fig 1/C/siLuc HU SIRF.tif]

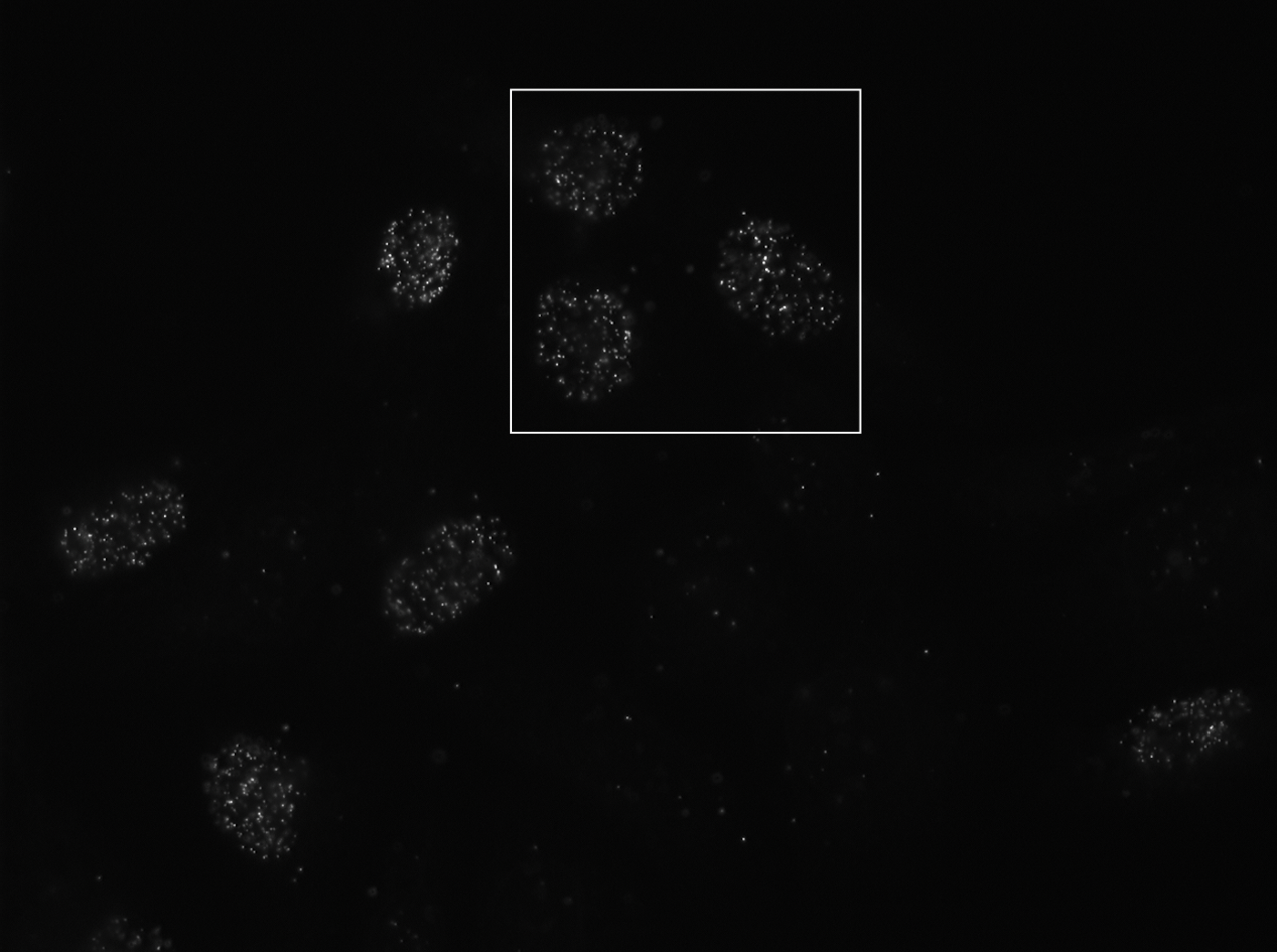

Supplement: Supplementary file 2 — Source data Fig. 1 [file 44318_2025_602_MOESM2_ESM.zip › Fig 1/C/siLuc HU SIRF Screenshot.png]

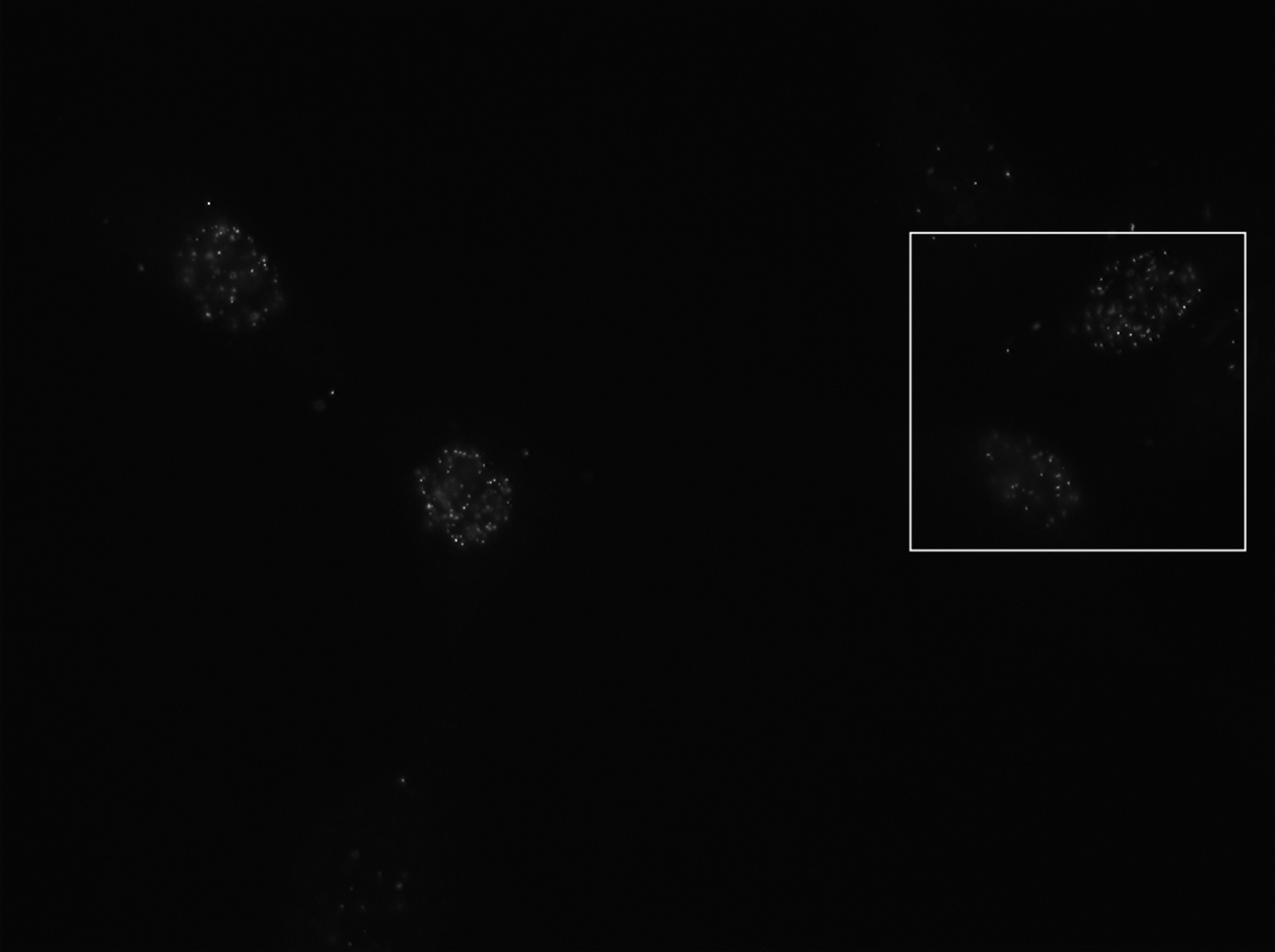

Supplement: Supplementary file 2 — Source data Fig. 1 [file 44318_2025_602_MOESM2_ESM.zip › Fig 1/C/siLuc CPT SIRF Screenshot.png]

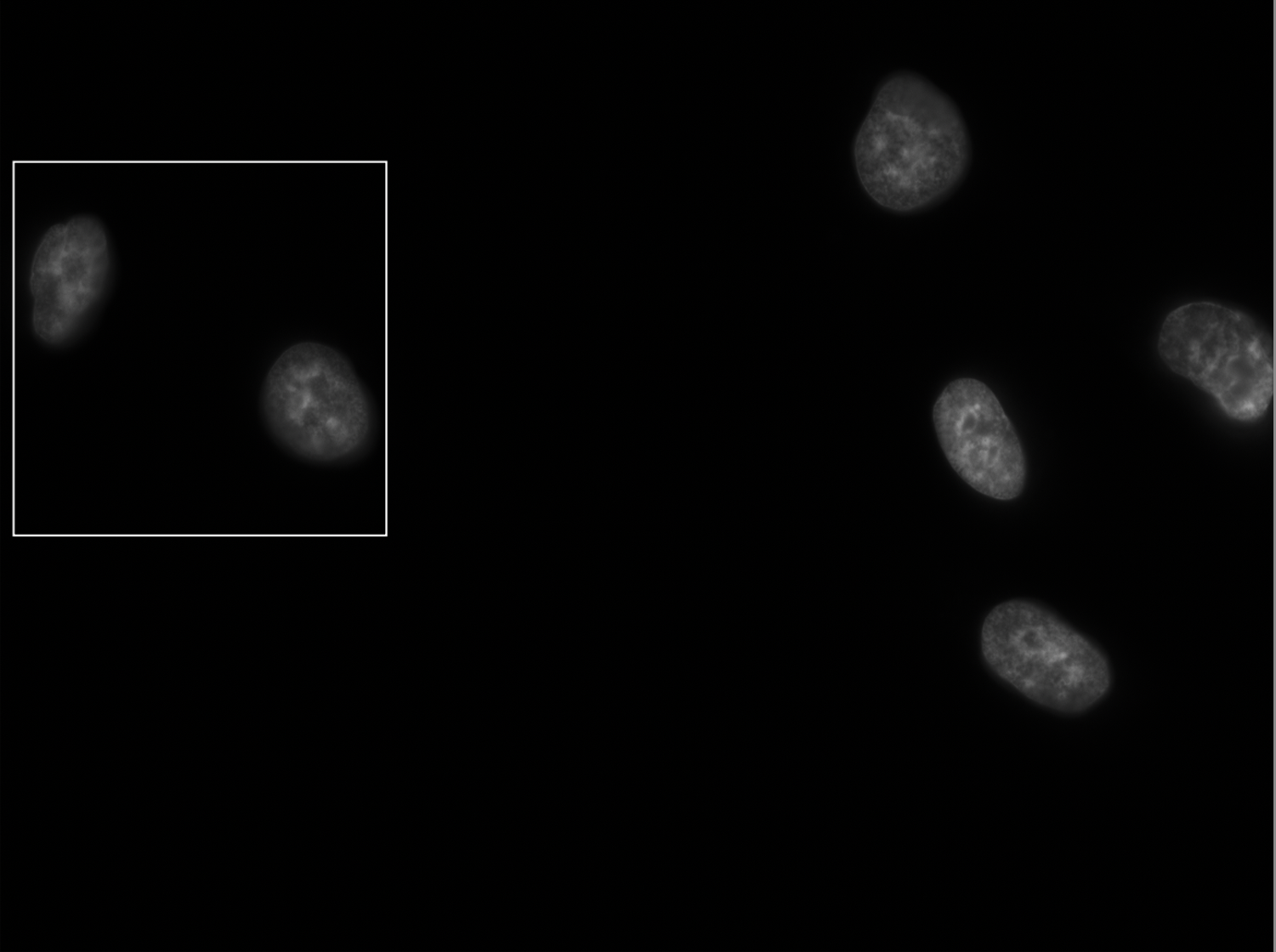

Supplement: Supplementary file 2 — Source data Fig. 1 [file 44318_2025_602_MOESM2_ESM.zip › Fig 1/C/siRNF20 UT DAPI Screenshot.png]

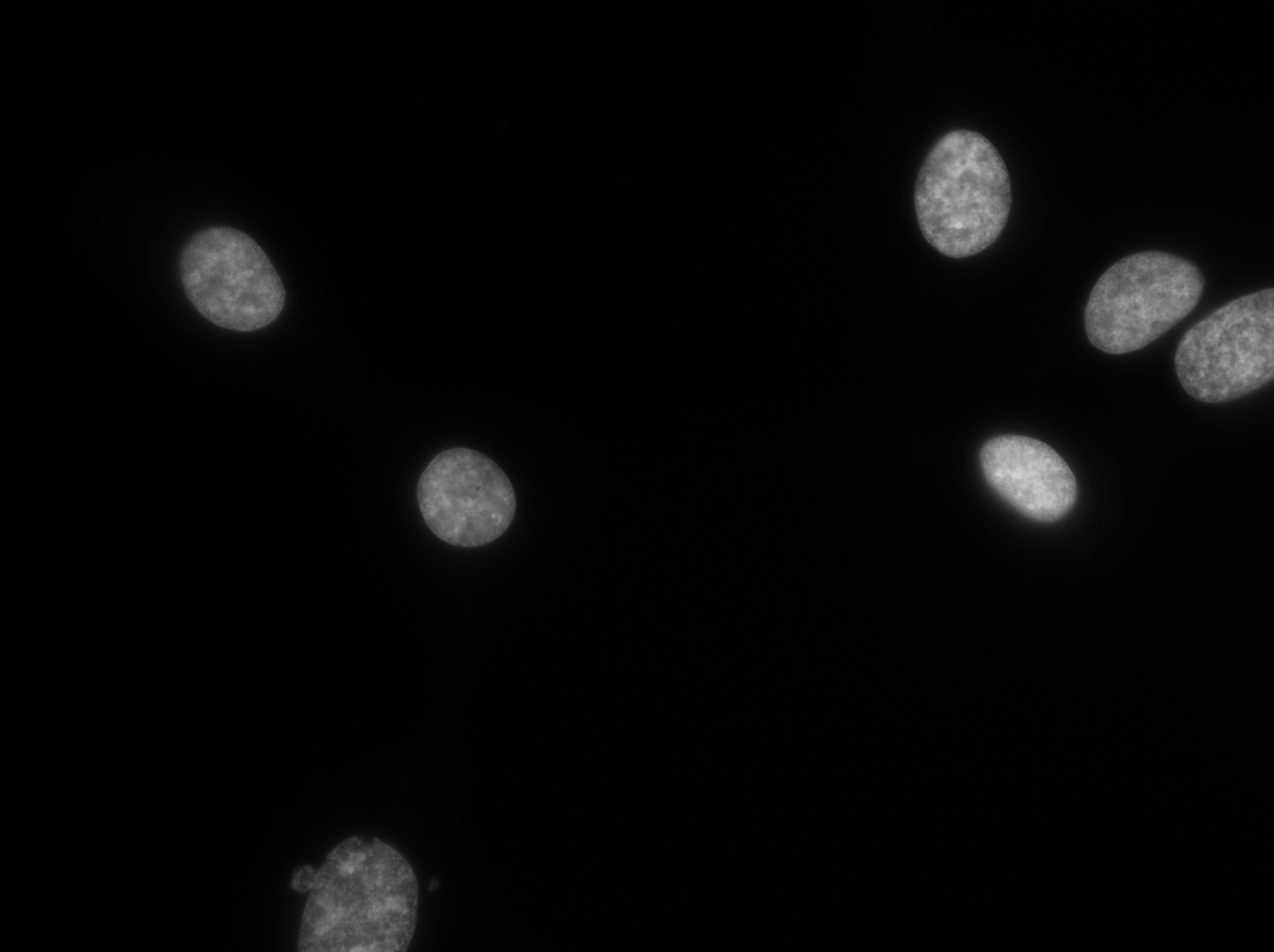

Supplement: Supplementary file 2 — Source data Fig. 1 [file 44318_2025_602_MOESM2_ESM.zip › Fig 1/C/siLuc CPT DAPI.tif]

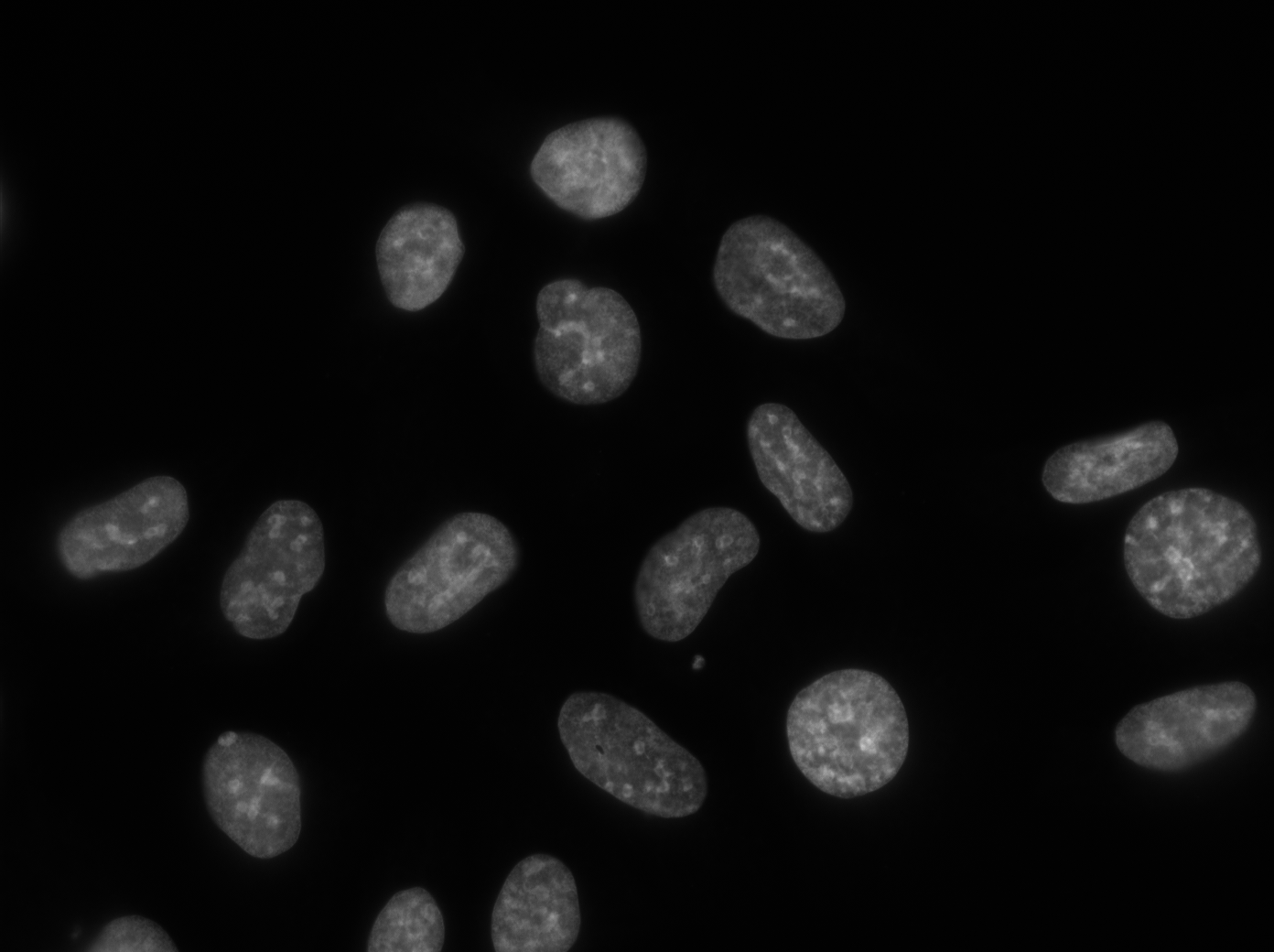

Supplement: Supplementary file 2 — Source data Fig. 1 [file 44318_2025_602_MOESM2_ESM.zip › Fig 1/C/siLuc HU DAPI.tif]

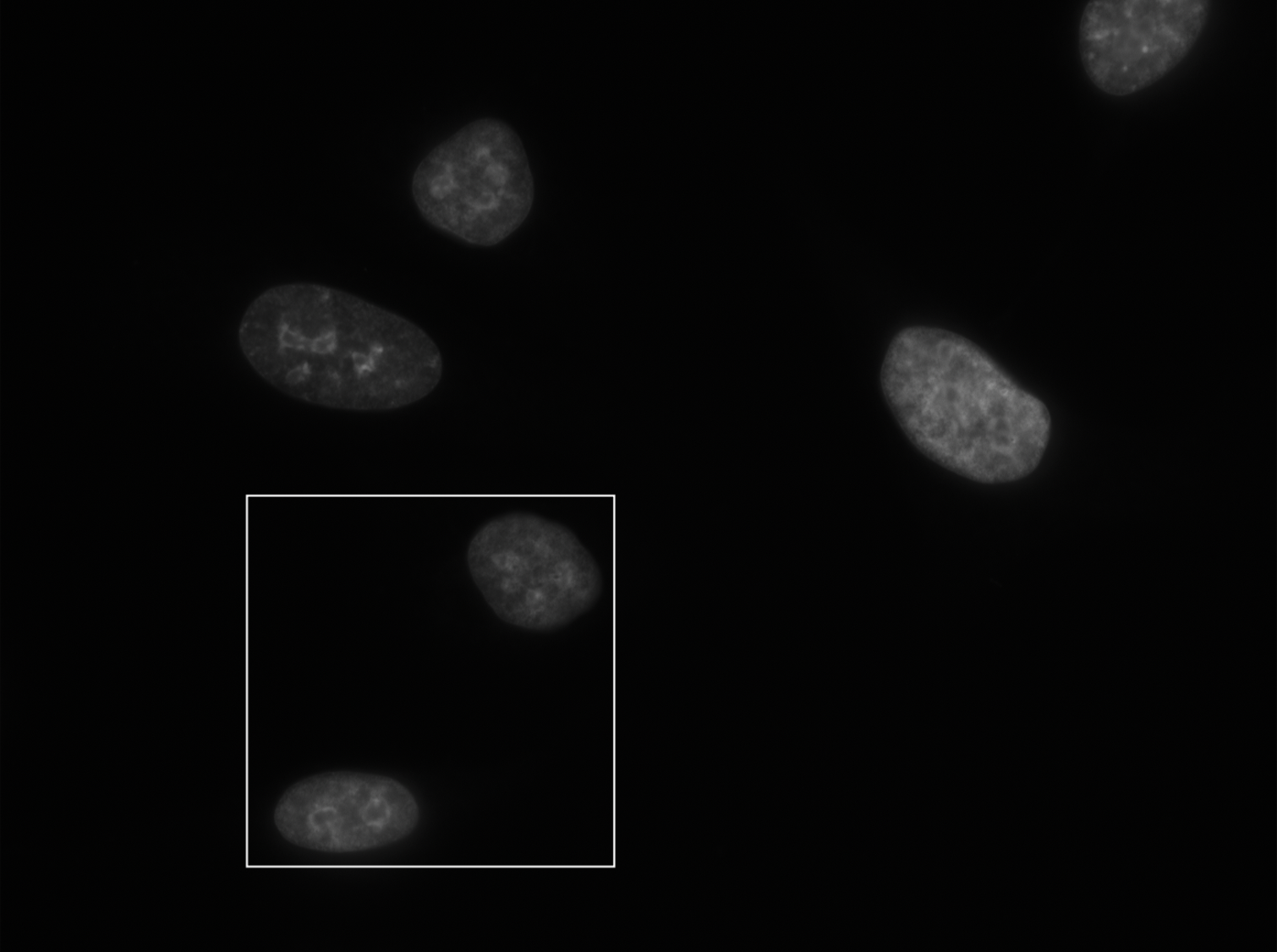

Supplement: Supplementary file 2 — Source data Fig. 1 [file 44318_2025_602_MOESM2_ESM.zip › Fig 1/C/siRNF20 CPT DAPI Screenshot.png]

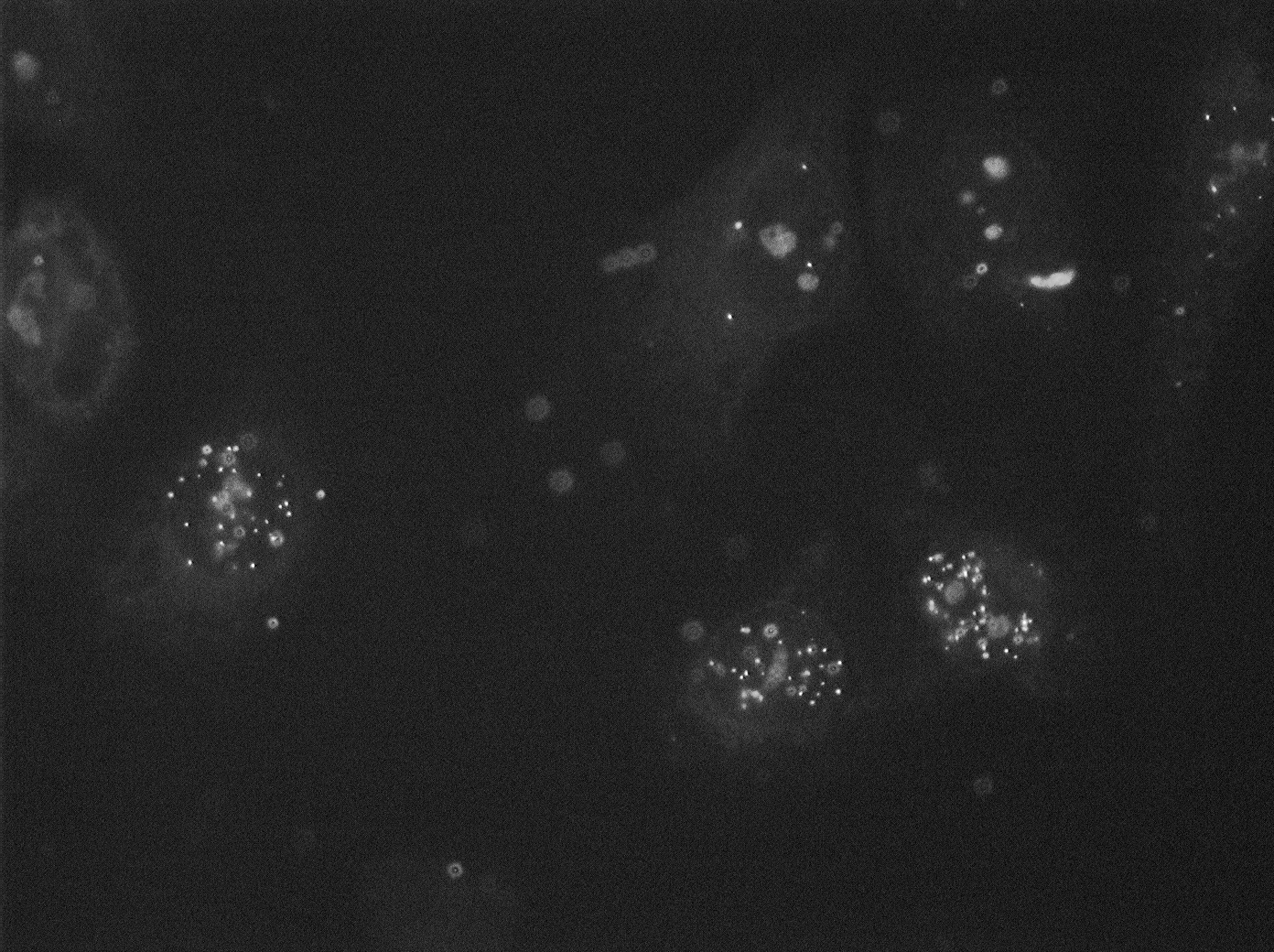

Supplement: Supplementary file 2 — Source data Fig. 1 [file 44318_2025_602_MOESM2_ESM.zip › Fig 1/C/siRNF20 HU SIRF.tif]

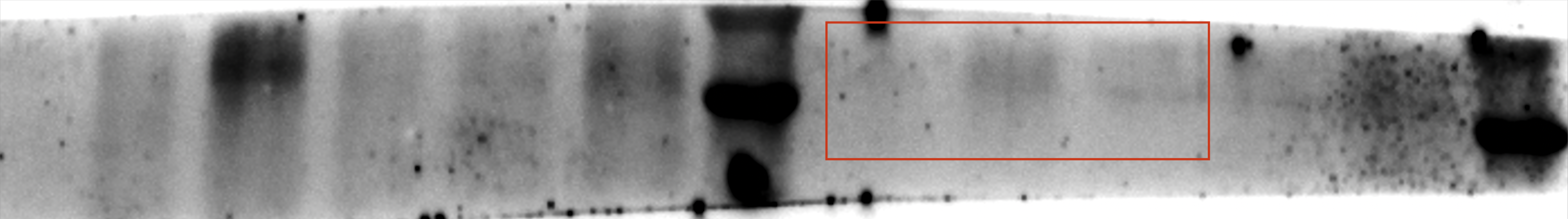

Supplement: Supplementary file 2 — Source data Fig. 1 [file 44318_2025_602_MOESM2_ESM.zip › Fig 1/E/iPOND WB pCHK1 Screenshot.tiff]

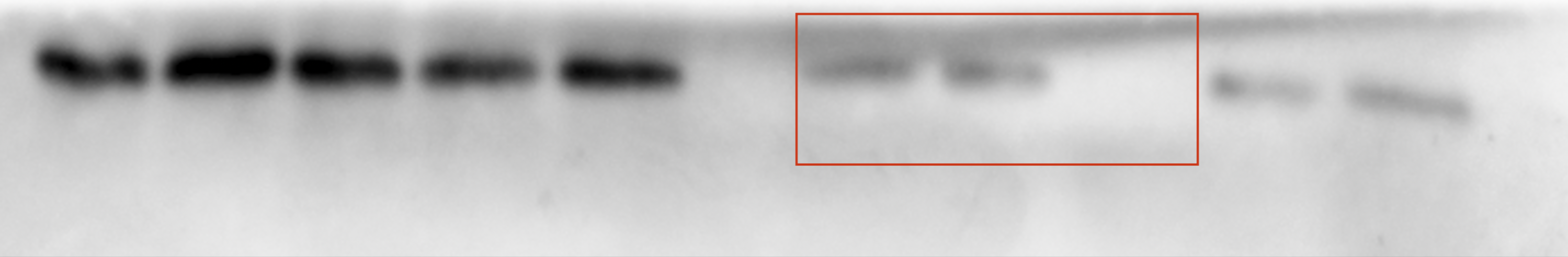

Supplement: Supplementary file 2 — Source data Fig. 1 [file 44318_2025_602_MOESM2_ESM.zip › Fig 1/E/iPOND WB H3 Screenshot.png]

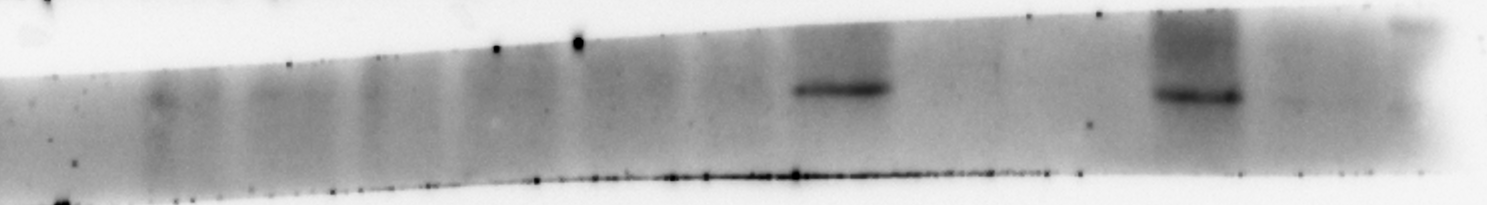

Supplement: Supplementary file 2 — Source data Fig. 1 [file 44318_2025_602_MOESM2_ESM.zip › Fig 1/E/iPOND WB PCNA.tiff]

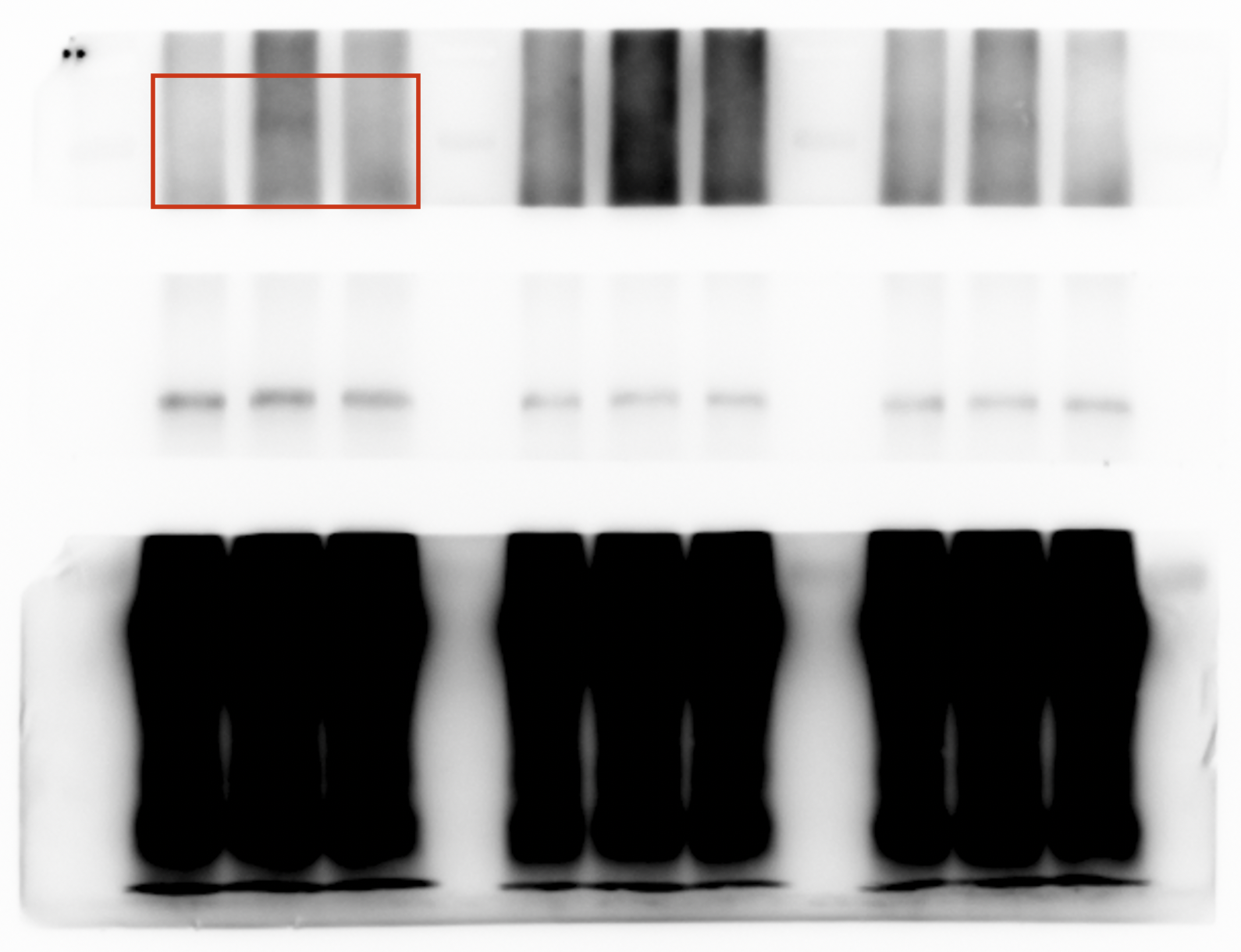

Supplement: Supplementary file 2 — Source data Fig. 1 [file 44318_2025_602_MOESM2_ESM.zip › Fig 1/E/INPUT WB pCHK1 Screenshot.png]

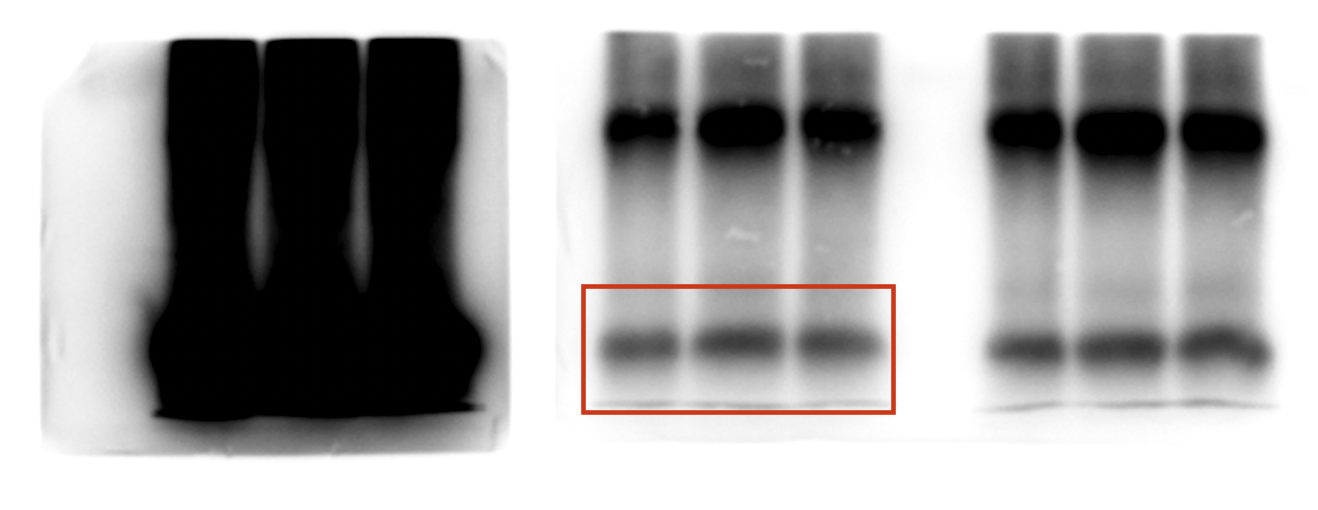

Supplement: Supplementary file 2 — Source data Fig. 1 [file 44318_2025_602_MOESM2_ESM.zip › Fig 1/E/INPUT WB H2B Screenshot.png]

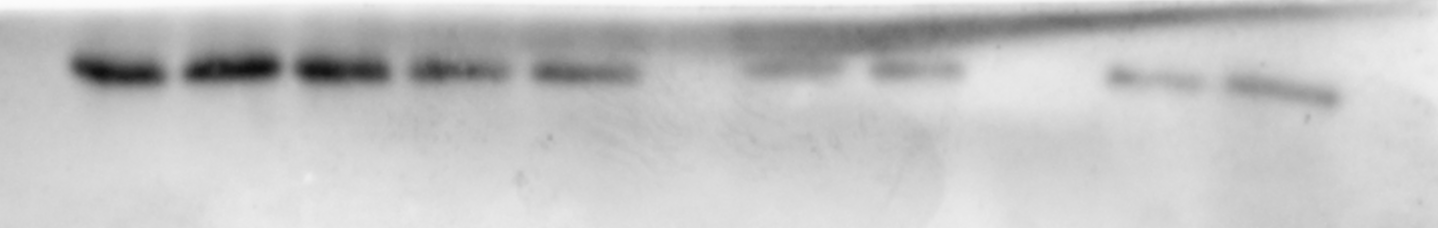

Supplement: Supplementary file 2 — Source data Fig. 1 [file 44318_2025_602_MOESM2_ESM.zip › Fig 1/E/iPOND WB H2B.tif]

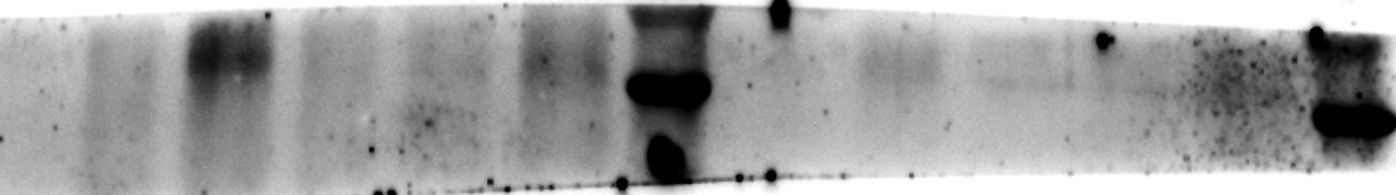

Supplement: Supplementary file 2 — Source data Fig. 1 [file 44318_2025_602_MOESM2_ESM.zip › Fig 1/E/iPOND WB pCHK1.tif]

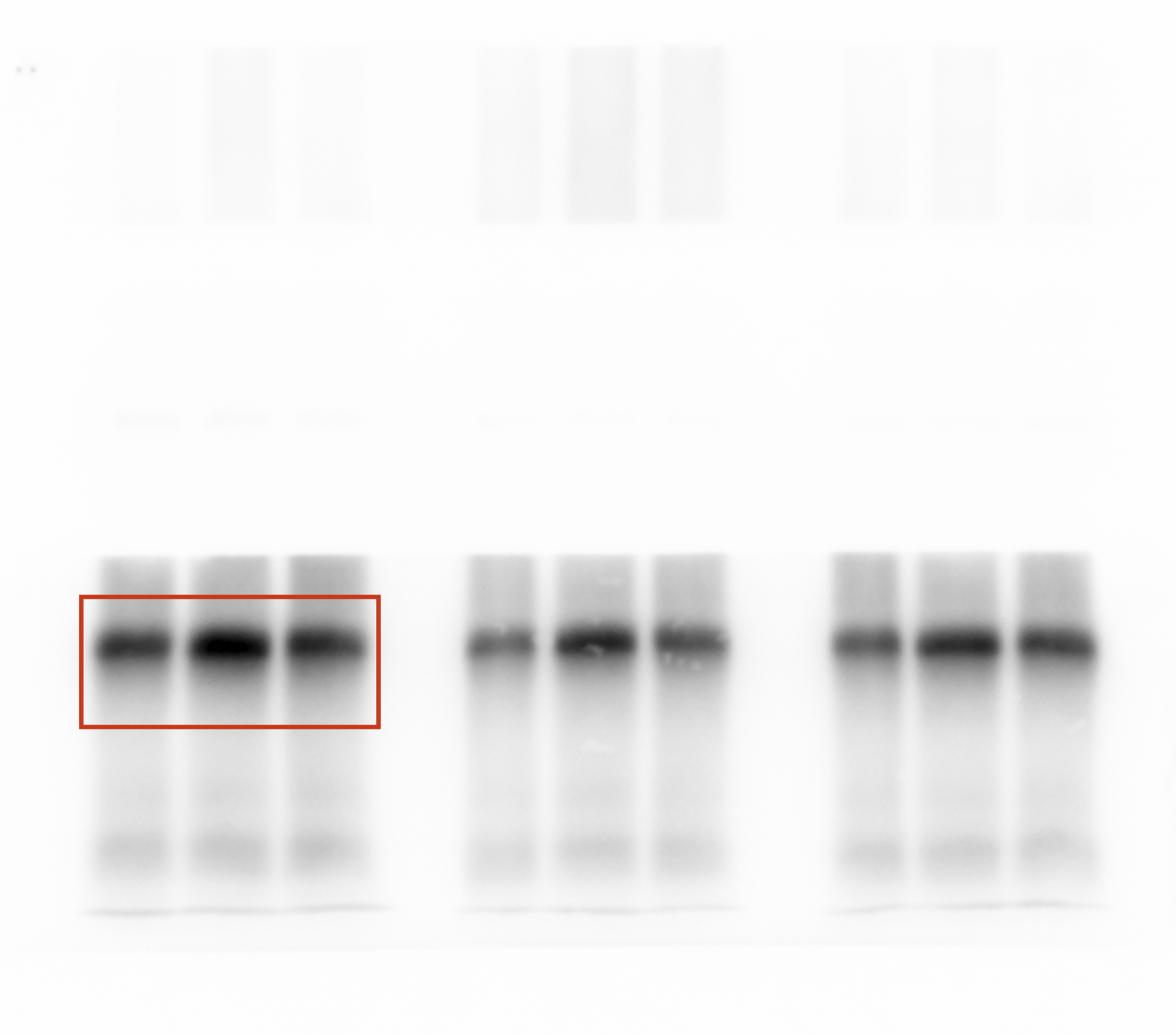

Supplement: Supplementary file 2 — Source data Fig. 1 [file 44318_2025_602_MOESM2_ESM.zip › Fig 1/E/INPUT WB H2Bub Screenshot.png]

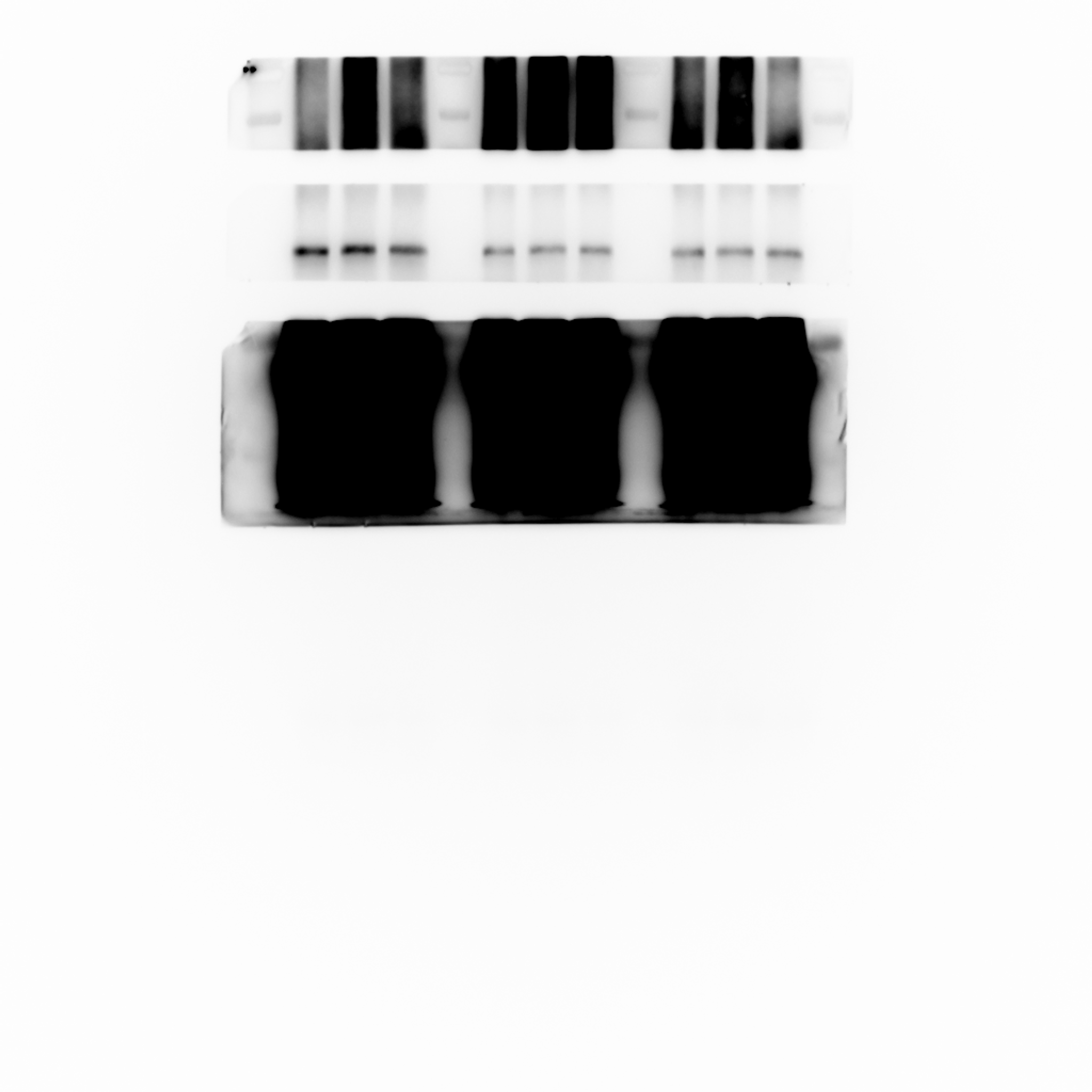

Supplement: Supplementary file 2 — Source data Fig. 1 [file 44318_2025_602_MOESM2_ESM.zip › Fig 1/E/INPUT WB PCNA.Tif]

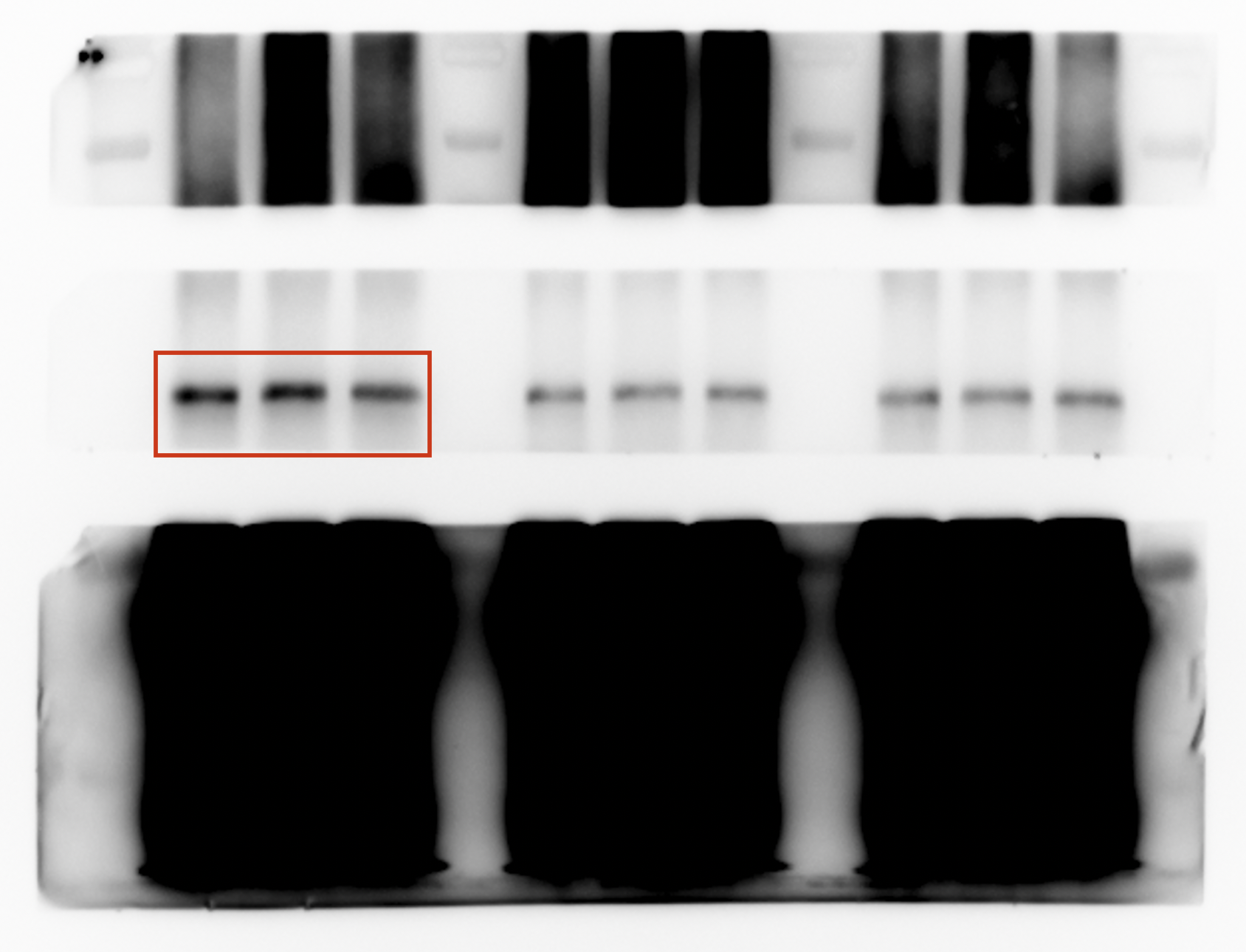

Supplement: Supplementary file 2 — Source data Fig. 1 [file 44318_2025_602_MOESM2_ESM.zip › Fig 1/E/INPUT WB PCNA Screenshot.png]

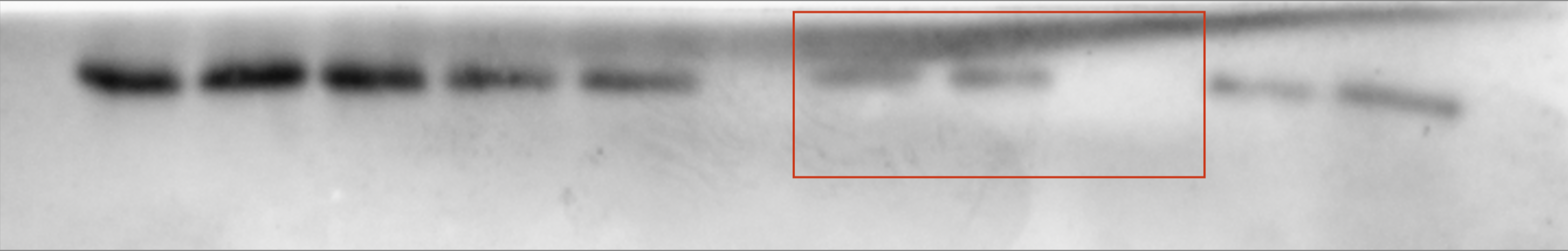

Supplement: Supplementary file 2 — Source data Fig. 1 [file 44318_2025_602_MOESM2_ESM.zip › Fig 1/E/iPOND WB H2B Screenshot.png]

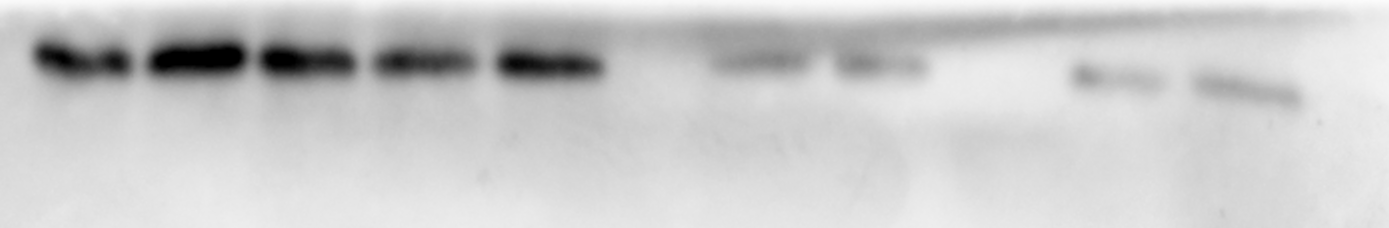

Supplement: Supplementary file 2 — Source data Fig. 1 [file 44318_2025_602_MOESM2_ESM.zip › Fig 1/E/iPOND WB H3.tif]

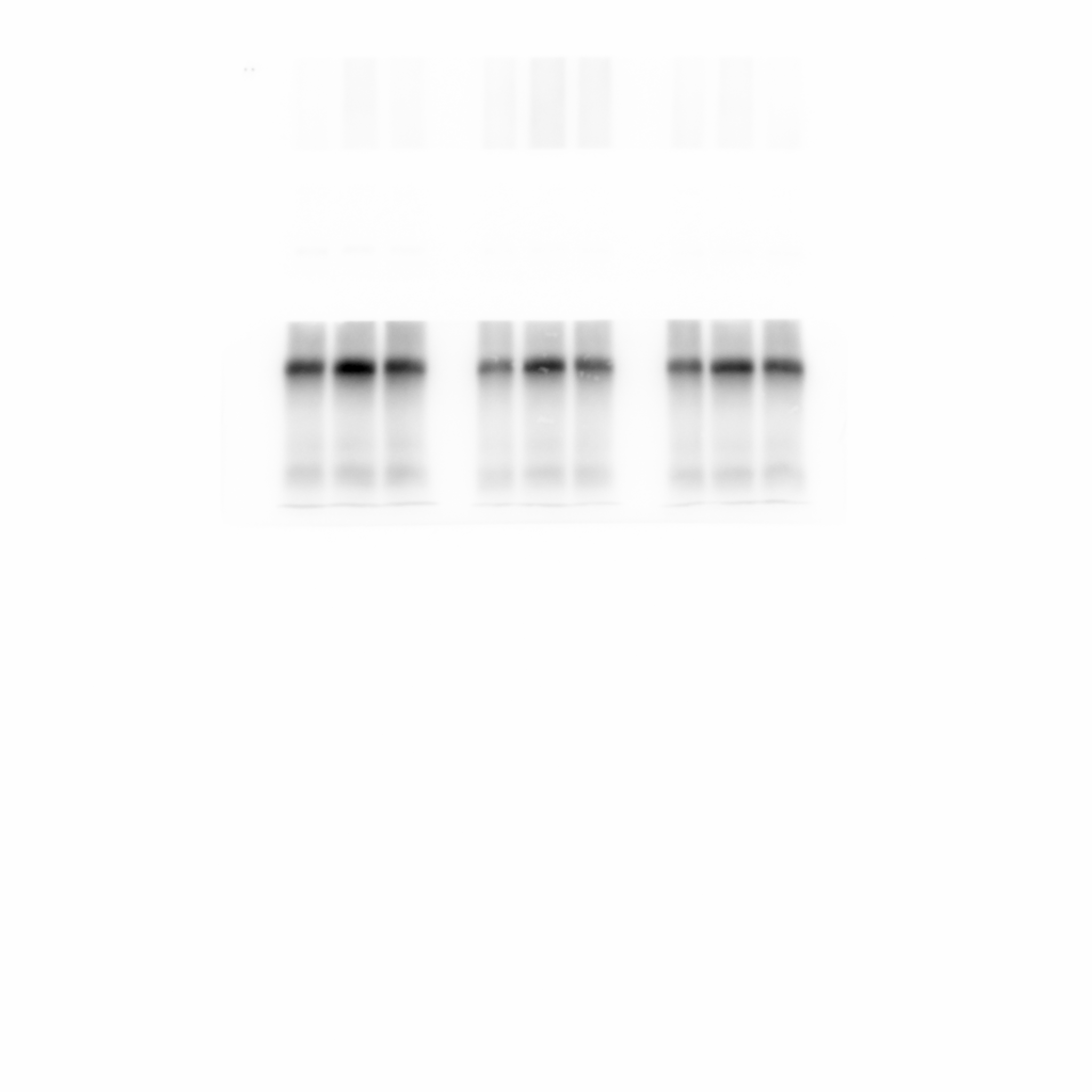

Supplement: Supplementary file 2 — Source data Fig. 1 [file 44318_2025_602_MOESM2_ESM.zip › Fig 1/E/INPUT WB H2Bub.Tif]

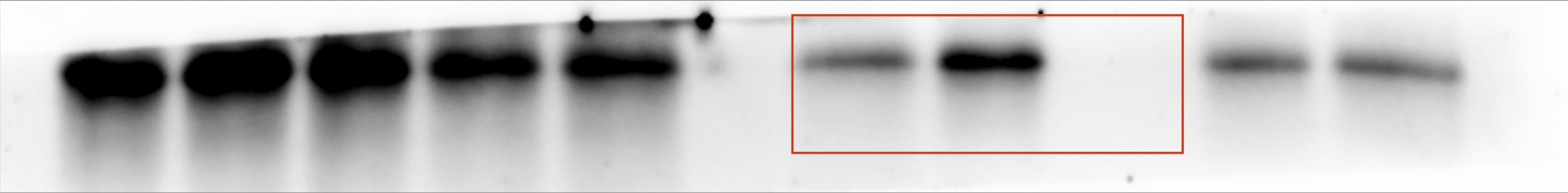

Supplement: Supplementary file 2 — Source data Fig. 1 [file 44318_2025_602_MOESM2_ESM.zip › Fig 1/E/iPOND WB H2Bub Screenshot.tiff]

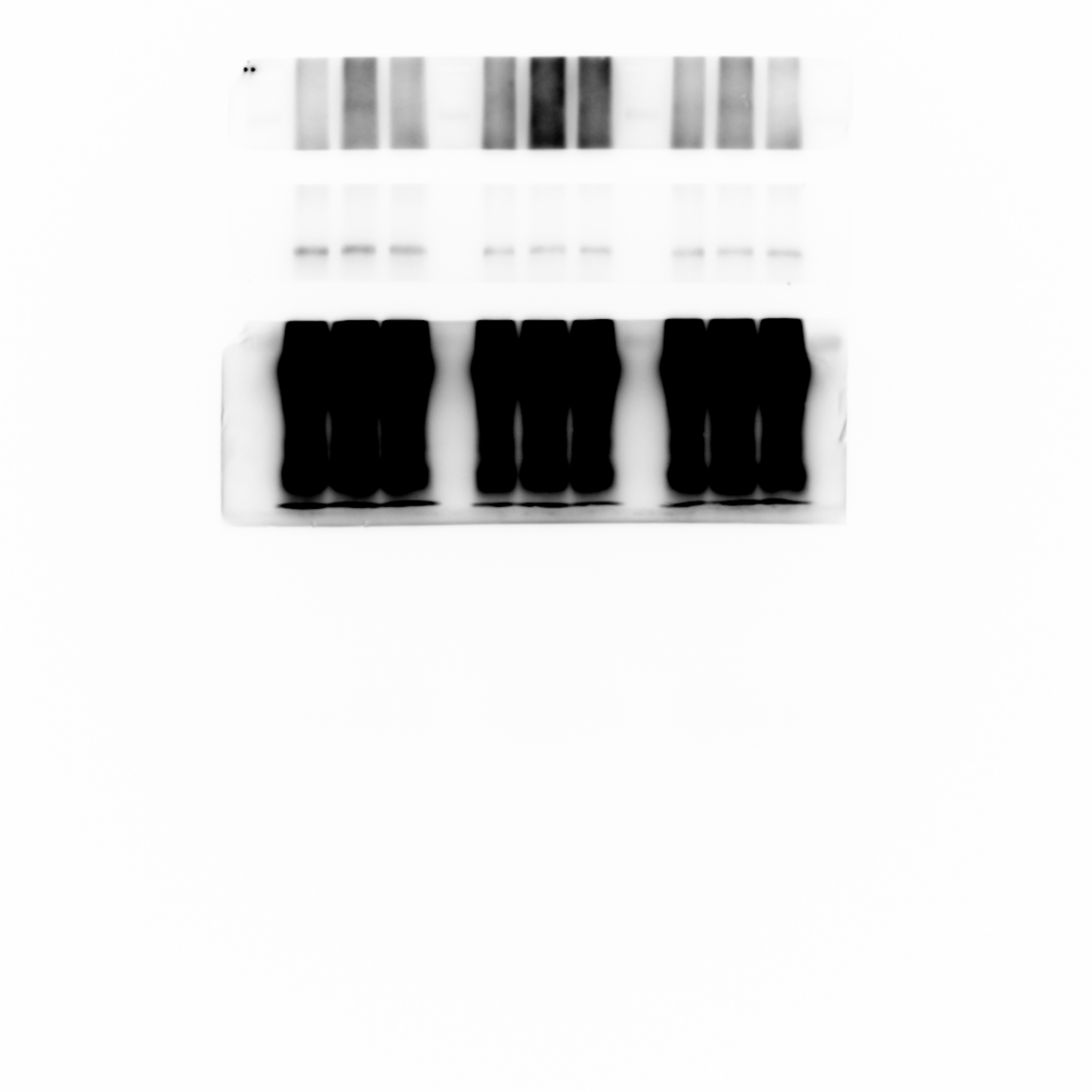

Supplement: Supplementary file 2 — Source data Fig. 1 [file 44318_2025_602_MOESM2_ESM.zip › Fig 1/E/INPUT WB pCHK1.Tif]

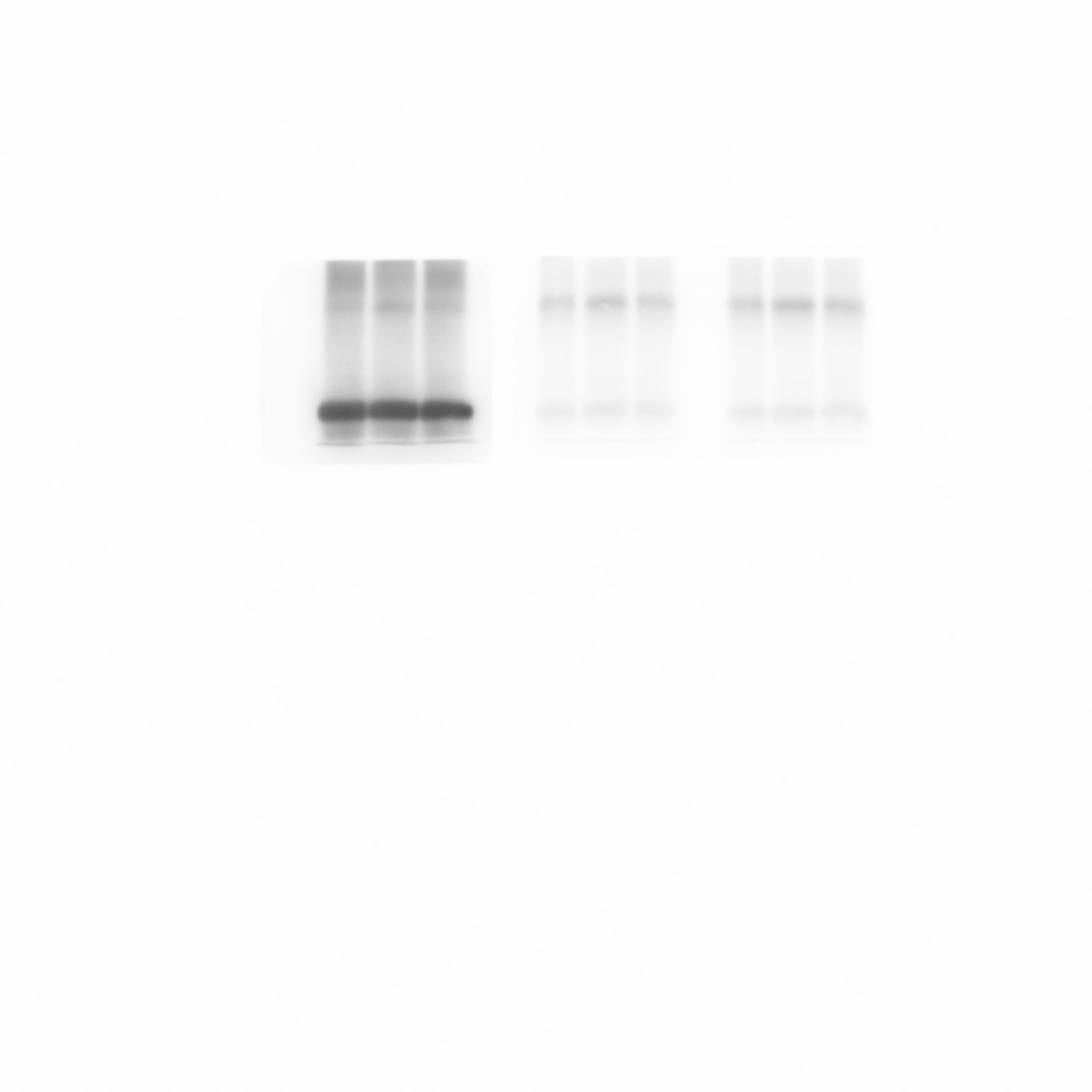

Supplement: Supplementary file 2 — Source data Fig. 1 [file 44318_2025_602_MOESM2_ESM.zip › Fig 1/E/INPUT WB H3.Tif]

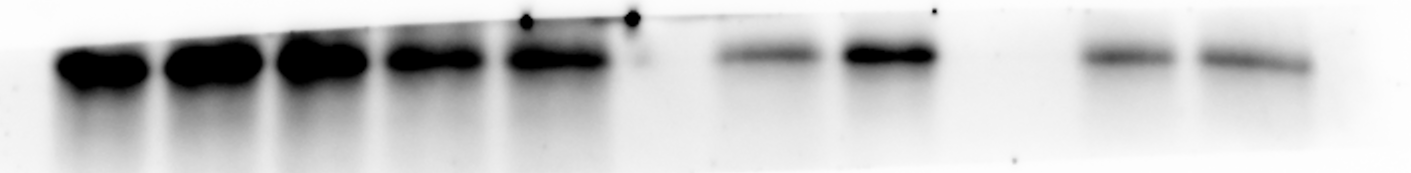

Supplement: Supplementary file 2 — Source data Fig. 1 [file 44318_2025_602_MOESM2_ESM.zip › Fig 1/E/iPOND WB H2Bub.tif]

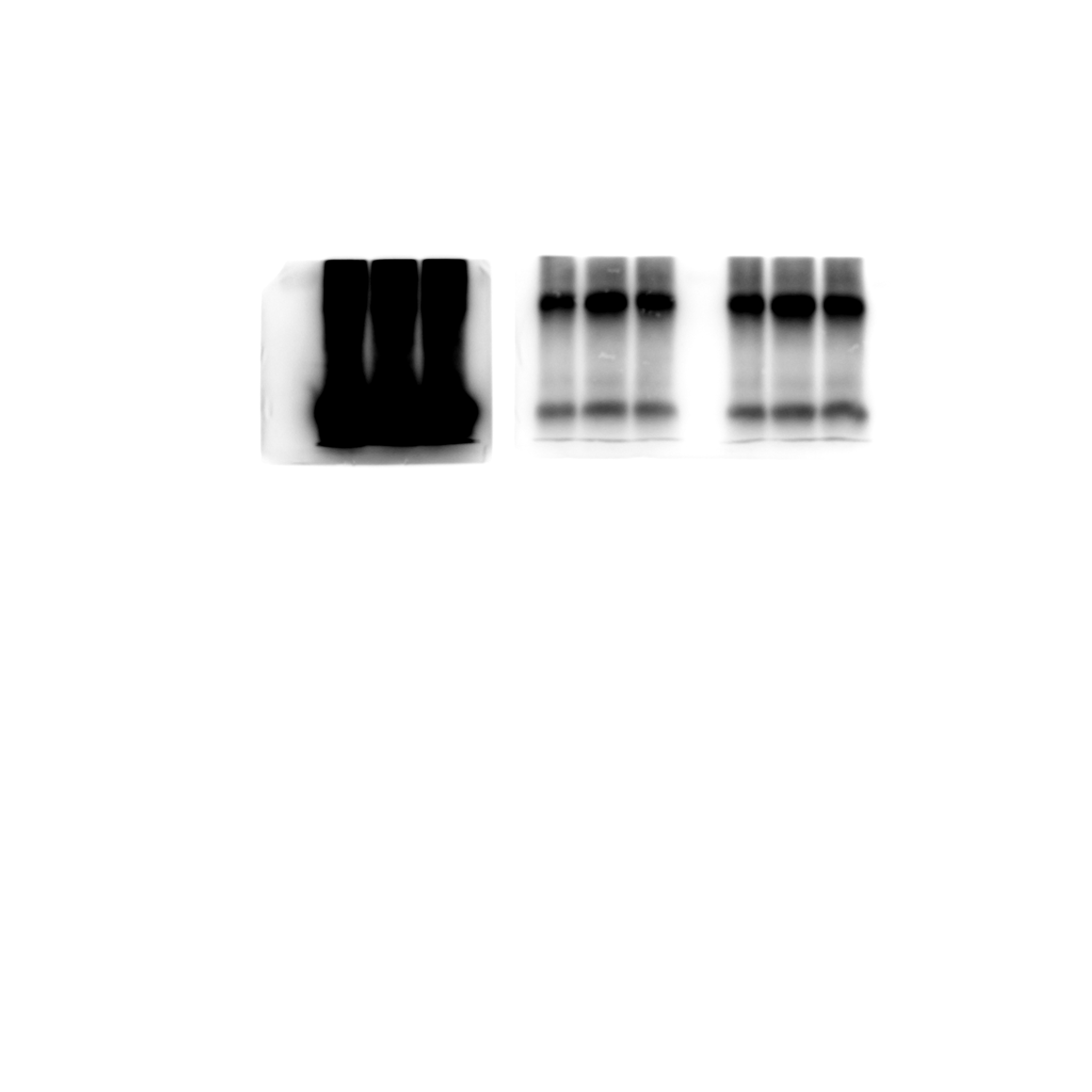

Supplement: Supplementary file 2 — Source data Fig. 1 [file 44318_2025_602_MOESM2_ESM.zip › Fig 1/E/INPUT WB H2B.Tif]

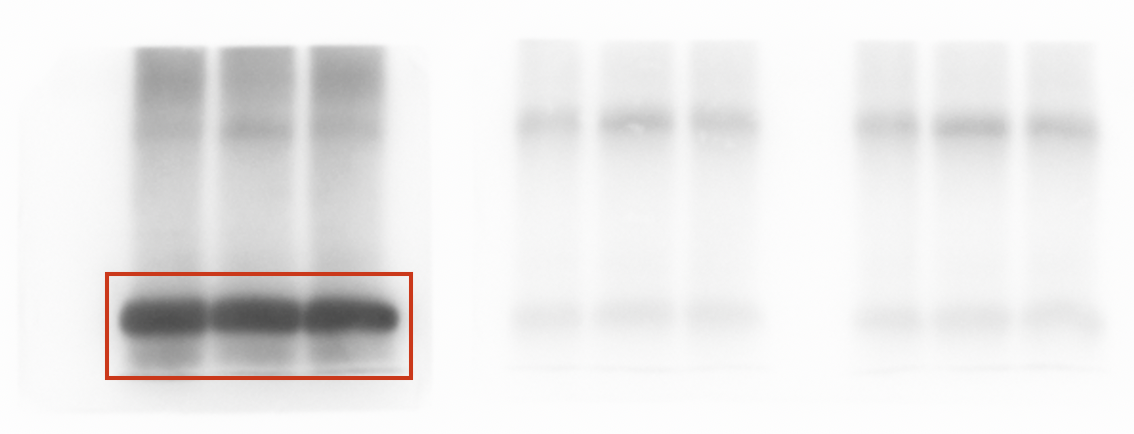

Supplement: Supplementary file 2 — Source data Fig. 1 [file 44318_2025_602_MOESM2_ESM.zip › Fig 1/E/INPUT WB H3 Screenshot.png]

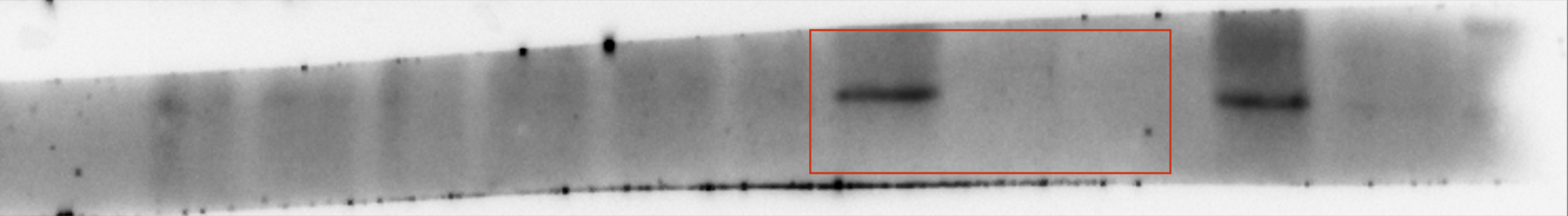

Supplement: Supplementary file 2 — Source data Fig. 1 [file 44318_2025_602_MOESM2_ESM.zip › Fig 1/E/iPOND WB PCNA Screenshot.tiff]

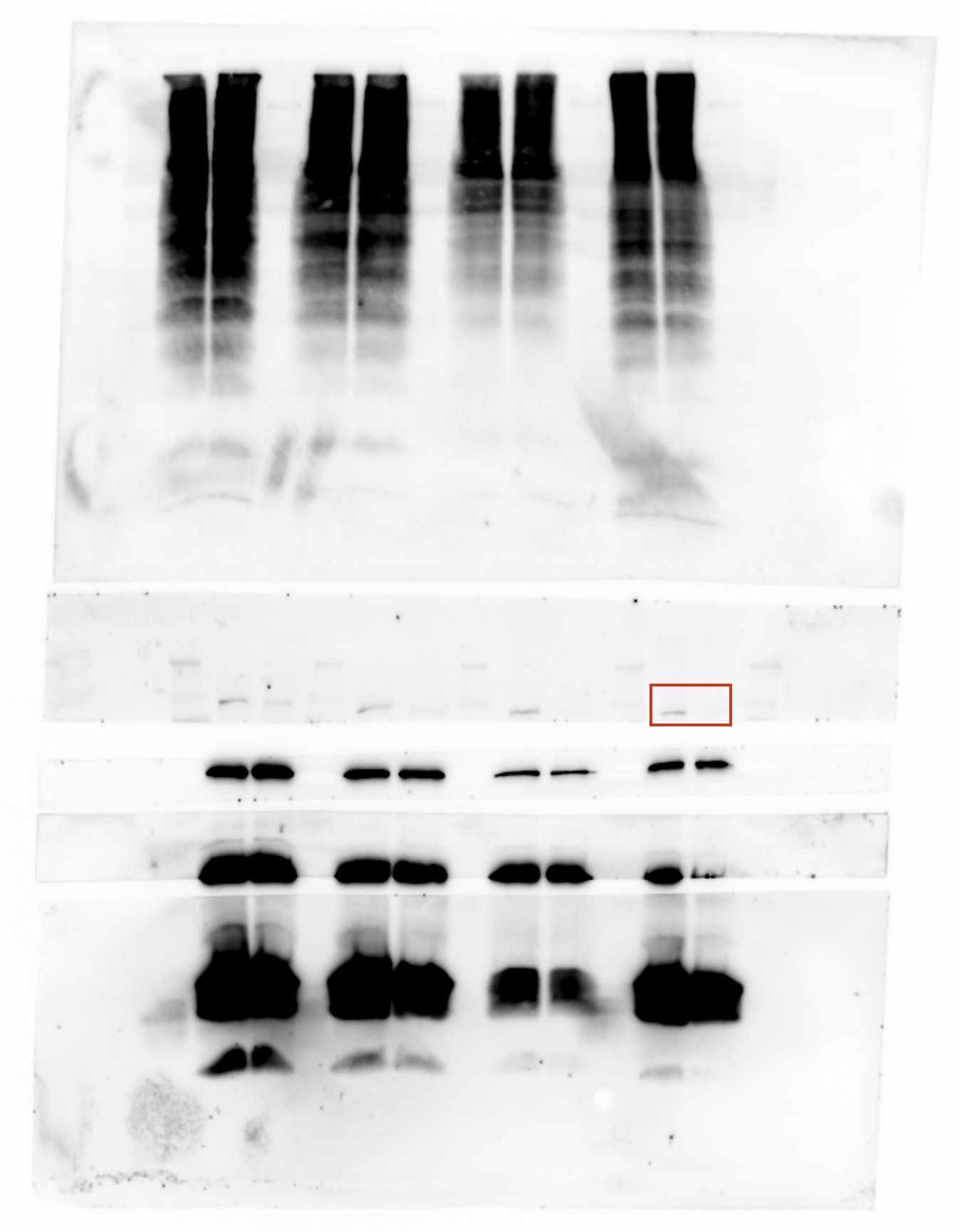

Supplement: Supplementary file 3 — Source data Fig. 2 [file 44318_2025_602_MOESM3_ESM.zip › Fig 2/A/WB RNF20 Screenshot.png]

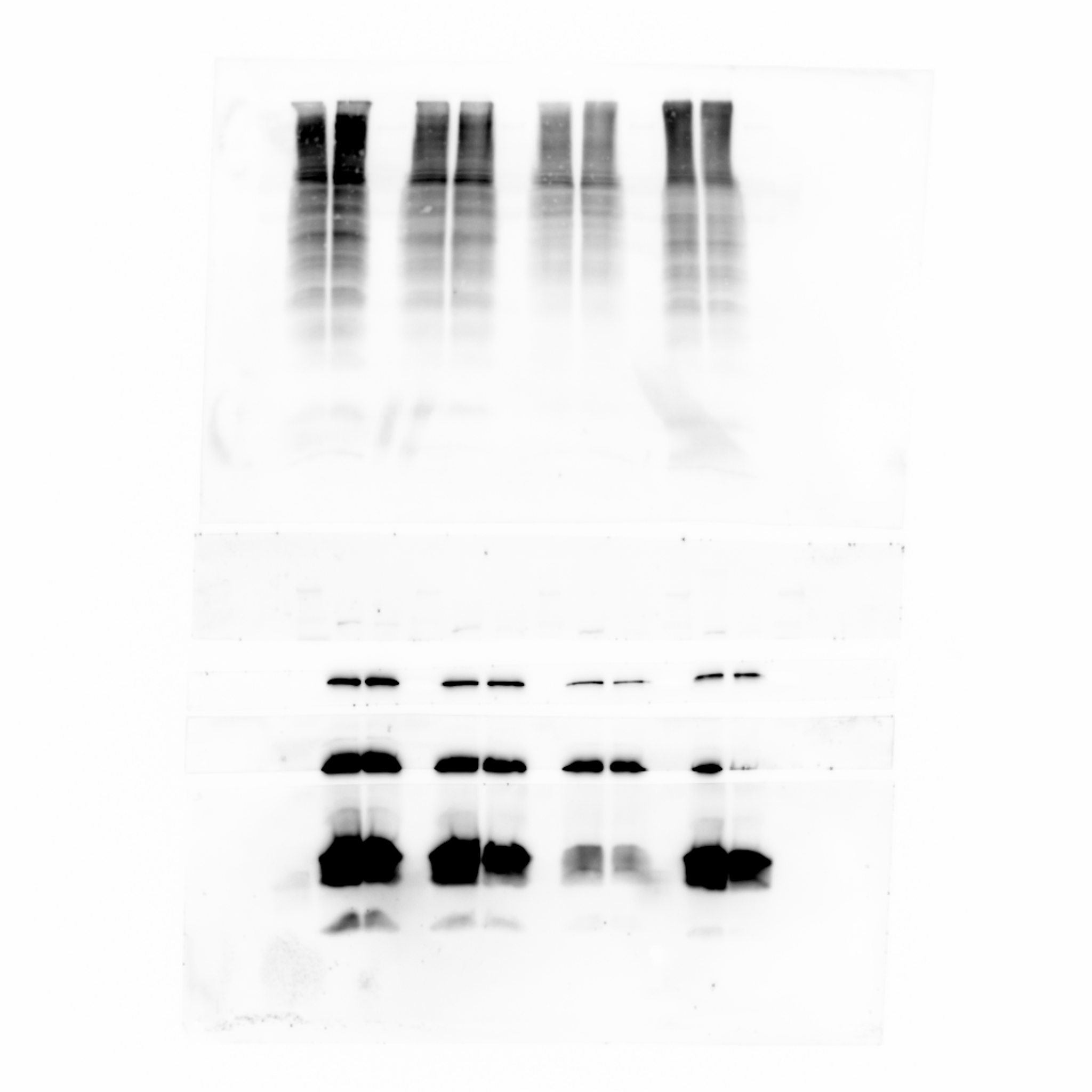

Supplement: Supplementary file 3 — Source data Fig. 2 [file 44318_2025_602_MOESM3_ESM.zip › Fig 2/A/WB Lamin.tif]

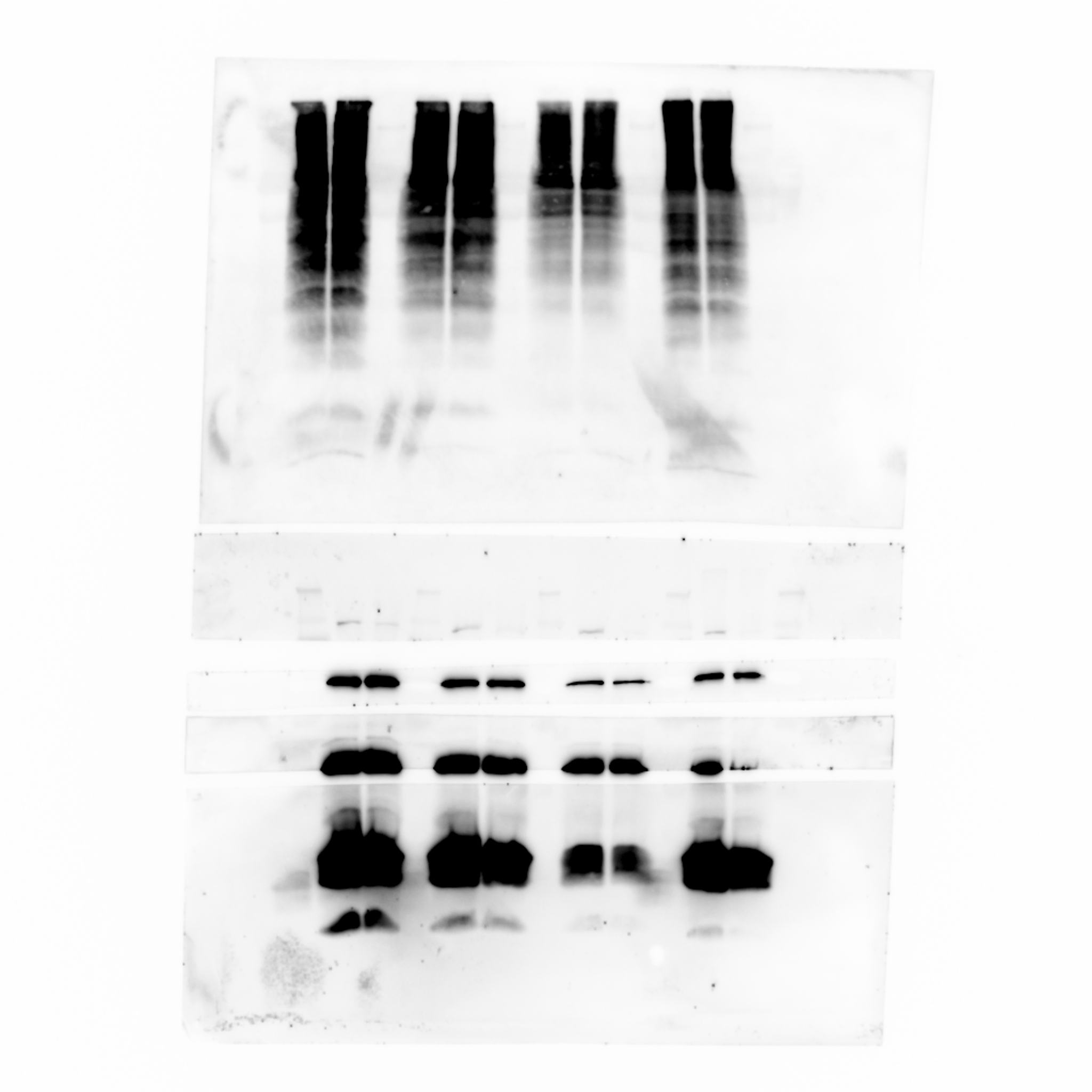

Supplement: Supplementary file 3 — Source data Fig. 2 [file 44318_2025_602_MOESM3_ESM.zip › Fig 2/A/WB RNF20.tif]

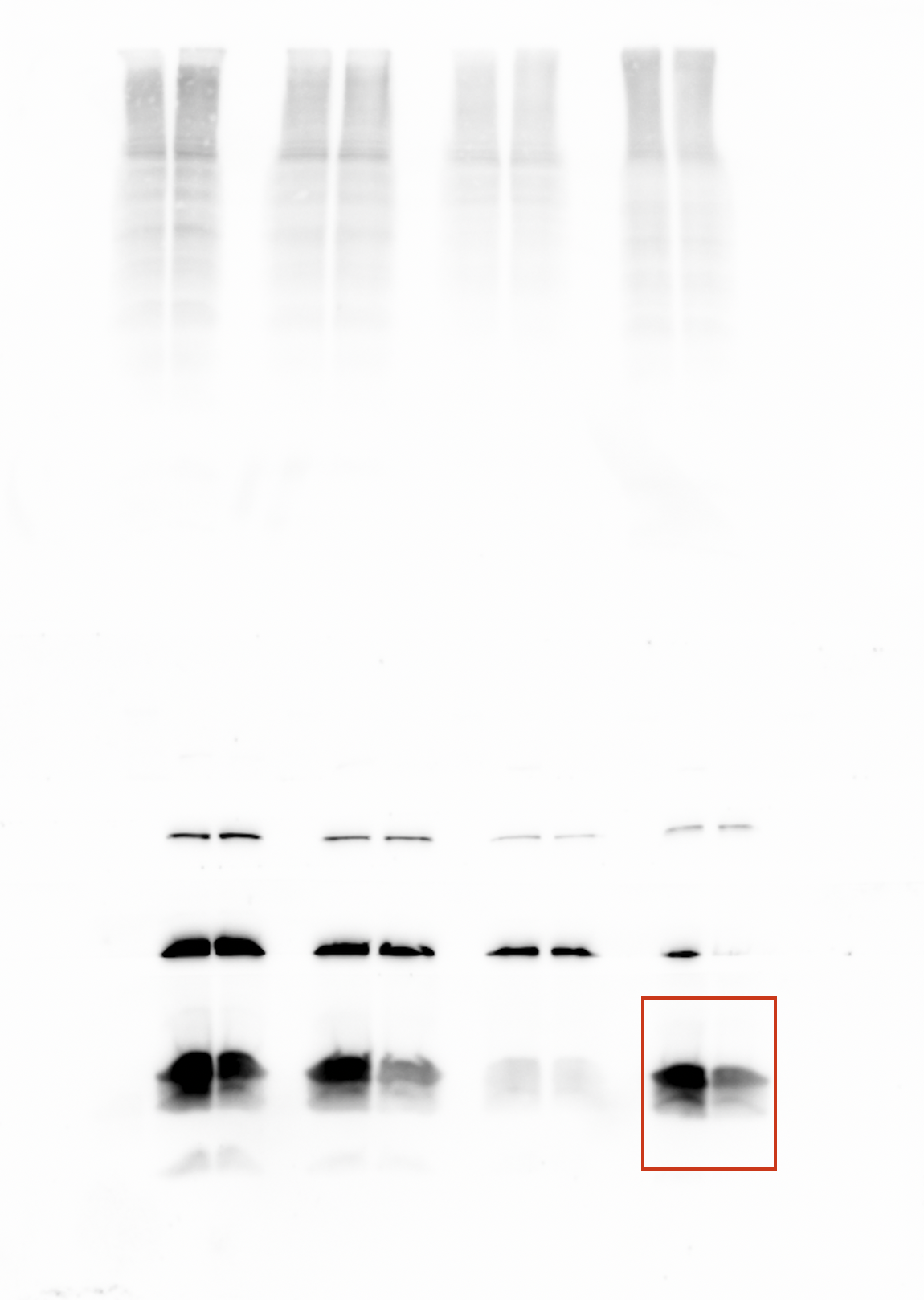

Supplement: Supplementary file 3 — Source data Fig. 2 [file 44318_2025_602_MOESM3_ESM.zip › Fig 2/A/WB H2Bub Screenshot.png]

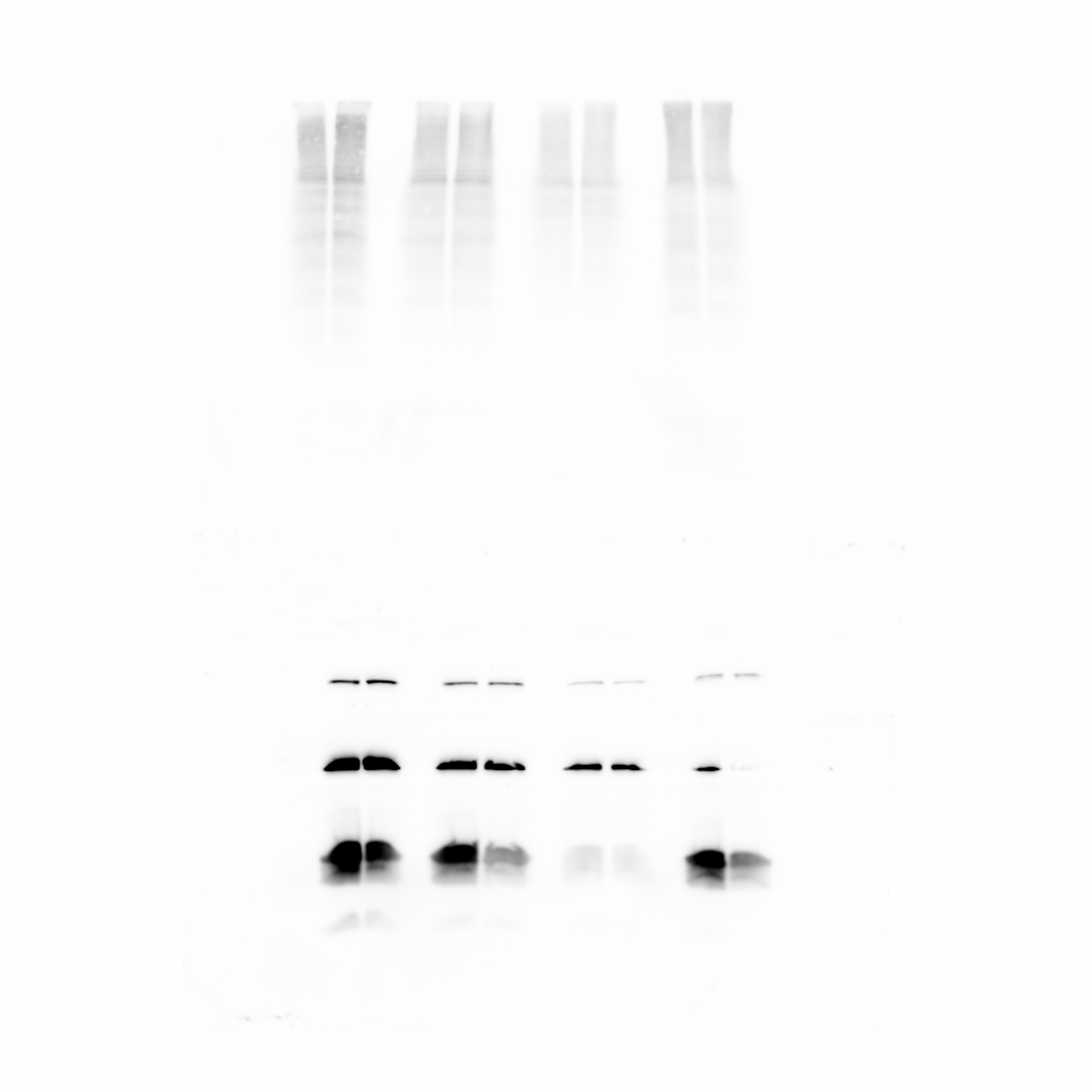

Supplement: Supplementary file 3 — Source data Fig. 2 [file 44318_2025_602_MOESM3_ESM.zip › Fig 2/A/WB H2Bub.tif]

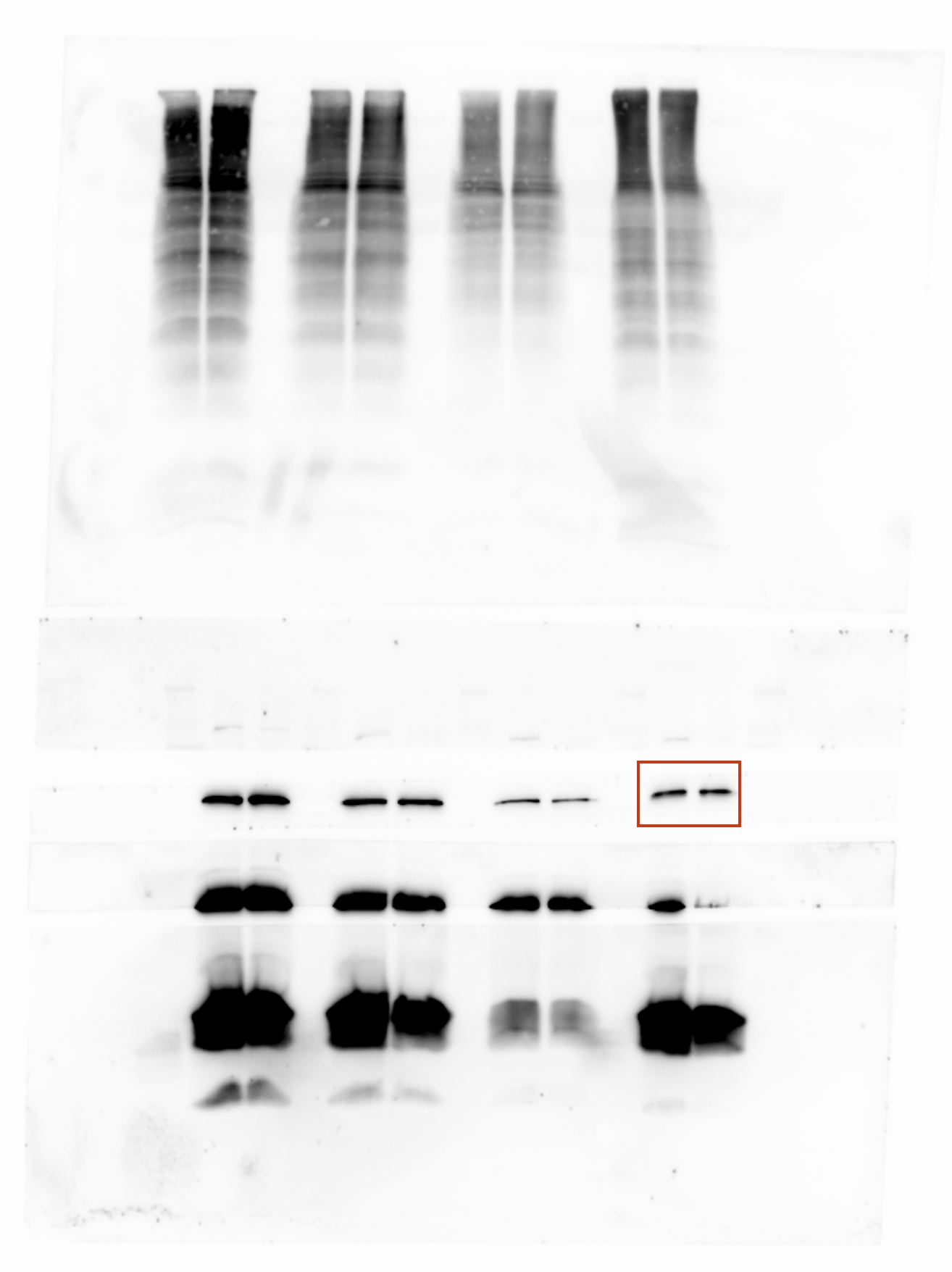

Supplement: Supplementary file 3 — Source data Fig. 2 [file 44318_2025_602_MOESM3_ESM.zip › Fig 2/A/WB Lamin Screenshot.png]

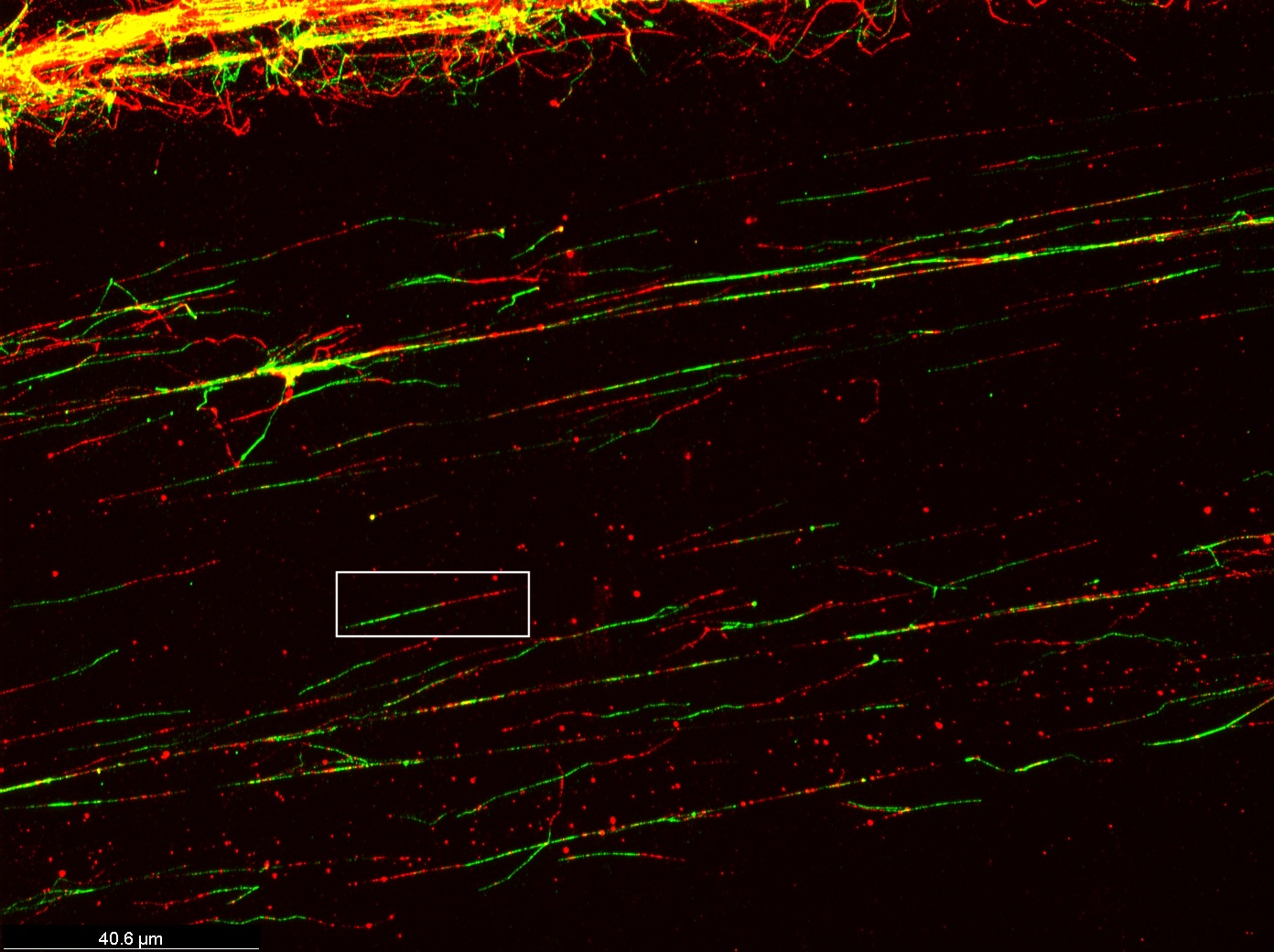

Supplement: Supplementary file 3 — Source data Fig. 2 [file 44318_2025_602_MOESM3_ESM.zip › Fig 2/E/siRNF20_CPT.jpg]

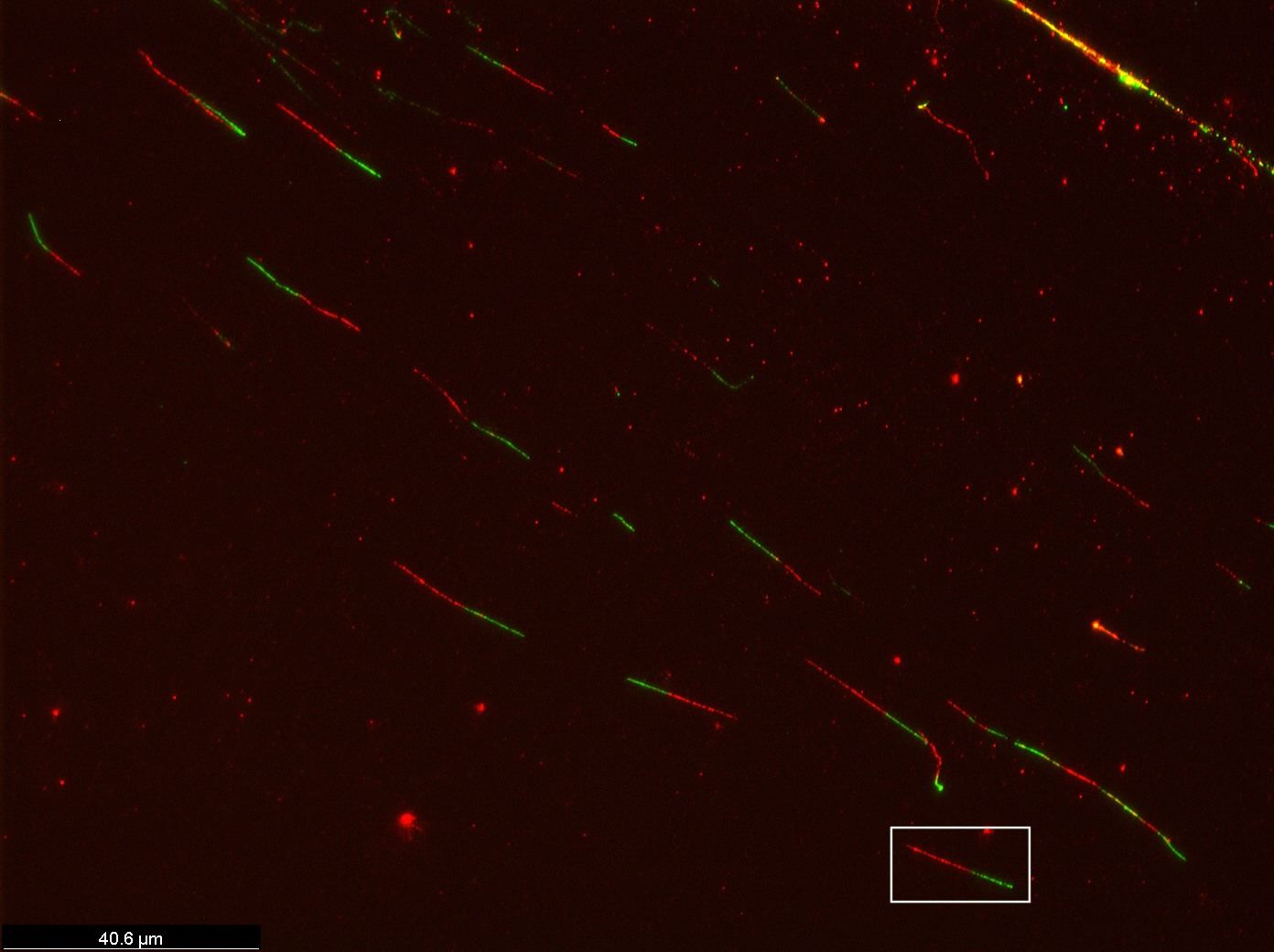

Supplement: Supplementary file 3 — Source data Fig. 2 [file 44318_2025_602_MOESM3_ESM.zip › Fig 2/E/siLuc CPT.jpg]

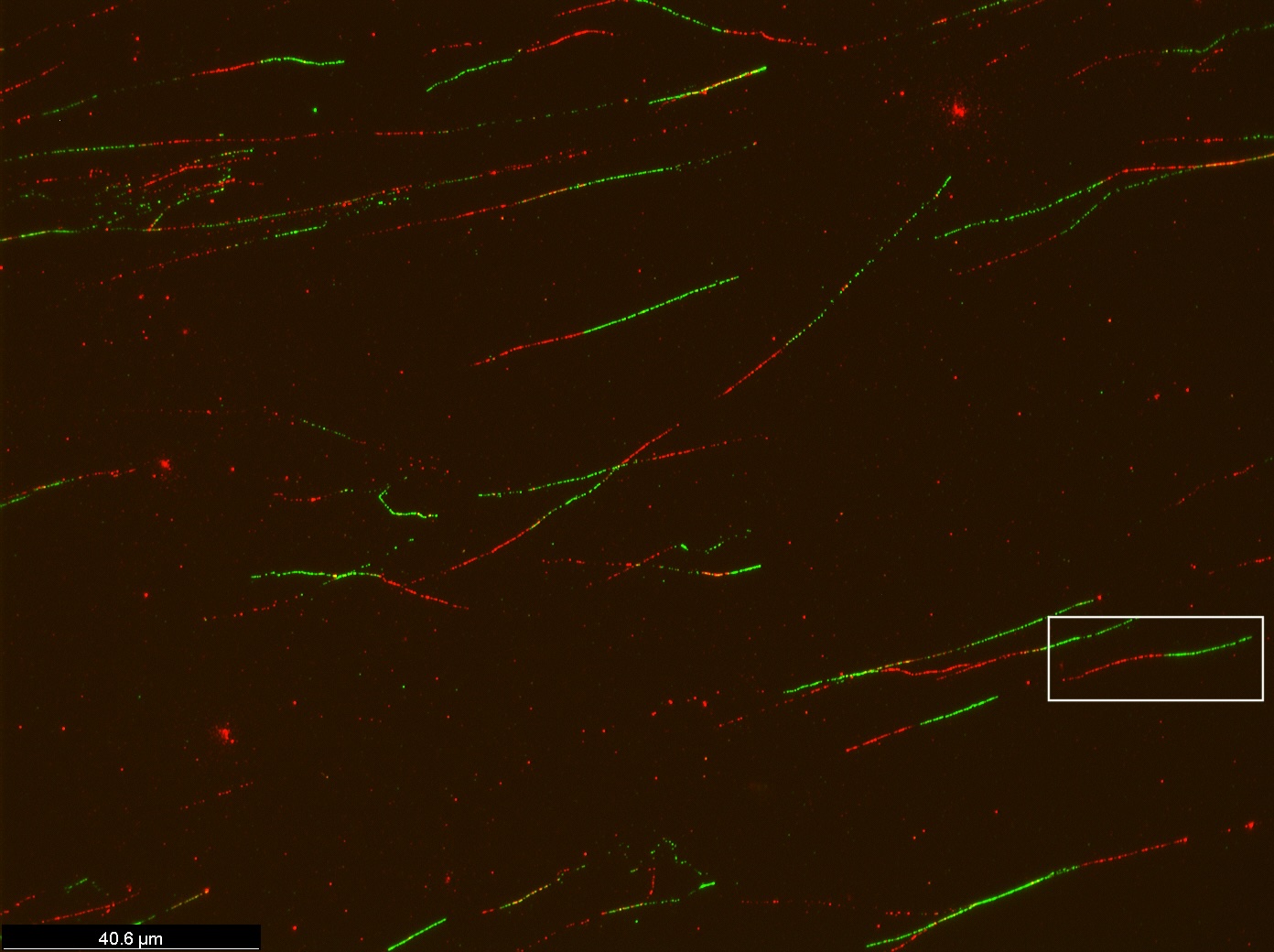

Supplement: Supplementary file 3 — Source data Fig. 2 [file 44318_2025_602_MOESM3_ESM.zip › Fig 2/E/siRNF20_UT.jpg]

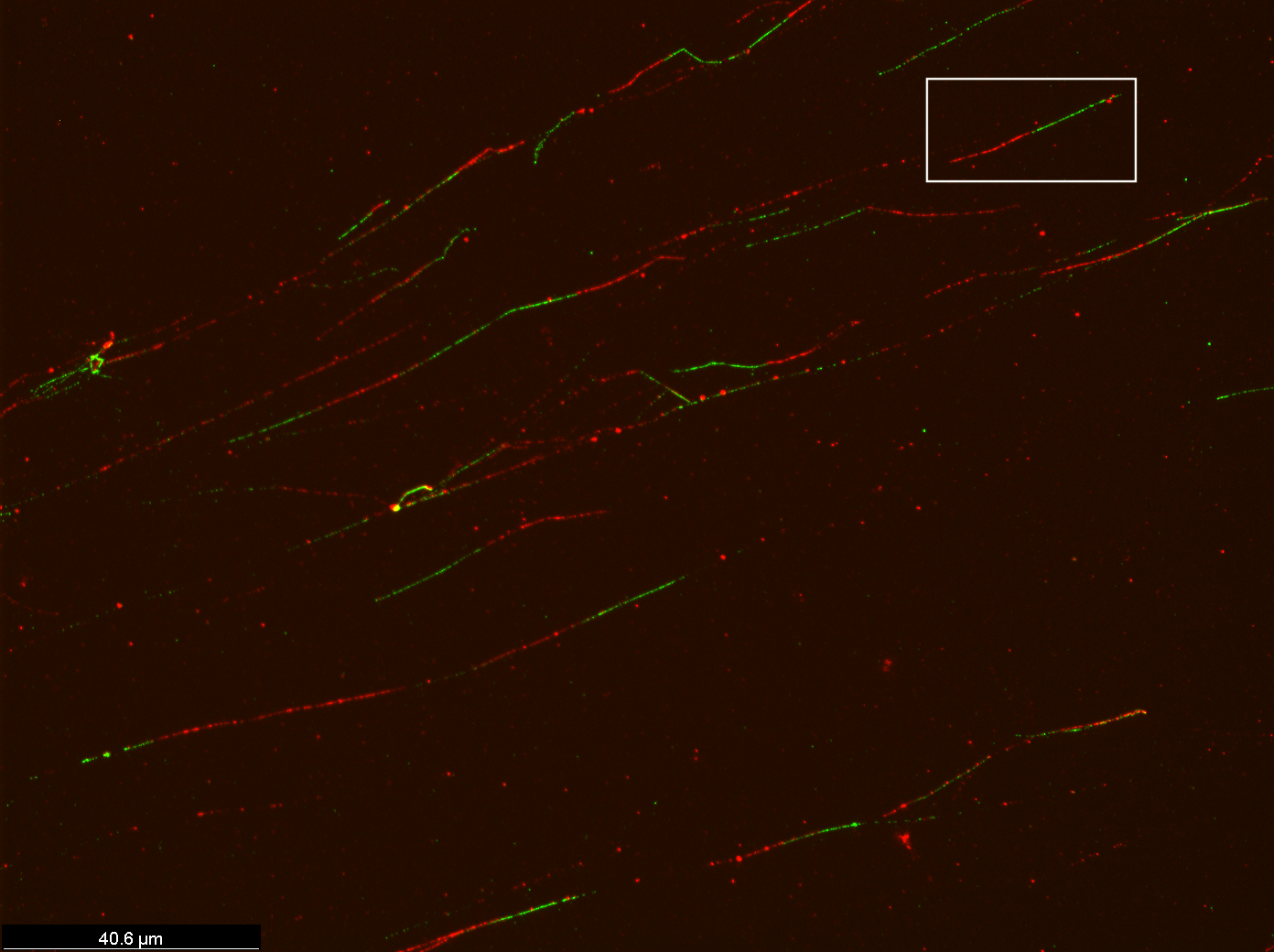

Supplement: Supplementary file 3 — Source data Fig. 2 [file 44318_2025_602_MOESM3_ESM.zip › Fig 2/E/siLuc UT.tif]

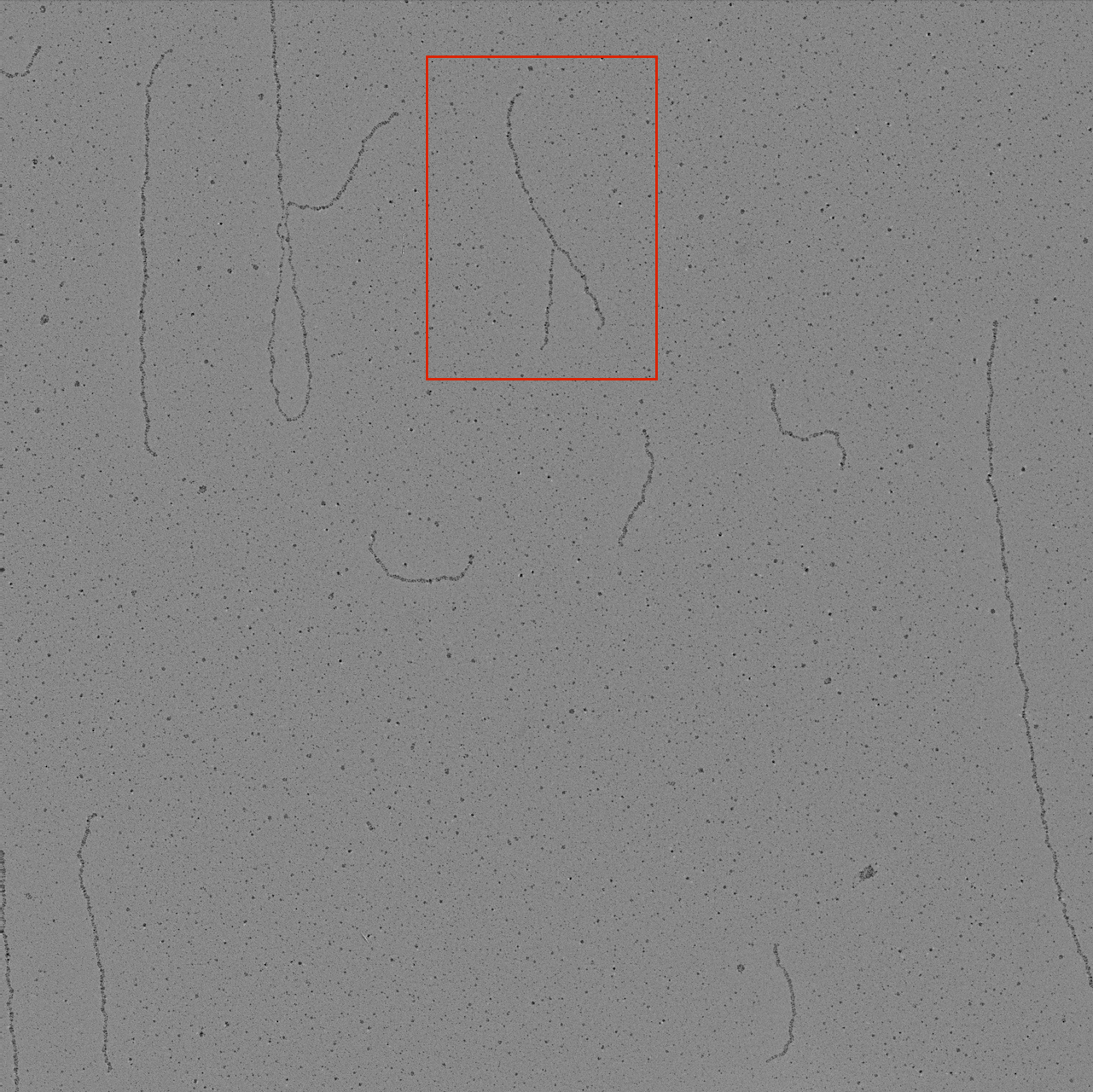

Supplement: Supplementary file 4 — Source data Fig. 3 [file 44318_2025_602_MOESM4_ESM.zip › Fig 3/G/EM Screenshot.png]

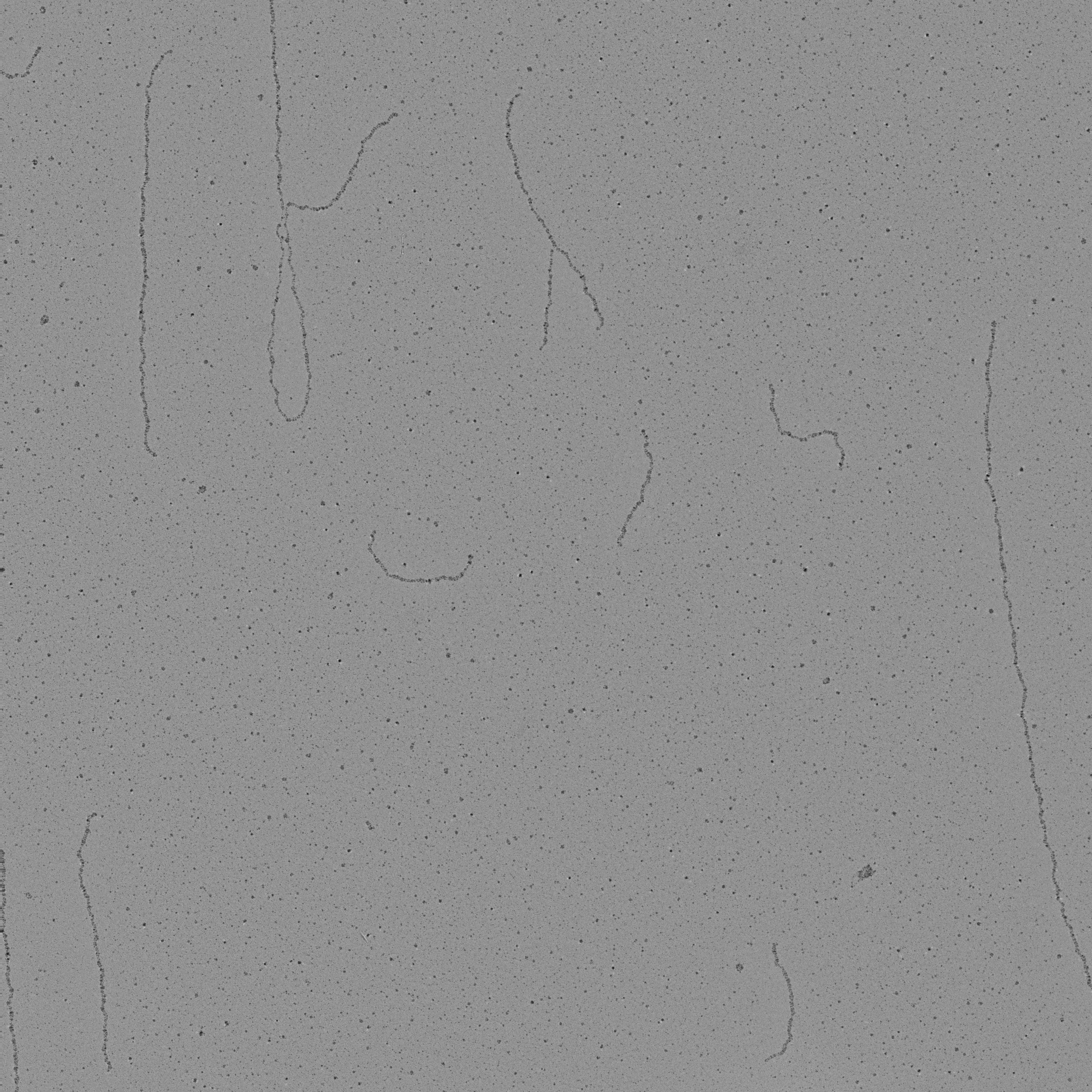

Supplement: Supplementary file 4 — Source data Fig. 3 [file 44318_2025_602_MOESM4_ESM.zip › Fig 3/G/EM.tif]

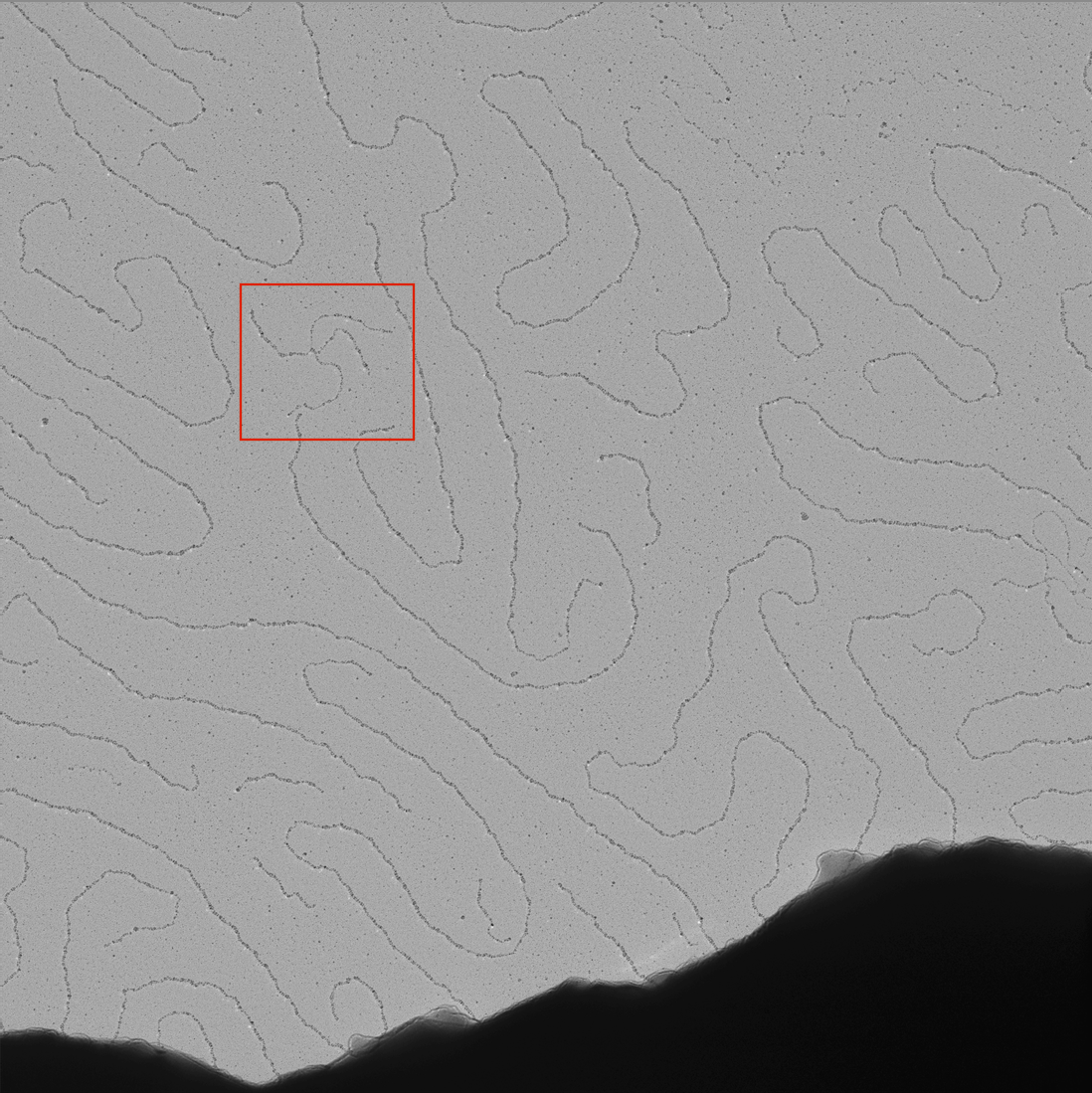

Supplement: Supplementary file 4 — Source data Fig. 3 [file 44318_2025_602_MOESM4_ESM.zip › Fig 3/H/EM Screenshot.png]

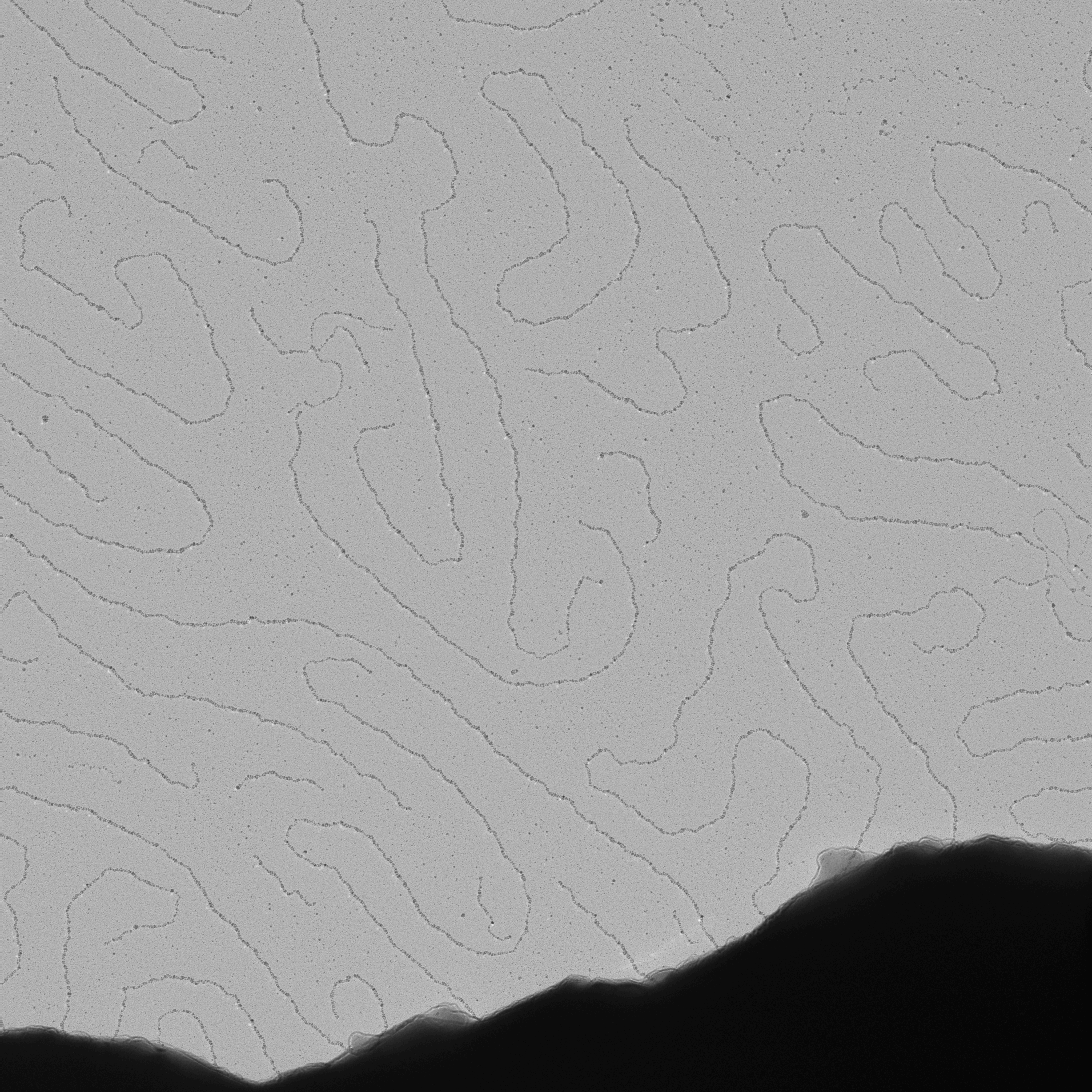

Supplement: Supplementary file 4 — Source data Fig. 3 [file 44318_2025_602_MOESM4_ESM.zip › Fig 3/H/EM.tif]

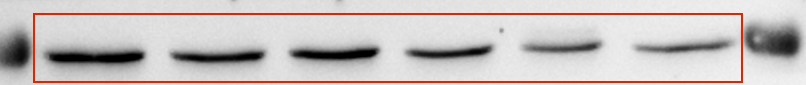

Supplement: Supplementary file 4 — Source data Fig. 3 [file 44318_2025_602_MOESM4_ESM.zip › Fig 3/D/WB CHK1.tiff]

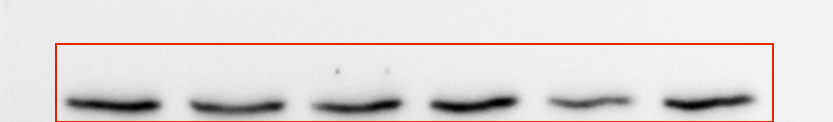

Supplement: Supplementary file 4 — Source data Fig. 3 [file 44318_2025_602_MOESM4_ESM.zip › Fig 3/D/WB RPA.tiff]

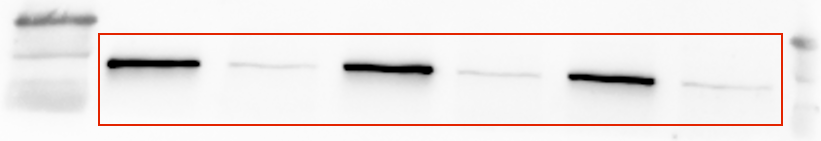

Supplement: Supplementary file 4 — Source data Fig. 3 [file 44318_2025_602_MOESM4_ESM.zip › Fig 3/D/WB RNF20.tiff]

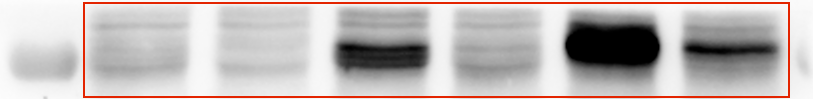

Supplement: Supplementary file 4 — Source data Fig. 3 [file 44318_2025_602_MOESM4_ESM.zip › Fig 3/D/WB pCHK1.tiff]

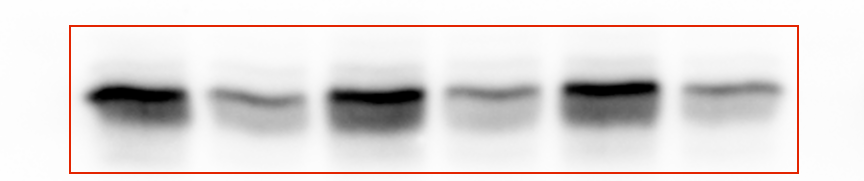

Supplement: Supplementary file 4 — Source data Fig. 3 [file 44318_2025_602_MOESM4_ESM.zip › Fig 3/D/WB H2Bub.tiff]

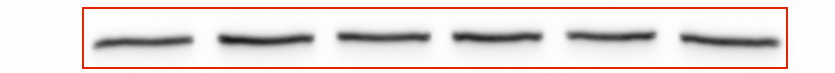

Supplement: Supplementary file 4 — Source data Fig. 3 [file 44318_2025_602_MOESM4_ESM.zip › Fig 3/D/WB Lamin.tiff]

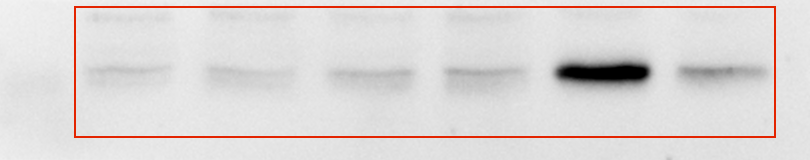

Supplement: Supplementary file 4 — Source data Fig. 3 [file 44318_2025_602_MOESM4_ESM.zip › Fig 3/D/WB pRPA.tiff]

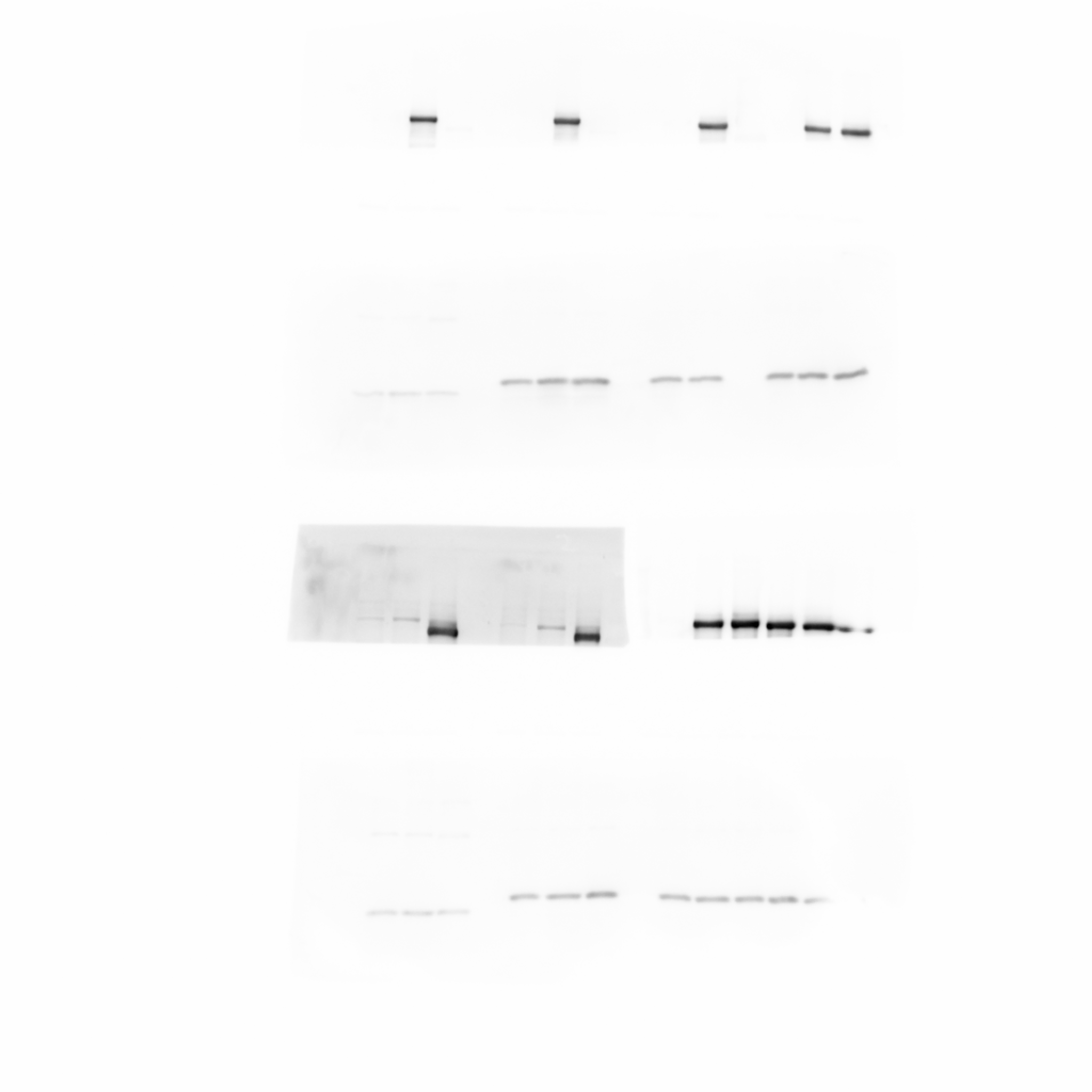

Supplement: Supplementary file 5 — Source data Fig. 4 [file 44318_2025_602_MOESM5_ESM.zip › Fig 4/F/RNF169.tif]

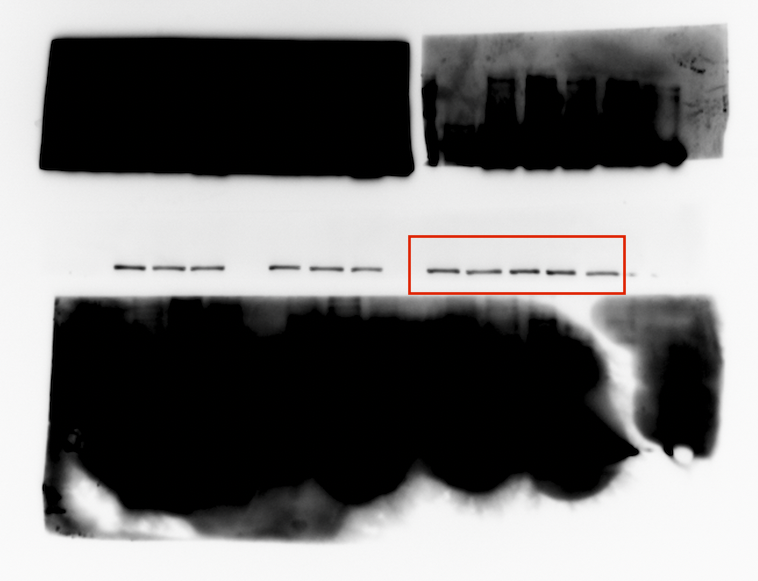

Supplement: Supplementary file 5 — Source data Fig. 4 [file 44318_2025_602_MOESM5_ESM.zip › Fig 4/F/b-actin Screenshot.png]

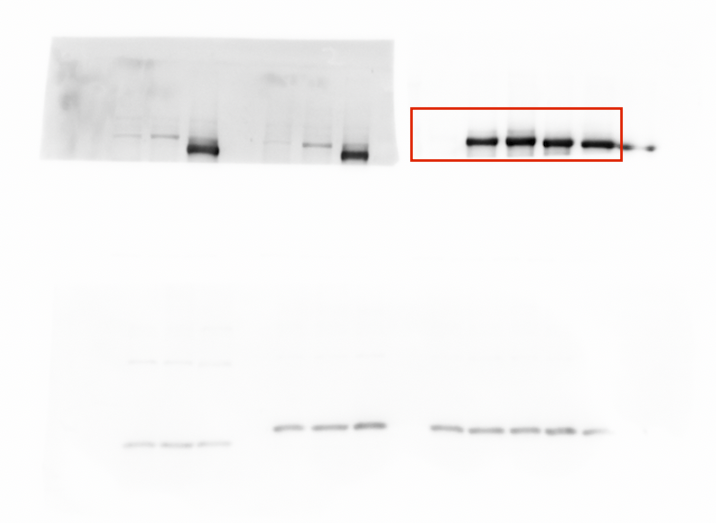

Supplement: Supplementary file 5 — Source data Fig. 4 [file 44318_2025_602_MOESM5_ESM.zip › Fig 4/F/RNF169 Screenshot.png]

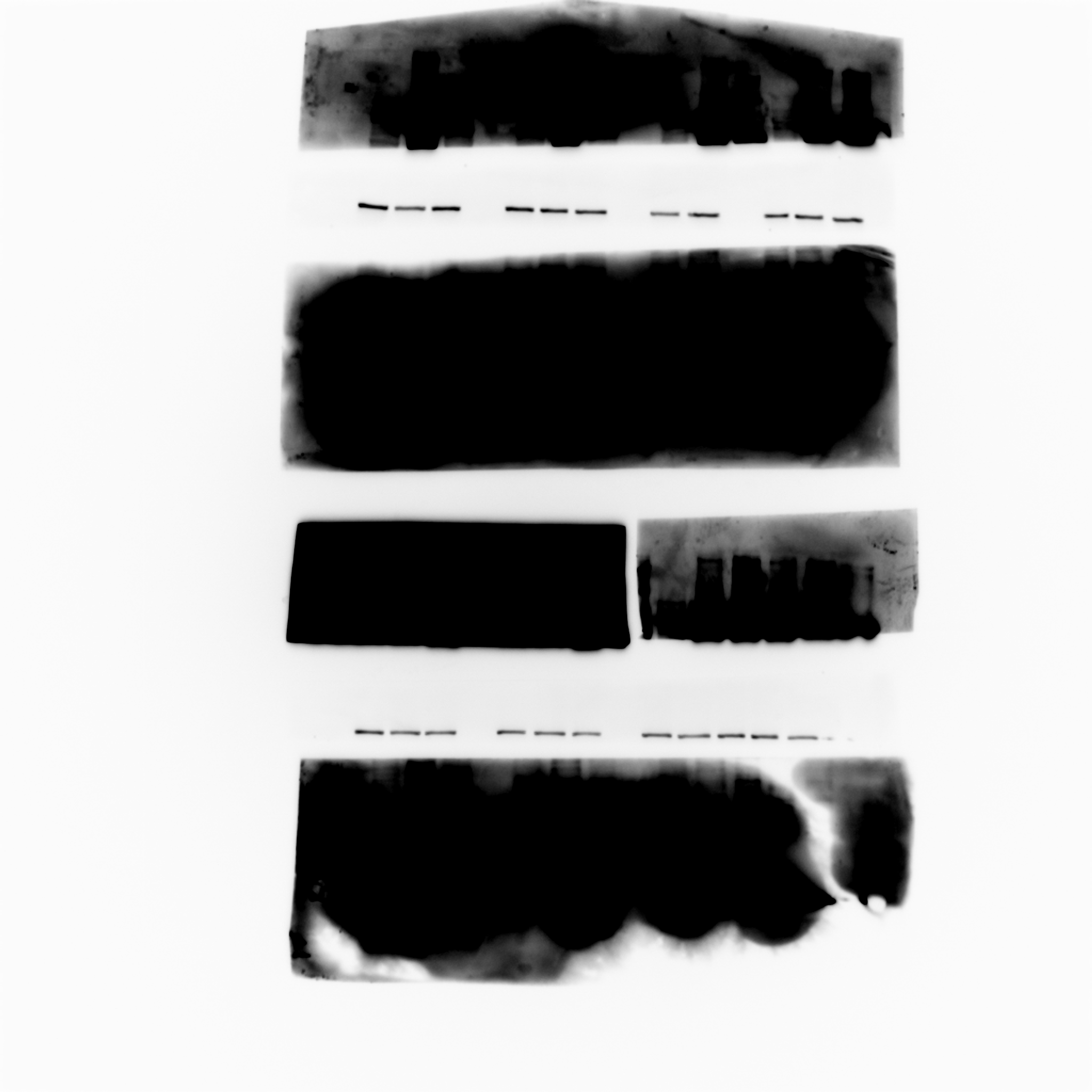

Supplement: Supplementary file 5 — Source data Fig. 4 [file 44318_2025_602_MOESM5_ESM.zip › Fig 4/F/b-actin.tif]

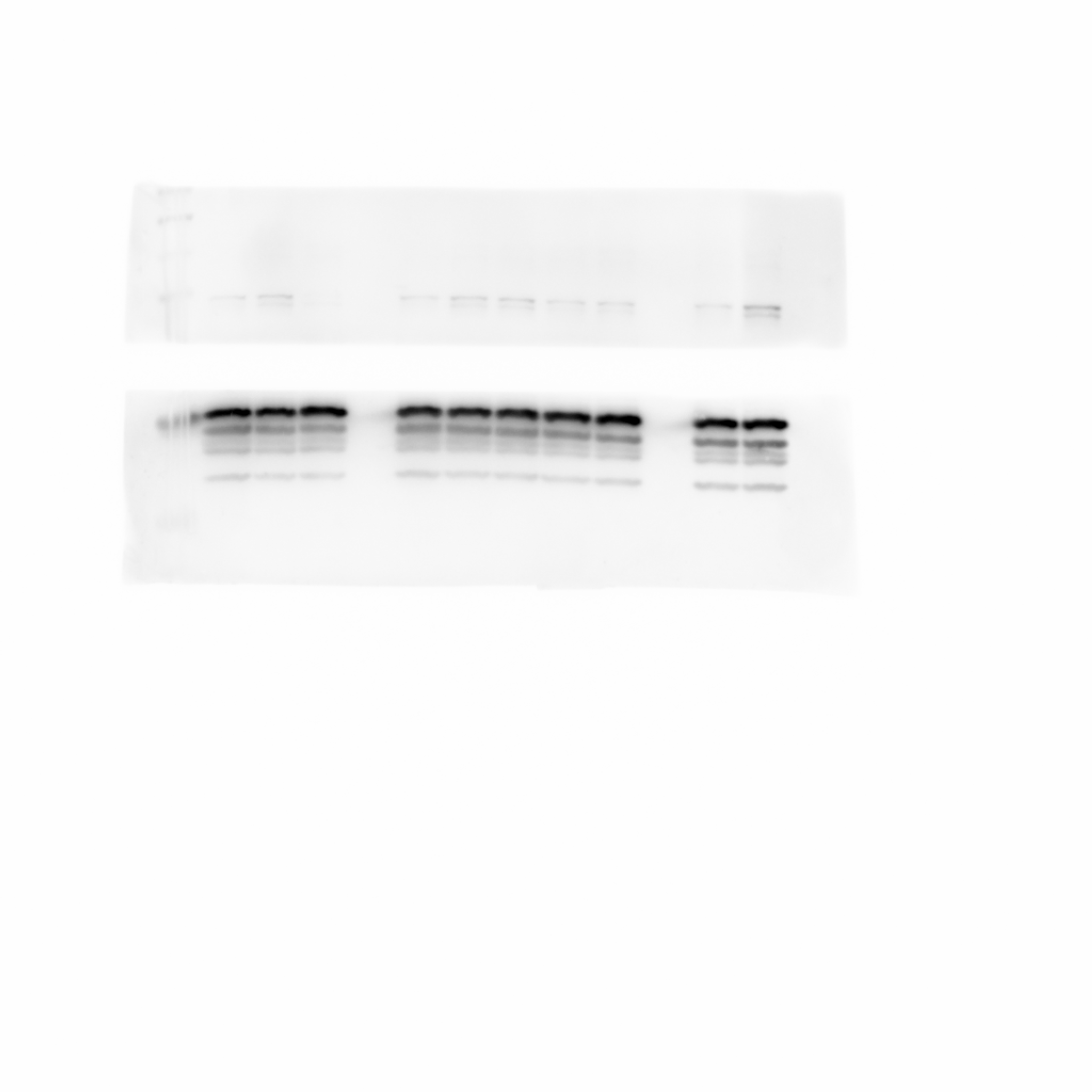

Supplement: Supplementary file 5 — Source data Fig. 4 [file 44318_2025_602_MOESM5_ESM.zip › Fig 4/F/FLAG_15kDa.tif]

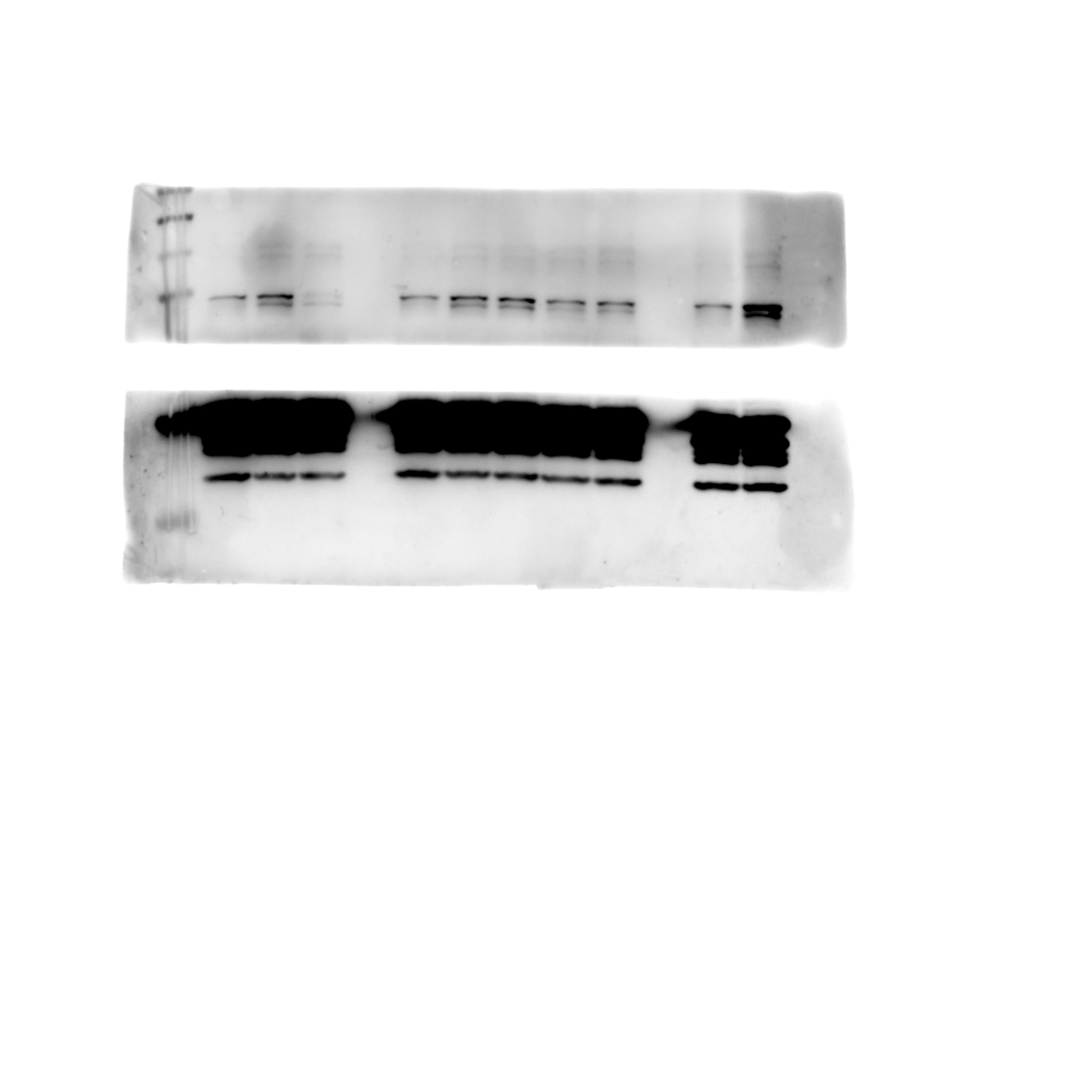

Supplement: Supplementary file 5 — Source data Fig. 4 [file 44318_2025_602_MOESM5_ESM.zip › Fig 4/F/FLAG_25kDa.tif]

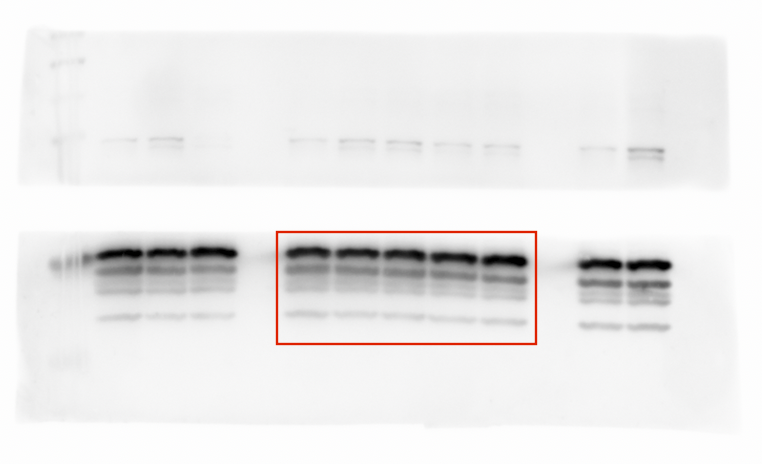

Supplement: Supplementary file 5 — Source data Fig. 4 [file 44318_2025_602_MOESM5_ESM.zip › Fig 4/F/FLAG_15kDa Screenshot.png]

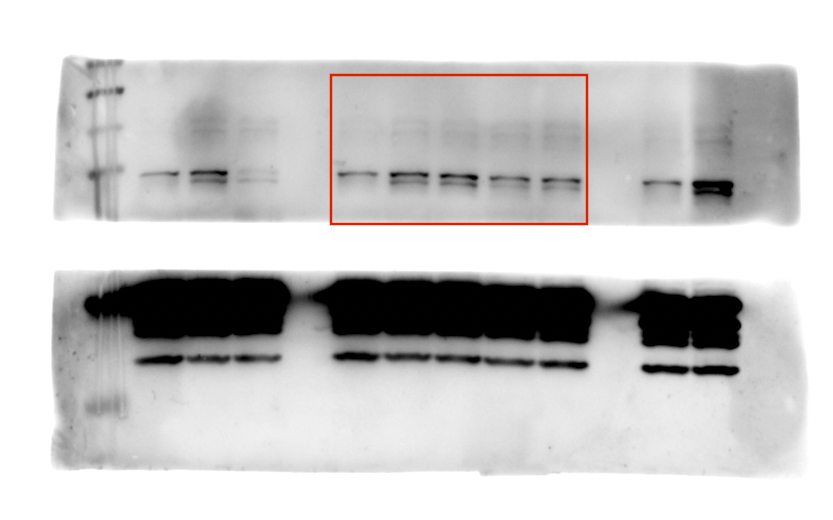

Supplement: Supplementary file 5 — Source data Fig. 4 [file 44318_2025_602_MOESM5_ESM.zip › Fig 4/F/FLAG_25kDa Screenshot.png]

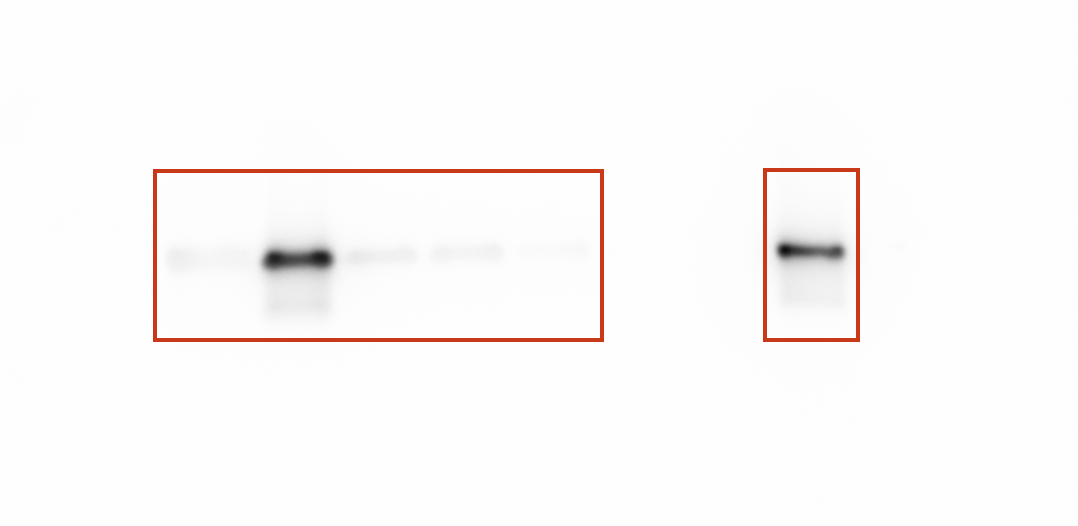

Supplement: Supplementary file 5 — Source data Fig. 4 [file 44318_2025_602_MOESM5_ESM.zip › Fig 4/C/H2Bub low exp Screenshot.png]

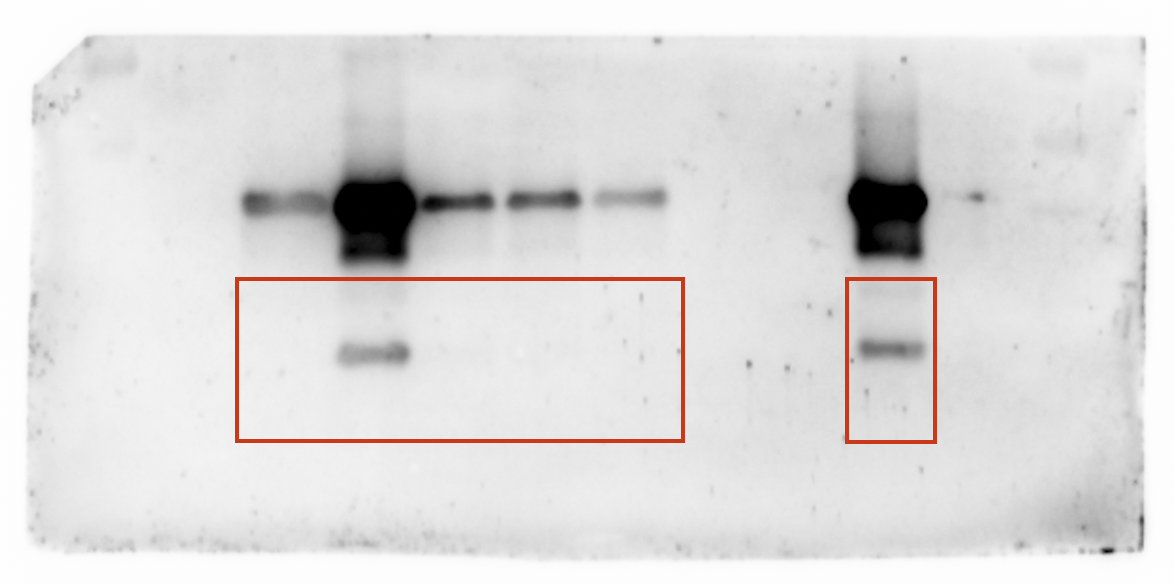

Supplement: Supplementary file 5 — Source data Fig. 4 [file 44318_2025_602_MOESM5_ESM.zip › Fig 4/C/WB H3 Screenshot.png]

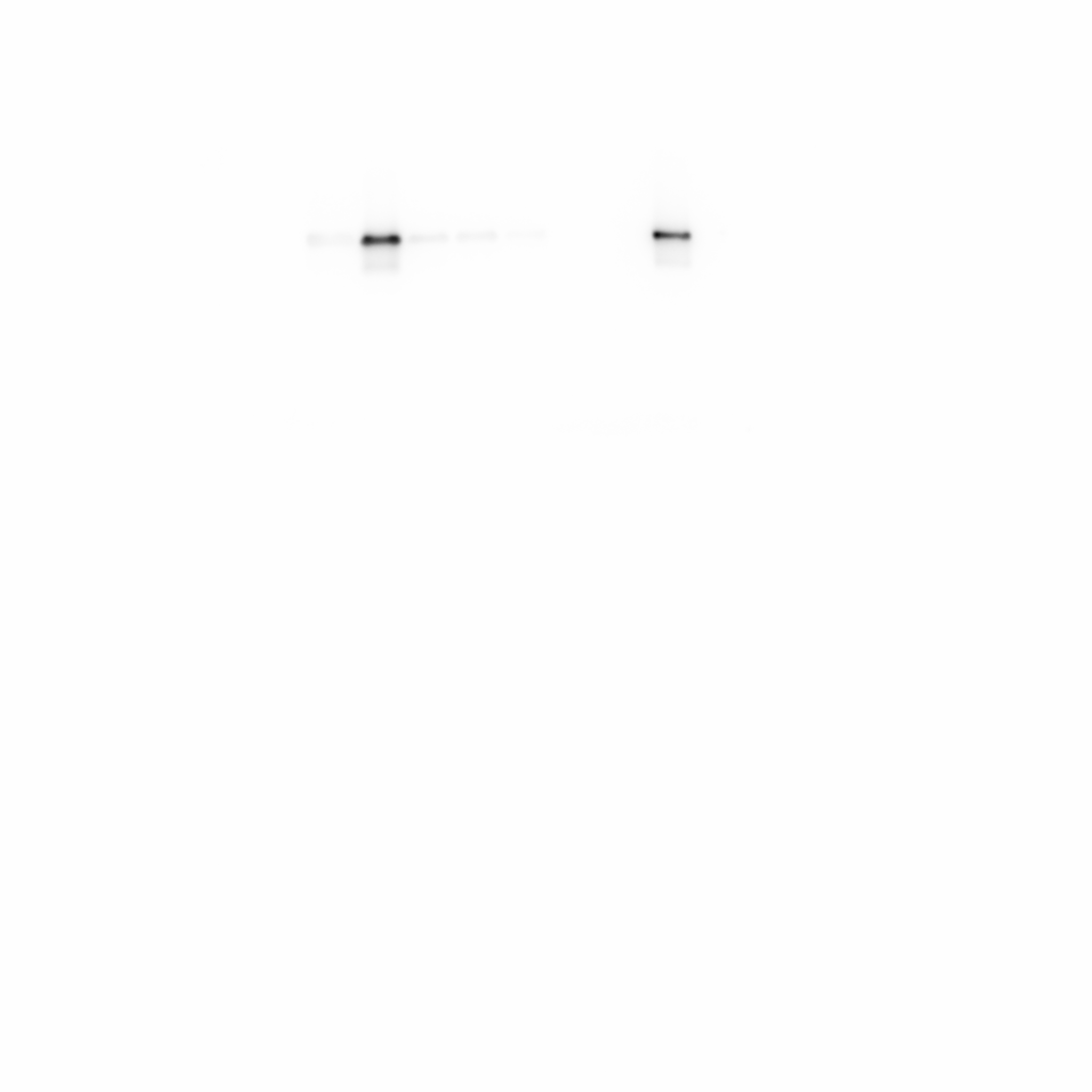

Supplement: Supplementary file 5 — Source data Fig. 4 [file 44318_2025_602_MOESM5_ESM.zip › Fig 4/C/H2Bub low exp.Tif]

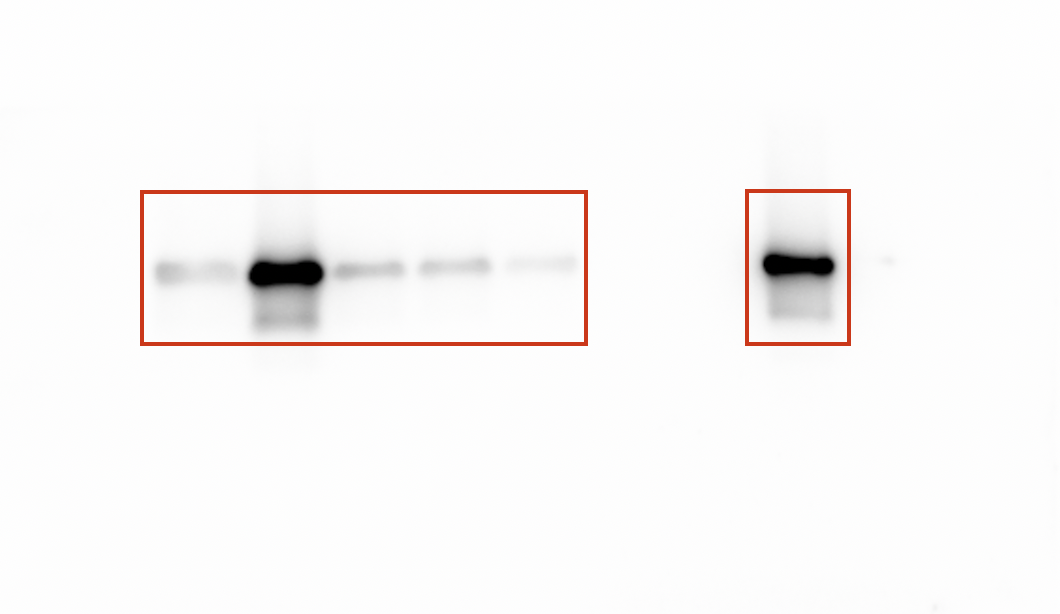

Supplement: Supplementary file 5 — Source data Fig. 4 [file 44318_2025_602_MOESM5_ESM.zip › Fig 4/C/WB H2Bub high exp Screenshot.png]

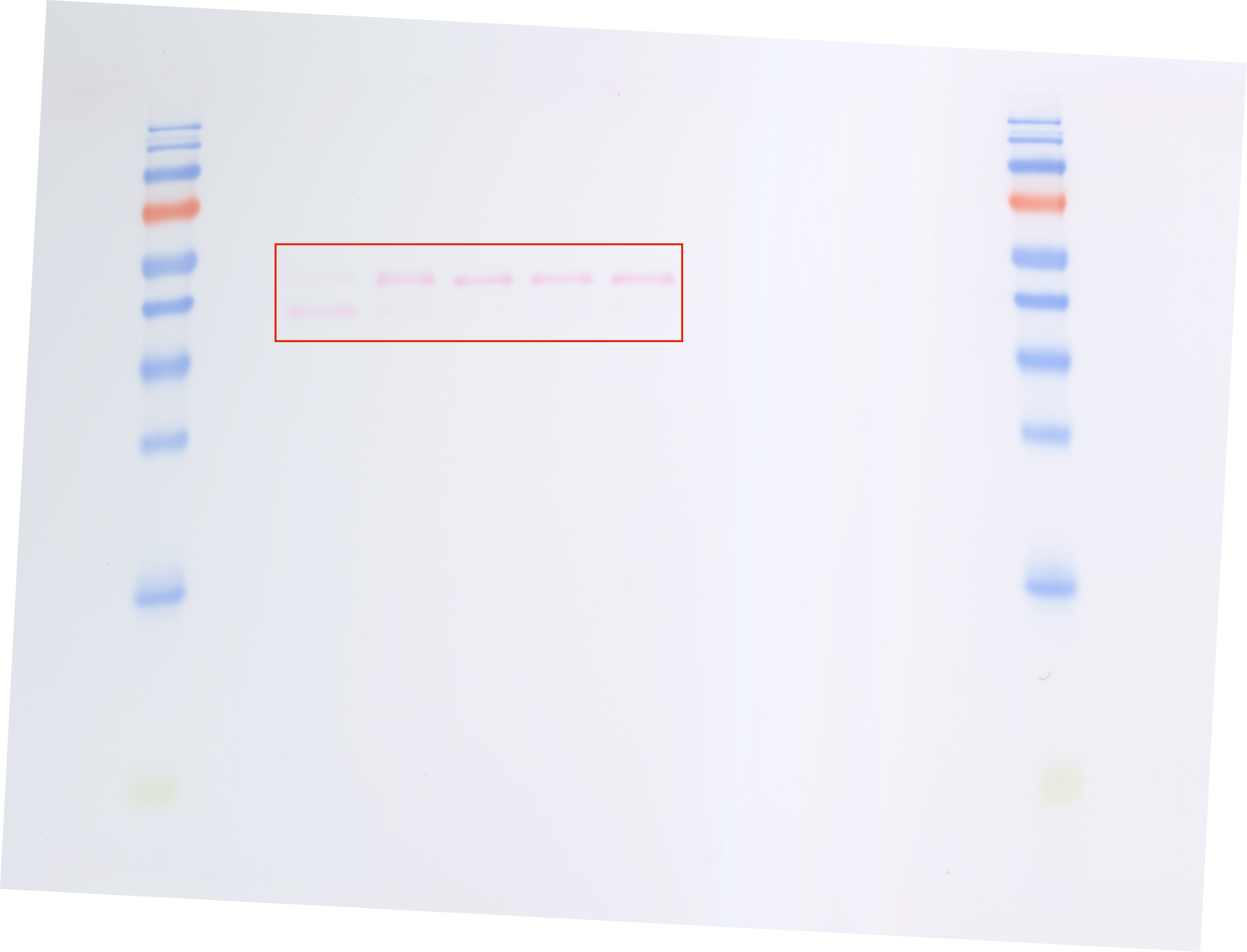

Supplement: Supplementary file 5 — Source data Fig. 4 [file 44318_2025_602_MOESM5_ESM.zip › Fig 4/C/WB PoncS.tif]

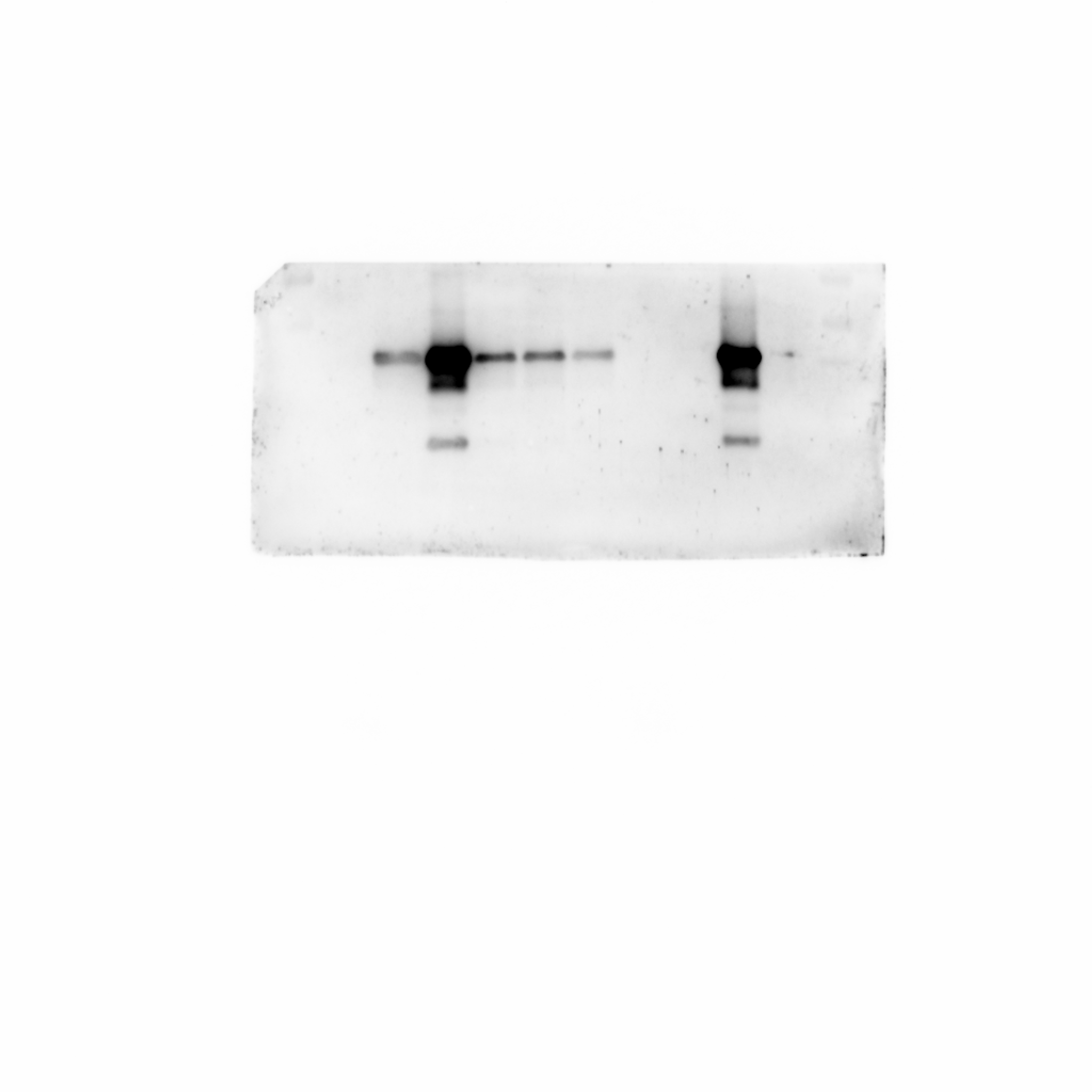

Supplement: Supplementary file 5 — Source data Fig. 4 [file 44318_2025_602_MOESM5_ESM.zip › Fig 4/C/WB H3.Tif]

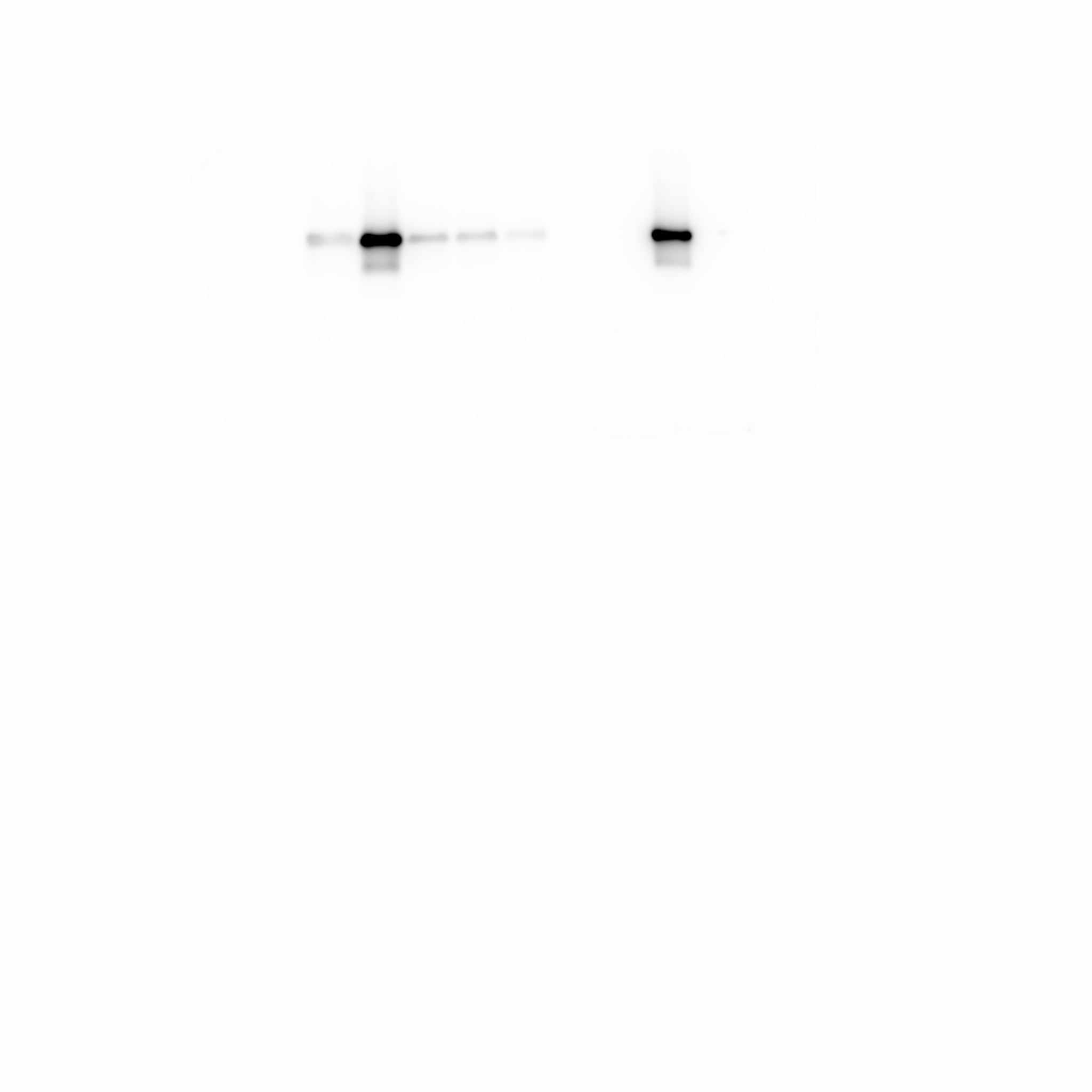

Supplement: Supplementary file 5 — Source data Fig. 4 [file 44318_2025_602_MOESM5_ESM.zip › Fig 4/C/WB H2Bub high exp.Tif]

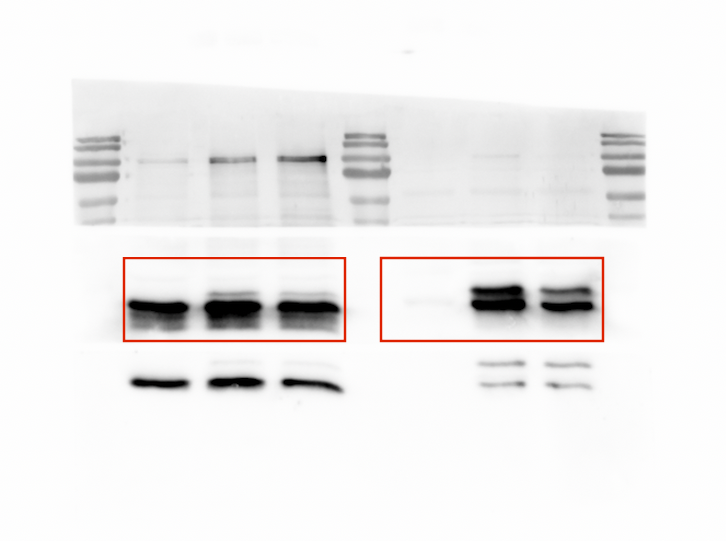

Supplement: Supplementary file 5 — Source data Fig. 4 [file 44318_2025_602_MOESM5_ESM.zip › Fig 4/D/H2Bub Screenshot.png]

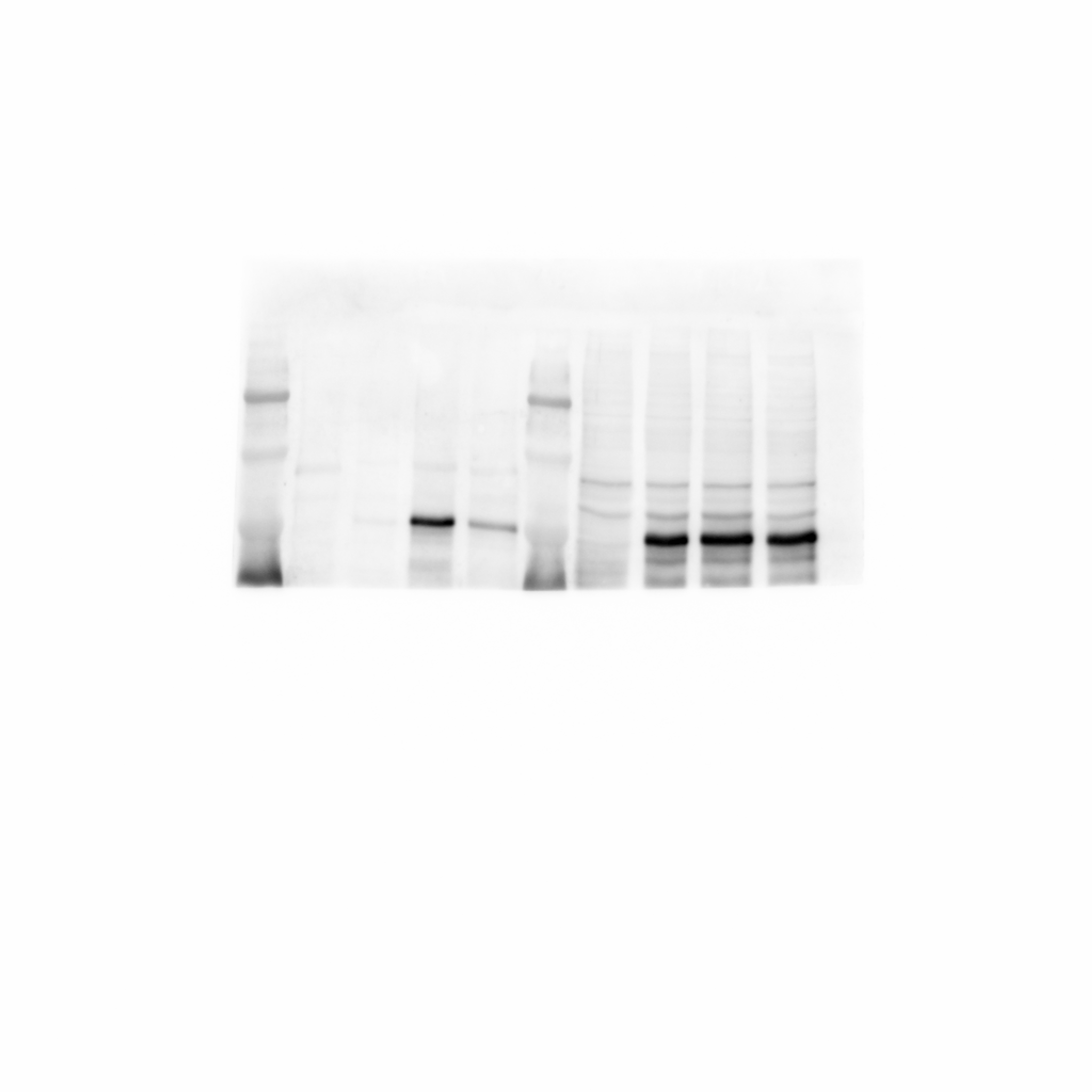

Supplement: Supplementary file 5 — Source data Fig. 4 [file 44318_2025_602_MOESM5_ESM.zip › Fig 4/D/RNF169.tif]

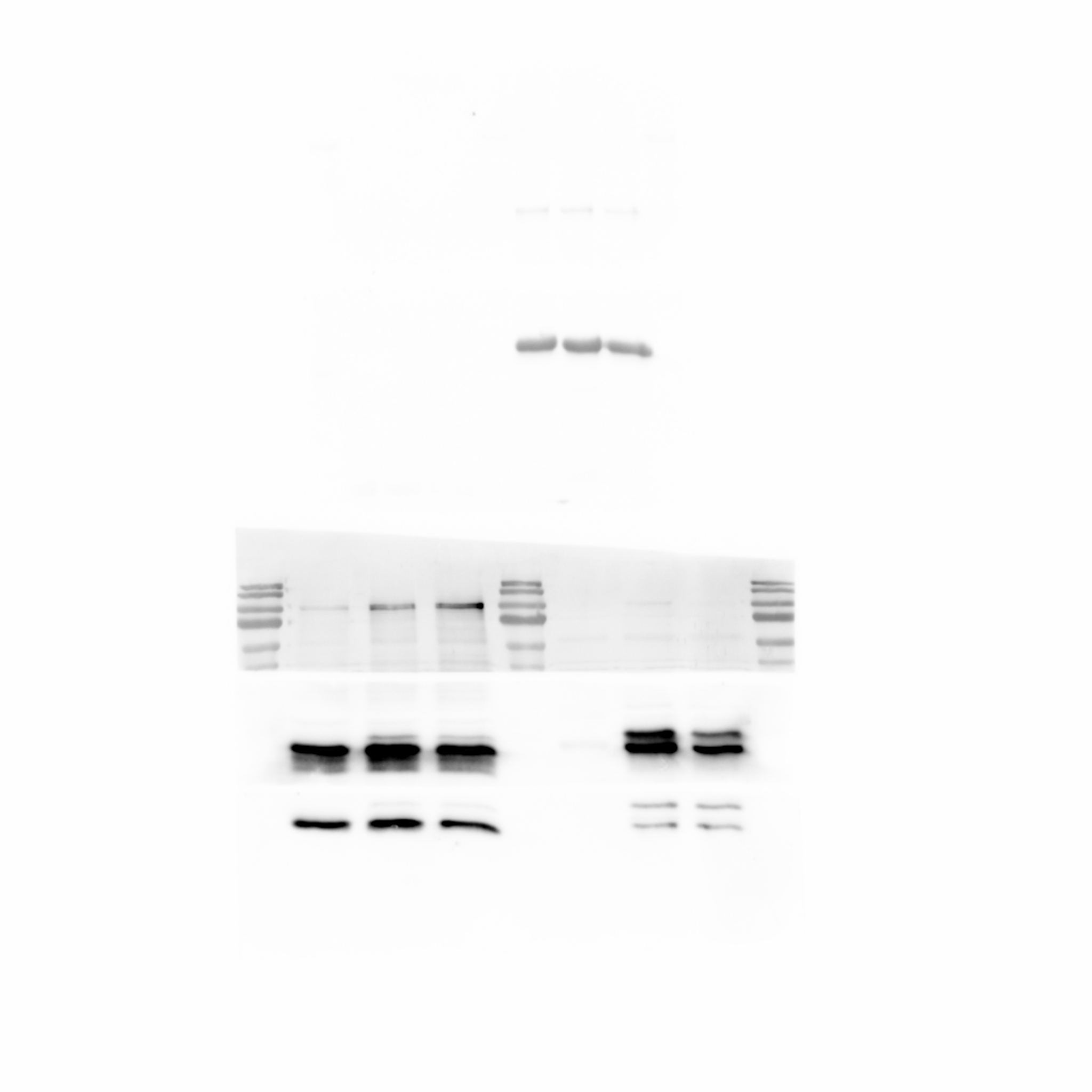

Supplement: Supplementary file 5 — Source data Fig. 4 [file 44318_2025_602_MOESM5_ESM.zip › Fig 4/D/H2Bub.tif]

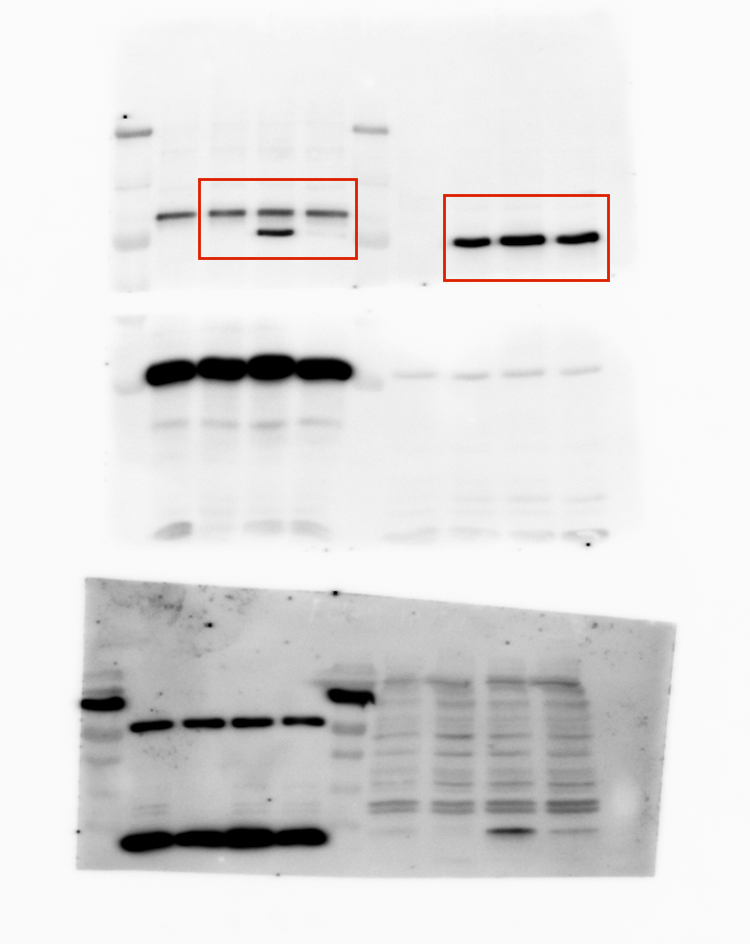

Supplement: Supplementary file 5 — Source data Fig. 4 [file 44318_2025_602_MOESM5_ESM.zip › Fig 4/D/Myc Screenshot.png]

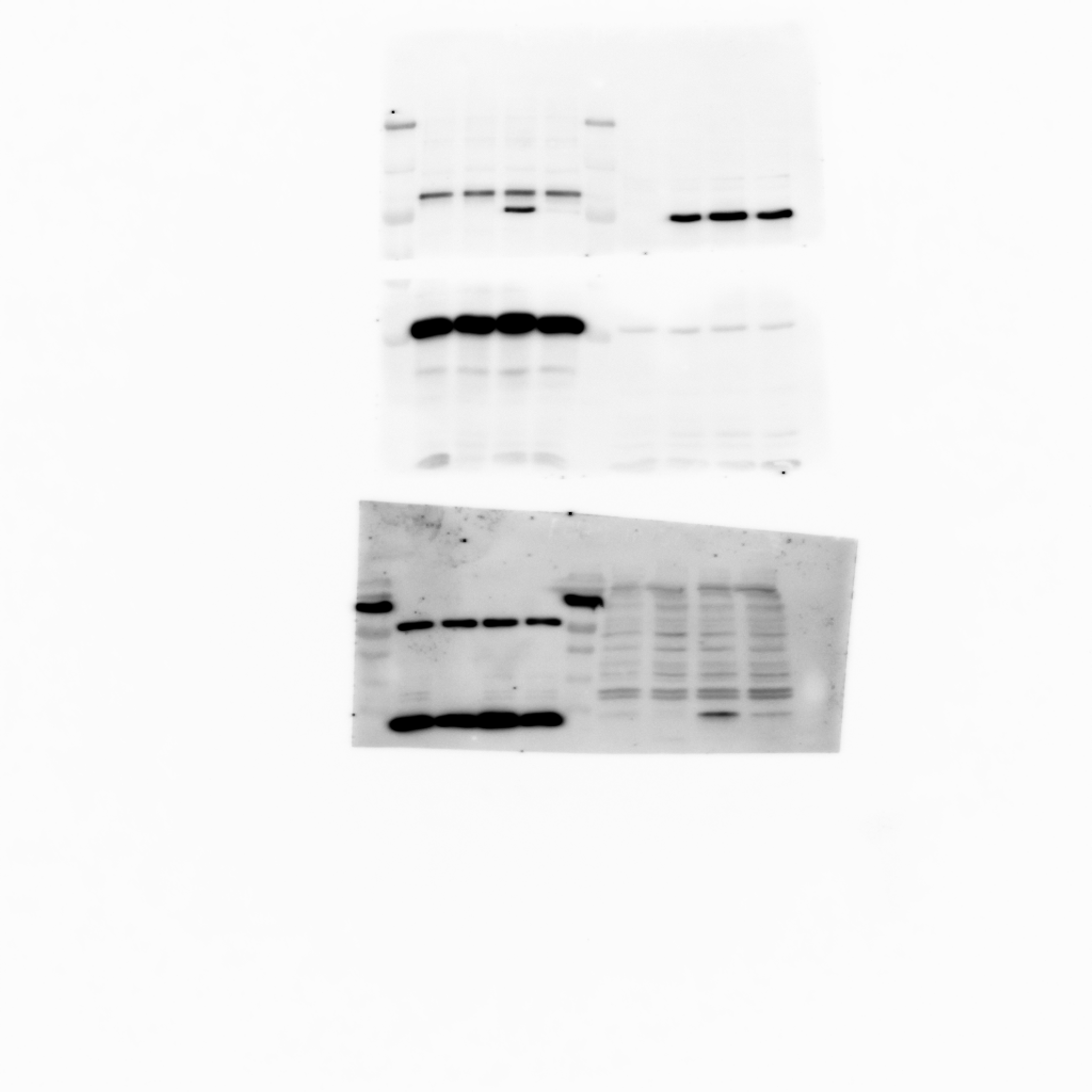

Supplement: Supplementary file 5 — Source data Fig. 4 [file 44318_2025_602_MOESM5_ESM.zip › Fig 4/D/Myc.tif]

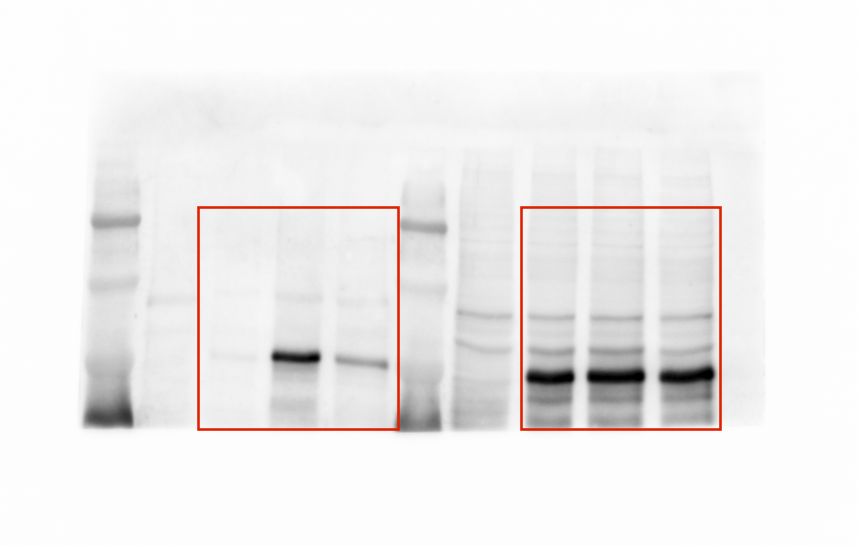

Supplement: Supplementary file 5 — Source data Fig. 4 [file 44318_2025_602_MOESM5_ESM.zip › Fig 4/D/RNF169 Screenshot.png]

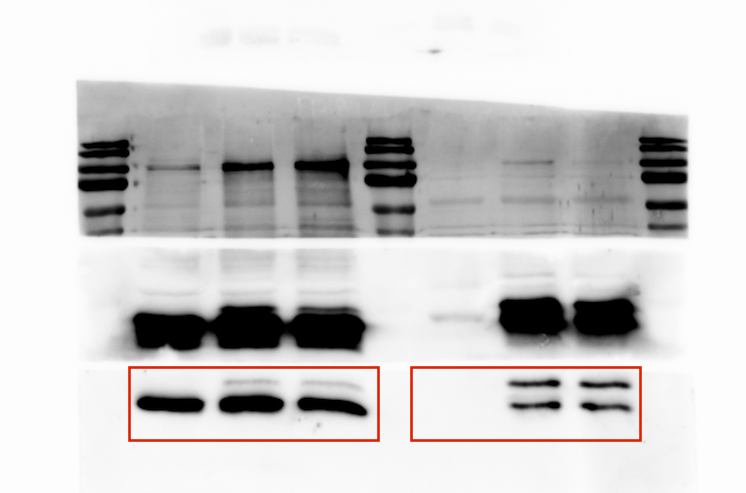

Supplement: Supplementary file 5 — Source data Fig. 4 [file 44318_2025_602_MOESM5_ESM.zip › Fig 4/D/H2B Screenshot.png]

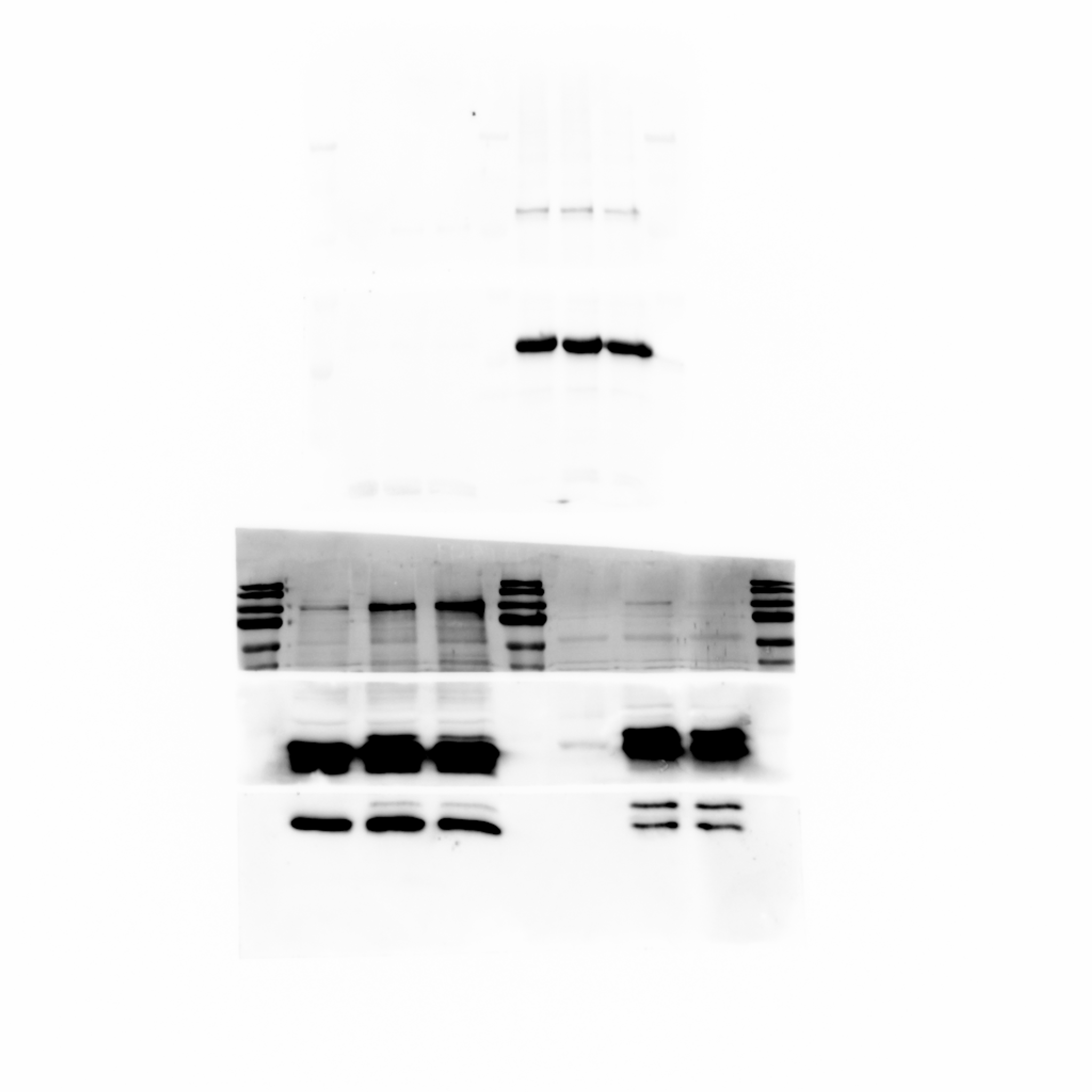

Supplement: Supplementary file 5 — Source data Fig. 4 [file 44318_2025_602_MOESM5_ESM.zip › Fig 4/D/H2B.tif]

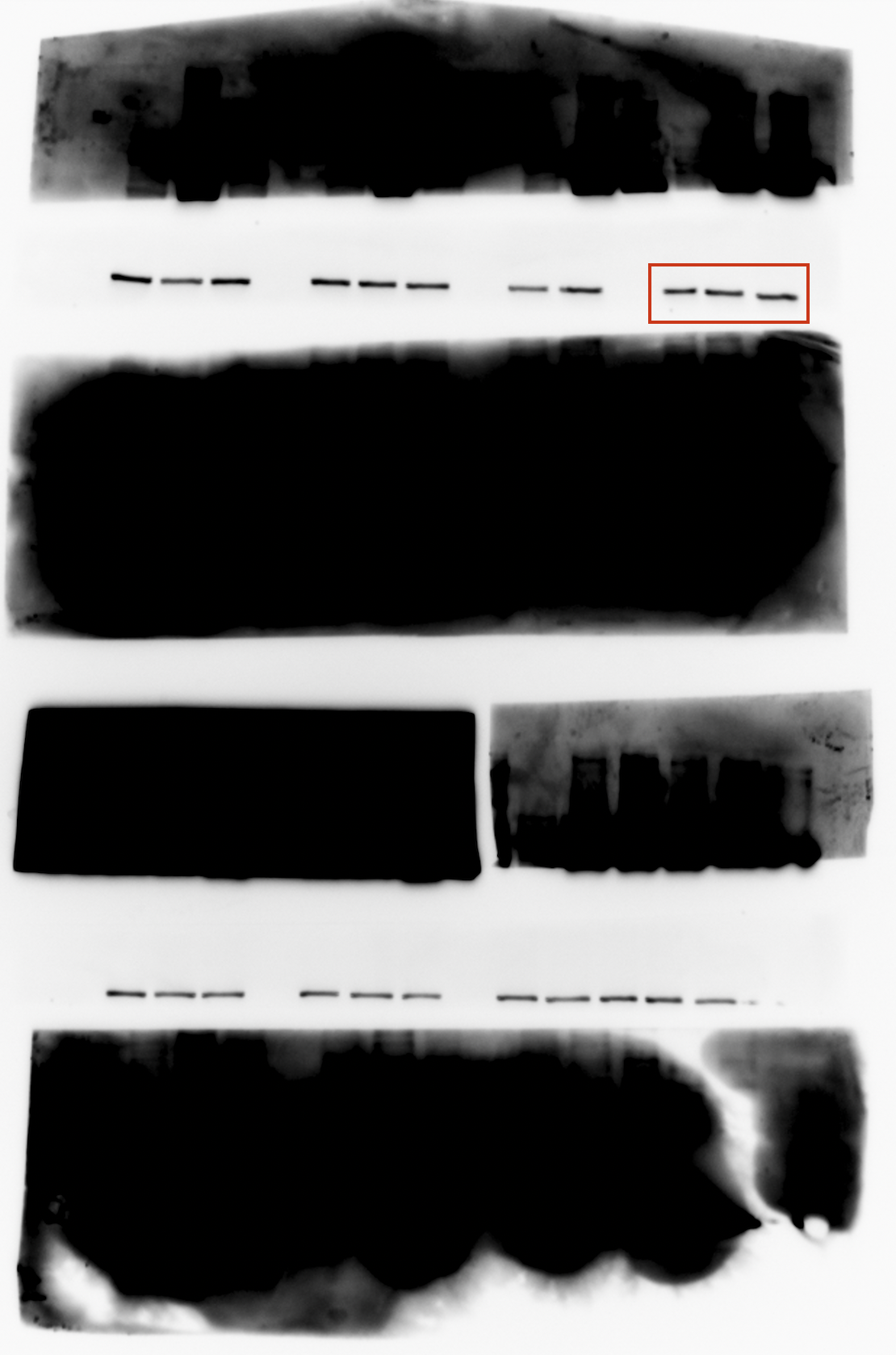

Supplement: Supplementary file 5 — Source data Fig. 4 [file 44318_2025_602_MOESM5_ESM.zip › Fig 4/E/b-actin Screenshot.png]

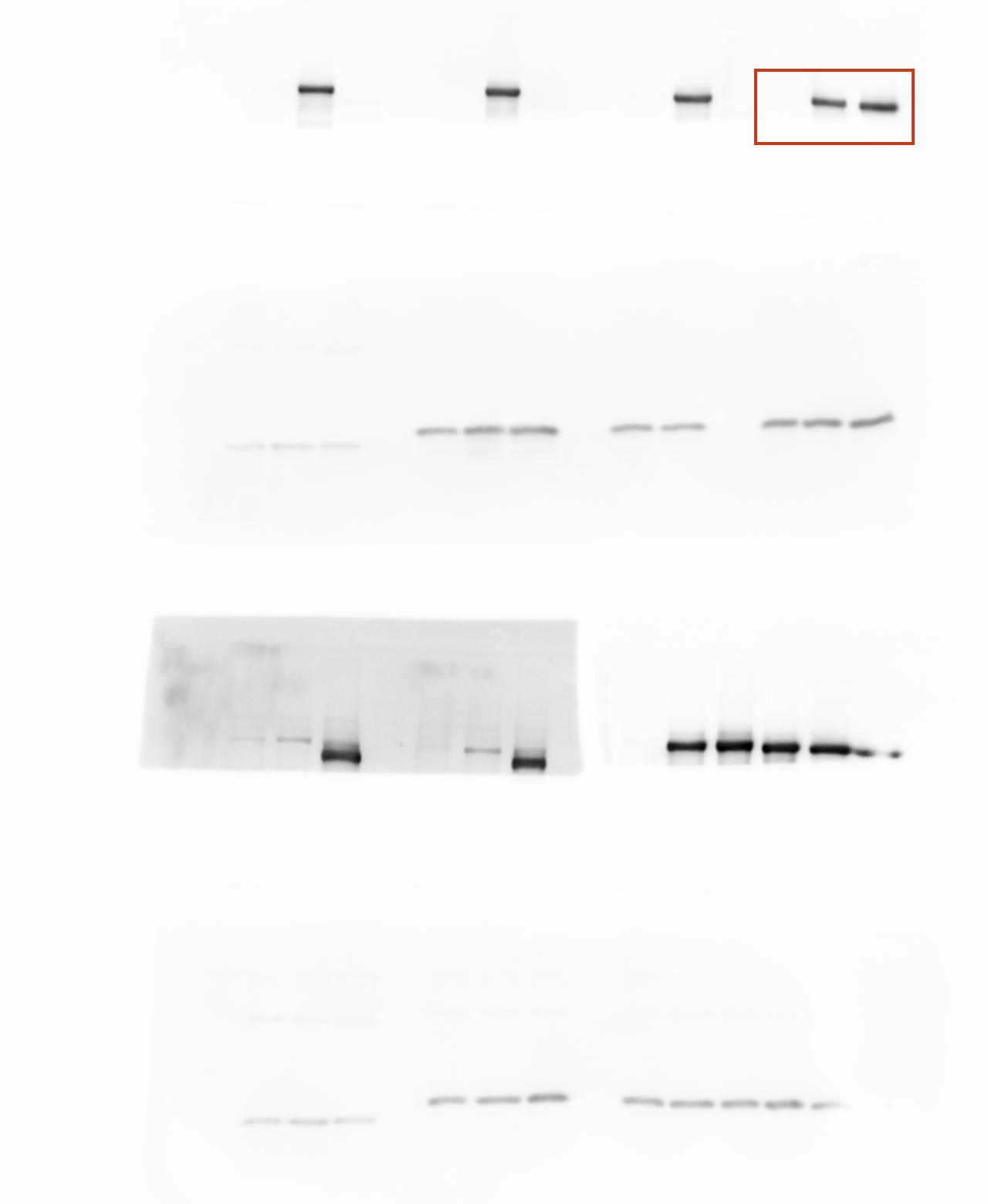

Supplement: Supplementary file 5 — Source data Fig. 4 [file 44318_2025_602_MOESM5_ESM.zip › Fig 4/E/RNF169 Screenshot.png]

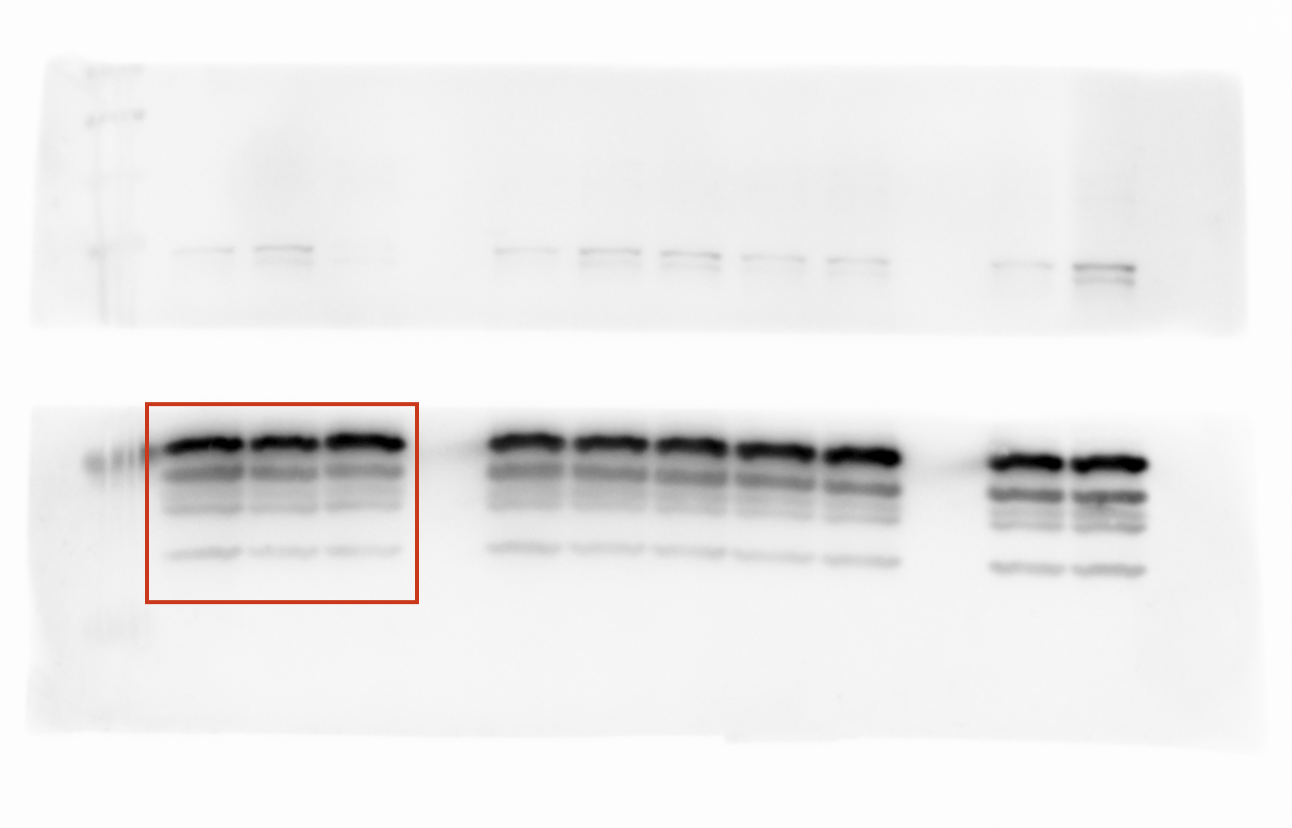

Supplement: Supplementary file 5 — Source data Fig. 4 [file 44318_2025_602_MOESM5_ESM.zip › Fig 4/E/FLAG_15kDa Screenshot.png]

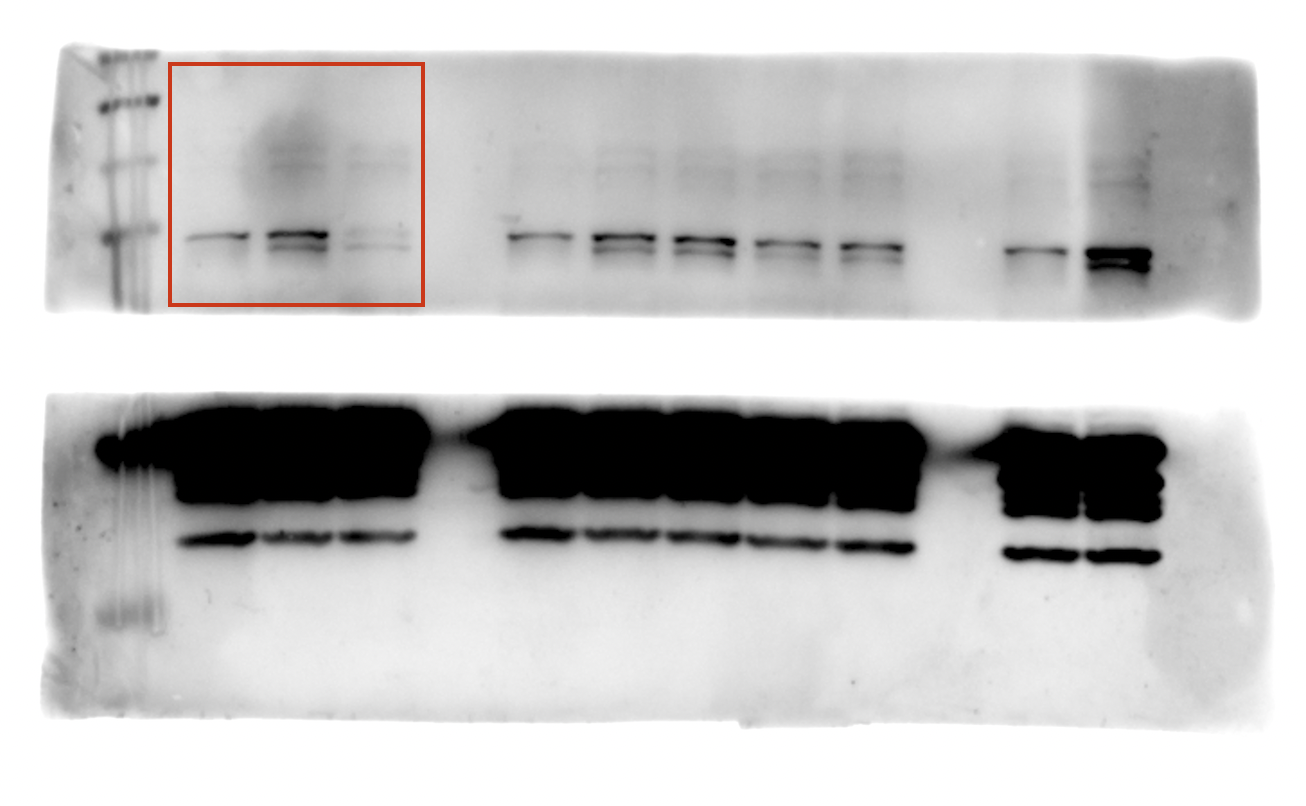

Supplement: Supplementary file 5 — Source data Fig. 4 [file 44318_2025_602_MOESM5_ESM.zip › Fig 4/E/FLAG_25kDa Screenshot.png]

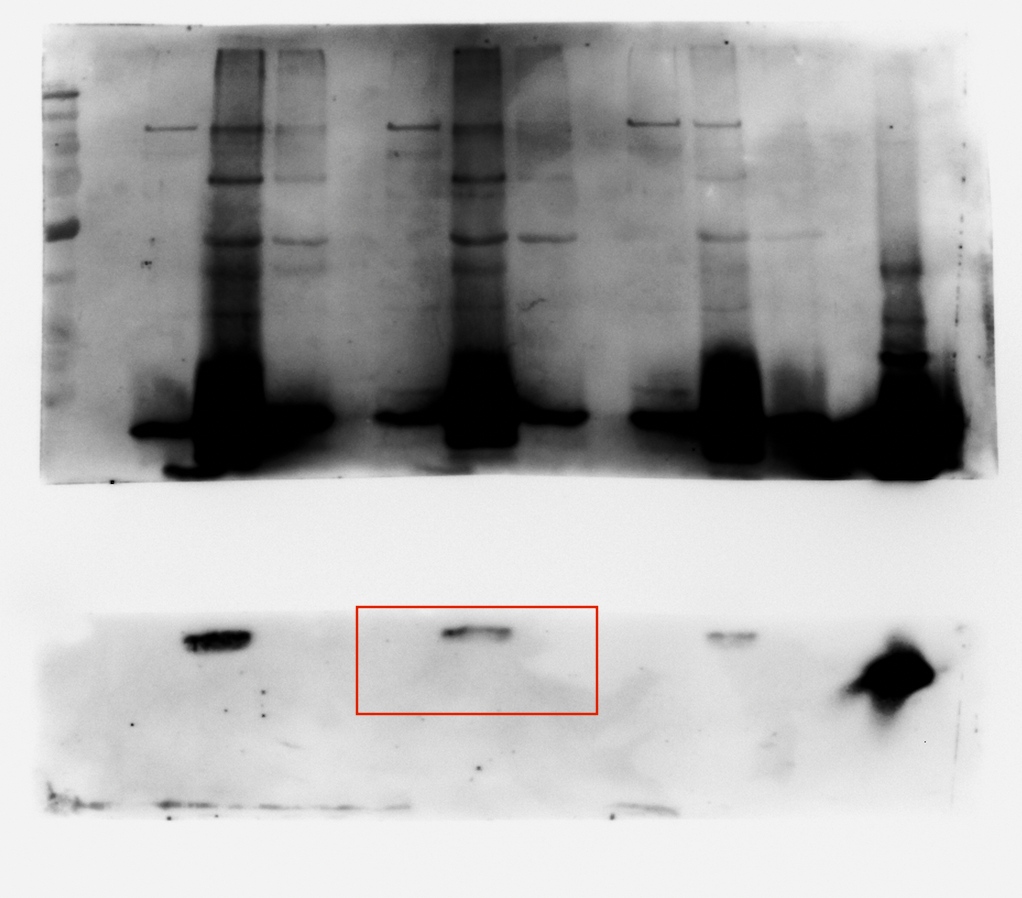

Supplement: Supplementary file 5 — Source data Fig. 4 [file 44318_2025_602_MOESM5_ESM.zip › Fig 4/B/H3 pulldown Screenshot.png]

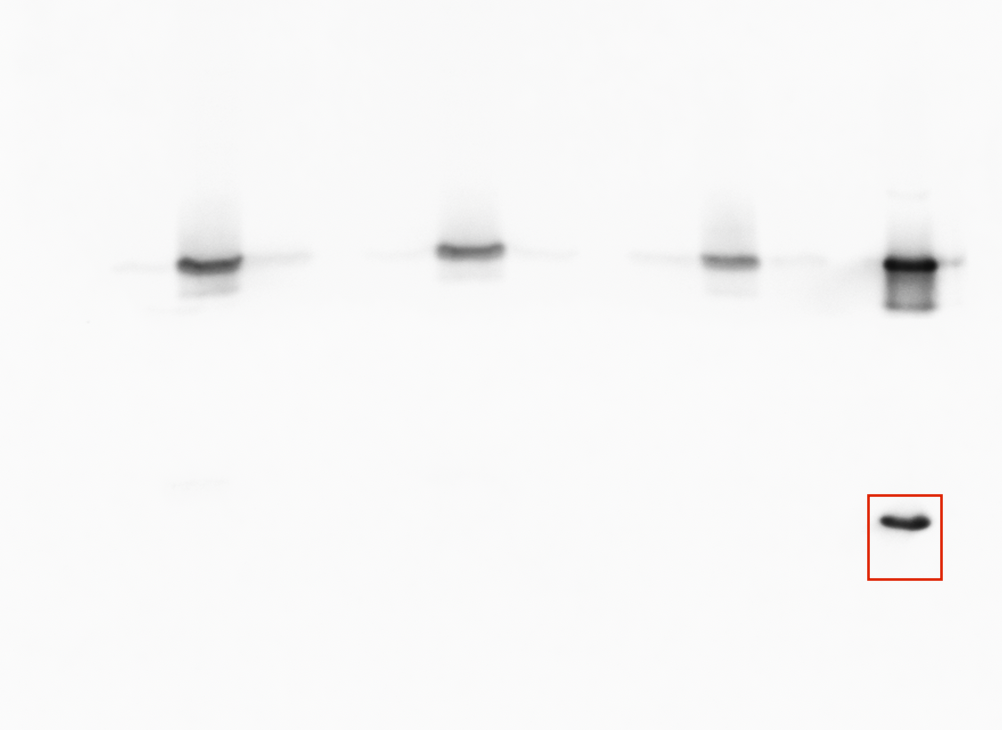

Supplement: Supplementary file 5 — Source data Fig. 4 [file 44318_2025_602_MOESM5_ESM.zip › Fig 4/B/H3 INPUT Screenshot.png]

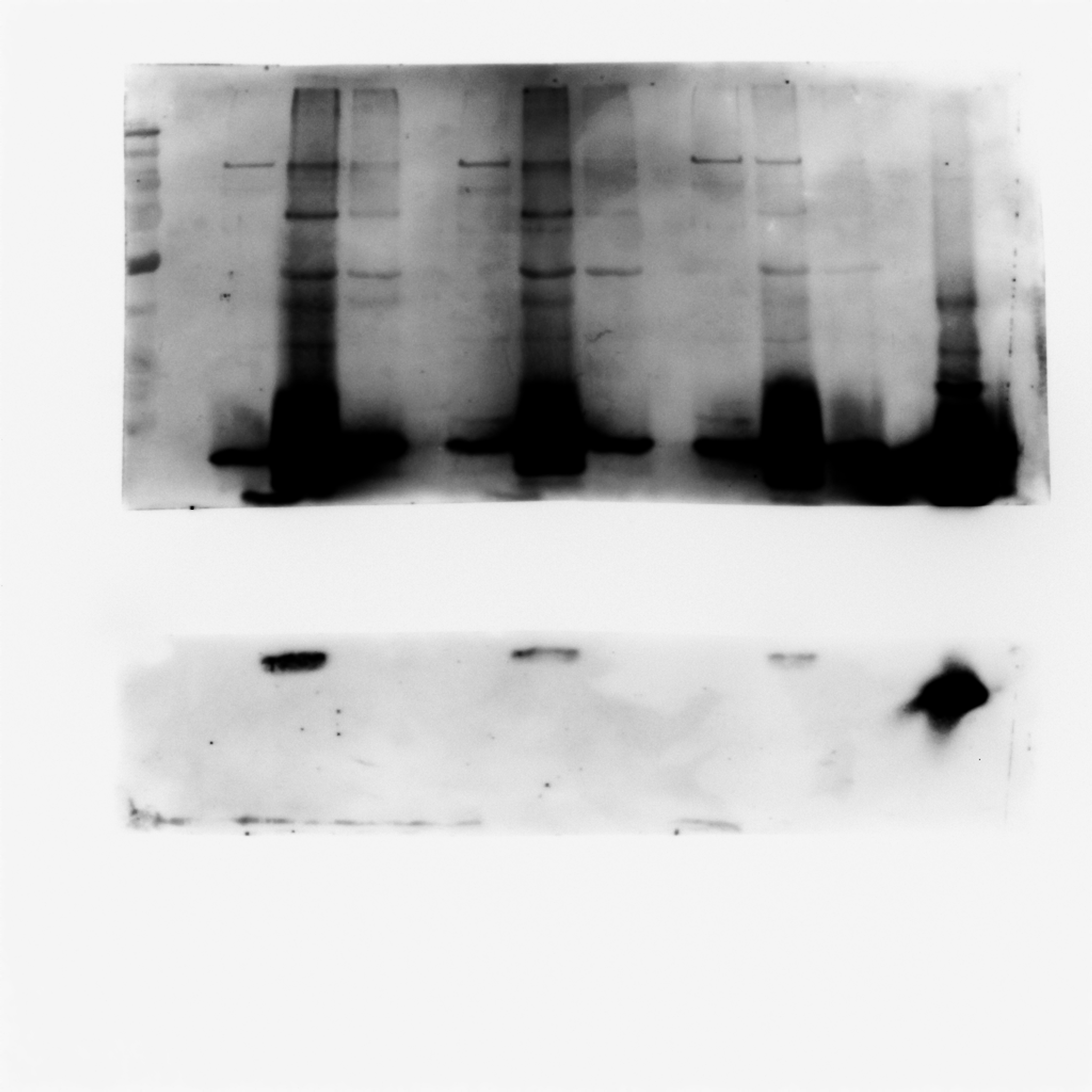

Supplement: Supplementary file 5 — Source data Fig. 4 [file 44318_2025_602_MOESM5_ESM.zip › Fig 4/B/H3 pulldown.Tif]
