# Supplementary figures and images for: H2BK120ub and its reader RNF169 sequentially regulate replication fork remodeling and stability (part 2 of 2)
Source: EMBO J. 2025 Oct 27;44(22):6598–625. doi: 10.1038/s44318-025-00602-1 (PMC12623888; doi:10.1038/s44318-025-00602-1)

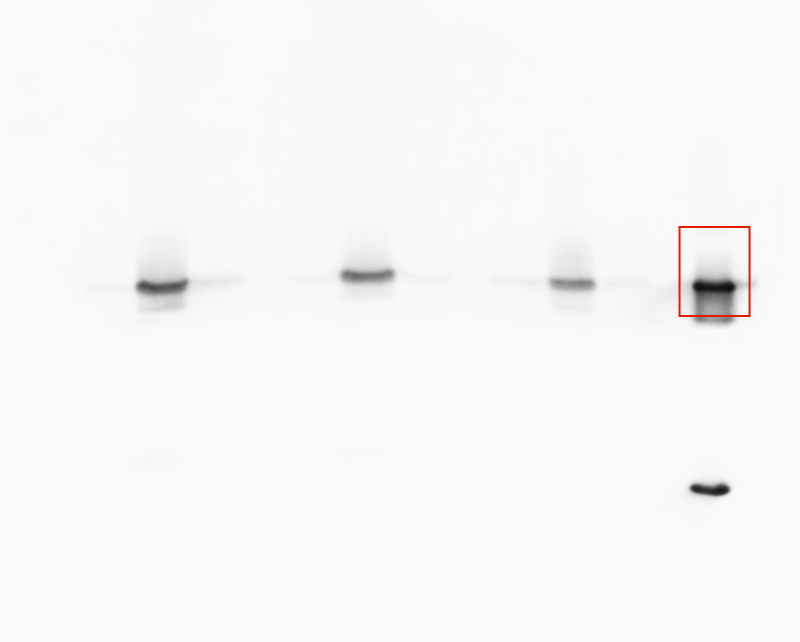

Supplement: Supplementary file 5 — Source data Fig. 4 [file 44318_2025_602_MOESM5_ESM.zip › Fig 4/B/H2BK120ub INPUT Screenshot.png]

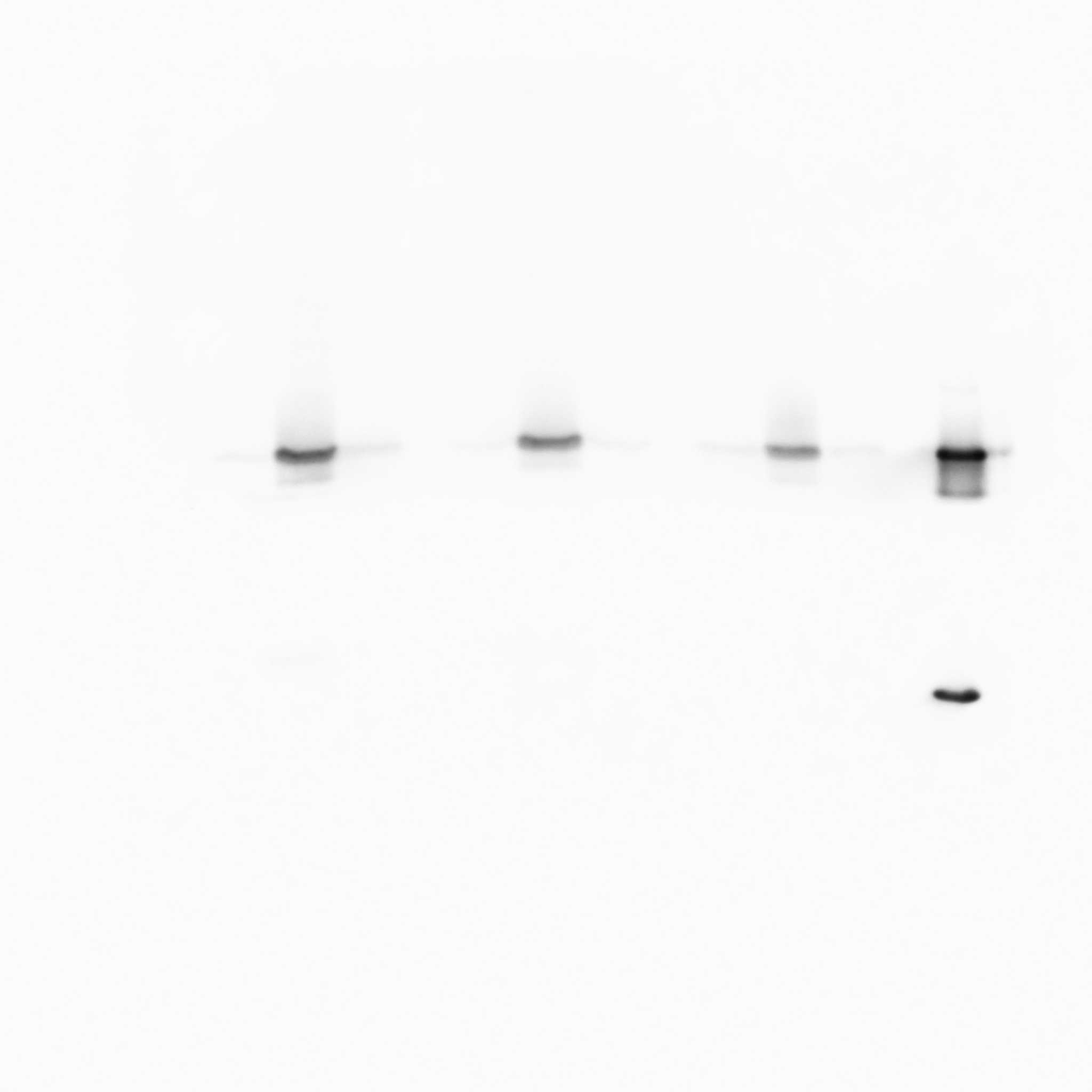

Supplement: Supplementary file 5 — Source data Fig. 4 [file 44318_2025_602_MOESM5_ESM.zip › Fig 4/B/H2BK120ub INPUT.Tif]

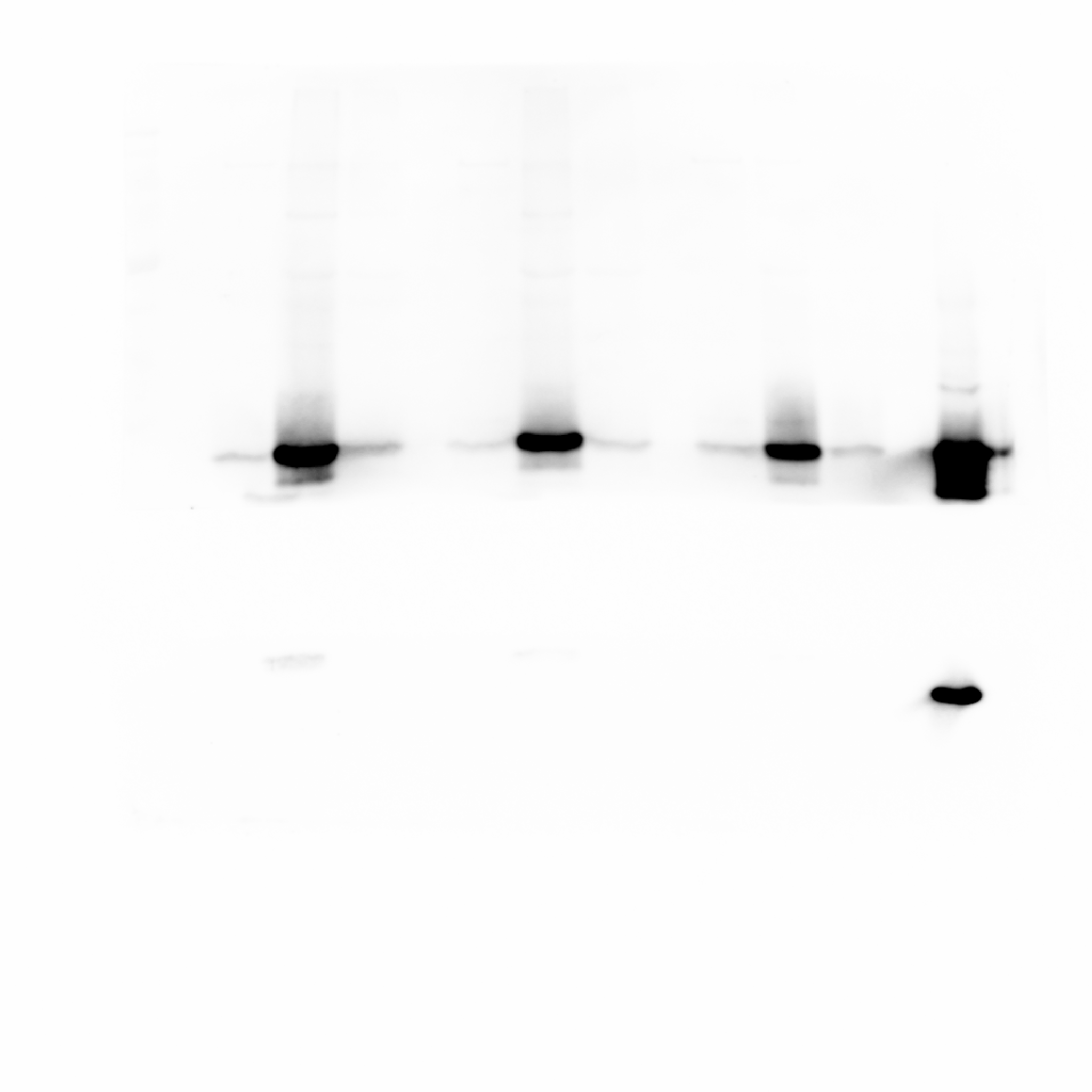

Supplement: Supplementary file 5 — Source data Fig. 4 [file 44318_2025_602_MOESM5_ESM.zip › Fig 4/B/H2BK120ub pulldown.Tif]

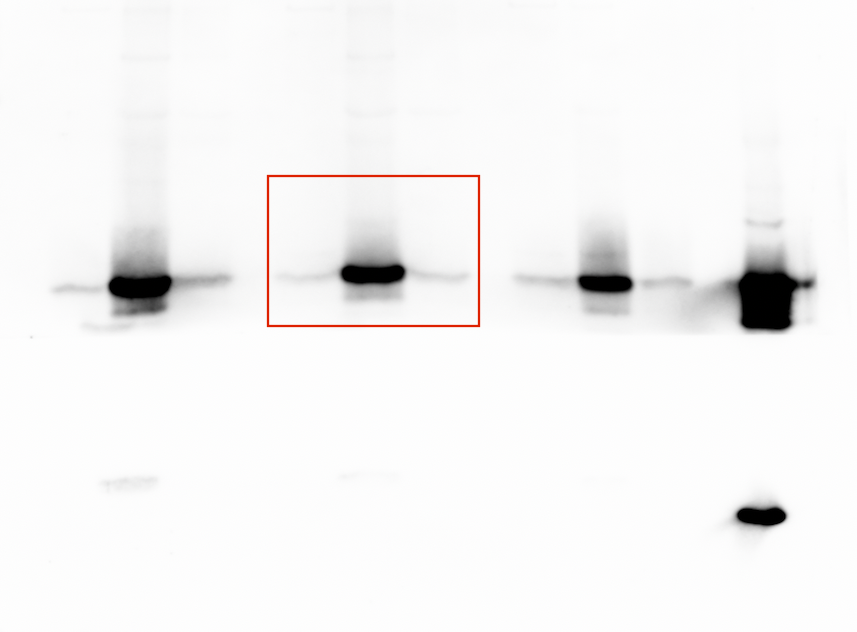

Supplement: Supplementary file 5 — Source data Fig. 4 [file 44318_2025_602_MOESM5_ESM.zip › Fig 4/B/H2BK120ub pulldown Screenshot.png]

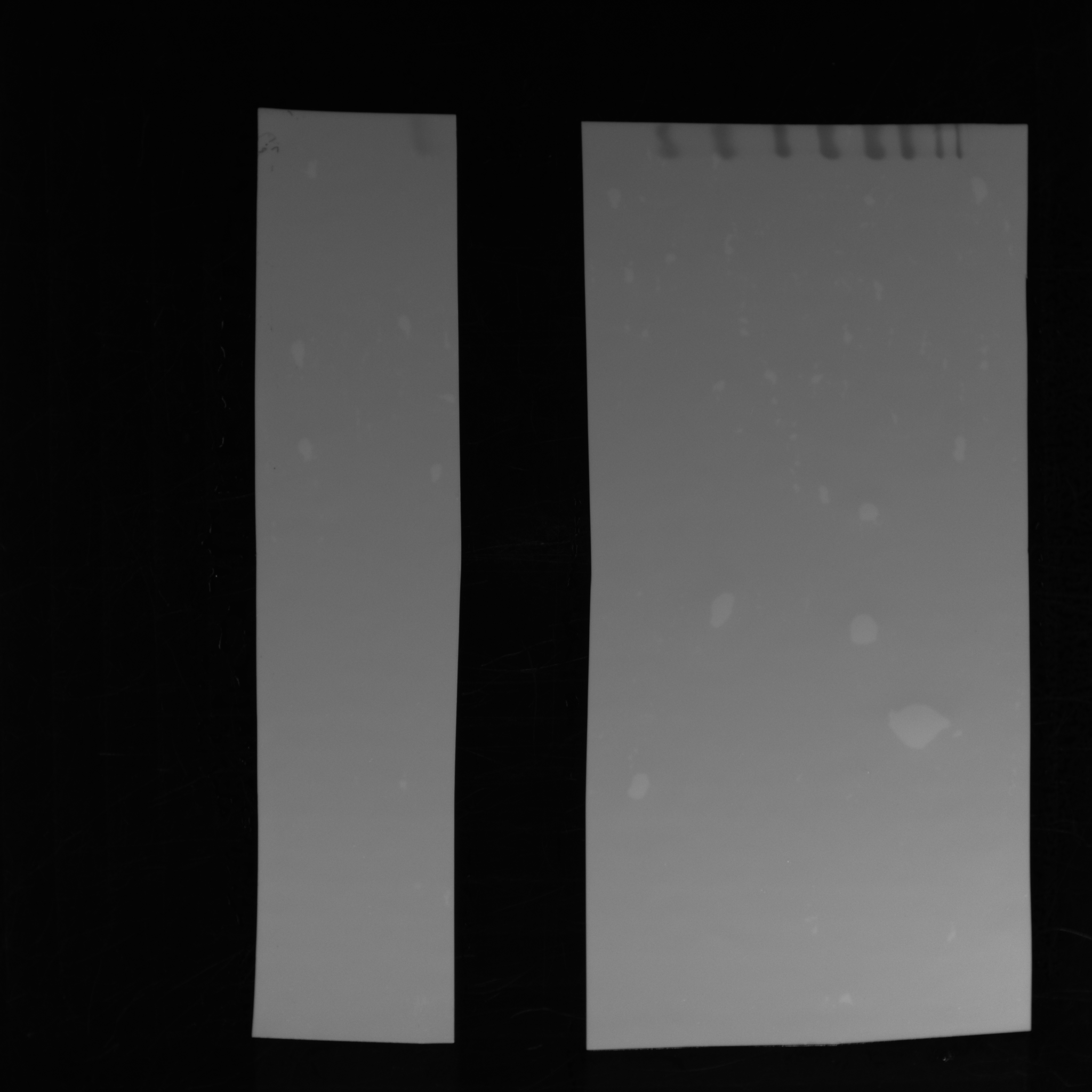

Supplement: Supplementary file 5 — Source data Fig. 4 [file 44318_2025_602_MOESM5_ESM.zip › Fig 4/B/230524/ladder.Tif]

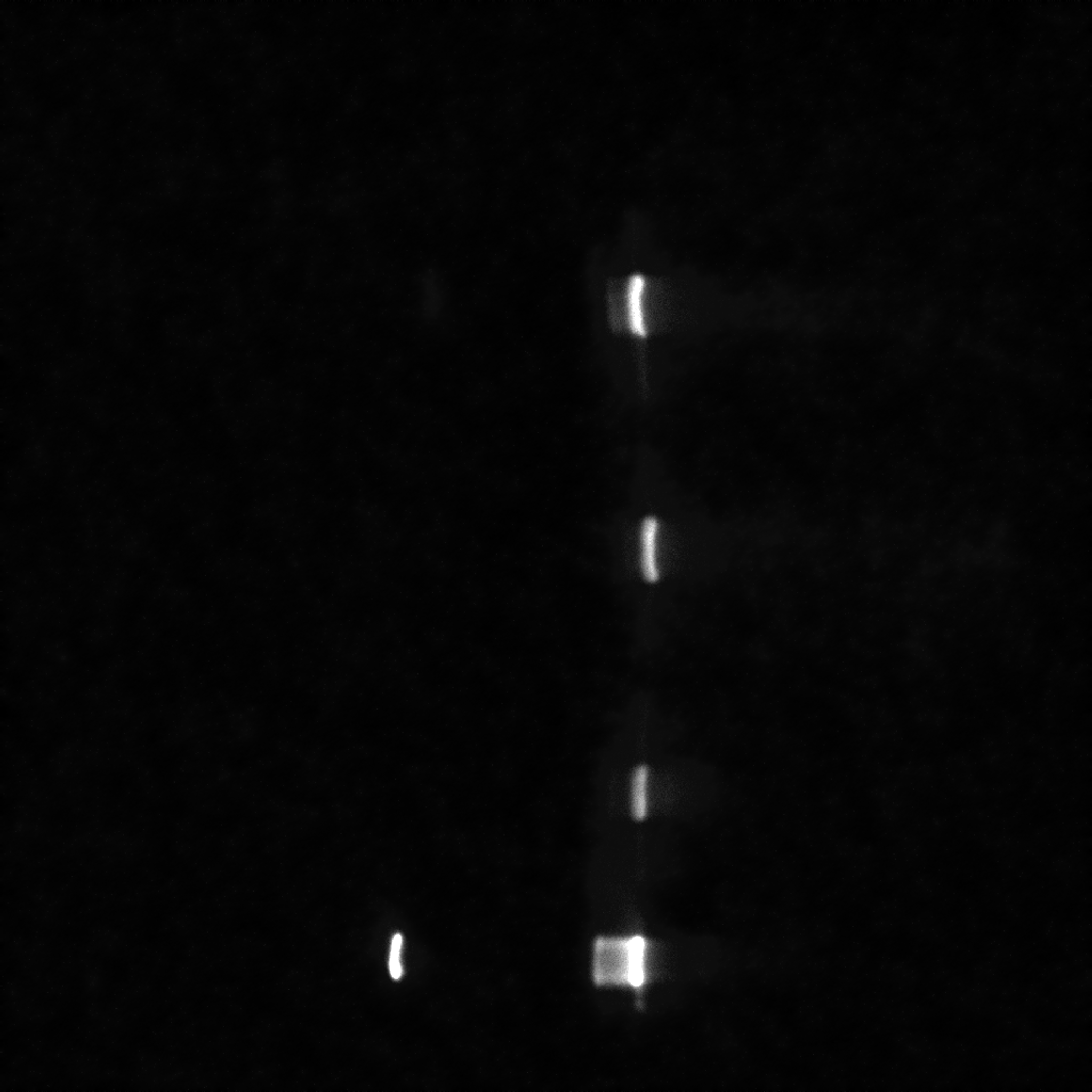

Supplement: Supplementary file 5 — Source data Fig. 4 [file 44318_2025_602_MOESM5_ESM.zip › Fig 4/B/230524/0.2s.Tif]

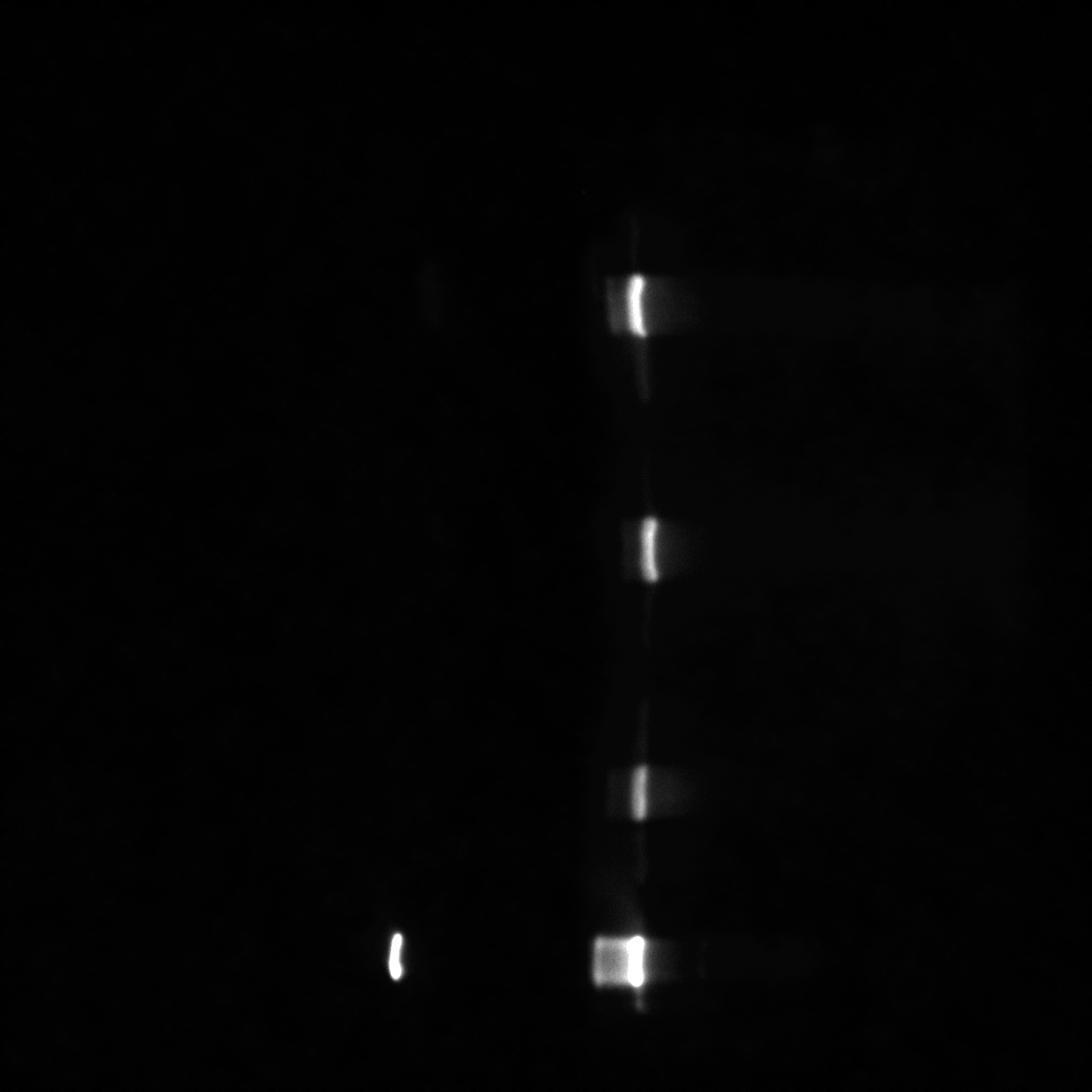

Supplement: Supplementary file 5 — Source data Fig. 4 [file 44318_2025_602_MOESM5_ESM.zip › Fig 4/B/230524/0.5s.Tif]

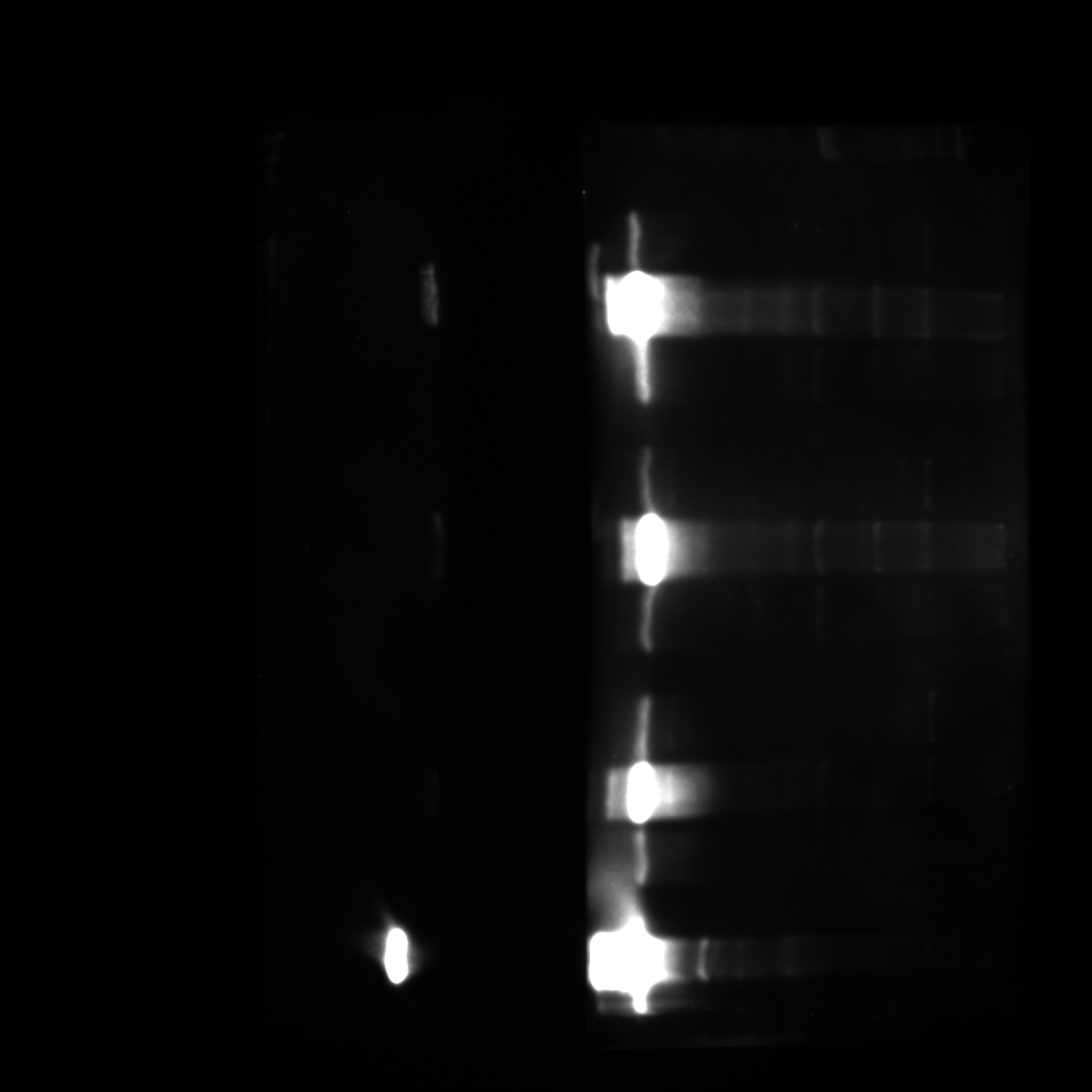

Supplement: Supplementary file 5 — Source data Fig. 4 [file 44318_2025_602_MOESM5_ESM.zip › Fig 4/B/230524/45s.Tif]

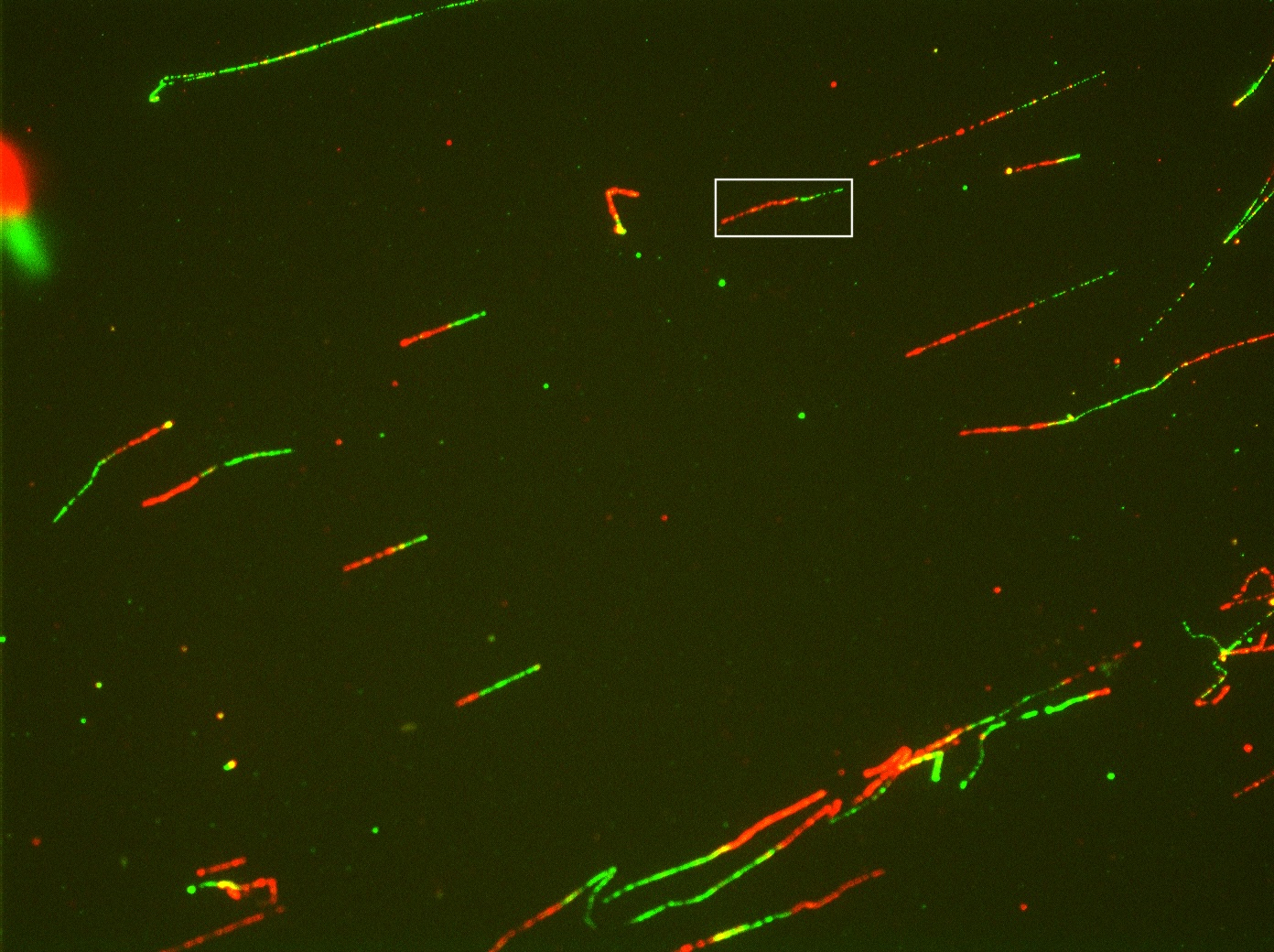

Supplement: Supplementary file 6 — Source data Fig. 5 [file 44318_2025_602_MOESM6_ESM.zip › Fig 5/I/siRNF169_EV.jpg]

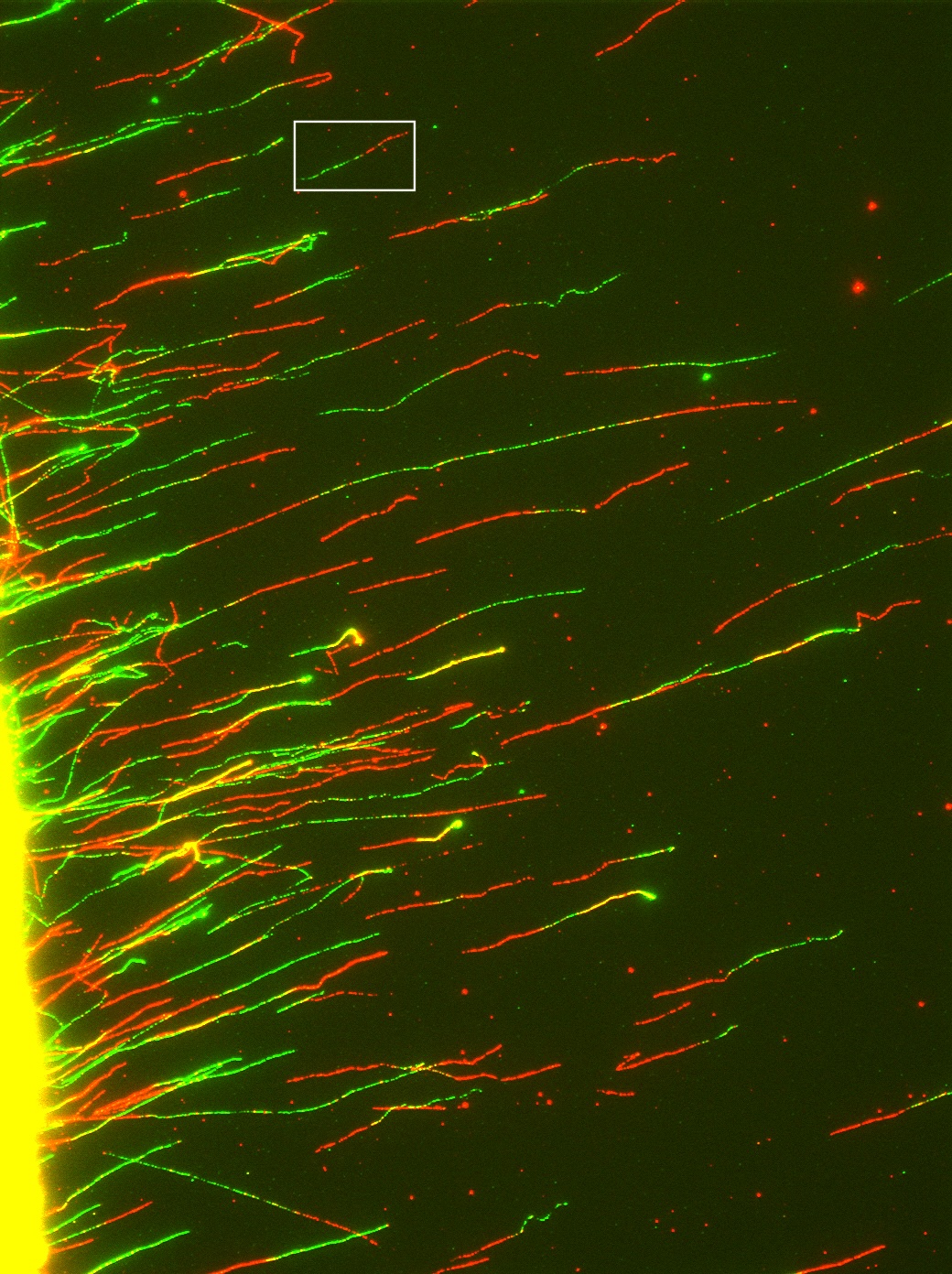

Supplement: Supplementary file 6 — Source data Fig. 5 [file 44318_2025_602_MOESM6_ESM.zip › Fig 5/I/siRNF169_WT.jpg]

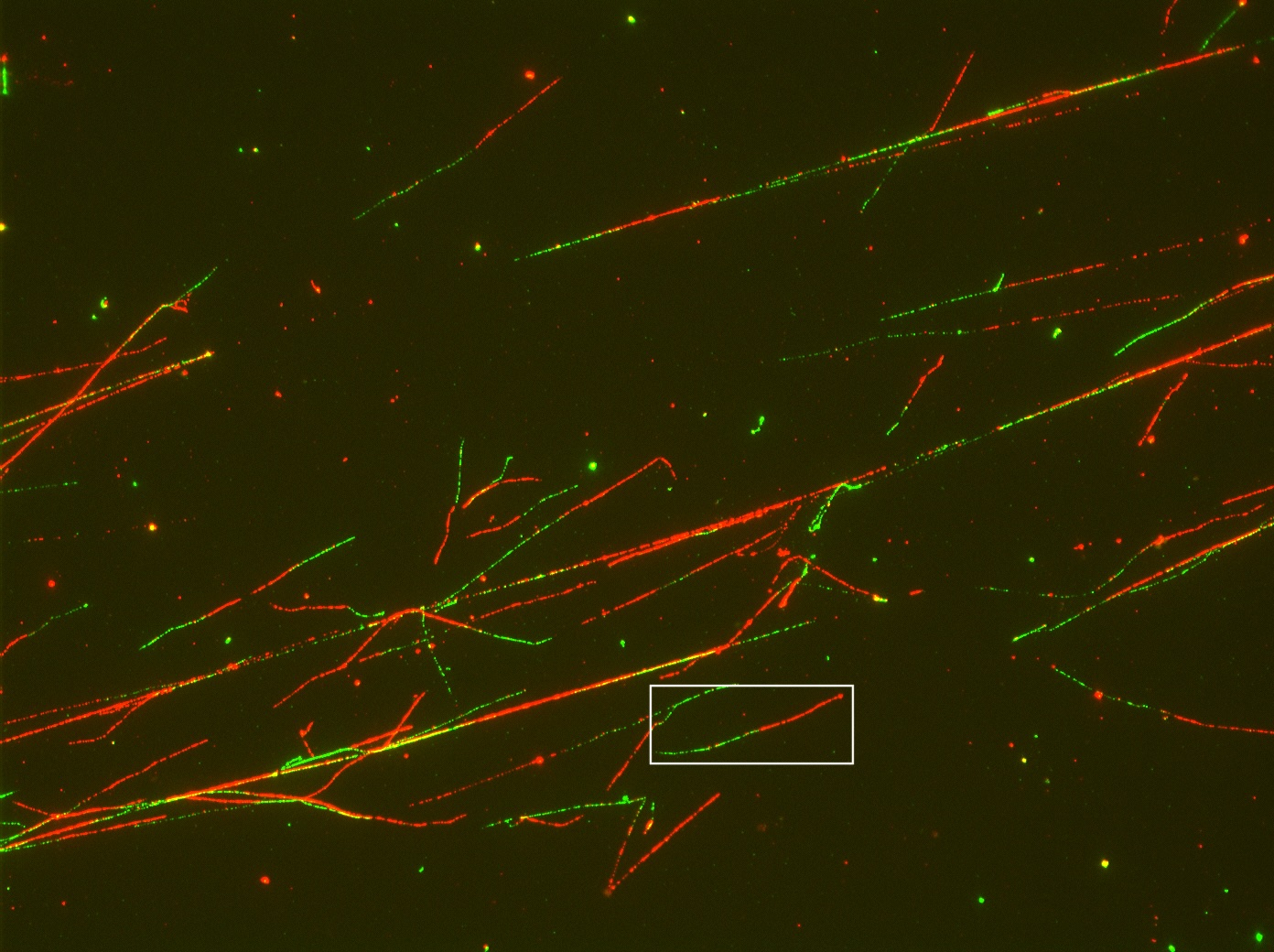

Supplement: Supplementary file 6 — Source data Fig. 5 [file 44318_2025_602_MOESM6_ESM.zip › Fig 5/I/siLuc_EV.jpg]

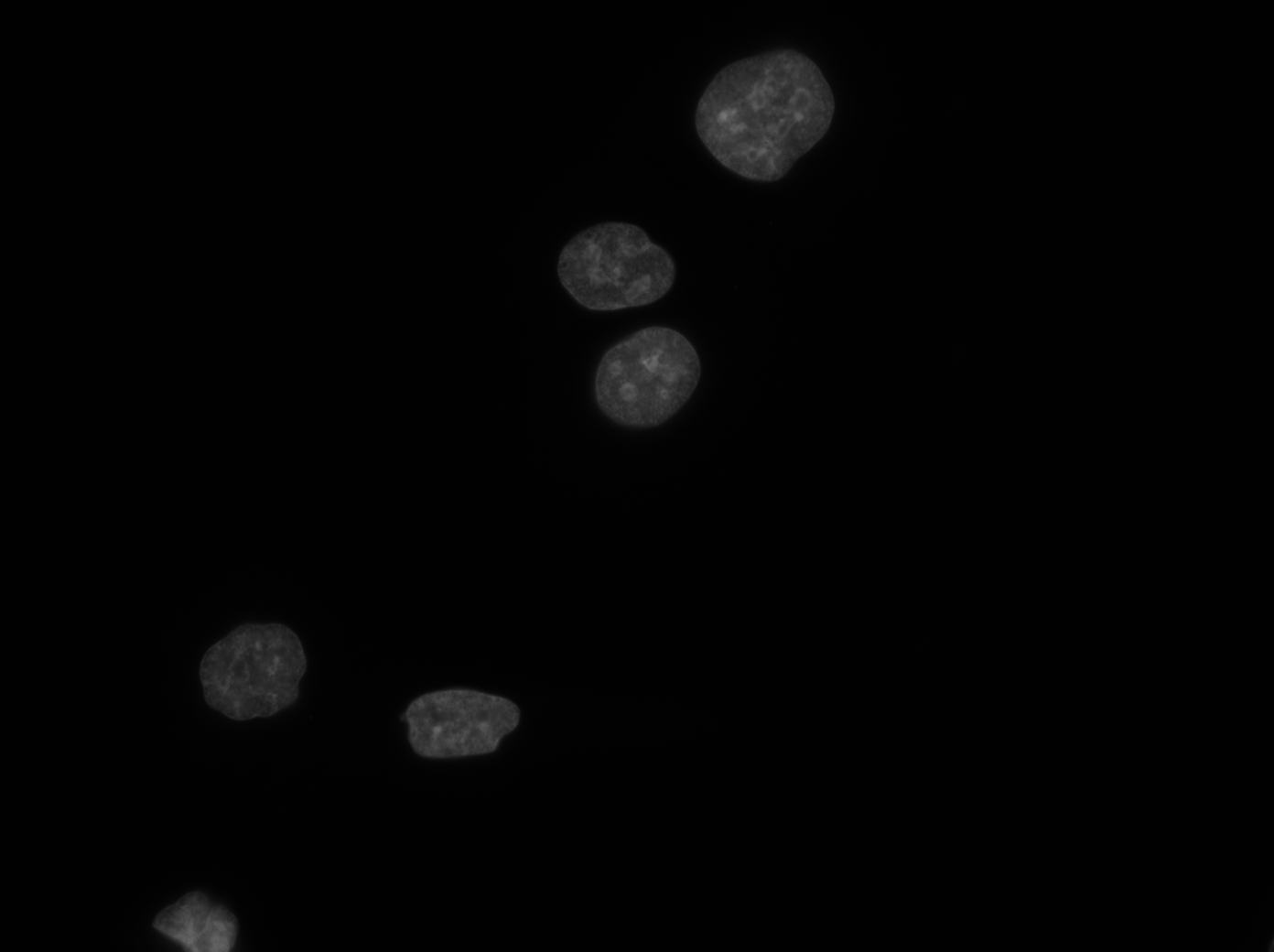

Supplement: Supplementary file 6 — Source data Fig. 5 [file 44318_2025_602_MOESM6_ESM.zip › Fig 5/A/siRNF20 CPT DAPI.tif]

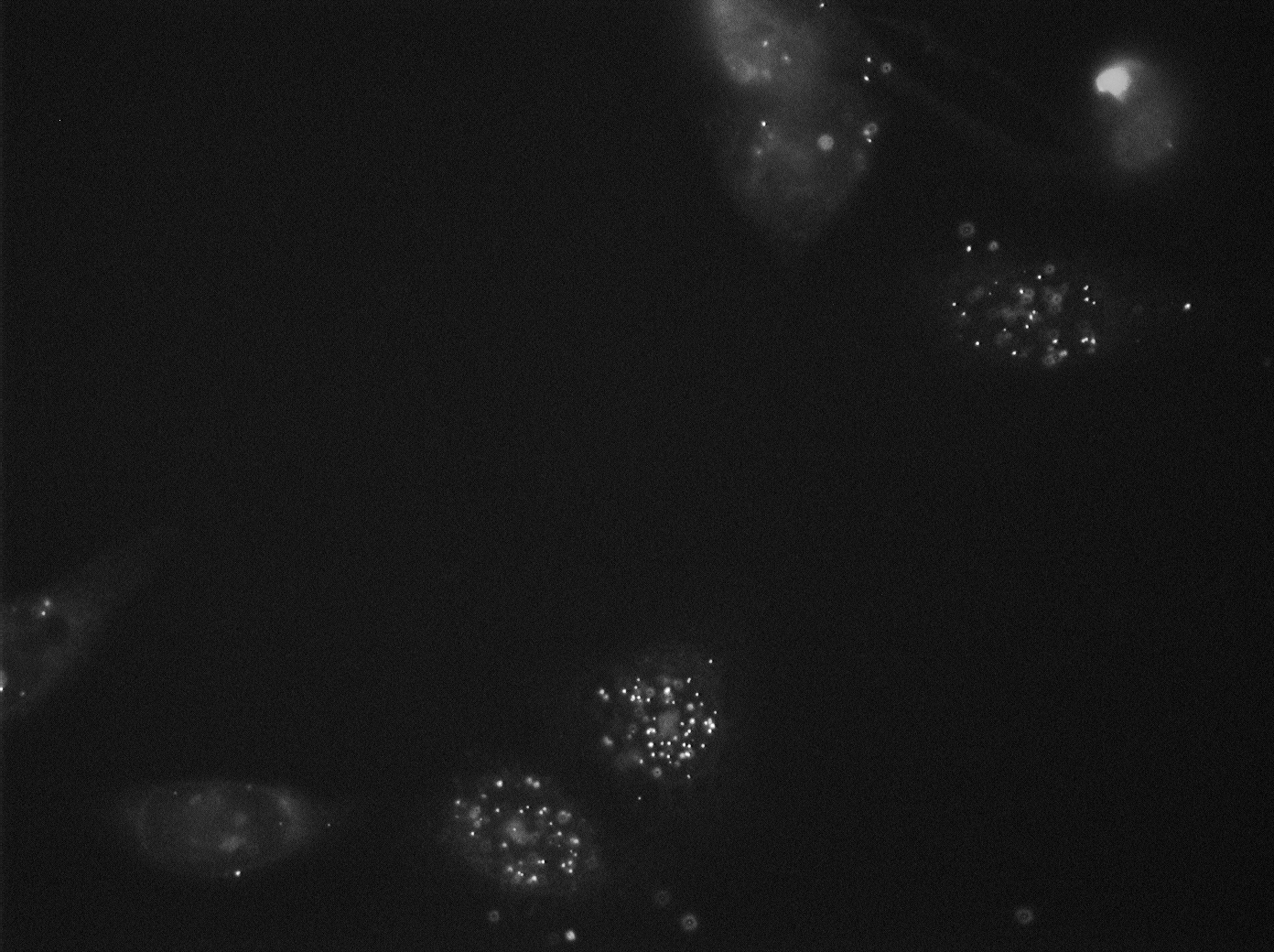

Supplement: Supplementary file 6 — Source data Fig. 5 [file 44318_2025_602_MOESM6_ESM.zip › Fig 5/A/siRNF20 UT SIRF.tif]

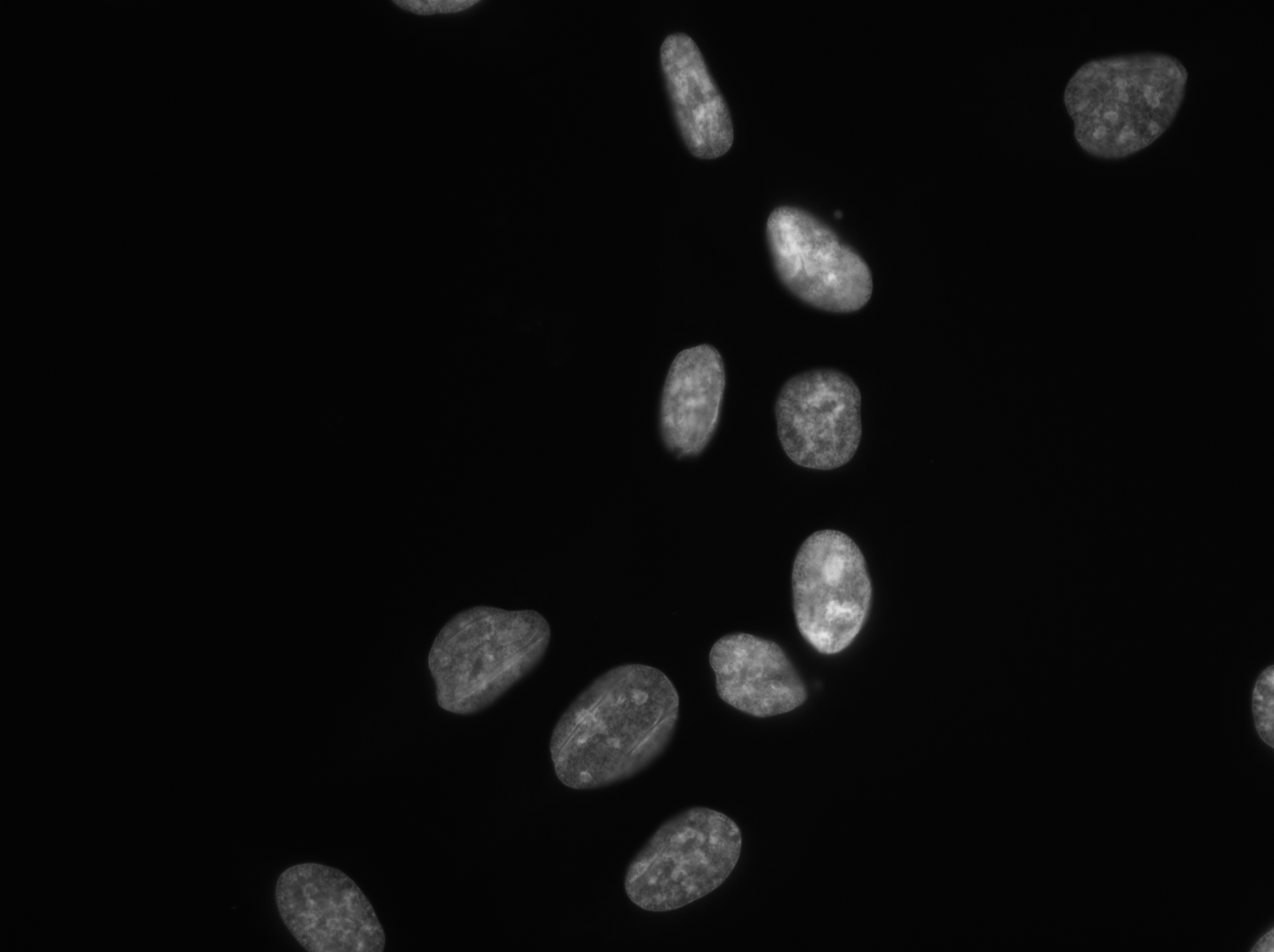

Supplement: Supplementary file 6 — Source data Fig. 5 [file 44318_2025_602_MOESM6_ESM.zip › Fig 5/A/siLuc UT DAPI.tif]

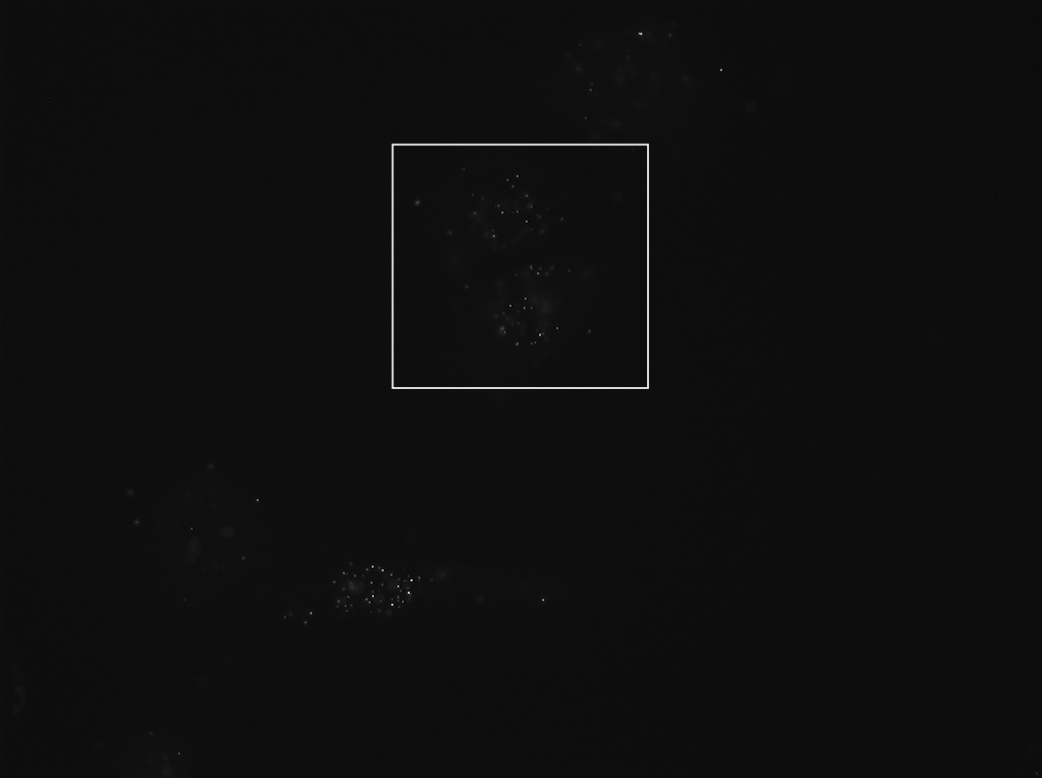

Supplement: Supplementary file 6 — Source data Fig. 5 [file 44318_2025_602_MOESM6_ESM.zip › Fig 5/A/siRNF20 CPT SIRF Screenshot.png]

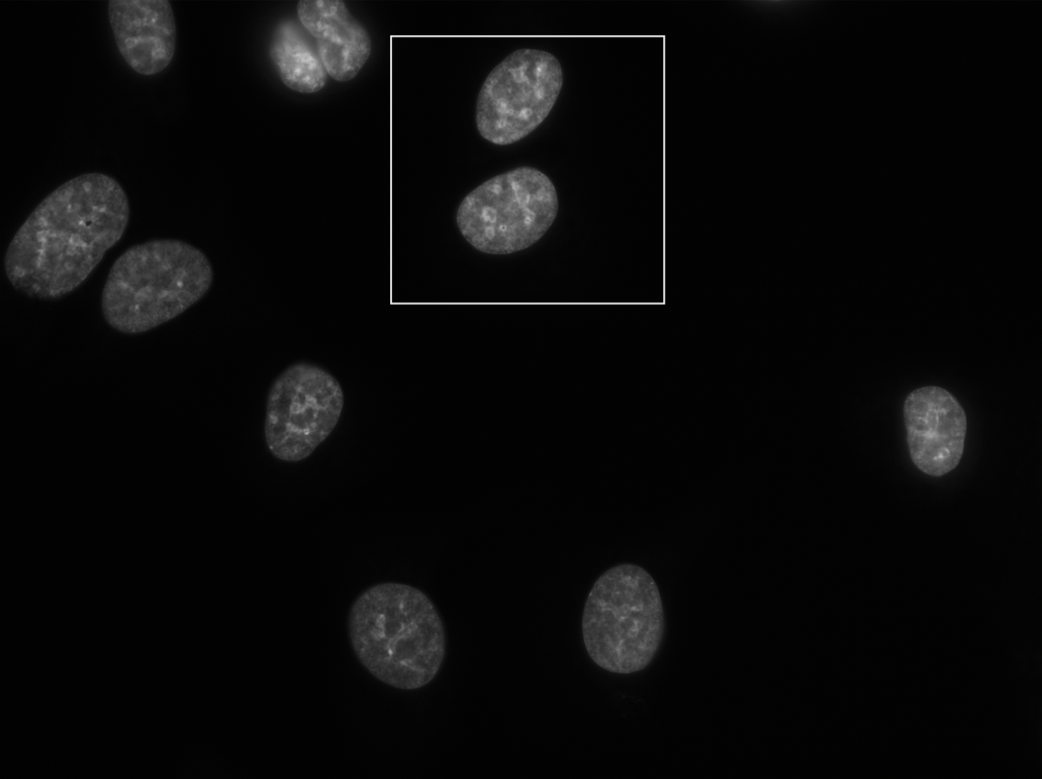

Supplement: Supplementary file 6 — Source data Fig. 5 [file 44318_2025_602_MOESM6_ESM.zip › Fig 5/A/siLuc CPT DAPI Screenshot.png]

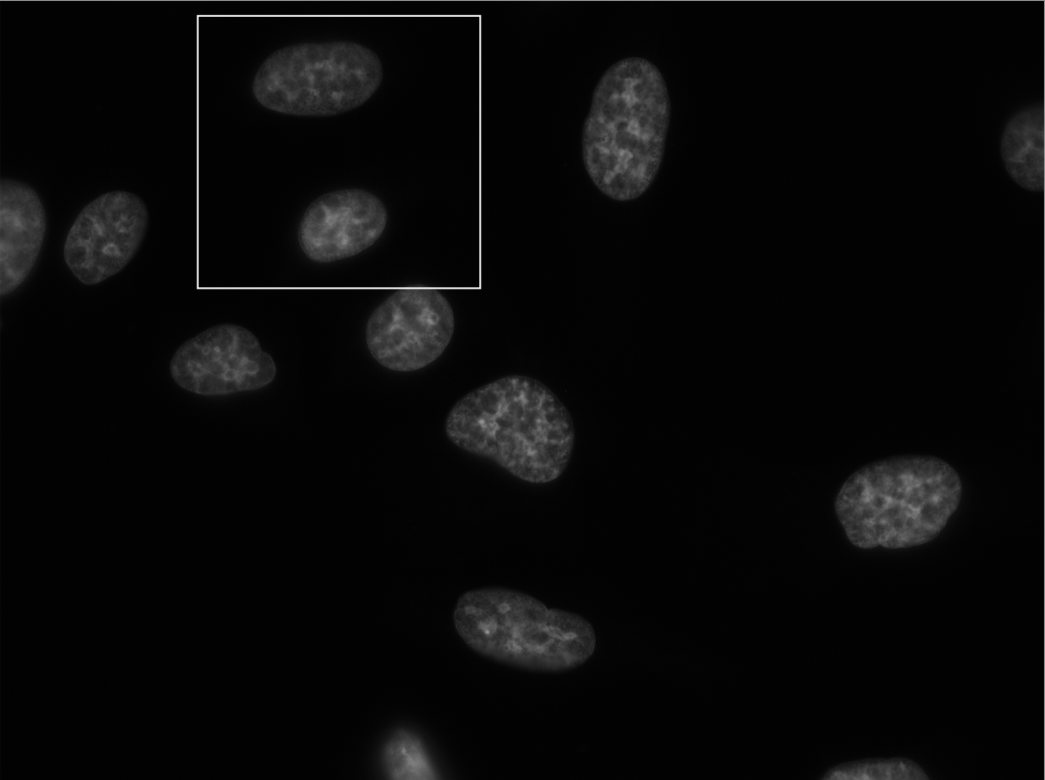

Supplement: Supplementary file 6 — Source data Fig. 5 [file 44318_2025_602_MOESM6_ESM.zip › Fig 5/A/siLuc HU DAPI Screenshot.png]

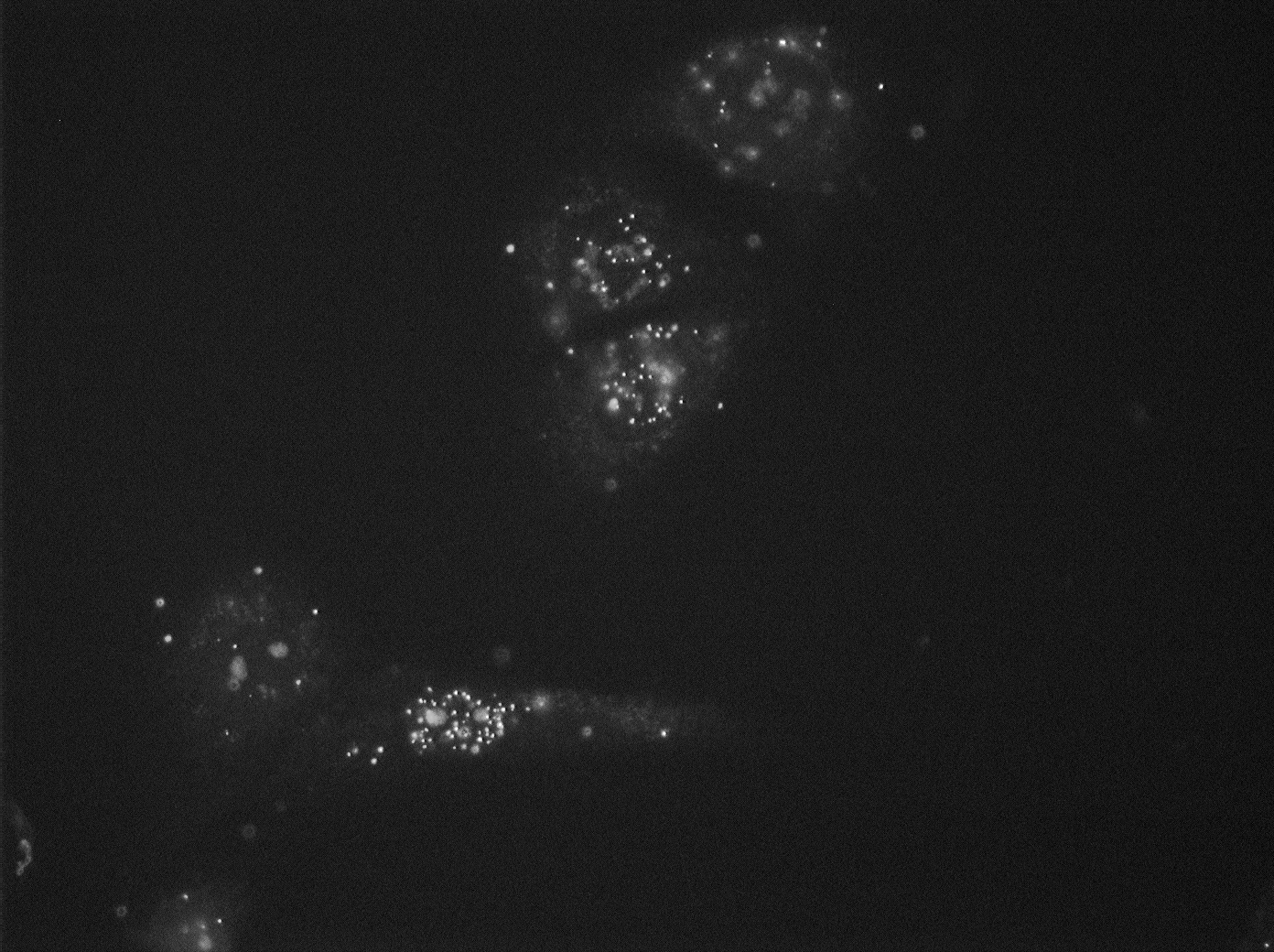

Supplement: Supplementary file 6 — Source data Fig. 5 [file 44318_2025_602_MOESM6_ESM.zip › Fig 5/A/siRNF20 CPT SIRF.tif]

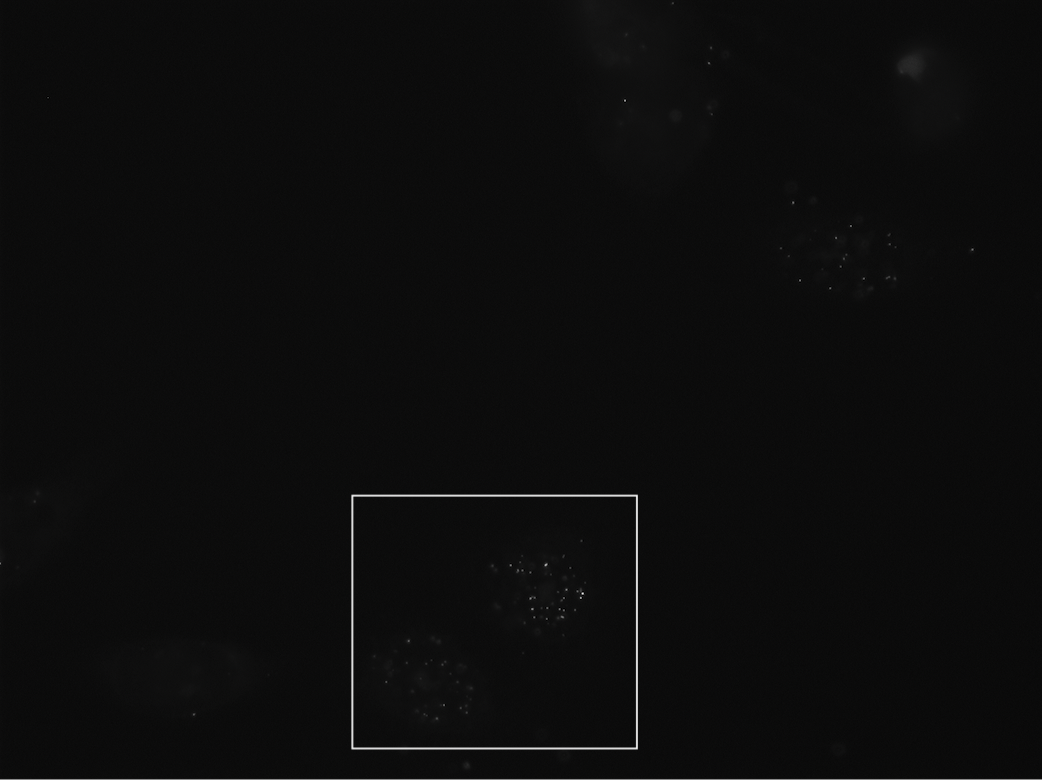

Supplement: Supplementary file 6 — Source data Fig. 5 [file 44318_2025_602_MOESM6_ESM.zip › Fig 5/A/siRNF20 UT SIRF Screenshot.png]

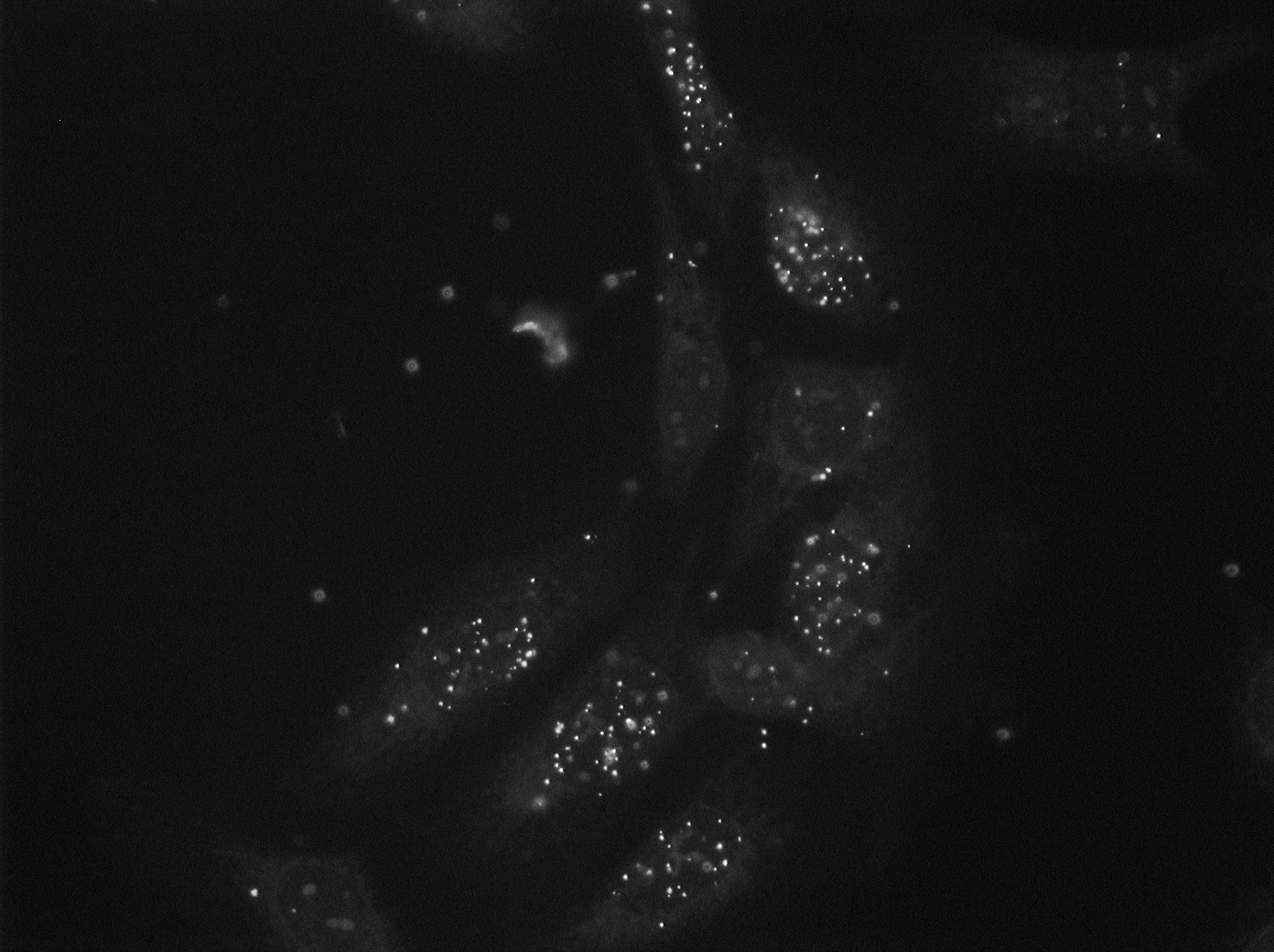

Supplement: Supplementary file 6 — Source data Fig. 5 [file 44318_2025_602_MOESM6_ESM.zip › Fig 5/A/siLuc UT SIRF.tif]

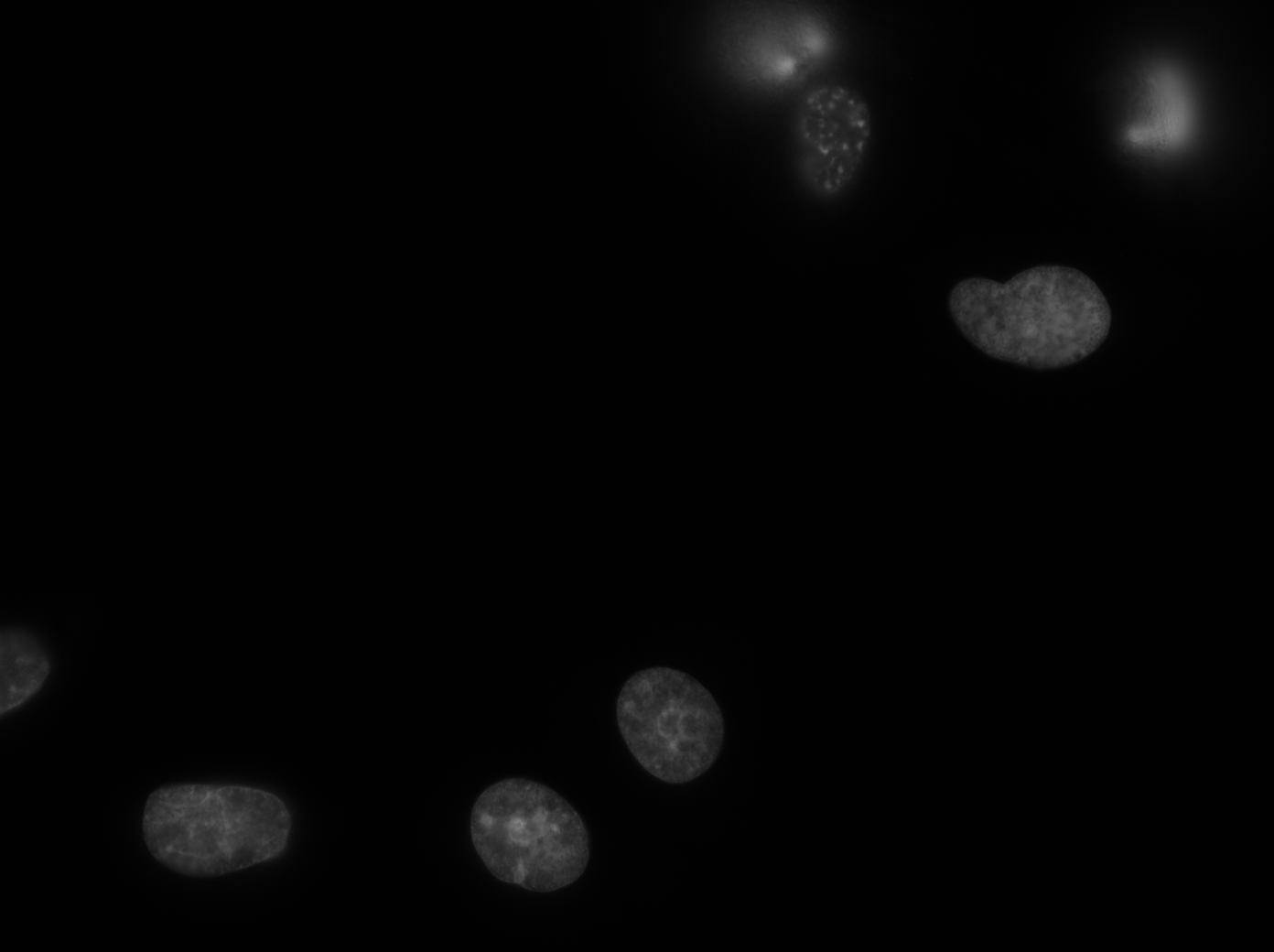

Supplement: Supplementary file 6 — Source data Fig. 5 [file 44318_2025_602_MOESM6_ESM.zip › Fig 5/A/siRNF20 UT DAPI.tif]

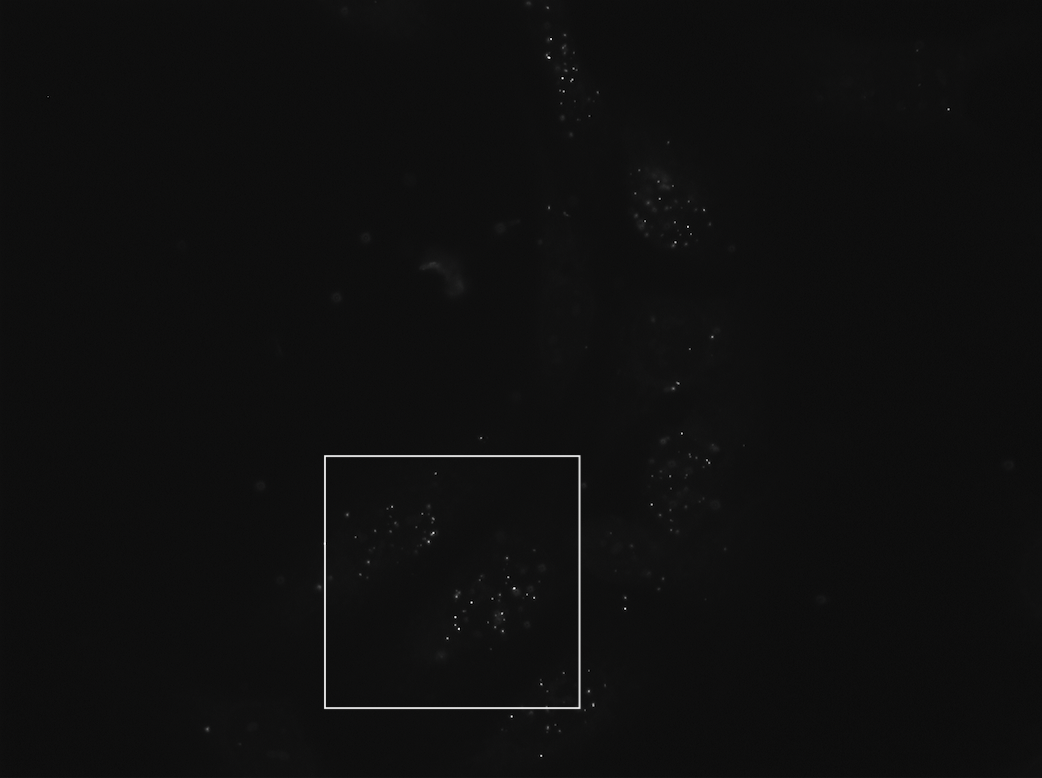

Supplement: Supplementary file 6 — Source data Fig. 5 [file 44318_2025_602_MOESM6_ESM.zip › Fig 5/A/siLuc UT SIRF Screenshot.png]

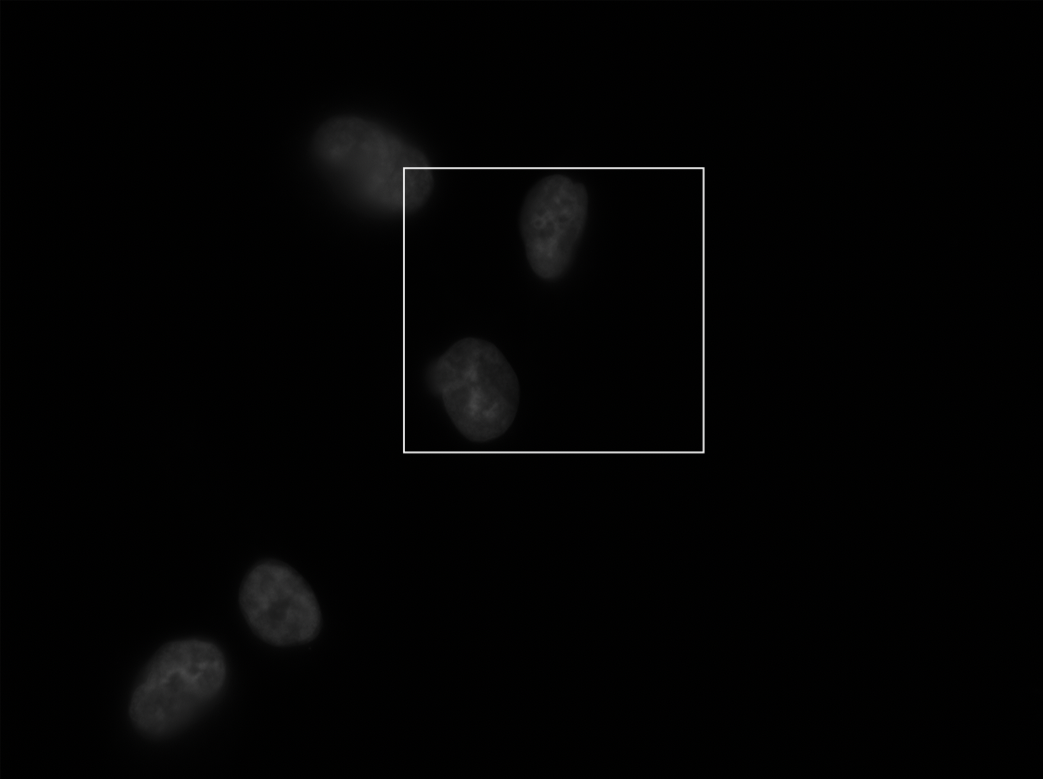

Supplement: Supplementary file 6 — Source data Fig. 5 [file 44318_2025_602_MOESM6_ESM.zip › Fig 5/A/siRNF20 HU DAPI Screenshot.png]

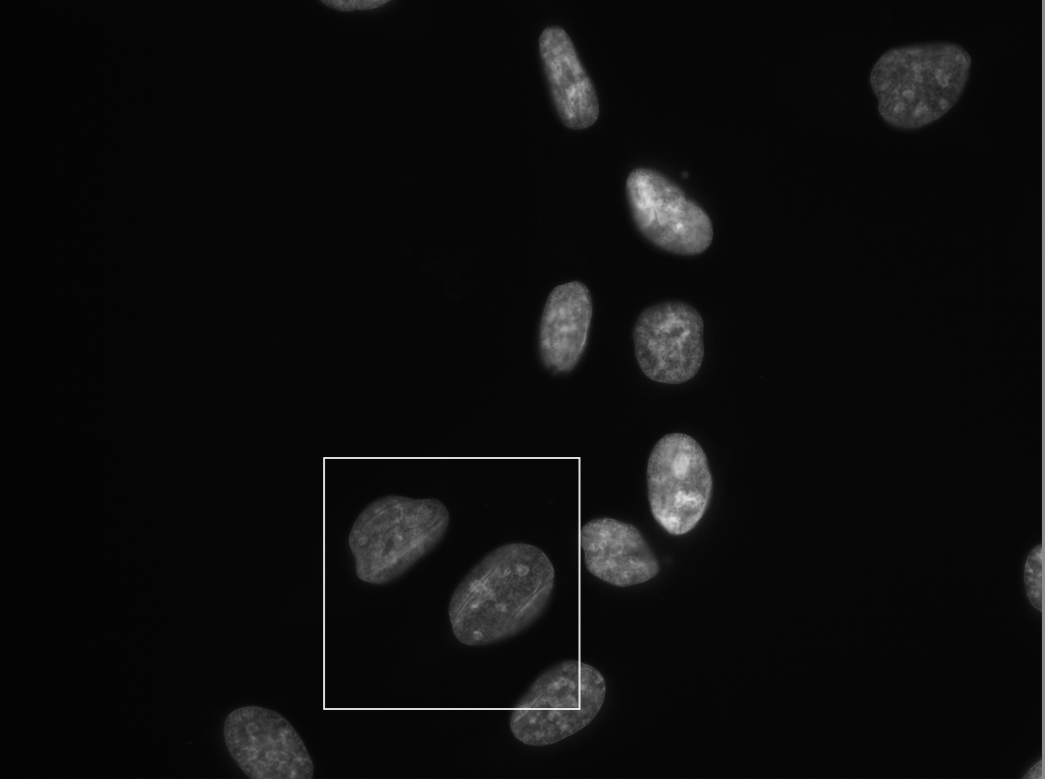

Supplement: Supplementary file 6 — Source data Fig. 5 [file 44318_2025_602_MOESM6_ESM.zip › Fig 5/A/siLuc UT DAPI Screenshot.png]

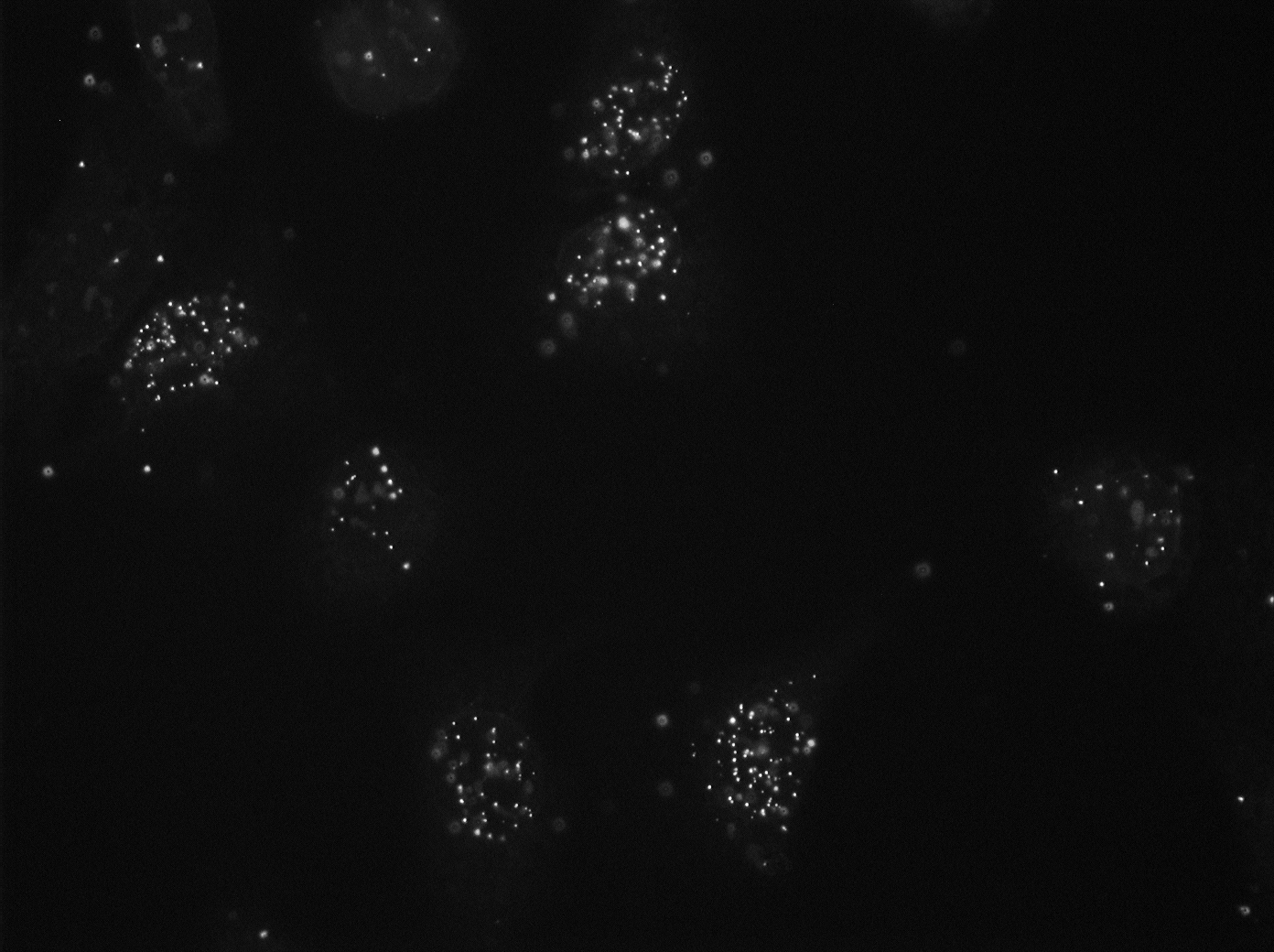

Supplement: Supplementary file 6 — Source data Fig. 5 [file 44318_2025_602_MOESM6_ESM.zip › Fig 5/A/siLuc CPT SIRF.tif]

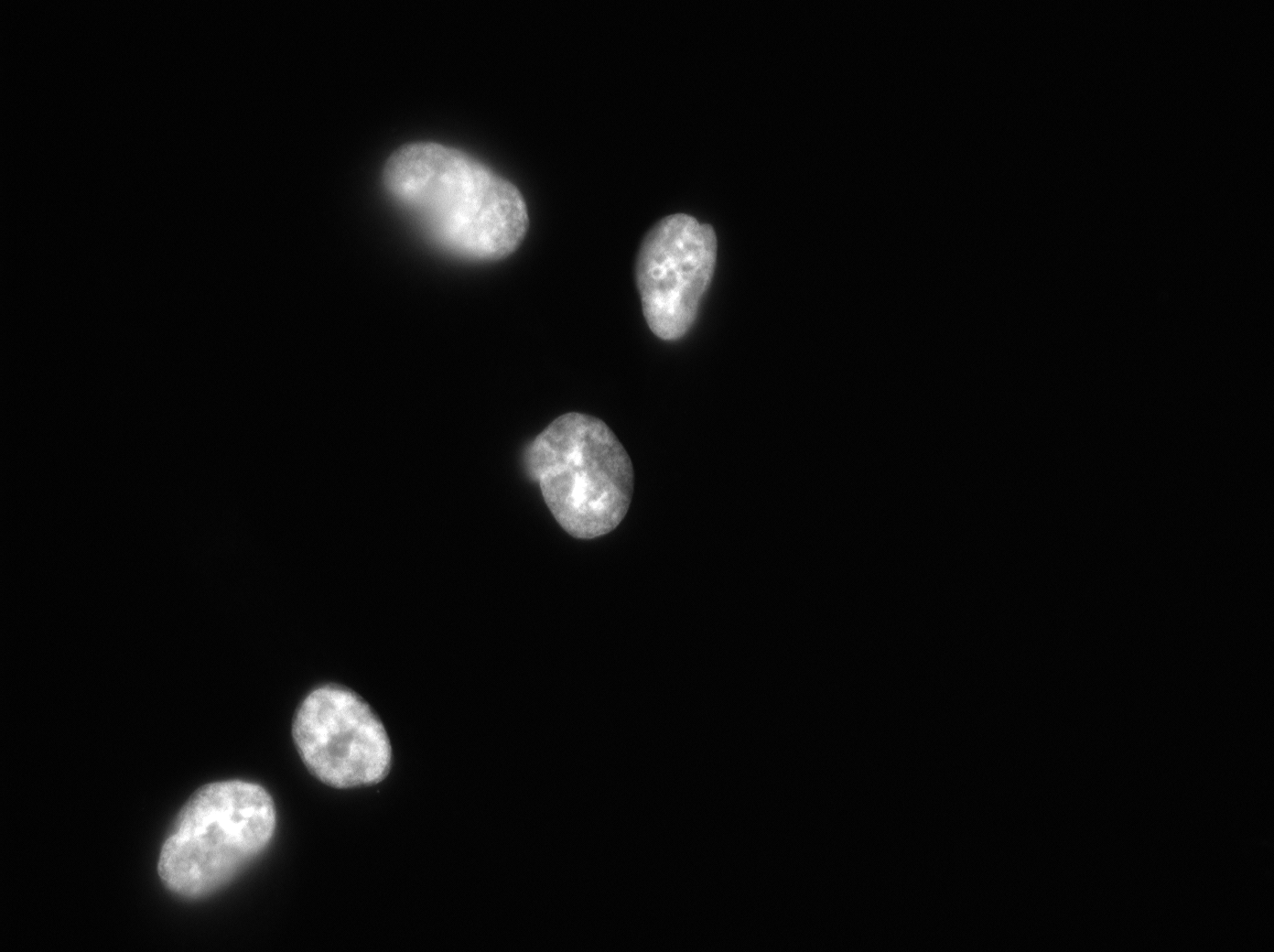

Supplement: Supplementary file 6 — Source data Fig. 5 [file 44318_2025_602_MOESM6_ESM.zip › Fig 5/A/siRNF20 HU DAPI.tif]

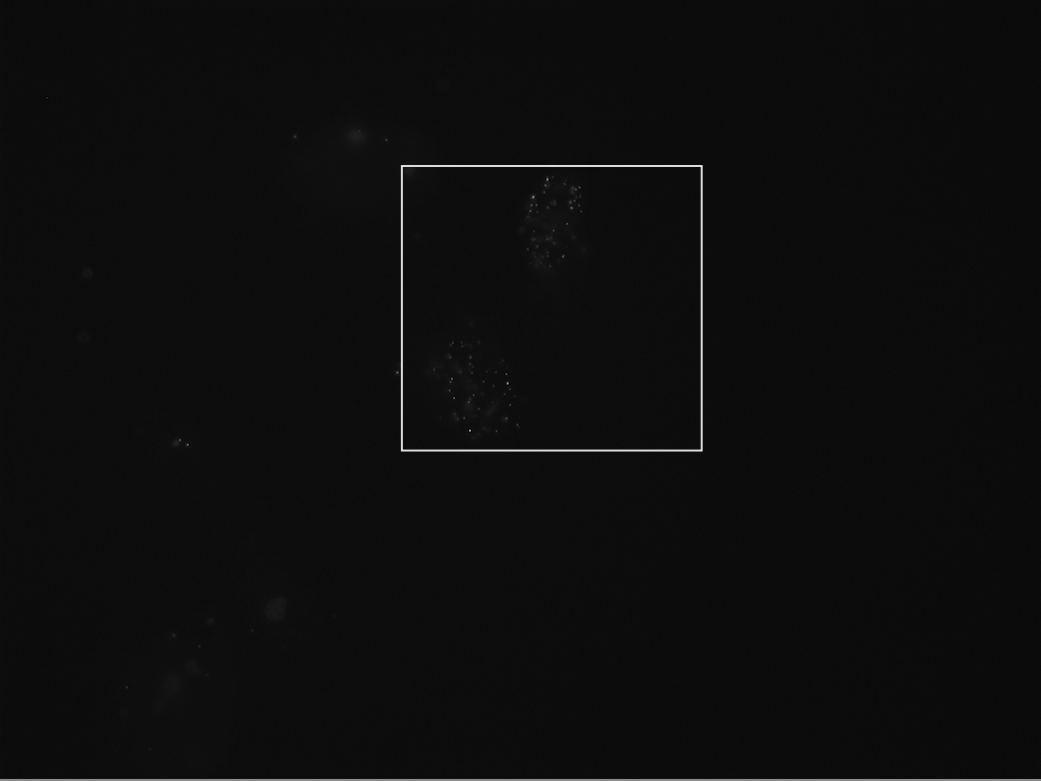

Supplement: Supplementary file 6 — Source data Fig. 5 [file 44318_2025_602_MOESM6_ESM.zip › Fig 5/A/siRNF20 HU SIRF Screenshot.png]

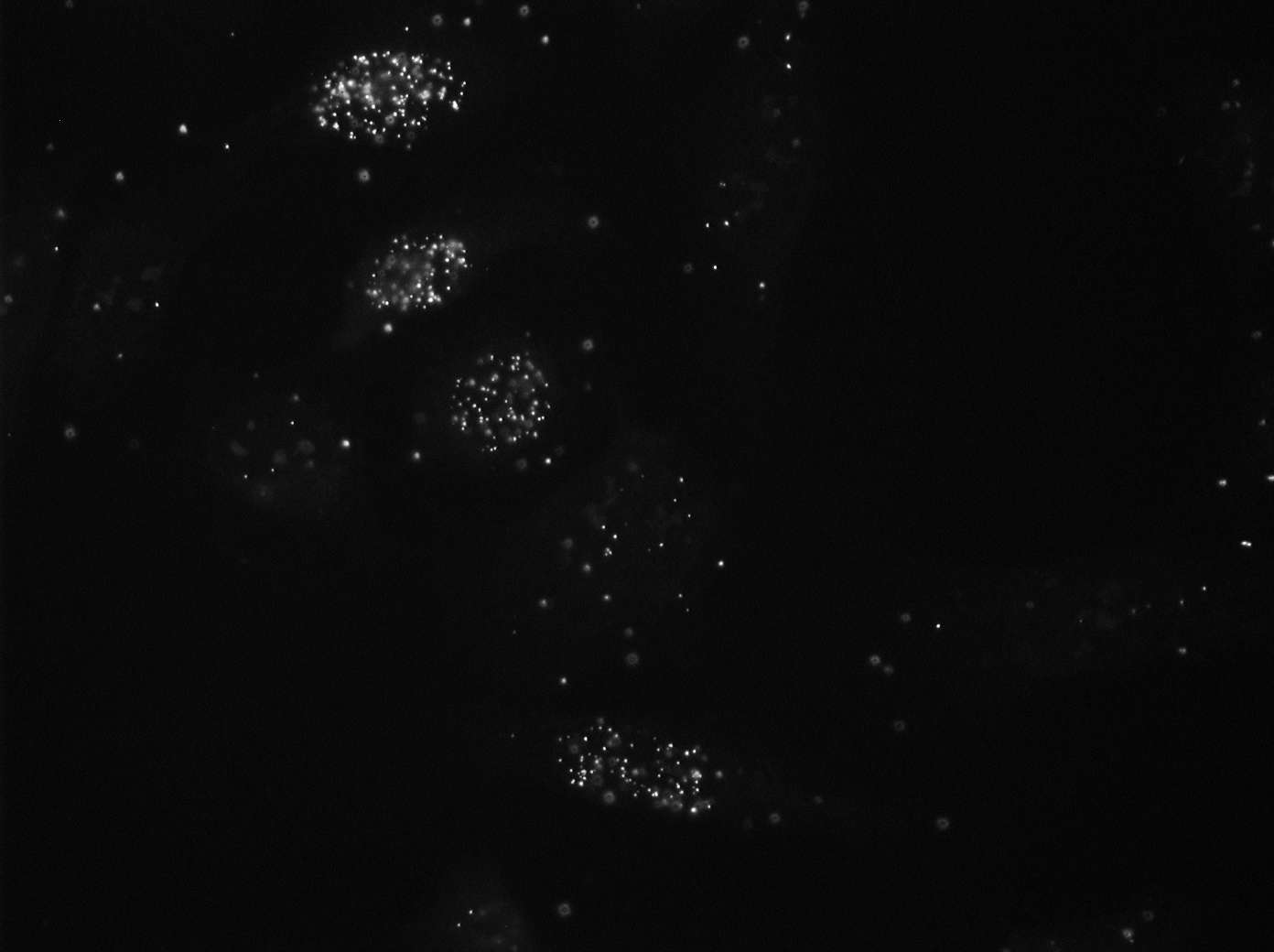

Supplement: Supplementary file 6 — Source data Fig. 5 [file 44318_2025_602_MOESM6_ESM.zip › Fig 5/A/siLuc HU SIRF.tif]

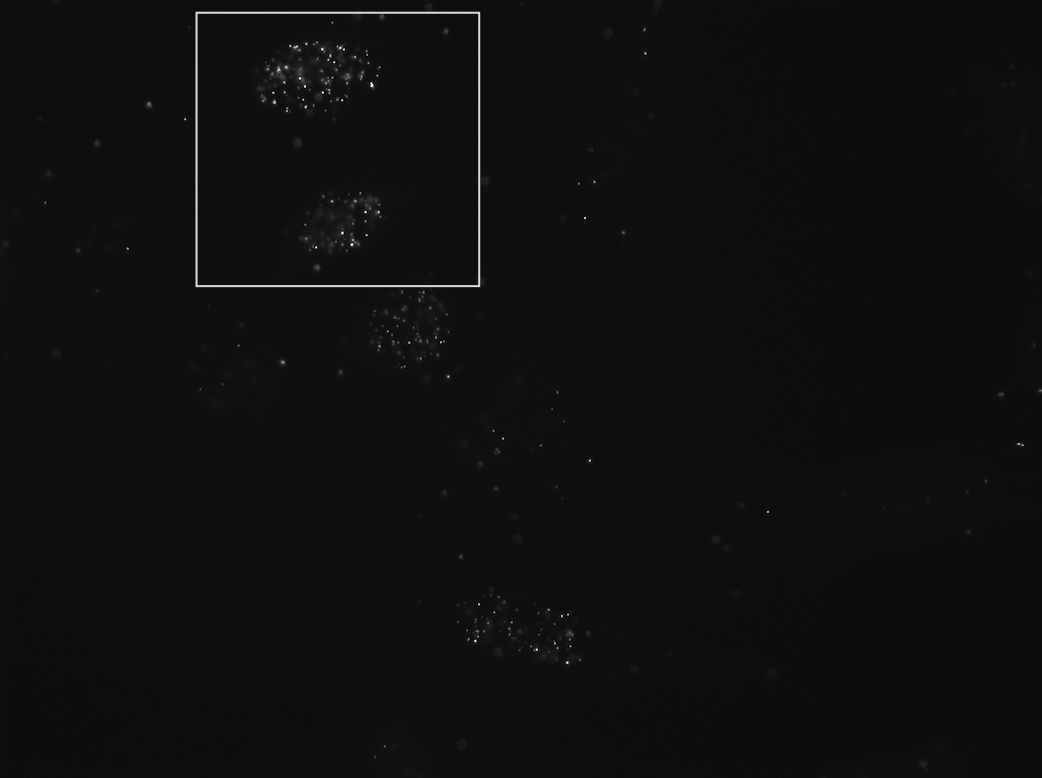

Supplement: Supplementary file 6 — Source data Fig. 5 [file 44318_2025_602_MOESM6_ESM.zip › Fig 5/A/siLuc HU SIRF Screenshot.png]

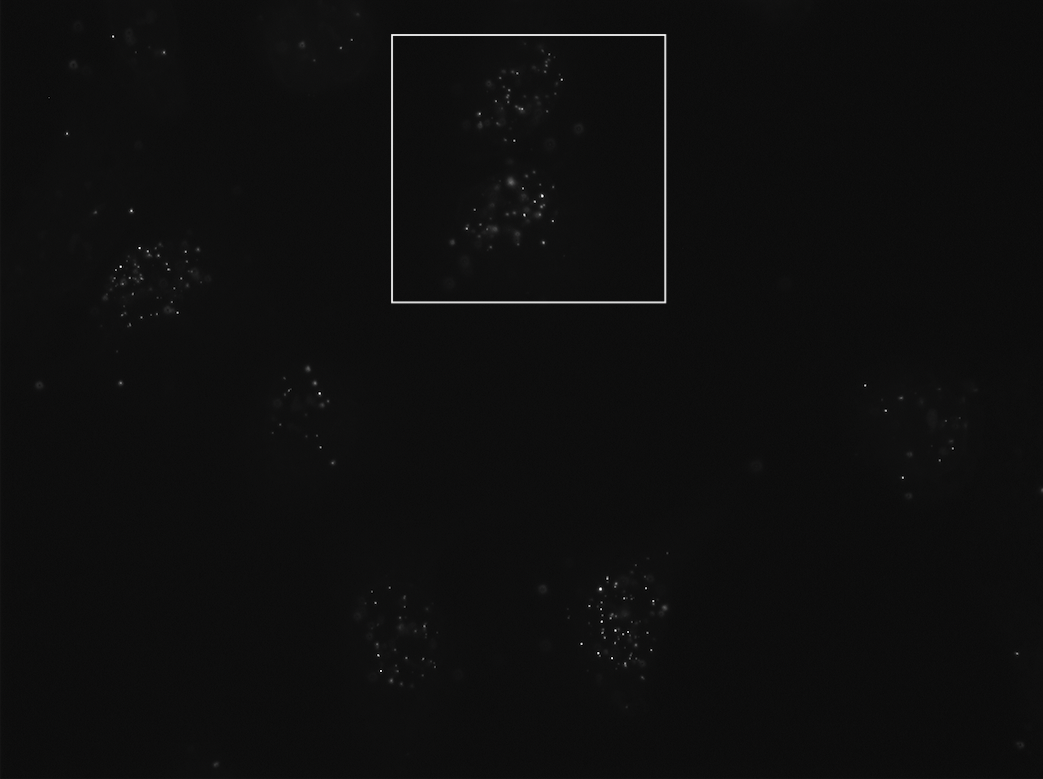

Supplement: Supplementary file 6 — Source data Fig. 5 [file 44318_2025_602_MOESM6_ESM.zip › Fig 5/A/siLuc CPT SIRF Screenshot.png]

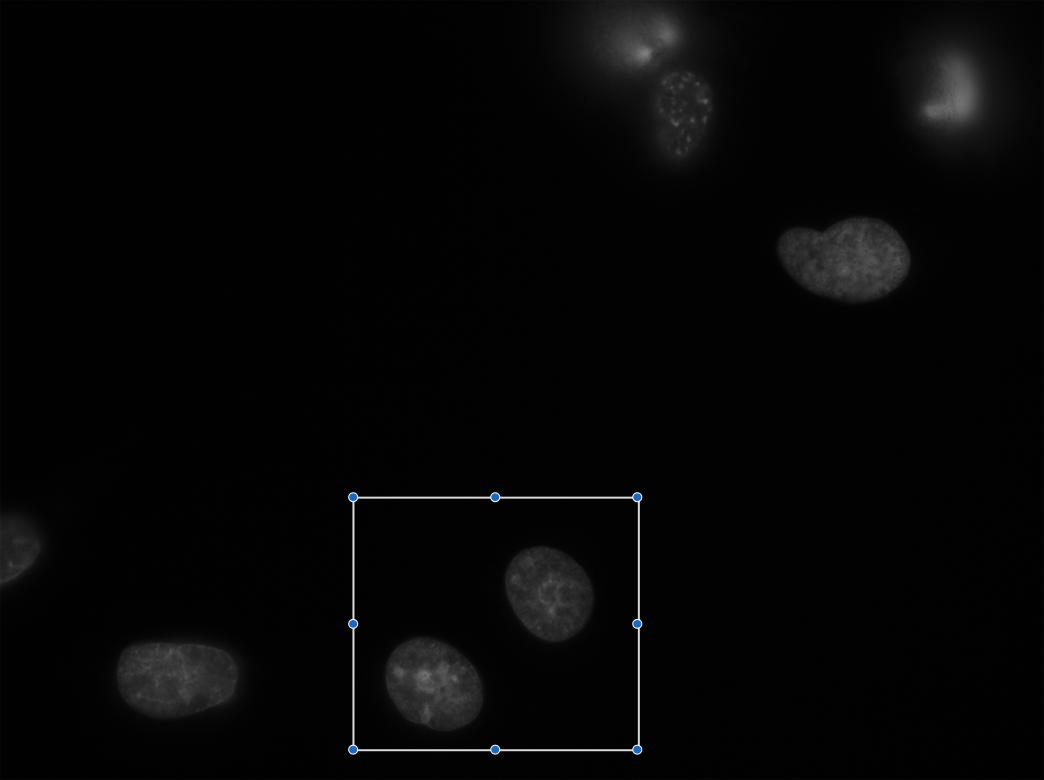

Supplement: Supplementary file 6 — Source data Fig. 5 [file 44318_2025_602_MOESM6_ESM.zip › Fig 5/A/siRNF20 UT DAPI Screenshot.png]

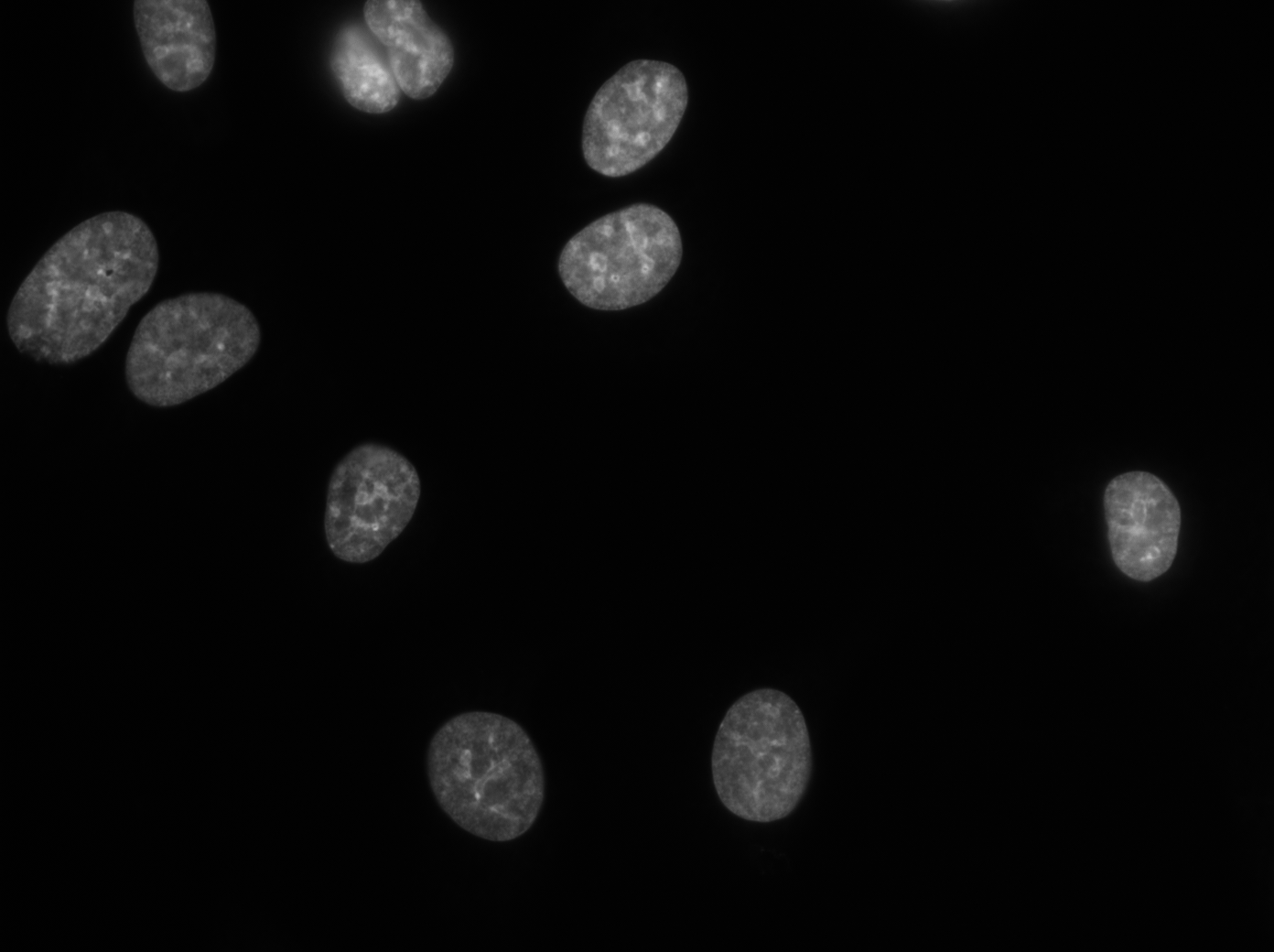

Supplement: Supplementary file 6 — Source data Fig. 5 [file 44318_2025_602_MOESM6_ESM.zip › Fig 5/A/siLuc CPT DAPI.tif]

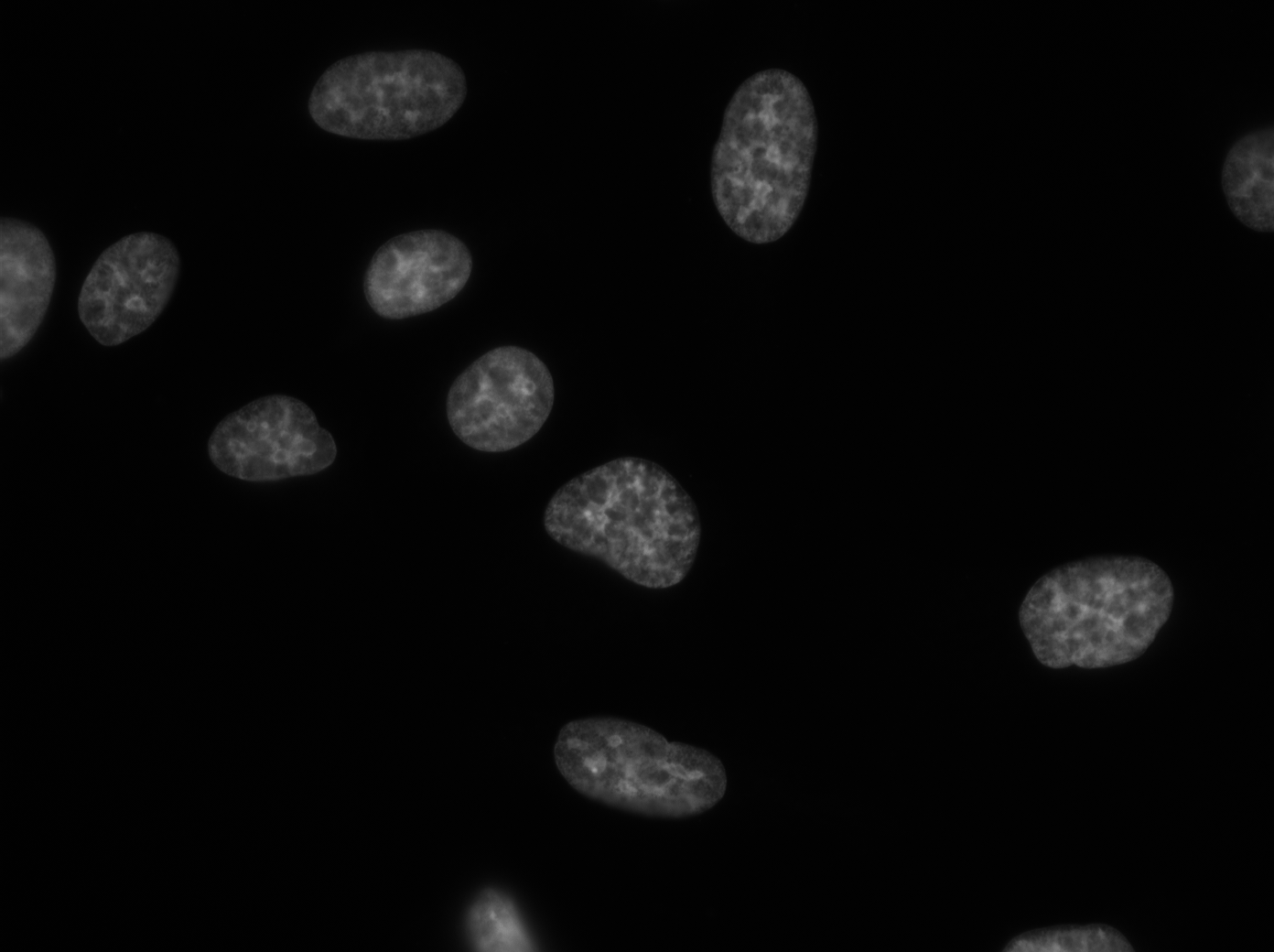

Supplement: Supplementary file 6 — Source data Fig. 5 [file 44318_2025_602_MOESM6_ESM.zip › Fig 5/A/siLuc HU DAPI.tif]

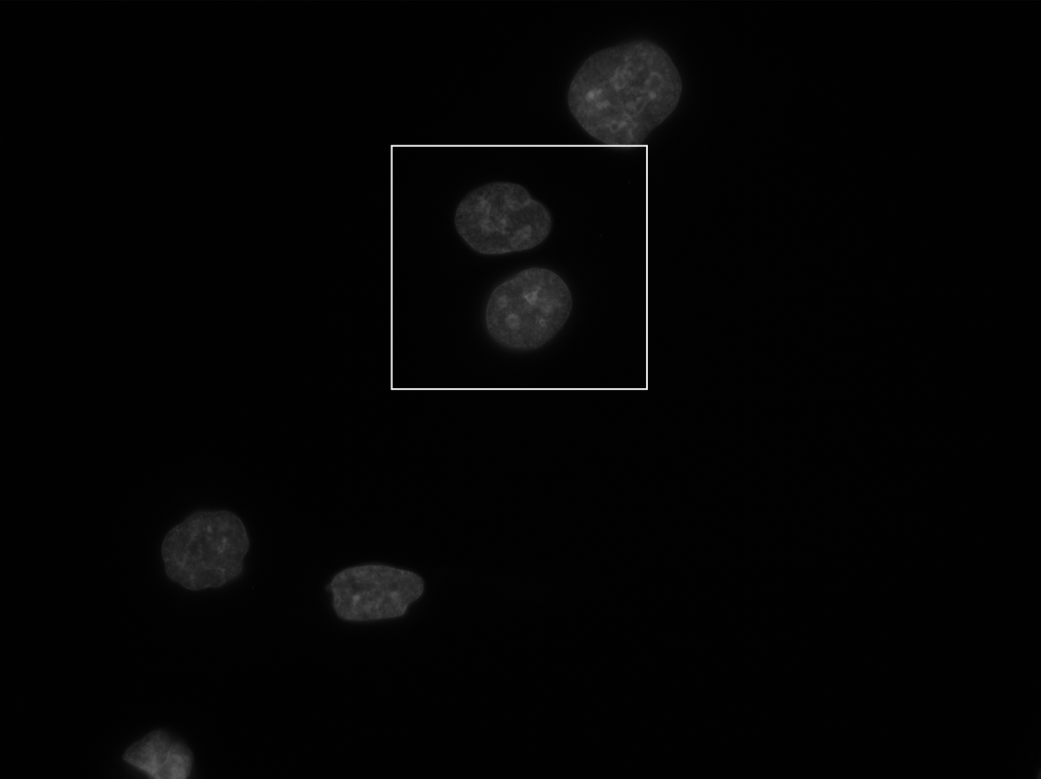

Supplement: Supplementary file 6 — Source data Fig. 5 [file 44318_2025_602_MOESM6_ESM.zip › Fig 5/A/siRNF20 CPT DAPI Screenshot.png]

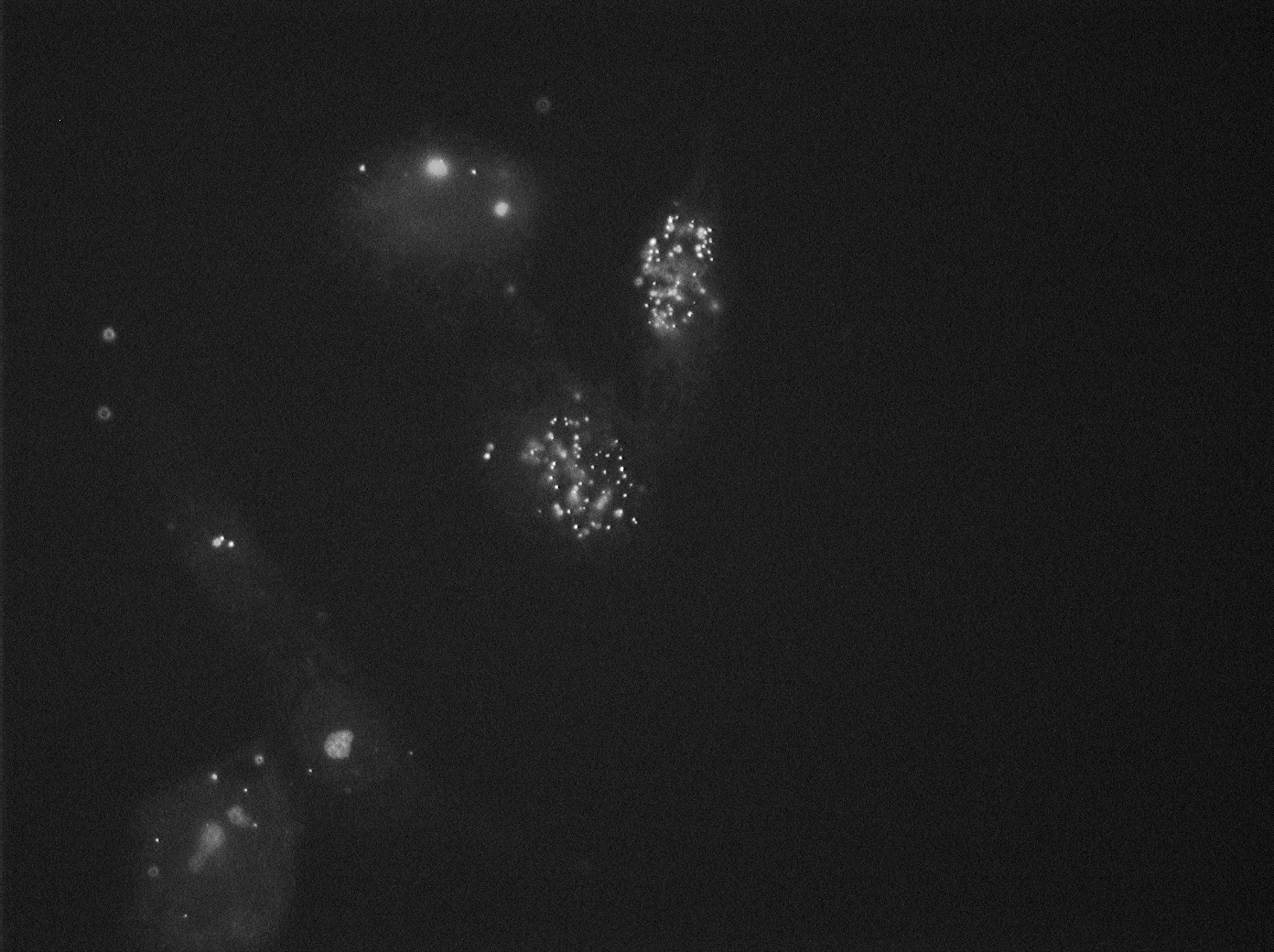

Supplement: Supplementary file 6 — Source data Fig. 5 [file 44318_2025_602_MOESM6_ESM.zip › Fig 5/A/siRNF20 HU SIRF.tif]

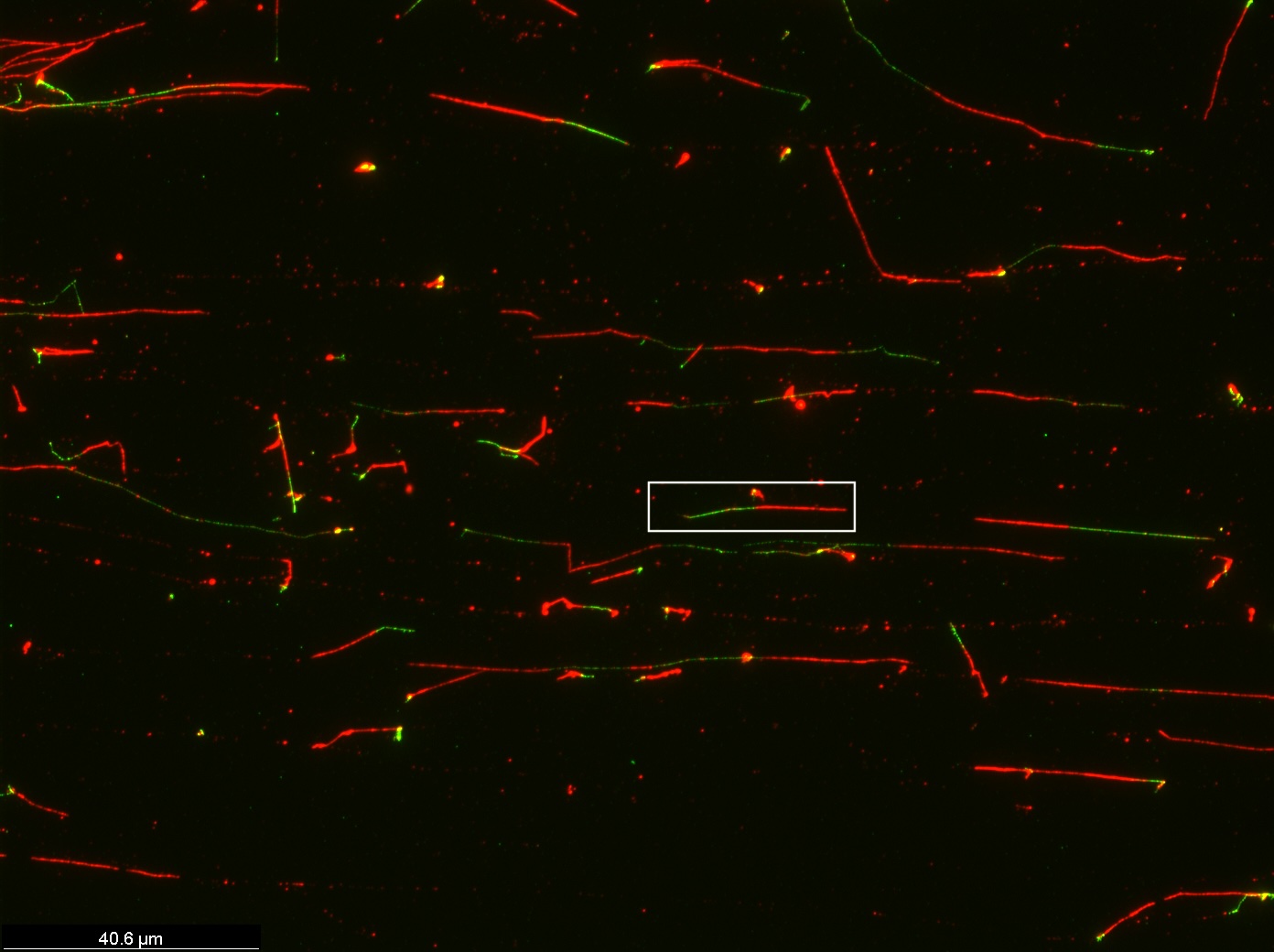

Supplement: Supplementary file 6 — Source data Fig. 5 [file 44318_2025_602_MOESM6_ESM.zip › Fig 5/F/siRNF169_2.jpg]

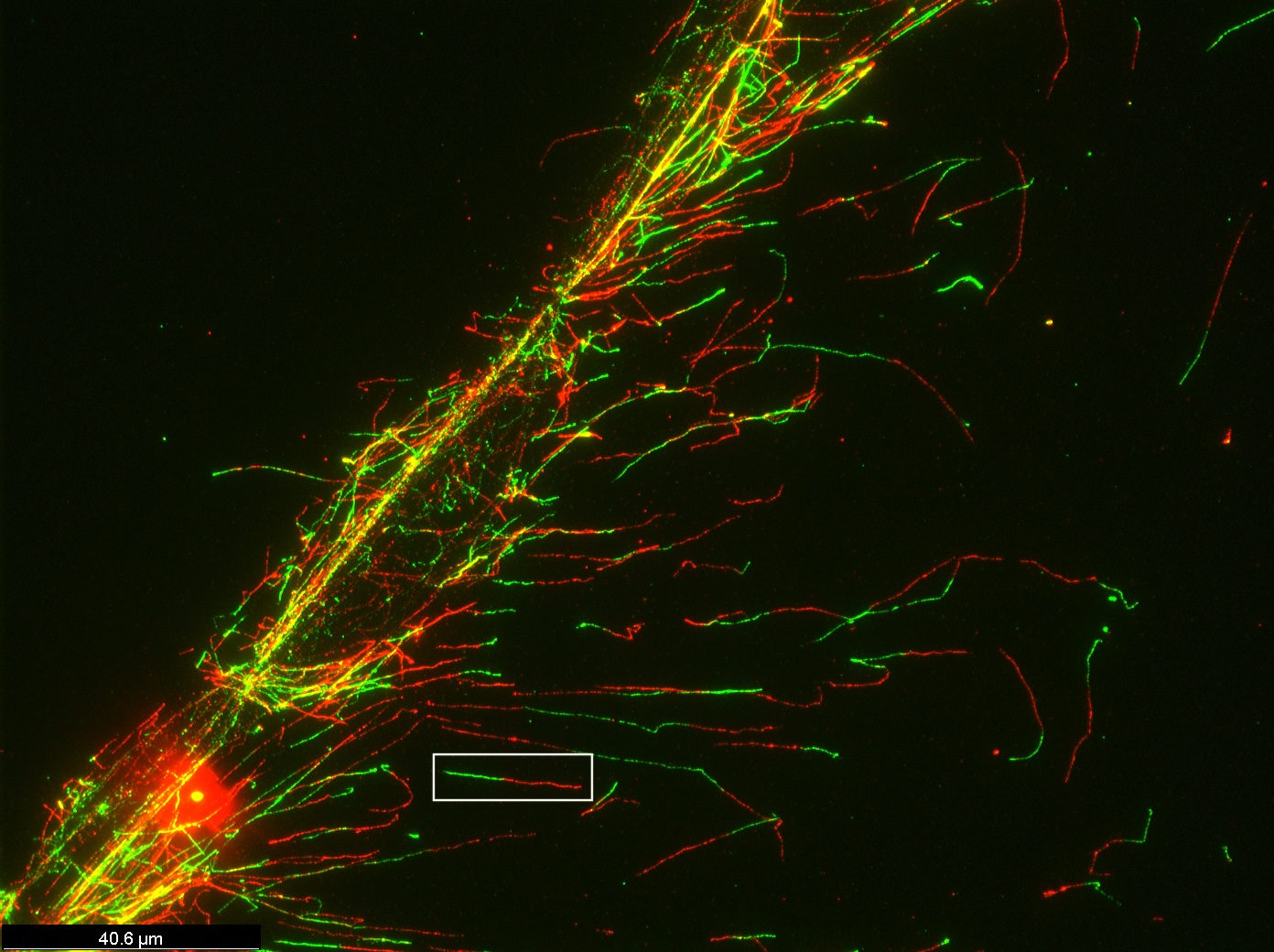

Supplement: Supplementary file 6 — Source data Fig. 5 [file 44318_2025_602_MOESM6_ESM.zip › Fig 5/F/siRNF169_1.jpg]

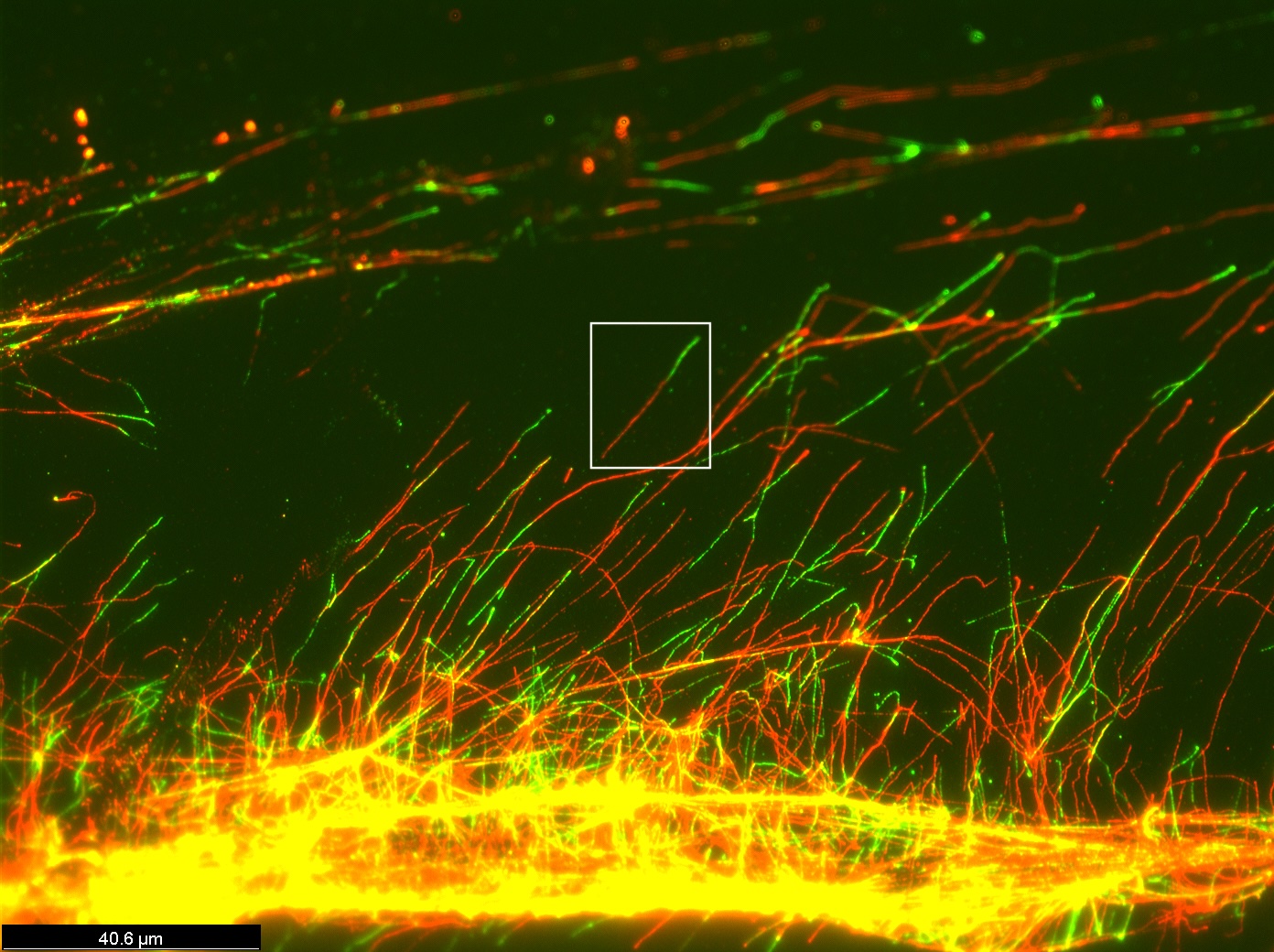

Supplement: Supplementary file 6 — Source data Fig. 5 [file 44318_2025_602_MOESM6_ESM.zip › Fig 5/F/siBRCA2.jpg]

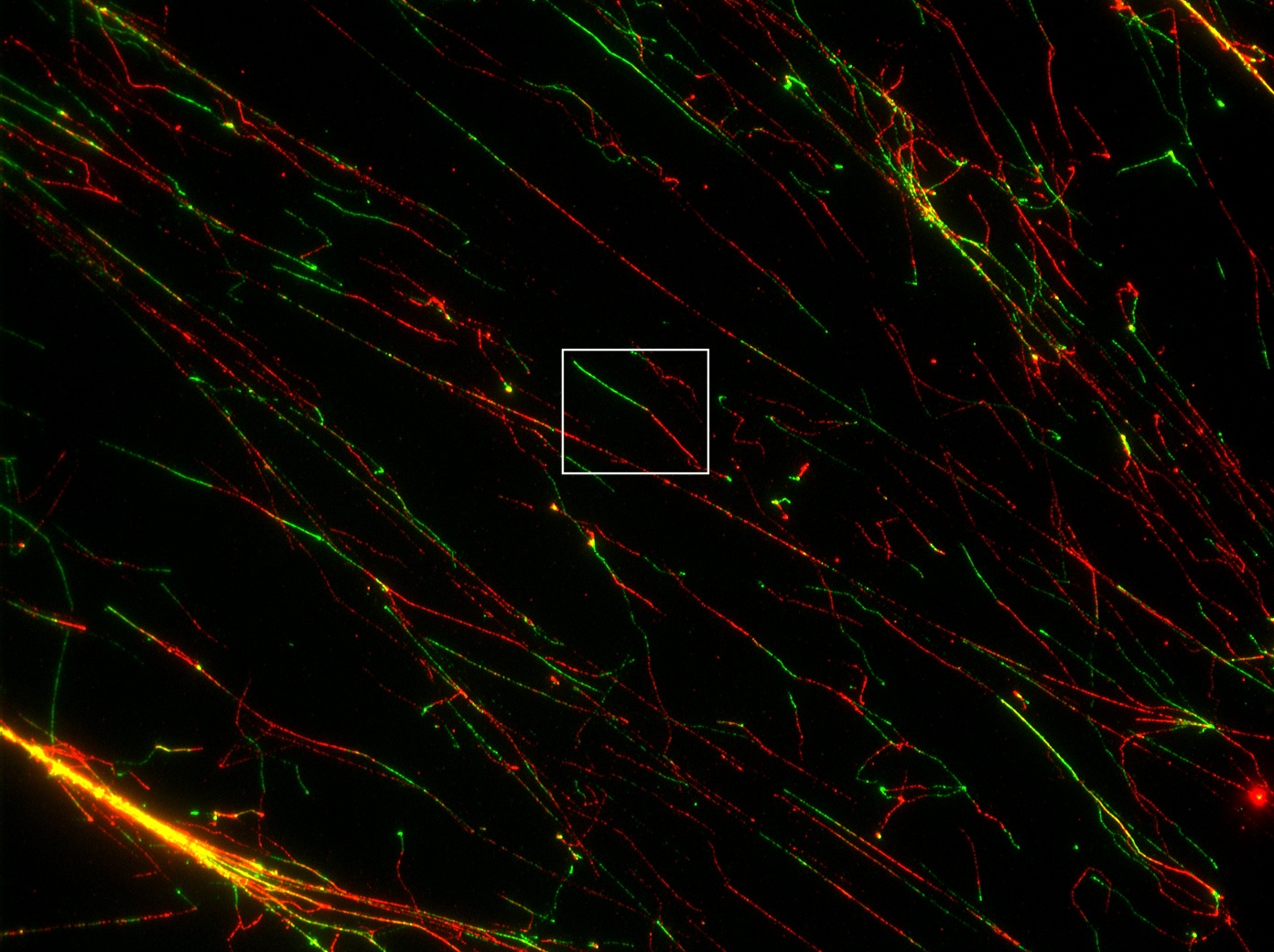

Supplement: Supplementary file 6 — Source data Fig. 5 [file 44318_2025_602_MOESM6_ESM.zip › Fig 5/F/siLuc.jpg]

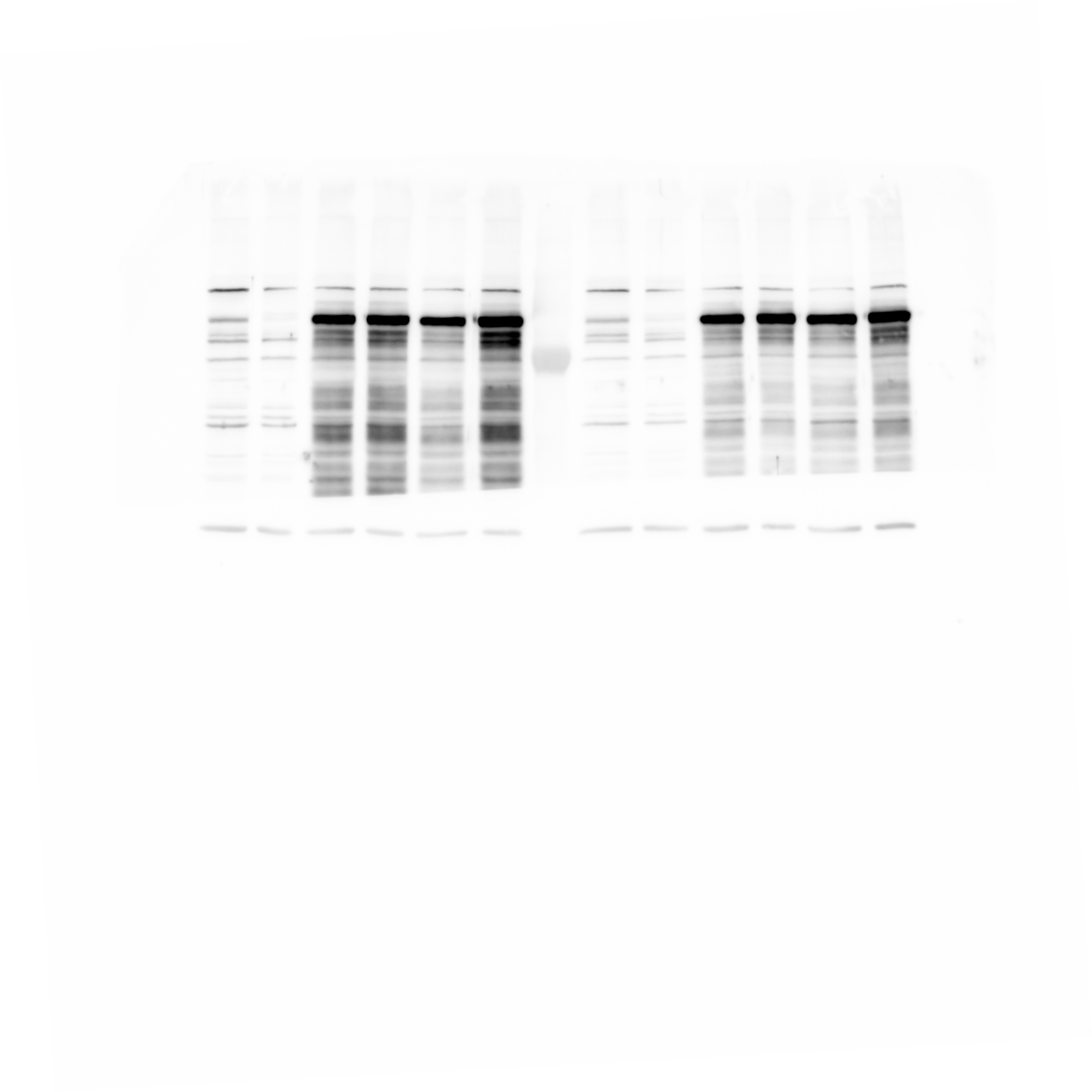

Supplement: Supplementary file 6 — Source data Fig. 5 [file 44318_2025_602_MOESM6_ESM.zip › Fig 5/H/RNF169.TIF]

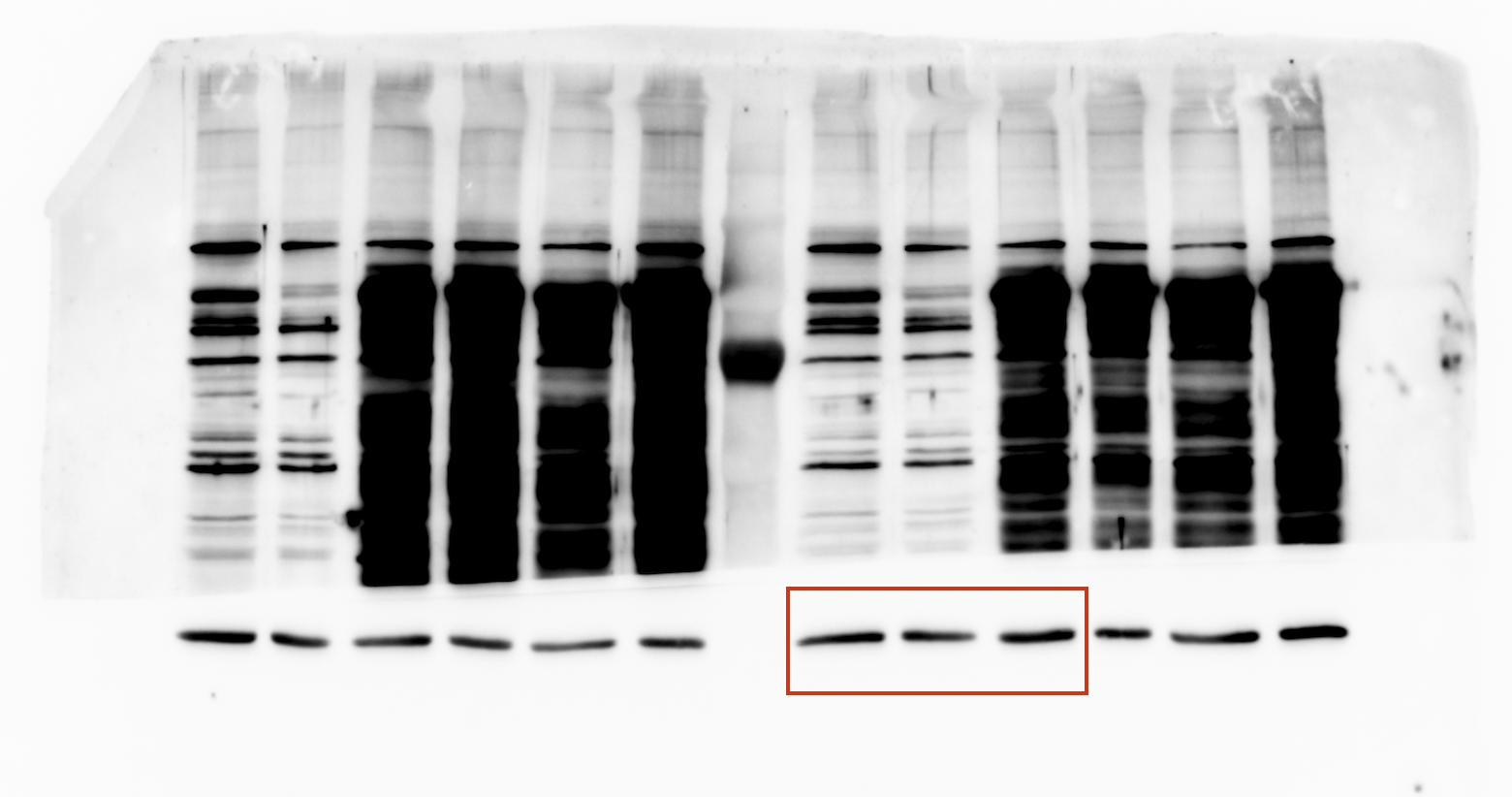

Supplement: Supplementary file 6 — Source data Fig. 5 [file 44318_2025_602_MOESM6_ESM.zip › Fig 5/H/GAPDH Screenshot.png]

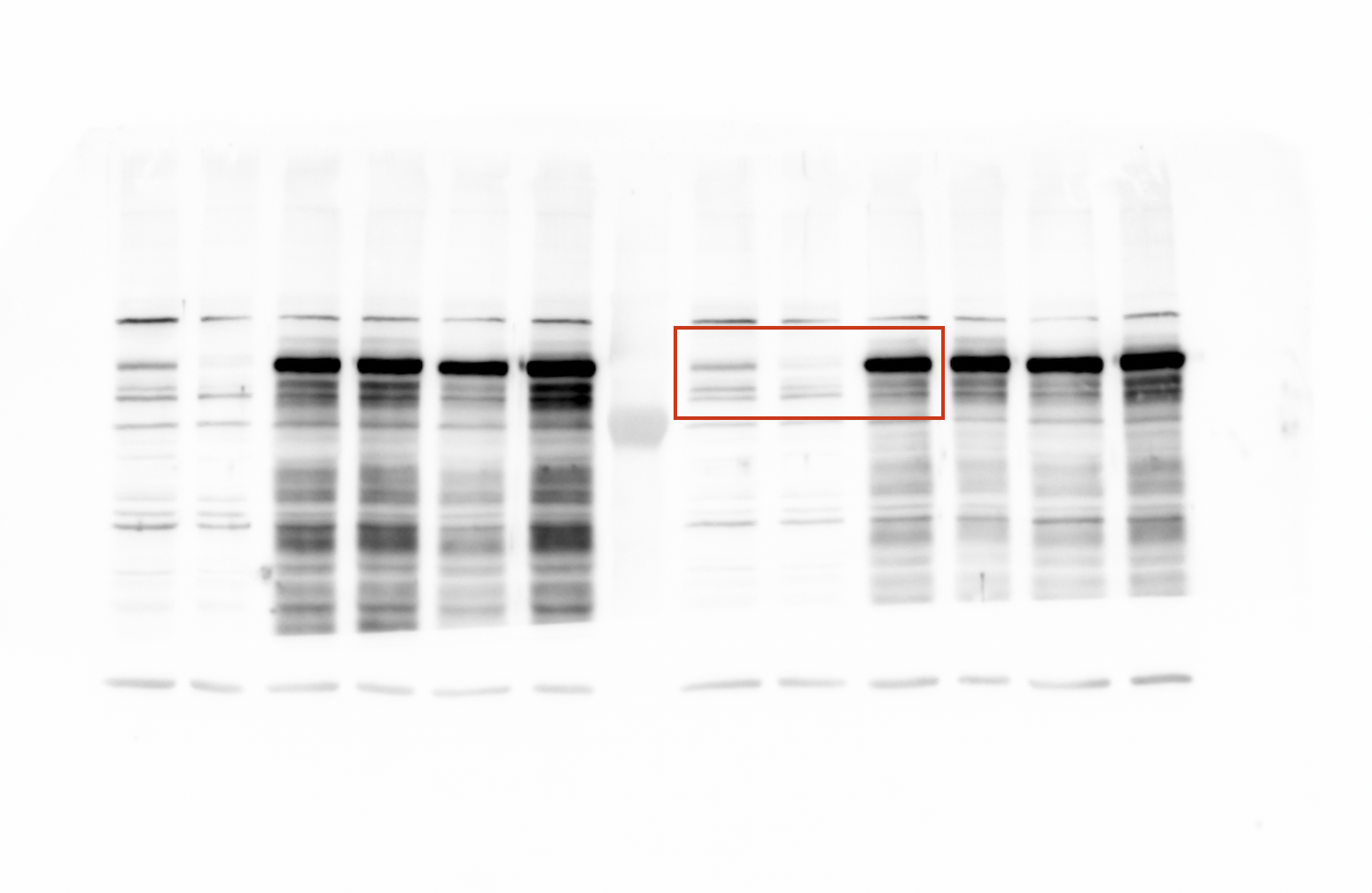

Supplement: Supplementary file 6 — Source data Fig. 5 [file 44318_2025_602_MOESM6_ESM.zip › Fig 5/H/RNF169 Screenshot.png]

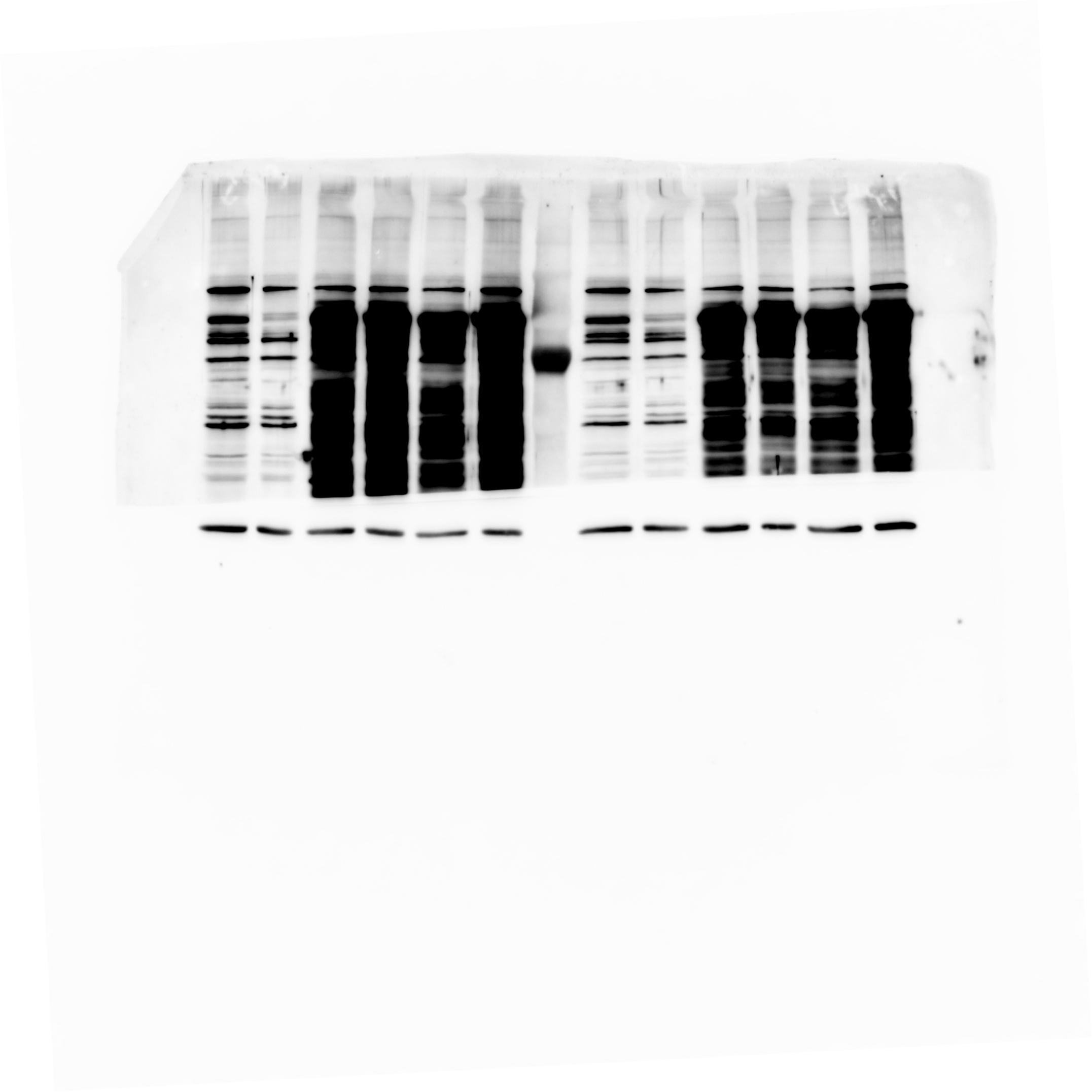

Supplement: Supplementary file 6 — Source data Fig. 5 [file 44318_2025_602_MOESM6_ESM.zip › Fig 5/H/GAPDH.TIF]

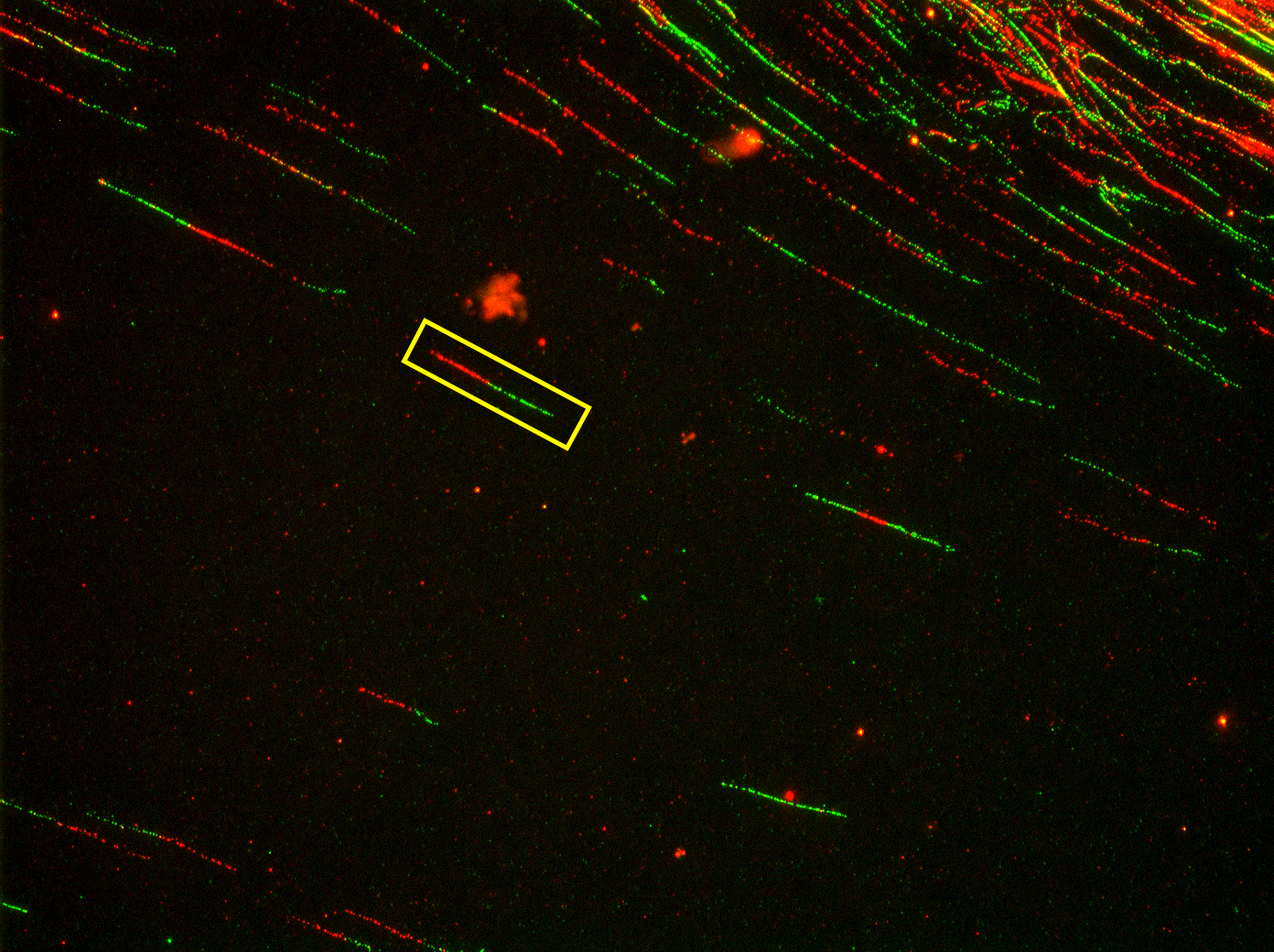

Supplement: Supplementary file 6 — Source data Fig. 5 [file 44318_2025_602_MOESM6_ESM.zip › Fig 5/K/si20.tif]

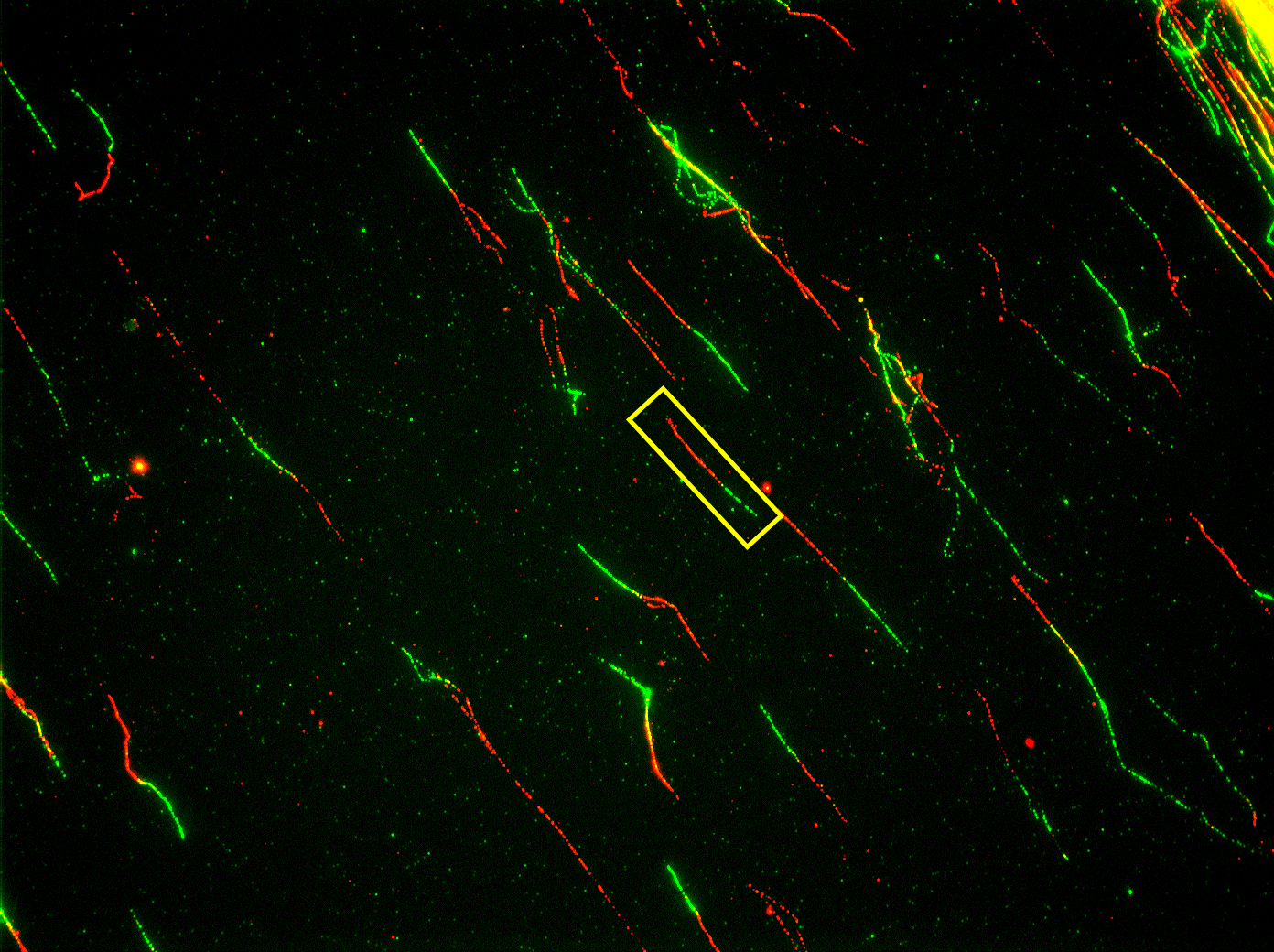

Supplement: Supplementary file 6 — Source data Fig. 5 [file 44318_2025_602_MOESM6_ESM.zip › Fig 5/K/si169_2.tif]

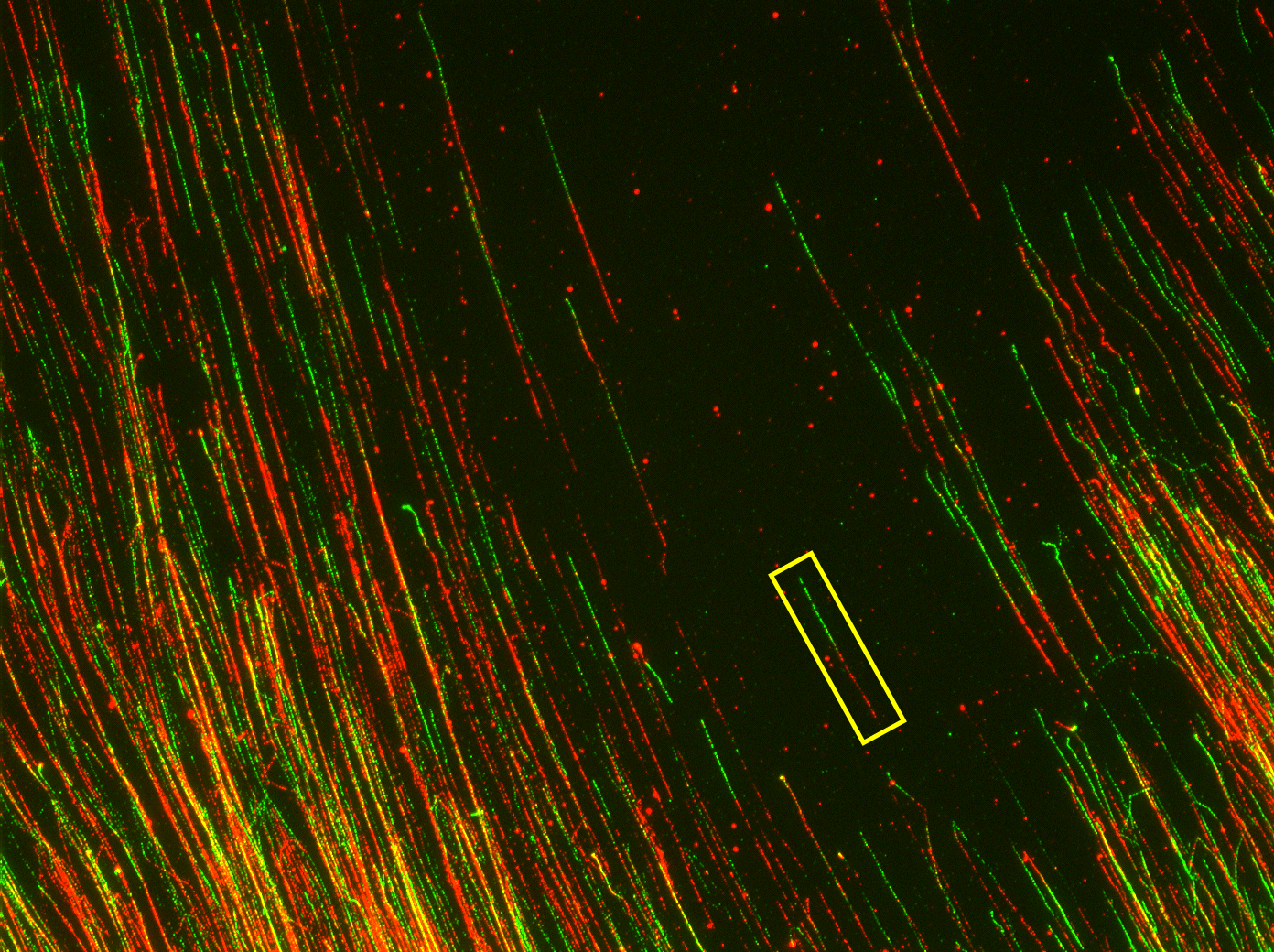

Supplement: Supplementary file 6 — Source data Fig. 5 [file 44318_2025_602_MOESM6_ESM.zip › Fig 5/K/siLUC.tif]

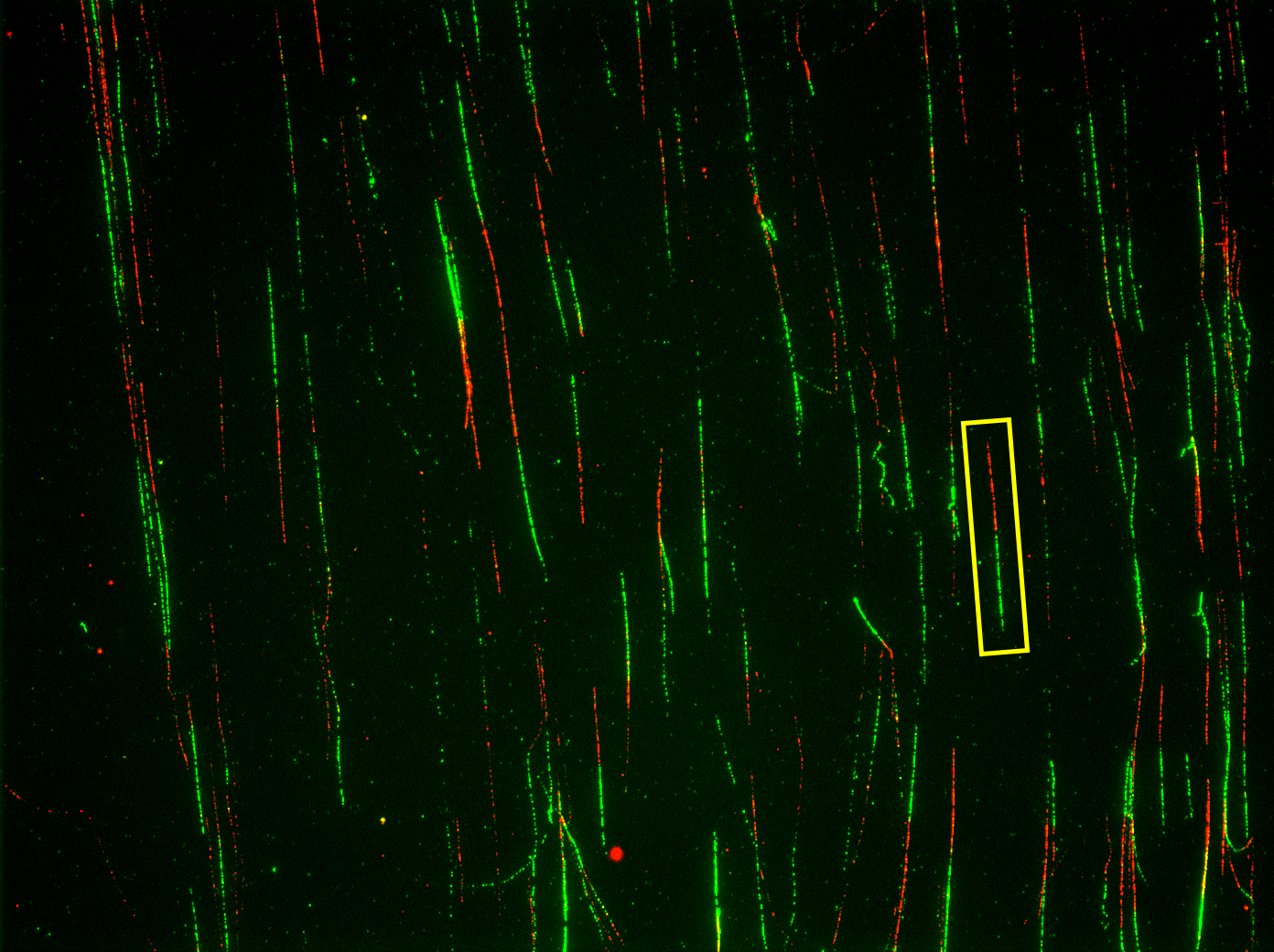

Supplement: Supplementary file 6 — Source data Fig. 5 [file 44318_2025_602_MOESM6_ESM.zip › Fig 5/K/si20+169_2.tif]
