# Supplementary material for: H2BK120ub and its reader RNF169 sequentially regulate replication fork remodeling and stability
Source: EMBO J. 2025 Oct 27;44(22):6598–625. doi: 10.1038/s44318-025-00602-1 (PMC12623888; doi:10.1038/s44318-025-00602-1)
Supplement: Supplementary file 7 — Expanded View Figures [file 44318_2025_602_MOESM7_ESM.pdf]

# Expanded View Figures

**Figure EV1. H2BK120ub transiently accumulates at the stressed DNA replication forks in an ATR-dependent manner (related to Fig. 1).**

(**A**, **B**) Immunoblot analysis of U2OS cell extracts upon siLUC and siRNF20 using the indicated antibodies. (**C**, **D**) Representative images (**C**; scale bars: 5 µm) and quantitative analysis of EdU-labeled U2OS cells upon siLUC and siRNF20, stained with H2BK120ub, DAPI and EdU antibodies. Quantification of the average nuclear H2BK120ub signal intensity per EdU-positive and EdU-negative cells (at least 120 cells were analyzed per condition; ****$P < 0.0001$, ***$P < 0.001$, ns nonsignificant, *P*(siRNF20 EdU pos. vs siRNF20 EdU neg) = 0.0002; *P* values were calculated by one-way ANOVA, followed by Kruskal–Wallis test). Bars represent median values. $n = 3$ experiments. (**E**, **F**) Scatter plot representing the H2BK120ub signals (**E**) or the H2BK120ub SIRF (**F**) per nucleus throughout the cell cycle, based on the total DAPI intensity and EdU mean intensity, of siLUC or siRNF20-treated cells as in Fig. 1A. Each dot represents an individual nucleus. Dots are colored according to increasing average H2BK120ub or H2BK120ub SIRF signal intensities. (**G**, **H**) Immunoblot of U2OS cell extracts, using the indicated antibodies (**G**), and H2BK120ub SIRF analysis (**H**) upon transfection of different siRNAs, optionally treated with HU (4 mM, 2 h). At least 150 cells were analyzed per condition. ****$P < 0.0001$, *P* values determined using one-way ANOVA coupled with Kruskal–Wallis test. $n = 3$ experiments. (**I**, **J**) Immunoblot of U2OS cell extracts, using the indicated antibodies, and RNF20 SIRF analysis upon siLUC and siRNF20, optionally treated with CPT and HU as in Fig. 1A. At least 120 EdU-positive cells were analyzed per condition. ****$P < 0.0001$, ns nonsignificant, *P* (siLUC UT vs. siLUC CPT) > 0.9999, *P* (siLUC UT vs. siLUC HU) > 0.9999, *P* (siRNF20_1 UT vs. siRNF20_1 HU) > 0.9999; *P* values were determined by one-way ANOVA followed by Kruskal–Wallis test. (**K**) Whiskers plot indicating H2BK120ub signal intensity per individual EdU-labeled chromatin fiber in untreated and HU-treated (4 mM, 1 h) U2OS cells. **$P = 0.0025$; *P* values were determined using the Mann–Whitney *U*-test (nonparametric). Box plots show the 25th, 50th (median), and 75th percentiles; whiskers indicate the 10th and 90th percentiles; "+" represents the mean. $n = 3$ experiments. (**L**) Whiskers plot indicating H3K9me3 signal intensity per individual EdU-labeled chromatin fiber in untreated and HU-treated (4 mM, 1 h) U2OS cells, upon siLUC and siRNF20. ****$P < 0.0001$, ns nonsignificant, *P* (siLUC UT vs siRNF20_1 UT) = 0.2372, *P* (siLUC HU vs siRNF20_1 HU) > 0.9999; *P* values were determined using Kruskal–Wallis test, followed by Dunn's multiple comparisons test. Box plots show the 25th, 50th (median), and 75th percentiles; whiskers indicate the 10th and 90th percentiles; "+" represents the mean. $n = 3$ experiments. (**M**) RNF20 mRNA levels were quantified by qPCR in U2OS cells transfected with siLUC or siRNF20. (**N**, **O**) Scatter plot representing the H2BK120ub SIRF per nucleus throughout the cell cycle, based on the total DAPI intensity and EdU mean intensity, of cells treated as in Fig. 1J, K, respectively.

 © The Author(s)

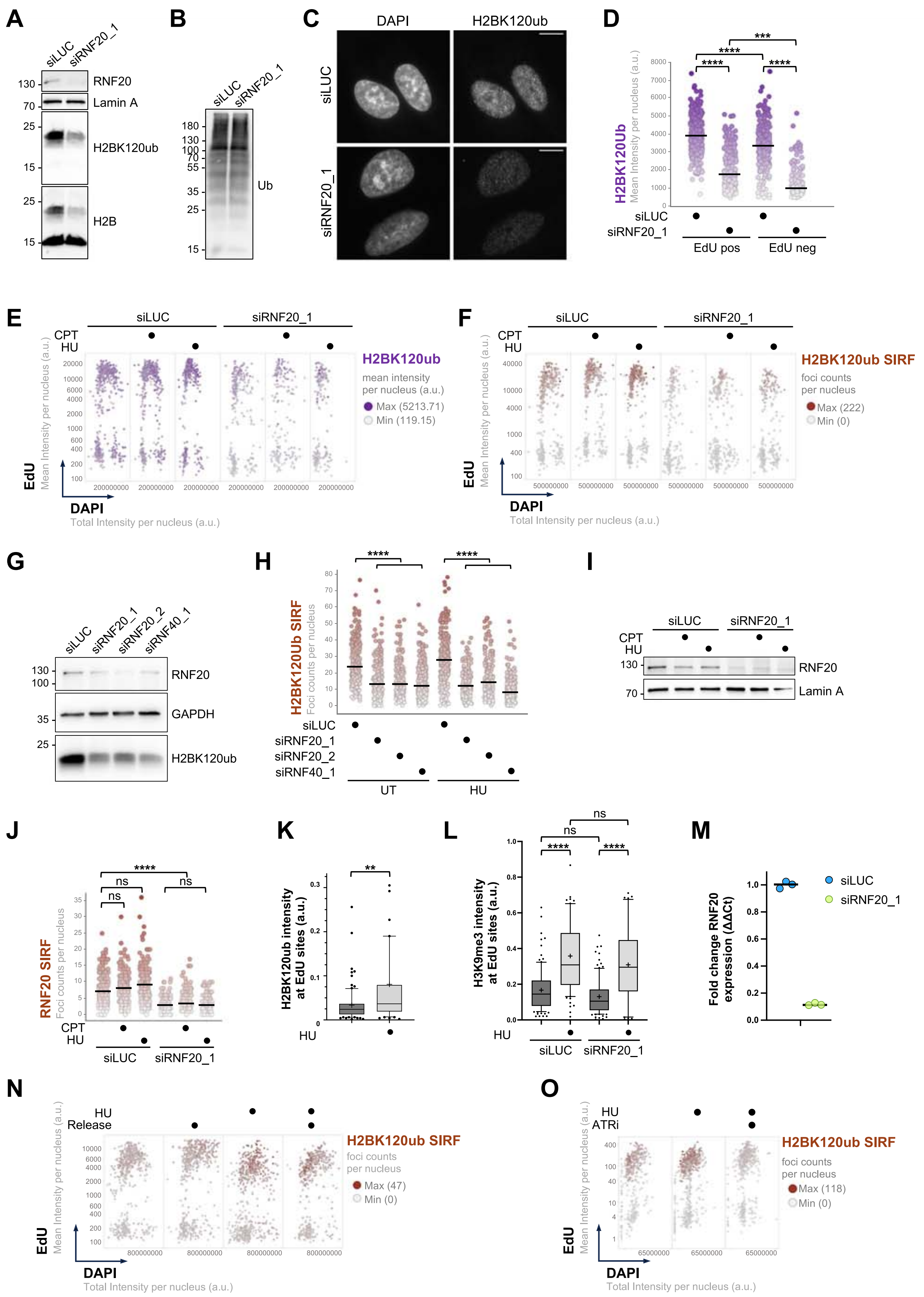
A
siLUC
siRNF20_1
RNF20
Lamin A
H2BK120ub
H2B
B
Ub
C
DAPI
H2BK120ub
siLUC
siRNF20_1
D
H2BK120Ub
Mean Intensity per nucleus (a.u.)
EdU pos
EdU neg
E
CPT
HU
H2BK120ub
mean intensity per nucleus (a.u.)
Max (5213.71)
Min (119.15)
EdU
DAPI
Total Intensity per nucleus (a.u.)
F
H2BK120ub SIRF
foci counts per nucleus
Max (222)
Min (0)
G
siRNF20_2
siRNF40_1
GAPDH
H
H2BK120Ub SIRF
Foci counts per nucleus
UT
HU
I
J
RNF20 SIRF
K
H2BK120ub intensity at EdU sites (a.u.)
L
H3K9me3 intensity at EdU sites (a.u.)
M
Fold change RNF20 expression (ΔΔCt)
N
Release
Max (47)
O
ATRi
Max (118)

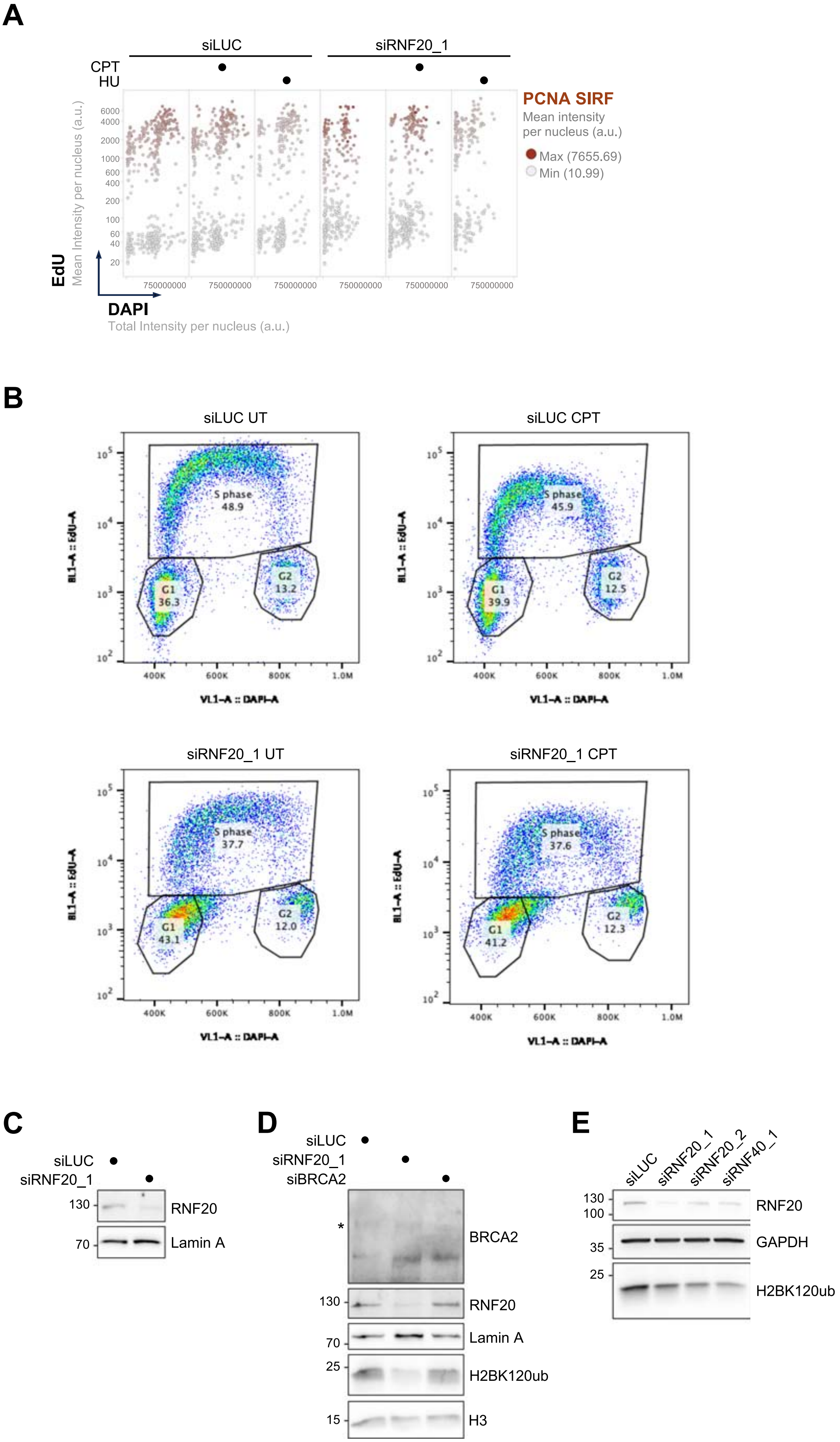
A
siLUC
siRNF20_1
CPT
HU
PCNA SIRF
Mean intensity per nucleus (a.u.)
Max (7655.69)
Min (10.99)
EdU
Mean Intensity per nucleus (a.u.)
DAPI
Total Intensity per nucleus (a.u.)
B
siLUC UT
siLUC CPT
siRNF20_1 UT
siRNF20_1 CPT
S phase 48.9
G1 36.3
G2 13.2
S phase 45.9
G1 39.9
G2 12.5
S phase 37.7
G1 43.1
G2 12.0
S phase 37.6
G1 41.2
G2 12.3
BL1-A :: EdU-A
VL1-A :: DAPI-A
C
siLUC
siRNF20_1
RNF20
Lamin A
D
siLUC
siRNF20_1
siBRCA2
BRCA2
RNF20
Lamin A
H2BK120ub
H3
E
siLUC
siRNF20_1
siRNF20_2
siRNF40_1
RNF20
GAPDH
H2BK120ub

 © The Author(s)

**Figure EV2. Loss of RNF20/H2BK120ub leads to unrestrained fork progression without major disruption of the cell cycle (related to Fig. 2).**

(**A**) Scatter plot representing the PCNA SIRF per nucleus throughout the cell cycle, based on the total DAPI intensity and EdU mean intensity, of cells treated as in Fig. 2B. Each dot represents an individual nucleus, with color coding reflecting the increasing intensity of PCNA SIRF intensity. (**B**) Flow cytometry analysis of the EdU incorporation (5 µM, 30 min) by control and U2OS cells depleted of RNF20 in unperturbed conditions or upon mild replicative stress (100 nM CPT 30 min pretreatment + 30 min during EdU labeling). $n = 3$ experiments. (**C**) Immunoblot analysis of RNF20 levels in siLUC- and siRNF20-transfected U2OS cells related to Fig. 2C. (**D**) Immunoblot analysis using the indicated antibodies of cell extracts upon siLUC, siRNF20 or siBRCA2 related to Fig. 2D. The asterisk indicates the size of BRCA2. (**E**) Immunoblot analysis using the indicated antibodies of extracts of U2OS cells upon siLUC, siRNF20_1, siRNF20_2, or siRNF40_1 related to Fig. 2F.

© The Author(s)

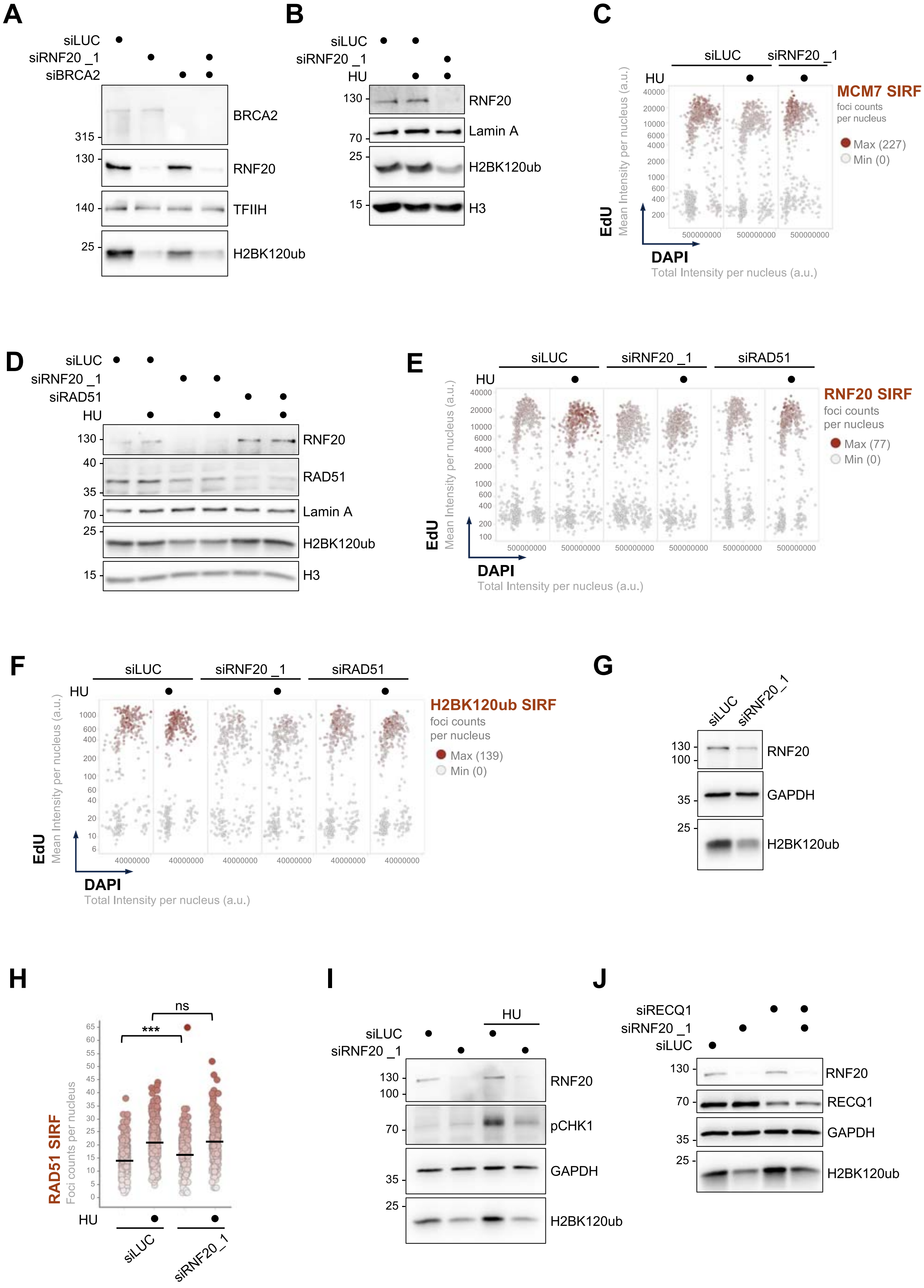
A
siLUC
siRNF20 _1
siBRCA2
315
130
140
25
BRCA2
RNF20
TFIIH
H2BK120ub
B
siLUC
siRNF20 _1
HU
130
70
25
15
RNF20
Lamin A
H2BK120ub
H3
C
siLUC
siRNF20 _1
HU
MCM7 SIRF
foci counts
per nucleus
Max (227)
Min (0)
EdU
Mean Intensity per nucleus (a.u.)
DAPI
Total Intensity per nucleus (a.u.)
D
siLUC
siRNF20 _1
siRAD51
HU
130
40
35
70
25
15
RNF20
RAD51
Lamin A
H2BK120ub
H3
E
siLUC
siRNF20 _1
siRAD51
HU
RNF20 SIRF
foci counts
per nucleus
Max (77)
Min (0)
EdU
Mean Intensity per nucleus (a.u.)
DAPI
Total Intensity per nucleus (a.u.)
F
siLUC
siRNF20 _1
siRAD51
HU
H2BK120ub SIRF
foci counts
per nucleus
Max (139)
Min (0)
EdU
Mean Intensity per nucleus (a.u.)
DAPI
Total Intensity per nucleus (a.u.)
G
siLUC
siRNF20_1
130
100
35
25
RNF20
GAPDH
H2BK120ub
H
ns
***
RAD51 SIRF
Foci counts per nucleus
HU
siLUC
siRNF20_1
I
HU
siLUC
siRNF20 _1
130
100
70
35
25
RNF20
pCHK1
GAPDH
H2BK120ub
J
siRECQ1
siRNF20 _1
siLUC
130
70
35
25
RNF20
RECQ1
GAPDH
H2BK120ub

 © The Author(s)

**Figure EV3. Loss of RNF20/H2BK120ub perturbs replication fork dynamics (related to Fig. 3).**

(**A**) Immunoblot of U2OS cell extracts, using the indicated antibodies, upon transfection with siLUC, siRNF20_1 or siBRCA2. Related to Fig. 3A. (**B**) Immunoblot analysis using the indicated antibodies of extracts of U2OS cells transfected with siLUC and siRNF20_1, optionally treated with HU. Related to Fig. 3C. (**C**) Scatter plot representing the MCM7 SIRF per nucleus throughout the cell cycle, based on the total DAPI intensity and EdU mean intensity, of cells treated as in Fig. 3C. Each dot represents an individual nucleus, with color coding reflecting the increasing intensity of MCM7 SIRF intensity. (**D**) Immunoblot of U2OS cell extracts, using the indicated antibodies, upon transfection with siLUC, siRNF20_1 or siRAD51. Related to Fig. 3E,F. (**E**, **F**) Scatter plot representing the RNF20 SIRF and H2BK120ub per nucleus throughout the cell cycle, based on the total DAPI intensity and EdU mean intensity, of cells treated as in Fig. 3E,F, respectively. Each dot represents an individual nucleus, with color coding reflecting the increasing intensity of SIRF intensity. (**G**) Immunoblot of U2OS cell extracts, using the indicated antibodies, upon transfection with siLUC and siRNF20_1. (**H**) Scatter plot representing the RAD51 SIRF per nucleus throughout the cell cycle, based on the total DAPI intensity and EdU mean intensity, upon siLUC and siRNF20_1. Each dot represents an individual nucleus, with color coding reflecting the increasing intensity of RAD51 SIRF intensity. At least 150 EdU-positive cells were analyzed per condition. ***$P < 0.001$, ns nonsignificant, $P$ (siLUC HU vs. siRNF20_1 HU) > 0.9999, $P$ (siLUC UT vs. siRNF20_1 UT) = 0.0009; $P$ values were determined using one-way ANOVA followed by Kruskal–Wallis test. (**I**) Immunoblot analysis using the indicated antibodies of extracts of U2OS cells transfected with siLUC and siRNF20_1, optionally treated with HU. Related to Fig. 3I. (**J**) Immunoblot analysis using the indicated antibodies of extracts of U2OS cells transfected with siLUC, siRNF20_1 or siRECQ1. Related to Fig. 3G.

© The Author(s) 

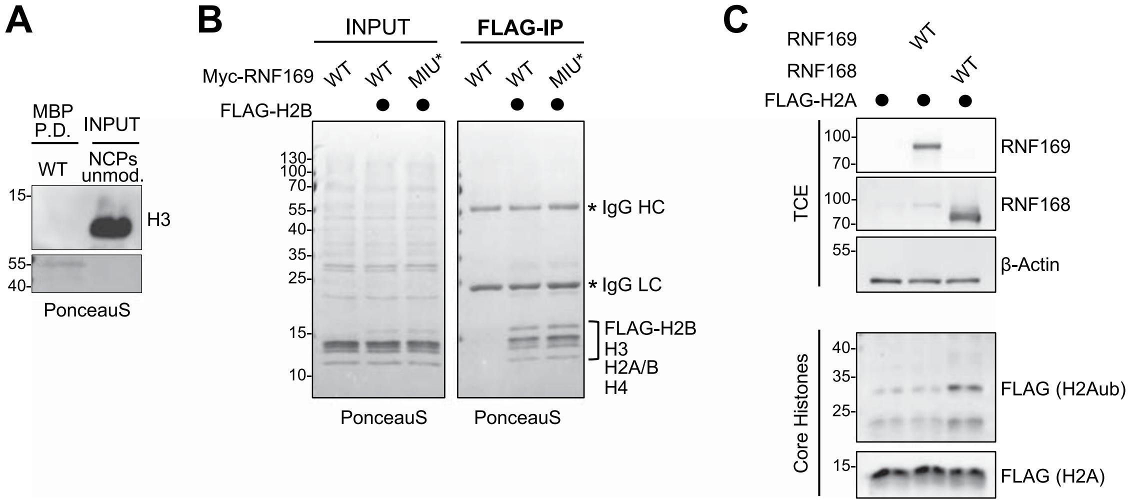


**Figure EV4. RNF169 is a reader of H2BK120ub (related to Fig. 4).**

(**A**) Pulldown assay using the C-terminal domain of RNF169 (amino acids 662–708) and unmodified nucleosome core particles (NCPs), followed by immunoblot. (**B**) Ponceau S staining of the membranes in Fig. 4D. (**C**) Immunoblot analysis on TCE and histone extracts of HEK293T cells transiently transfected with the indicated plasmids. $n = 3$ experiments.

 © The Author(s)

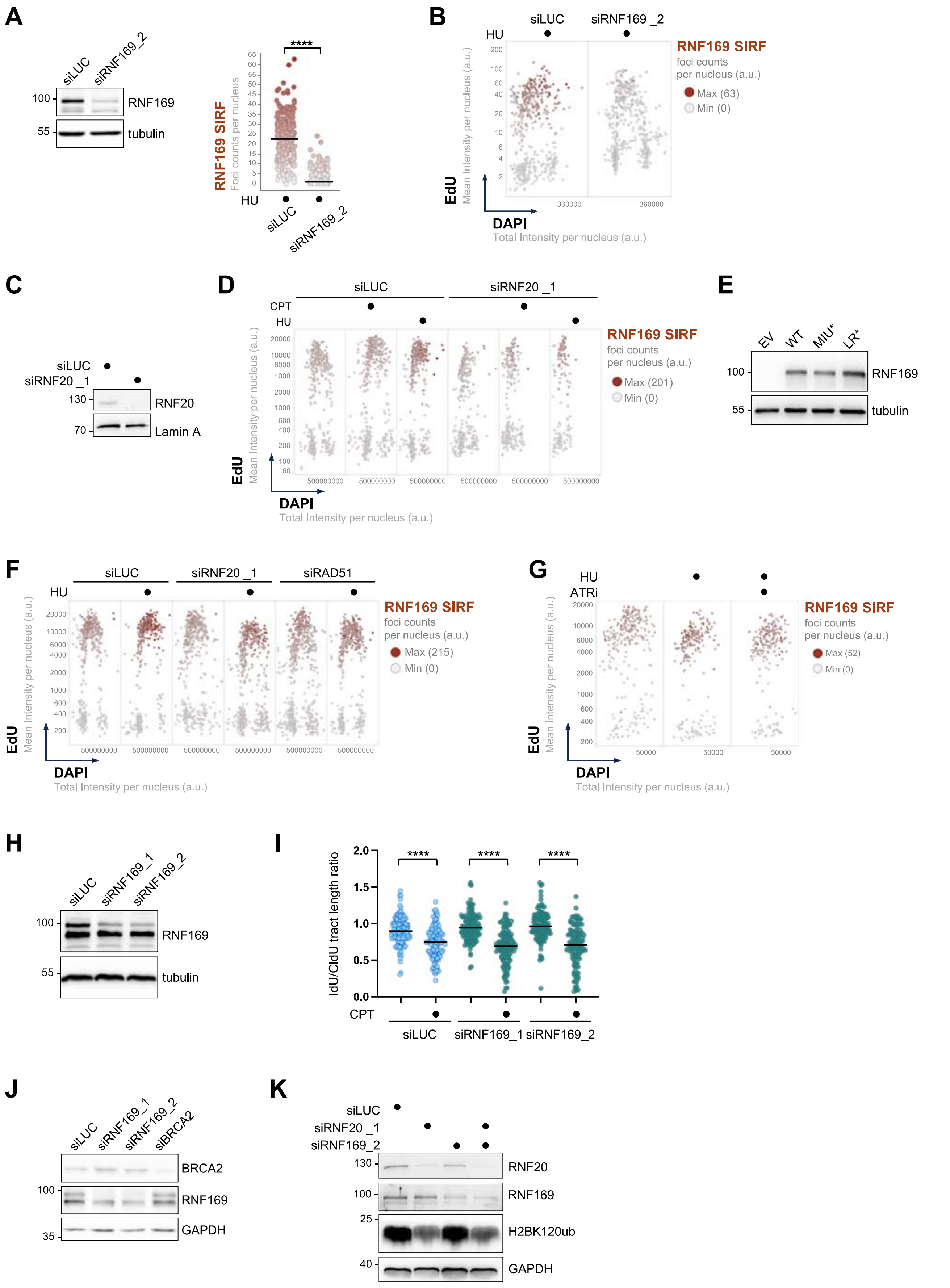
A
siLUC
siRNF169_2
100
RNF169
55
tubulin
****
RNF169 SIRF
Foci counts per nucleus
HU
B
siLUC
siRNF169 _2
HU
RNF169 SIRF
foci counts
per nucleus (a.u.)
Max (63)
Min (0)
EdU
Mean Intensity per nucleus (a.u.)
DAPI
Total Intensity per nucleus (a.u.)
C
siLUC
siRNF20 _1
130
RNF20
70
Lamin A
D
siLUC
siRNF20 _1
CPT
HU
RNF169 SIRF
foci counts
per nucleus (a.u.)
Max (201)
Min (0)
E
EV
WT
MIU*
LR*
100
RNF169
55
tubulin
F
siLUC
siRNF20 _1
siRAD51
HU
RNF169 SIRF
foci counts
per nucleus (a.u.)
Max (215)
Min (0)
G
HU
ATRi
RNF169 SIRF
foci counts
per nucleus (a.u.)
Max (52)
Min (0)
H
siLUC
siRNF169_1
siRNF169_2
100
RNF169
55
tubulin
I
IdU/CldU tract length ratio
****
CPT
siLUC
siRNF169_1
siRNF169_2
J
siLUC
siRNF169_1
siRNF169_2
siBRCA2
BRCA2
100
RNF169
35
GAPDH
K
siLUC
siRNF20 _1
siRNF169_2
130
RNF20
100
RNF169
25
H2BK120ub
40
GAPDH

**Figure EV5. RNF169 is recruited to the stressed DNA replication forks via H2BK120ub to limit fork resection (related to Fig. 5).**

(**A**, **B**) RNF169 immunoblot and RNF169 SIRF analysis upon optional treatment with HU (4 mM, 2 h) in U2OS upon siLUC and siRNF169 transfection. Quantification of the RNF169 SIRF foci in EdU-positive nuclei in indicated conditions (at least 120 EdU-positive cells analyzed per condition; ****$P < 0.0001$, $P$ value calculated using unpaired *t*-test). Bars indicate median values. $n = 3$ experiments. Color code in B indicates RNF169 SIRF foci numbers in individual nuclei across the cell cycle. (**C**, **D**) Immunoblot on U2OS cell extracts, using the indicated antibodies, upon transfection with siLUC, siRNF20_1 (**C**), and scatter plot representing the RNF169 SIRF per nucleus throughout the cell cycle, based on the total DAPI intensity and EdU mean intensity (**D**), of cells treated as in Fig. 5B. Each dot represents an individual nucleus, with color coding reflecting the increasing intensity of SIRF. (**E**) Immunoblot on U2OS cell extracts upon transient expression of the Myc-tagged forms of RNF169 (related to Fig. 5C). (**F**, **G**) Scatter plot representing the RNF169 SIRF per nucleus throughout the cell cycle, based on the total DAPI intensity and EdU mean intensity, of cells treated as in Fig. 5D,E, respectively. Each dot represents an individual nucleus, with color coding reflecting the increasing intensity of SIRF intensity. (**H**, **I**) U2OS cells were transfected with siLUC, siRNF169_1 and siRNF169_2 to assess replication fork progression in unperturbed conditions and following mild replicative stress (CPT 100 nM, 30 min; **I**) as in Fig. 2E,F (at least 115 fibers analyzed per condition; ****$P < 0.0001$; $P$ values were determined using Mann–Whitney *U*-test. Bars represent median values). Immunoblot on U2OS cell extracts reveals RNF169 protein levels (**H**). $n = 2$ experiments. (**J**, **K**) Immunoblot on U2OS cell extracts upon transfection with the indicated siRNAs, related to Fig. 5J–L.

 © The Author(s)
